# Abstracts From PVRI 2026 Dublin

**DOI:** 10.1002/pul2.70315

**Published:** 2026-06-05

## A001 Targeting ZIP12 to Modulate Intracellular Zinc and Vascular Dysfunction in Pulmonary Arterial Hypertension

Mr Iyobel Kibreab^1^, Dr Chien‐Nien Chen^1^, Dr Stephanie Hopley^2^, Dr Richard Butt^2^, Prof Martin Wilkins^1^, Prof Lan Zhao^1^

^1^Imperial College London, London, United Kingdom, ^2^Apollo Therapeutics Limited, Cambridge, United Kingdom

Pulmonary arterial hypertension (PAH) is a progressive vascular disorder characterized by excessive pulmonary vasoconstriction and remodeling of peripheral pulmonary arteries. Disruption of intracellular zinc homeostasis has emerged as a potential contributor to disease pathogenesis, yet its mechanistic role remains poorly understood. Previous work from our laboratory identified upregulation of the zinc transporter ZIP12 in the pulmonary vasculature of patients with idiopathic PAH (IPAH). ZIP12 facilitates the influx of zinc into the intracellular space, and its dysregulation is associated with pathological cellular behaviors such as proliferation, migration, and metabolic reprogramming. In this study, we investigated the effects of ZIP12 inhibition using APL‐9796, a novel humanised monoclonal antibody on intracellular labile zinc levels, cellular proliferation, and vasoconstrictive responses using pulmonary artery fibroblasts (PAAFs) derived from IPAH patients and pulmonary smooth muscle cells (PASMCs). Western blot analysis confirmed ZIP12 upregulation in IPAH‐derived PAAFs and in PASMCs exposed to hypoxia or platelet‐derived growth factor (PDGF). High‐throughput FRET‐based zinc imaging revealed elevated intracellular labile zinc in these cells, which was significantly attenuated by APL‐9796 treatment. BrdU incorporation assays demonstrated that APL‐9796 reduced hyperproliferation in IPAH PAFs and PDGF/hypoxia‐stimulated PASMCs. APL‐9796 also reduced the stress fibre formation and myosin light chain phosphorylation, indicating suppression of contractile signalling. In engineered pulmonary artery tissues (EPATs), APL‐9796 mitigated PDGF‐induced vasoconstriction, supporting its functional relevance in a 3D vascular model.

Collectively, these findings identify ZIP12 as a key regulator of intracellular zinc homeostasis and a driver of proliferative and vasoconstrictive phenotypes in PAH. Targeting ZIP12 with APL‐9796 restored zinc balance and reversing vascular dysfunction in PAH.

## A002 Role of The Protein Ttk in the Vascular Remodeling Processes in Pulmonary Artery Hyerpenstion

Dr Yann Grobs^1^, Sarah‐Éve Lemay, Magalie Boucher, Maud Fillion, Alice Bourgeois, Manon Mougin, Marie‐loup Guenette, Coralie Bilodeau, Jessica Tremblay, François Potus, Olivier Boucherat, Steeve Provencher, Sebastien Bonnet

^1^Iucpq

Pulmonary arterial hypertension (PAH) is a complex and incurable disease characterized by obliteration of distal pulmonary arteries (PAs), due to exaggerated proliferation of PA smooth muscle cells (PASMCs) from the wall, leading to heart failure. Despite advances in treatment, the prognosis for patients with PAH remains poor, emphasizing the need for new therapeutic targets. Integrative analysis of three independent RNA‐sequencing datasets identified the protein threonine and tyrsine kinase (TTK) consistently overexpressed in PAH‐PASMCs versus controls. TTK is a kinase known for its crucial role as regulator of mitosis sustaining cancer cell proliferation and vascular remodeling in coronary artery disease. Its established involvement in cellular metabolism and gene regulation, both processes that support PASMC proliferation, TTK emerges as a promising therapeutic target in PAH. We therefore hypothesize that TTK is overexpressed in PAH, contributing to pulmonary vascular remodeling by sustaining the hyperproliferative phenotype of PAH‐PASMCs.

Our preliminary data demonstrated that TTK expression was elevated in human PAH‐PASMCs and in PAs from monocrotaline and Sugen‐hypoxia rat models. In PAH‐PASMCs, pharmacological inhibition using CFI‐402257 and molecular knockdown with silencer RNA (siTTK) significantly reduced protein levels of proliferation markers (MCM2, PLK1) and survivin by Western blot, decreased proliferation (Ki67 immunofluorescence), and increased apoptosis (Annexin V). RNA sequencing and phosphoproteomics analysis of PAH‐PASMCs treated with CFI‐402257 is ongoing to elucidate the molecular pathways impacted by TTK inhibition. In human precision‐cut lung slices from controls stimulated with growth factors cocktail (endothelin‐1, PDGF‐BB, FGF2), TTK inhibition improves vascular remodeling. In vivo, the inhibition of TTK with CFI‐402257 improves pulmonary hemodynamics parameters by decreasing mean pulmonary artery pressure and right ventricle systolic pressure and improves cardiac function by increasing cardiac output in Sugen‐hypoxia rats.

This project aims to validate TTK as a novel therapeutic target for PAH, potentially transforming current treatment strategies and improving long‐term outcomes for patients.

## A003 Developing A Novel Prognostic Risk Score for Sarcoidosis‐Associated Pulmonary Hypertension

Dr Christopher Chew^1,2,3,4^, A/Prof Athénaïs Boucly^5,6,7^, Prof Konstantinos Dimopoulos^3,4^, Prof Marc Humbert^5,6,7^, Dr Xavier Jaïs^5,6,7^, Prof Olivier Sitbon^5,6,7^, Dr Vasilis Kouranos^3,8^, Prof Athol Wells^3,8^, Dr Colm McCabe^3,4^, Dr Heba Nashat^3,4^, Prof Stephen John Wort^3,4^, Dr Laura Price^3,4^, Prof Laurent Savale^5,6,7^

^1^Department of Respiratory Medicine, Alfred Health, Melbourne, Australia, ^2^Department of Respiratory Medicine, Northern Health, Melbourne, Australia, ^3^National Heart and Lung Institute, Imperial College, London, United Kingdom, ^4^National Pulmonary Hypertension Service, Royal Brompton Hospital, Chelsea, United Kingdom, ^5^School of Medicine, Université Paris‐Saclay, Le Kremlin‐Bicêtre, France, ^6^Pulmonary Hypertension National Referral Center, Department of Respiratory and Intensive Care Medicine, Bicêtre Hospital, Assistance Publique ‐ Hôpitaux de Paris, Le Kremlin‐Bicêtre, France, ^7^INSERM UMR_S 999, Marie Lannelongue Hospital and Bicêtre Hospital, Le Kremlin‐Bicêtre, France, ^8^Interstitial Lung Diseases Unit, Royal Brompton Hospital, Chelsea, United Kingdom

Risk stratification is crucial in managing pulmonary hypertension (PH). However, no validated prognostic tools exist for sarcoidosis‐associated PH (SAPH), a Group 5 PH subgroup with high mortality. We aimed to evaluate the prognostic performance of the 2022 ESC/ERS model and to develop and externally validate a novel, simple risk score for SAPH. We conducted a retrospective analysis of two independent cohorts of patients with invasively confirmed pre‐capillary SAPH from the French (n = 300) and Royal Brompton, UK (n = 128) PH registries. After assessing the prognostic utility of the 2022 ESC/ERS model, a new risk score was developed in the French cohort using multivariable Cox regression and subsequently validated in the UK cohort. Performance was evaluated using time‐dependent receiver operating characteristic curves (timeROC AUC) and Kaplan‐Meier analysis, using R Statistical Software (v4.4.3; R Core Team 2024). In the combined cohorts (n = 428), the 2022 ESC/ERS risk model showed poor prognostic discrimination (C‐index 0.56, log‐rank p = 0.10). We subsequently developed a novel six‐variable, points‐based score (maximum 17 points) comprising: age > 55 (+3), male sex (+3), forced vital capacity < 45% (+3), 6‐minute walk distance < 400 m (+4), mean pulmonary arterial pressure > 55 mmHg (+3), and TLCO < 25% (+1). The score demonstrated good discrimination in the derivation cohort (timeROC AUC at 1 and 3 years of 0.75 and 0.79, respectively), with performance preserved on external validation. Stratification into low (≤ 4 points), intermediate‐ (5‐8 points), and high‐risk (> 8 points) groups predicted markedly distinct survival outcomes. The estimated 1‐year survival was 97% in the low‐risk group, compared to 87% and 79% in the intermediate and high‐risk groups, respectively (log‐rank p < 0.0001). Existing standard PH risk stratification models appear to perform poorly in SAPH. Our novel, externally validated, disease‐specific risk score effectively risk stratifies patients and may help guide clinical decision‐making and patient counselling, through estimation of likely prognosis.

## A004 A Broncho‐centric Reticulation Pattern Dominates in a Tertiary Referral Sarcoidosis‐Associated Pulmonary Hypertension Cohort

Dr Christopher Chew^1,2,3,4,5,6^, Dr Bhavin Rawal^5,7^, Prof Athol Wells^4,6^, Dr Vasilis Kouranos^4,6^, Dr Colm McCabe^4,5^, Dr Heba Nashat^4,5^, Dr Stephen John Wort^4,5^, Prof Sujal Desai^4,6,7^, Dr Laura Price^4,5^

^1^Department of Respiratory Medicine, Alfred Health, Melbourne, Australia, ^2^Department of Respiratory Medicine, Western Health, Melbourne, Australia, ^3^Department of Respiratory Medicine, Northern Health, Melbourne, Australia, ^4^National Heart and Lung Institute, Imperial College, London, United Kingdom, ^5^National Pulmonary Hypertension Service, Royal Brompton Hospital, Chelsea, United Kingdom, ^6^Interstitial Lung Disease Unit, Royal Brompton Hospital, Chelsea, United Kingdom, ^7^United Kingdom, Chelsea, United Kingdom

Sarcoidosis‐associated pulmonary hypertension (SAPH) is a severe complication, most associated with advanced pulmonary sarcoidosis. We applied a recent novel, multinational Delphi consensus classification for sarcoidosis HRCT phenotypes to a large, RHC‐verified single‐centre SAPH cohort to establish phenotype prevalence and evaluate associations with disease severity and survival.

Retrospective analysis of all patients diagnosed with SAPH via right heart catheterization (RHC) and expert multi‐disciplinary team consensus at a UK National Pulmonary Hypertension Centre, with thoracic CT imaging within one year of RHC. Baseline demographics, lung function, and survival data were collected. Two independent reviewers (thoracic radiologist and respiratory physician, both trained on the Delphi criteria) scored CT scans, resolving disagreements by consensus. Inter‐observer agreement was assessed using Cohen's Kappa. All analyses were performed using R Statistical Software (v4.4.3; R Core Team 2024). 131 patients (median age 59, 50% male) were included. Initial inter‐observer agreement for all 15 phenotypes was fair (Kappa 0.37), improving to moderate (Kappa 0.41) with phenotype grouping, reflecting the novel classification's complexity. A broncho‐centric reticulation pattern was the dominant consensus phenotype (n = 85, 65%). This phenotype was associated with significantly lower percent predicted FVC (median 57 ± 2% vs. 67 ± 3% SEM; p = 0.03) and TLCO (median 28 ± 1% vs 39 ± 3% SEM; p = 0.02) and increased main pulmonary artery diameter (median 38 ± 1 mm vs 34 ± 1 mm SEM; p < 0.01). There were no statistically significant differences in baseline demographics, RHC haemodynamics or survival. A broncho‐centric reticulation CT pattern was the dominant phenotype in this large, well‐characterised SAPH cohort and was associated with significantly worse lung function. While the modest inter‐observer agreement for the full classification highlights its complexity, our study provides initial, real‐world evidence for clinical utility of the novel phenotyping framework. Future prospective studies are required to determine if this phenotype is a risk factor for the development of PH in sarcoidosis patients.

## A005 IMPROVE‐PAH: A Phase 2b, Randomized, Double‐Blind, Placebo‐Controlled Trial of IKT‐001 in Pulmonary Arterial Hypertension (PAH)

Dr. Vallerie V. McLaughlin^1^, Dr. Kelly Chin^2^, Dr. Robert Frantz^3^, Dr. Hossein A. Ghofrani^4^, Dr. Marius Hoeper^5^, Dr. Marc Humbert^6^, Dr. Rogerio Souza^7^, Dr. John Adams^8^, Dr. Chris Cabell^8^, Head Of Clinical Development Allison Widlitz^8^

^1^University of Michigan, Ann Arbor, US, ^2^University of Texas Southwestern, Dallas, US, ^3^Mayo Clinic, Rochester, US, ^4^Justus‐Liebig University Giessen and Marburg Lung Center, Giessen, Germany, ^5^Hannover Medical School, Hannover, Germany, ^6^Université Paris‐Saclay, Paris, France, ^7^University of Sao Paulo, Sao Paulo, Brazil, ^8^Inhibikase Therapeutics, Wilmington, US

Pulmonary arterial hypertension (PAH) is a progressive, life‐threatening disease characterized by pulmonary vascular remodeling and elevated pulmonary vascular resistance (PVR). IKT‐001 is a novel oral prodrug of imatinib, designed to improve tolerability while inhibiting PDGFR and c‐Kit signaling involved in abnormal vascular remodeling. Prior studies of imatinib demonstrated efficacy in PAH but were limited by adverse events. To evaluate the efficacy, safety, and pharmacokinetics of two doses of IKT‐001 versus placebo in adults with WHO Group 1 PAH and WHO functional class II–III symptoms. In this Phase 2b, multi‐center, randomized, double‐blind, placebo‐controlled trial, approximately 150 participants will be randomized 1:1:1 to receive 300 mg IKT‐001, 500 mg IKT‐001, or placebo once daily for 26 weeks, in addition to stable background PAH therapy. The primary efficacy endpoint is change in PVR at Week 26. Secondary endpoints include 6‐minute walk distance (6MWD), WHO functional class, and pharmacokinetics. Exploratory endpoints include echocardiographic and hemodynamic measures, NT‐proBNP, IL‐6, EmPHasis‐10 score, and incidence of clinical worsening events. Safety will be assessed by the incidence and severity of treatment emergent adverse events. The IMPROVE‐PAH study will assess whether IKT‐001 offers a clinically meaningful reduction in PVR with acceptable tolerability in patients with moderate to severe PAH. Results will inform the potential of IKT‐001 as a novel anti‐proliferative treatment option targeting vascular remodeling pathways in PAH.

## A006 Autophagy Regulator GABARAPL2 Modulates Endothelial Function in Pulmonary Arterial Hypertension

Miss Aeman Ansari^1^, Dr Rehab J. Alharbi^1^, Dr Nadia Fernandes^2^, Ms Nathalie Lambie^2^, Dr Felicia New^3^, Dr Harry J. Whitwell^4^, Professor Allan Lawrie^1^, Professor Beata Wojciak‐Stothard^1^

^1^National Heart and Lung Institute (NHLI), Imperial College London, London, UK, ^2^NIHR Imperial BRC Genomics Facility, Imperial College London, London, UK, ^3^Bruker Spatial Biology, Bruker Corporation, Seattle, USA, ^4^Division of Systems Medicine, Imperial College London, London, UK

Pulmonary arterial hypertension (PAH) is a progressive disease marked by vascular remodelling, leading to elevated pulmonary pressure and right heart failure. Despite higher female prevalence, survival rates are paradoxically better in women. Endothelial dysfunction – driven by aberrant proliferation, inflammation, and metabolic reprogramming – plays a key role, though mechanisms remain unclear. Impaired autophagy, particularly mitophagy, contributes to mitochondrial dysfunction and a glycolytic metabolic shift in PAH. Spatial transcriptomic analysis of PAH vasculature revealed an upregulation of several autophagy regulators, with GABARAPL2 being the highest upregulated gene in PAH, with female‐specific prominence. GABARAPL2 mediates endothelial dysfunction in PAH. To investigate GABARAPL2's role in autophagy, mitochondrial homeostasis, and its potential crosstalk with oestrogen signalling. GABARAPL2 was overexpressed or silenced in human pulmonary artery endothelial cells (HPAECs) under PAH‐relevant stress conditions induced by hypoxia and TNF‐α. Endothelial viability, proliferation, autophagy, barrier function, and mitochondrial respiration (oxygen consumption, membrane potential, superoxide levels) were assessed. Proteomic analysis of GABARAPL2‐overexpressing and oestrogen‐treated HPAECs, was performed to characterise associated pathways and interactors and identify crosstalk between GABARAPL2 and oestrogen signalling. GABARAPL2 overexpression improved endothelial cell survival, proliferation, autophagic flux, and barrier integrity under stress. Proteomic analysis identified 317 differentially expressed proteins (DEPs), p < 0.05, logFC>log2(1.5), enriched in autophagy and mitochondrial pathways. Interactive proteomics showed interactions of GABARAPL2 with mitochondrial membrane proteins and key regulators of mitophagy. Overexpression of GABARAPL2 restored mitochondrial respiration, increased mitochondrial coverage, and reduced membrane potential and superoxide, counteracting hypoxia/TNF‐α effects. Oestradiol improved endothelial cell survival in starved cells and this effect was mediated by GABARAPL2. Pathway analysis of shared DEP datasets between oestrogen‐treated and GABARAPL2‐overexpressing HPAECs indicates common effects on mitochondrial structure and function. GABARAPL2 is a critical regulator of endothelial health in PAH, influencing autophagy, mitochondrial function, and oestrogen signalling. Its therapeutic potential requires further investigation.

## A007 Group V Pulmonary Hypertension in a Pediatric Patient: Report of a Case

Miss Ludmila Borgna^1^

^1^Sanatorio De Niños, rosario, Argentina

Pulmonary Hypertension is defined as mPAP > 20 mmHg, pulmonary capillary wedge pressure < 15 mmHg and iPVR ≥3 WU.m2 to identify pre‐capillary PH; through cardiac catheterization.

The most common pulmonary hypertension in children is associated with congenital heart disease, most of which include lung desease, PHNB (group 3) but also with left heart disease (group 2). A systematic review of the literature was performed. The search was conducted through the databases: Pubmed. Data were collected anonymously from the patient's medical history, and a descriptive analysis was performed. A 16‐year‐old female patient with autoinflammatory syndrome, juvenile rheumatoid arthritis, and celiac disease, (diagnosed 3 years ago). The last 6 months, the patient presents rigth ventricule failure, pulmonary hypertension and functional class III.

First echocardiogram shows right ventricle dysfunction and dilatation. Moderate PH, mild pericardial effusion, preserved left ventricle contractility, with paradoxical interventricle septum motion. Catheterization study reports severe hypertension. She is currently taking tadalafil, ambrisentan, selexipag, spironolactone and underlying disease treatment. Last echocardiogram shows good RV function. Moderate but improved PH. Preserved LV contractility. 6‐minute walk test: Distance 265 meters in 6 minutes; Borg 3. Improved functional class II. Treatment goals are multiple and include symptom improvement, quality of life, and survival. Objective assessments to measure treatment response include progress in exercise capacity, hemodynamic profile, and survival.

Group 5 PH consists of a complex group of associated disorders, the cause of which is usually multifactorial and secondary to increased precapillary and postcapillary pressure, as well as direct effects on the pulmonary vasculature. The true incidence is unknown; it is often associated with increased morbidity and mortality. Studies investigating this treatment are few and involve limited numbers of patients. It is generally aimed at treating the underlying disorder. Currently, no specific treatment is approved for this group

## A008 Hemodynamic Behavior and Mortality During Mechanical Ventilation

Dr. Angel Benjamin Rojas^1^, Dr Juan Carlos Vergottini

^1^National University of Cordoba, Córdoba City, Argentina

Mechanical Ventilation (MV) can induce acute pulmonary hypertension (aPH), defined as a mean pulmonary artery pressure (mPAP) ≥ 20 mmHg with increased pulmonary vascular resistance (PVR). MV settings such as FiO₂, tidal volume (VT), plateau pressure (Pplat), positive end‐expiratory pressure (PEEP), driving pressure (DP), and static compliance (Cst) may be associated with the development of aPH. General: To correlate mPAP values during the first day of MV with 30‐day mortality. Specific: (a) To correlate mPAP levels with pH, PaCO₂, shunt fraction, PaO₂/FiO₂ ratio, and MV parameters (PEEP, VT, FiO₂, Cst, DP, and Pplat). A cross‐sectional, analytical study including patients > 18 years of age of both sexes, admitted to the ICU of the Hospital Nacional de Clínicas, monitored with right heart catheterization during the first day of MV between 2021 and 2024. Statistical analysis included case description and inferential bivariate analysis (Wilcoxon tests, Spearman's correlation, and ROC curve analysis). A total of 18 patients were included. Sixty‐one percent were male. Mean age was 68.39 ± 10.24 years, BMI 30 ± 9.71 kg/m², duration of MV 11.78 ± 11.41 days, and APACHE II score 18.94 ± 5.02 points. Survivors (n = 3) had an mPAP of 20.67 ± 2.08 mmHg, whereas non‐survivors (n = 15) had an mPAP of 24.4 ± 2.64 mmHg (p = 0.001). ROC analysis yielded an AUC of 0.844, sensitivity 100%, specificity 66.67%, and an optimal cutoff value of 22.6 mmHg. mPAP correlated with pH (p = 0.04), PaCO₂ (p = 0.01), PaO₂ (p = 0.02), shunt fraction (p = 0.04), wedge pressure (p = 0.01), PVR (p = 0.045), and Cst (p = 0.02). In patients under MV, the development of acute pulmonary hypertension was associated with higher mortality, metabolic acidosis, hypoxemia, increased shunt fraction, elevated PVR, and increased wedge pressures and Cst.

## A009 Pulmonary Vasodilation With Tpip in Rat Isolated Perfused Lungs

Dr Tam Nguyen^1^, Christina Chang, David Cipolla, Mary Kelly, Zhili Li, Vladimir Malinin, Walter Perkins, Veronica Viramontes, Junguo Zhou, Michel Corboz

^1^Insmed Incorporated, Bridgewater, United States

Treprostinil palmitil inhalation powder (TPIP) is developed for treatment of pulmonary arterial hypertension (PAH) and pulmonary hypertension associated with interstitial lung disease (ILD‐PH). Treprostinil palmitil (TP), a prodrug with extended lung residence time in preclinical in vivo studies,¹ is converted by lung enzymes to treprostinil (TRE), which in turn produces sustained pulmonary vasodilation.² In this ex vivo lung study, we examined the pharmacodynamics and kinetics of various TPIP formulations. Adult rat lungs were isolated and maintained at 37°C. The pulmonary vasculature was perfused with a modified Krebs‐Henseleit buffer without recirculation. Phenylephrine (PHE, 1 µM) was added in the perfusate to elevate pulmonary artery pressure (PAP). TPIP formulations containing 0.5%, 2%, or 4% TP were administered via the trachea at about 18.9 nmol TP per lung (~48 µg TP per kg bodyweight), while ~29.7 nmol TRE was tracheal‐instilled in a control group. PAP was then monitored for 3 hours. Despite the lack of perfusate recirculation, all TPIP formulations reversed PHE‐induced PAP elevation. There was no difference in the reversal rate or extent among the TPIP formulations. The decreases in PAP were significant 30 minutes after administration and maintained for at least 2.5 additional hours. In contrast, tracheal TRE administration resulted in an immediate PAP decrease which lasted for less than 1 hour. TPIP formulations with 0.5%, 2%, or 4% TP provided sustained pulmonary vasodilation with minimal or no contribution from TRE in the pulmonary vasculature in ex vivo rat perfused lungs, consistent with previous in vivo observations.¹

References:

1. Chapman et al. Pulm Pharmacol Ther (2018) 49:104–111.

2. Ismat et al. Adv Ther (2022) 39:5144–5157.

## A010 Twelve Months of Therapeutic Progress: Our Journey With Sotatercept

Mrs. Debra Kittel^1^, Mrs. Emily Garber^1^, Ms. Michaela Schlegel^1^, Mrs. Lauren Boehler^1^, Dr. Scott Visovatti^1^, Dr. Andrew Wodarcyk^1^, Dr Curtis Daniels^1^, Dr. Saurabh Rajpal^1^, Dr. Elie Homsy^1^, Dr. Veronica Franco^1^

^1^The Ohio State University, Columbus, United States of America

We evaluated our institutional practice patterns following the incorporation of Sotatercept into the management of patients with pulmonary arterial hypertension (PAH). We began prescribing Sotatercept following FDA approval in March 2024. While all providers followed manufacturer recommendations for initiation and management of Sotatercept, provider‐to‐provider variations in some aspects of patient care were identified; these real‐world experiences with Sotatercept are of great interest to the PH community. This retrospective analysis of the practice patterns within our program was performed using de‐identified data from patients initiated on Sotatercept during the first year following FDA approval. Forty‐three patients with group 1 PAH were initiated on Sotatercept during the period studied. All patients had been on standard‐of‐care background therapies, with most (32) on triple therapy involving prostacyclin analogues, endothelin receptor antagonists, and either a PDE‐5 inhibitor or sGC stimulator. Hemoglobin and platelet counts were monitored every 3 weeks for the first five doses, and then every 3 to 12 weeks thereafter. We identified fifteen dose reductions due to polycythemia or thrombocytopenia. There were no dose reductions due to major bleeding or thromboembolic events. Echocardiograms, 6MWT, NT Pro‐BNP and functional class were performed every 3‐6 months after initiation. Prostacyclins were reduced or discontinued in 11 patients. Five patients were transitioned from parenteral prostacyclin therapy to oral prostacyclin. While Sotatercept has been successfully integrated into clinical practice at OSU, variability persists in key aspects of management, including laboratory monitoring intervals, timing of reassessment testing, and dose adjustments of Sotatercept as well as background therapies. To address these discrepancies, we propose a structured paradigm designed to standardize initiation, monitoring, and dose modification of Sotatercept, with incorporation into the electronic medical record to ensure consistency and facilitate longitudinal evaluation.

## A011 Proteomic Comparison of Rabbit Fetal Pulmonary Arteries and Ductus Arteriosus by Mass Spectroscopy Reveals Significant Metabolic Differences that may Underlie their Opposing Oxygen Responses

Dr. Rachel Bentley^1^, Dr. Kurt Prins^2,3^, Ashley Martin^1^, Dr. Elahe Alizadeh^1,4^, Benjamin Ott^1,4^, Isaac Emon^1^, Dr. Charles Hindmarch^1,4,5^, Dr. Stephen Archer^1,4^

^1^Department of Medicine, Queen's University, Kingston, Canada, ^2^Medical University of South Carolina, Charleston, USA, ^3^Director of Gazes Cardiac Research Institute, Charleston, USA, ^4^Queen's Cardiopulmonary Unit, Translational Institute of Medicine (TIME), Kingston, Canada, ^5^Department of Biomedical and Molecular Sciences (DBMS), Queen's University, Kingston, Canada

Transitioning from fetal to neonatal circulation occurs rapidly at birth, relying on the opposing oxygen responses of two key vessels. The ductus arteriosus (DA) connects the fetal pulmonary artery (PA) and aorta, diverting placentally oxygenated blood away from developing lungs to the systemic circulation. This shunting is supported by constriction and remodelling of small PA in fetal lungs, increasing vascular resistance. Increased arterial oxygen at birth induces simultaneous PA vasodilation, perfusing the newly ventilated lungs, and DA constriction, separating the pulmonary and systemic circulations. The underlying mechanism of the opposing oxygen responses is not fully understood. A failure of either vessel to appropriately respond to oxygen results in clinical complications: persistent pulmonary hypertension of the newborn or persistent ductus arteriosus. To examine the underlying differences in these fetal vessels, we performed proteomics on fetal rabbit DA and PA. DA and PA oxygen responsiveness was confirmed via microCT. Whole DA and PA were isolated from male (n = 4) and female (n = 4) term (30 days gestation) fetal NZ white rabbits. Protein was extracted and labelled with TMTpro mass tag labelling. Following HPLC fractionation, samples were run on the ThermoFisher Orbitrap Eclipse Mass Spectrometer and analyzed using ThermoFisher Proteome Discoverer software and DESeq. 2 differential expression analysis. There were 3013 significantly differentially expressed proteins between the DA and PA (padj< 0.05). KEGG pathway analysis revealed metabolic differences. The most significantly enriched pathways were “Starch and Sucrose Metabolism” (map00500) in the DA and “Sulfur Metabolism” (map00920) in the PA. There was also PA enrichment of “Fatty Acid Degradation” (map00071), “Pyruvate Metabolism” (map00620), and “Glycolysis/Gluconeogenesis” (map00010). Functional assessment of metabolic consequences of these proteomic differences via micropolarimetry is pending. We identified novel metabolic differences between the DA and PA, which may underlie the opposing oxygen‐induced mitochondrial redox responses that initiate vasoconstriction of these vessels.

## A012 Dynamic Monitoring of Right Ventricular Function in Acute Pulmonary Embolism Using AI‐Enhanced ECG Analysis

Dr. Dingyi Wang^1^, Dr Feiya Xu^1^, Dr Jing Ma^1^, Dr Guohui Fan^1^, Professor Shenda Hong^2^, Professor Zhenguo Zhai^1^

^1^China‐Japan Friendship Hospital, Beijing, China, ^2^Peking University, Beijing, China

Right ventricular dysfunction (RVD) is the major risk of short‐term mortality and long‐term sequelae in acute pulmonary embolism (PE). About 30% of patients retain RVD at 1 year, yet its recovery trajectory remains unclear. We aimed to characterize dynamic changes in right ventricular function in intermediate‐/high‐risk PE using continuous ECG monitoring with artificial intelligence (AI)‐based analysis. In this prospective multicenter cohort, 60 patients with confirmed intermediate‐/high‐risk PE were enrolled across four tertiary hospitals. Exclusion criteria included symptom onset > 14 days before diagnosis, chronic PE without recurrence, and contraindications to ECG patch. Patients received either reperfusion (systemic thrombolysis or catheter‐directed therapy) and anticoagulation only. Continuous ECG signals were recorded using patch‐type monitors for 72 h within the first 5 days after admission. ECG frequency‐domain features were decomposed via convolutional neural networks (CNNs), and temporal modeling was applied to analyze dynamic changes. Within 72 h, heart rate, P‐wave amplitude, QRS amplitude, QT interval, and T‐wave amplitude increased, while P‐wave width, QRS duration, ST‐segment amplitude, and T‐wave width decreased. RR interval declined after 48 h; PR interval remained stable. Heart rate variability (HRV) indices (SDNN, vitality index, RMSSD, NN_50%) progressively improved, with decreasing LF/HF ratio, indicating enhanced parasympathetic activity. Compared with intermediate‐low risk patients, intermediate‐high risk group showed wider P waves, higher QRS amplitude, longer RR/PR/ST intervals, more pronounced ST depression, higher HRV indices, and lower LF/HF. Reperfusion group (n = 10) showed lower HRV indices (total power, SDNN, SDANN, RMSSD) than anticoagulation group, suggesting faster autonomic stabilization. Dynamic ECG and HRV demonstrate progressive recovery within 72 h after acute PE, with parasympathetic activation as a marker of improvement. Intermediate‐high risk patients display more severe disturbances, while reperfusion accelerates stabilization. AI‐assisted ECG offers a practical, non‐invasive approach to monitor RVD, complementing echocardiography and guiding clinical management.

## A013 Bridging Gaps in Pulmonary Arterial Hypertension Care in the Gulf Region: Insights on Diagnostic Delays and Access Challenges

MD Abdallah Aldalaan, MD Khaled Saleh^1^, Mr Mohamed Safwat

^1^Cleveland Clinic Abudhabi, Abudhabi, United Arab Emirates

To examine time to diagnosis, treatment pathways, and access barriers for patients with pulmonary arterial hypertension (PAH) across Gulf Cooperation Council (GCC) countries, and to identify opportunities to improve patient outcomes and health system responsiveness. A retrospective chart review was conducted across PH referral centers in Saudi Arabia (n = 181), Oman (n = 25), United Arab Emirates (n = 24), and Qatar (n = 20). Patients diagnosed between January 2018–December 2022 with ≥ 12 months’ follow‐up were included. Data on demographics, time to diagnosis, treatment patterns, and referrals for advanced interventions were analyzed. Among 250 patients (mean age 40.3 ± 14.0 years; 82.0% female), the average time from symptom onset to confirmed diagnosis was 1.6 ± 2.4 years, highlighting delays in care. At initial assessment, 54.7% were on monotherapy; postreferral to PH specialized centers, 81.8% escalated to combination therapy, including 45.8% to triple therapy. Despite these efforts, 17.6% of patients remained high risk, 7.2% were referred for lung transplantation, and only 2.4% received a transplant. Mortality reached 22% in the high‐risk group, reflecting the serious outcomes of delayed care and limited access to advanced interventions.

This multi‐country review demonstrates that delayed diagnosis and late referral remain major barriers in PAH care across the Gulf region. Despite treatment intensification at specialized centers, many patients experienced disease worsening, reflecting the challenges of managing PAH once patients present at more advanced stages. The prolonged time to diagnosis observed also suggests potential under diagnosis in the wider population, as some patients may never be identified or referred. Addressing these critical gaps requires coordinated efforts to expand PAH awareness among community and healthcare providers to support earlier identification and to establish structured referral pathways, supported by strong policy and funding commitments to ensure timely access to life‐saving care, ultimately improving outcomes for patients living with PAH.

## A014 The Epidemiological and Clinical Burden of Pulmonary Arterial Hypertension in Latin America and the Caribbean: A Systematic Literature Review

Dr Victoria Wurcel^1^, Natalia Tassara^1^, Amanda Bittencour^2^, Haliton Alves^2^, Hafez Antonio^3^, Claudia Folino^1^, Jose Rincon^3^, Felype Castelhano^2^, Ashley Humphries^4^, Ina Zile^4^, Dr Jacqueline Pavia, Fraser Williams^4^

^1^MSD Argentina, Buenos Aires, Argentina, ^2^MSD Brazil, São Paulo, Brazil, ^3^MSD Mexico, Ciudad de Mexico, Mexico, ^4^Adelphi Values PROVE, Bollington, United Kingdom

Pulmonary arterial hypertension (PAH) is a rare progressive, chronic disease, which, can lead to right heart failure and death. The burden of PAH across Latin America and the Caribbean (LatAM) is poorly understood. This systematic literature review (SLR) sought to assess the epidemiological and clinical disease burden of PAH in this region. Systematic literature searches of Embase, MEDLINE, Evidence‐Based Medicine Reviews, and LatAM Literature on Health Sciences databases were conducted in English, Spanish and Portuguese from 2015 to 2025. Identified articles were independently screened and extracted by two reviewers. This SLR identified 49 studies describing the epidemiological and clinical burden of PAH, patient population sizes ranged between 11 to 1,604 persons. Most identified studies (N = 21) reported on Brazilian patients. Evidence for other countries was limited, Mexico (N = 7), Argentina (N = 6), Chile (N = 4), the Caribbean (N = 2), Colombia (N = 1), Peru (N = 1) and Uruguay (N = 1). Epidemiology of PAH was fairly uniform across LatAm, with age‐standardized prevalence and incidence rates of 2.39‐3.25 and 0.33‐0.55 per 100,000 persons, respectively, which is comparable to the global rates (2.28 and 0.52 per 100,000 persons). Age‐standardized PAH‐related mortality rates varied, greater in the Tropical Latin America region (Brazil and Paraguay) compared to Central America (0.32 vs 0.08 per 100,000 persons); the global rate being 0.27 per 100,000 persons. Idiopathic PAH (IPAH) was the most reported subtype (21 studies). Patients typically presented with PAH World Health Organization (WHO) functional class (FC) II or III (27/33 studies). WHO FC II/III correspond to an intermediate mortality risk, where these patients typically require treatment escalation. Our study examines the epidemiological and clinical burden of PAH in LatAM. The burden of disease varies by country, some showing similar burden to global rate, while others show lesser burden, probably underreporting. However, information is scarce for a large part of LatAM.

## A015 Vascular Smooth Muscle Cell‐Derived Osteoprotegerin Drives Pulmonary Arterial Hypertension Pathogenesis Via AKT signalling

Dr Merve Keles^1^, Dr Laura E West^2^, Ms Nadine D Arnold^2^, Dr Astrid Weiss^3^, Ms Nadiya V Mykytyuk^1^, Ms Julia Baldauf^3^, Dr Josephine A. Pickworth^2^, Dr Amira A Zawia^2^, Dr Helena A Turton^2^, Dr Megan F Allen^2^, Dr Clemens Ruppert^3^, Dr A.A Roger Thompson^2^, Professor Sheila E Francis^2^, Professor Ralph T Shermuly^3^, Professor Allan Lawrie^1^

^1^Faculty of Medicine, National Heart and Lung Institute, Imperial College London, London, United Kingdom, ^2^Division of Clinical Medicine, University of Sheffield, Sheffield, United Kingdom, ^3^Department of Internal Medicine, German Center for Lung Research (DZL), Excellence Cluster Cardio‐Pulmonary Institute (CPI), Institute for Lung Health (ILH), Justus Liebig University, Giessen, Germany, ^4^UGMLC/DZL Giessen Biobank, Justus Liebig University, Giessen, Germany

Pulmonary arterial hypertension (PAH) is a progressive vasculopathy characterized by vasoconstriction and remodelling of distal pulmonary arteries. Osteoprotegerin (OPG, Tnfrsf11b), a TNF superfamily member, is upregulated in PAH and correlates with disease severity. Global deletion of Opg or monoclonal antibody blockade attenuates disease in preclinical models, however, cellular source and downstream mechanisms remain unclear. To investigate the cell‐specific role of OPG, we generated conditional Tnfrsf11b deletion using OPGfl/fl mice crossed with PGKcretg/+ (global‐knockout), LysMcretg/+ (myeloid), Myl2cretg/+ (cardiomyocytes), Cdh5cretg/+ (endothelial cells), smMHCcretg/+ (vascular smooth muscle cells, VSMCs), Col1a2cretg/+ (fibroblasts) drivers. Knockouts were induced by tamoxifen injection and PAH was established using Sugen5416/hypoxia model. Cardiopulmonary phenotyping included echocardiography, catheterisation, and histological analysis. Mechanistic studies in VSMCs were performed using peptide‐based kinase activity profiling (PamGene platform), analysing 380 kinases in patient‐derived PASMCs treated with an anti‐OPG antibody. PGK‐driven deletion of Opg has phenocopied the global knock‐out in reducing PAH phenotype, while myeloid‐ or cardiomyocyte‐specific deletion had no effect. Endothelial‐specific deletion reduced muscularisation but did not improve RVSP or PVR. Strikingly, VSMC‐specific Opg deletion significantly improved haemodynamics and attenuated vascular remodelling. Notably, tissue levels of OPG, but not serum, correlated with haemodynamic parameters and vascular remodelling. Myography showed no change in vasoreactivity, following VSMC‐specific Opg deletion indicating OPG's effects are driven by proliferation and phenotypic modulation. Kinome‐wide profiling revealed OPG‐induced activation of PI3K‐AKT and autophagy‐related pathways, with AKT1 emerging as a central hub. Anti‐OPG antibody treatment suppressed AKT1 phosphorylation in IPAH PASMCs and lung tissue from SuHx rats. OPG blockade also reduced kinases linked to apoptosis, stress, and senescence, consistent with its role as part of senescence‐associated secretory phenotype (SASP).

Conclusion: VSMC‐derived OPG drives pulmonary vascular remodelling and haemodynamic deterioration in PAH via AKT1‐dependent signalling. OPG remains a promising therapeutic target for PAH.

## A016 STOP‐RIO: A Randomized Controlled Non‐Inferiority Trial to Test the Safety of Discontinuation of Riociguat After Balloon Pulmonary Angioplasty in Chronic Thrombo‐Embolic Pulmonary Hypertension

Drs Tamara Rodenburg^1^, Drs. Sanne Boerman^2^, Prof. dr. Marco Post^3^, Prof. dr. Harm Jan Bogaard^1^, Dr. Jurjan Aman^1^

^1^Amsterdam Cardiovascular Sciences, Pulmonary Hypertension and Thrombosis, Amsterdam UMC location Vrije Universiteit Amsterdam, The Netherlands, ^2^Department of Respiratory Medicine, St. Antonius Hospital Nieuwegein, The Netherlands, ^3^Department of Cardiology, St. Antonius Hospital Nieuwegein, The Netherlands

Chronic thromboembolic pulmonary hypertension (CTEPH) is characterized by persistent pulmonary vascular obstruction and microvasculopathy. Approximately half of patients undergo balloon pulmonary angioplasty (BPA), frequently combined with riociguat. While this approach achieves favorable outcomes, there is no consensus on whether riociguat should be continued once hemodynamics improve. Current guidelines allow discontinuation after “successful” BPA, yet success remains undefined and patient selection unclear, leading to heterogeneous practice and prolonged riociguat treatment despite adverse effects and high annual costs (€33–51k per patient). The Japanese THERAPY‐HYBRID‐BPA trial demonstrated that maximal exercise cardiac output reduced after riociguat discontinuation post‐BPA, however this was not accompanied by significant decreases in mean pulmonary artery pressure (mPAP), six‐minute walk distance (6MWD), or other safety concerns. Structured riociguat discontinuation has not been evaluated in Western populations. We aim to assess the safety and feasibility of riociguat discontinuation after BPA in patients achieving mPAP < 30 mmHg (primary objective). Secondary objectives include evaluation of economic and societal impact, and patient‐reported outcomes. This multicentre, randomized, open‐label, non‐inferiority trial will enroll 74 adults with CTEPH from the two Dutch expert centres (Amsterdam UMC and St. Antonius Hospital). Eligible patients must have completed their final BPA ≥ 6 months prior and demonstrate mPAP < 30 mmHg by right heart catheterization. Participants will be randomized 1:1 to riociguat discontinuation or continuation. Non‐inferiority will be tested at 16 weeks in mPAP (primary endpoint), with secondary hierarchical endpoints including NT‐proBNP and 6MWD. Additional endpoints include other hemodynamics, cardiopulmonary exercise testing, echocardiography, and quality‐of‐life measures. A mid‐study safety evaluation (8 weeks) will assess predefined clinical worsening criteria, and allow re‐initiation of riociguat if required. This trial will be the first to prospectively evaluate riociguat discontinuation after BPA in a Western population, aiming to provide guidance for long‐term CTEPH management while reducing treatment burden and healthcare costs.

## A017 Validation and Prognostic Significance of MRI Derived Stroke Volume in Pulmonary Arterial Hypertension

Dr Daniel Taylor^1^, Dr Samer Alabed^1,2^, Dr Hamza Zafar^1,2^, Prof Alex Rothman^1,2^, Dr Alireza Hokmabadi^1^, Dr Michael Sharkey^1^, Dr Krit Dwivedi^1^, Dr Abdul Hameed^1^, Dr Athanasios Charalampopoulos^2^, Prof Roger Thompson^1,2^, Dr Robin Condliffe^2^, Prof Andy Swift^1,2^, Prof David Kiely^1,2^

^1^University Of Sheffield, ^2^Sheffield Teaching Hospitals

Four‐strata risk stratification is recommended to guide treatment in patients with pulmonary arterial hypertension (PAH) at follow‐up. Recent work has shown stroke volume index (SVI) obtained at right heart catheterisation (RHC) can be used to refine risk stratification in patients at intermediate‐low and intermediate‐high risk of mortality. The aim of this study was to examine whether SVI derived from MRI (SVIMRI) may be an alternative to RHC. We included patients from the ASPIRE registry with PAH who underwent cardiac MRI, suitable for analysis, at diagnosis and first follow‐up assessment. MRI images were analysed with a tensor‐based machine learning approach, which automatically measures ventricular volumes to compute left ventricular stroke volume and indexes these to body surface area. Bland‐Altman analysis was used to evaluate agreement between SVIMRI and SVIRHC. We included 373 patients and paired SVIMRI values were successfully computed for 335 (89.8%) patients. Mean SVIMRI significantly improved from baseline (34.5 mL/m2 □20.9) to follow‐up (41.9 mL/m2 □11.2, p < 0.0001). SVIMRI correlated SVIRHC (r = 0.77, p < 0.0001), with a mean bias of ‐4.0 mL/m2 (95% CI ‐17.6 to 9.6). Between baseline and follow‐up, there was a significant relationship between change in SVIMRI and incremental shuttle walking distance (r = 0.214, p = 0.0133). For patients with improvements in SVIMRI, this relationship was characterised by a 4.3 m increase in shuttle distance for every 1 mL/m2 improvement in SVIMRI. For those whose SVIMRI score decreased at follow‐up, there was no effect on walking distance (p = 0.647). There was a significant difference in one‐year mortality in patients with SVIMRI > 37 mL/min (compared to SVIMRI ≤ 37 mL/m2) at follow‐up (log rank p = 0.0169). SVIMRI correlates with SVIRHC. Further work will explore whether SVIMRI and other MRI metrics may further refine risk stratification at follow‐up and be an alternative to RHC for risk refinement in patients at intermediate‐low and intermediate‐high risk of mortality.

## A018 N Terminal Pro‐Brain Natriuretic Peptide And Serum Uric Acid As Predictors Of Prognosis In Patients Of Congenital Heart Disease With Severe Pulmonary Artery Hypertension Considered Inoperable: A Prospective Observational Study

Anita Saxena^1^, Dr Devender Yadav^1^, Professor KS Laller^1^, Professor Ashwani Kumar^1^

^1^Fortis Escorts Heart Institute, New Delhi, India, New Delhi, India

Introduction: In patients with Eisenmenger syndrome, some reports have published association of high serum uric acid and N‐Terminal Pro‐Brain natriuretic peptide (NT Pro‐BNP) with lower survival. We aimed to study the prognostic value of serum uric acid and NT Pro‐BNP levels in patients of CHD associated with severe PAH who were considered inoperable due to advanced PVOD. We prospectively followed 30 such patients. Besides clinical assessment, SaO2, 6MWD, a detailed echocardiography was performed to assess RV function. Serum uric acid and NT pro‐BNP estimations were also done. Medical therapy with oral pulmonary vasodilators (either as monotherapy or dual therapy) was started as per treating physician's discretion; she was blinded to the results of investigations, other than echocardiography. All the parameters were reassessed at 6 months and then at one year. Of the total 30 patients, 20 were > 20 years, 15 were males. 24 had Eisenmenger syndrome, 6 had severe PAH, four with atrial septal defect and two with patent ductus arteriosus. Over a follow up (FU) of one year, 22 patients remained clinically stable (group A), 8 patients required hospitalization for heart failure (group B). None of the patients died. The basal WHO functional class, 6MWD, SaO2, TAPSE on echocardiography were abnormal in both groups. The levels of NT pro‐BNP and serum uric acid were also abnormally high in both the groups. On a FU of one year, significant improvement was seen in all these parameters in group A. In contrast, Group B showed no significant change in any parameter, including NT Pro‐BNP and serum uric acid profile.

Absence of fall in elevated NT‐proBNP and uric acid levels on FU is associated with hospitalization for heart failure. These parameters could act as indicator for identifying high risk patients and the need for more aggressive therapy.

## A019 Pulmonary Artery to Aorta Ratio, Pulmonary Vascular Resistance, and Their Relationship to Survival in Pvdomics Participants with Copd and/or Interstitial Lung Disease

Dr. Matthew Jankowich^1^, Mr. Matthew Borgia^2^, Dr. Gaurav Choudhary^1^, Dr. Jason Lempel^3^, Dr. Rahul Renapurkar^3^, Ms. Elena Desanti^2^, Dr. Sharon Rounds^1^

^1^Brown University and Providence VA Medical Center, Providence, U.S.A., ^2^Providence VA Medical Center, Providence, U.S.A., ^3^Cleveland Clinic, Cleveland, U.S.A.

We previously reported that larger pulmonary artery (PA) to aorta (PA/A) ratio is associated with higher pulmonary vascular resistance (PVR) in PVDOMICS study participants with COPD and/or interstitial lung disease (ILD). In the current analysis, we examined the association of PA/A ratio with transplant‐free survival and whether PVR was a mediator of the relationship between PA/A ratio and survival in PVDOMICS participants with COPD, ILD, or both. We analyzed data from PVDOMICS participants with COPD and/or ILD, including those with group 3 or 2/3 PH and non‐PH comparators. Survival probability (to death or transplantation (lung and/or heart)) was plotted against time, stratifying participants by PA/A ratio > 0.9 versus ≤ 0.9. Cox proportional hazards modeling assessed hazard associated with PA/A ratio> 0.9. Additional models were examined with PVR rather than PA/A ratio. Finally, association of PA/A ratio with survival and mediation effect via PVR were estimated in logistic models using the CAUSALMED procedure in SAS. Study participants(n = 235, 45% male) were age 64.8 ± 11.3 yrs; 50.6% had COPD, 12.3% IPF, 45.5% other ILDs. PA/A ratio was > 0.9 in n = 170 (mean PVR:5.2 ± 4.2 Wood Units (WUs), PVR> 2WUs: 90.2%); with PA/A ≤ 0.9 (n = 65), mean PVR was 3.2 ± 2.1WUs, PVR> 2WUs: 66.7%. Over 37.9 ± 24 months follow‐up, 57.5% died or underwent transplantation. Kaplan‐Meier survival curves showed worse survival with PA/A ratio> 0.9 (log‐rank p‐value 0.037). In an age‐, sex‐, and BMI‐adjusted model, PA/A ratio> 0.9 was associated with 70% worse survival (p = 0.011). Higher PVR was associated with 5.6% higher hazard of death or transplant (per 1 WU increment) in similarly adjusted modeling (p = 0.002). However, in causal mediation analysis, PVR was not a significant mediator of the relationship of PA/A ratio with odds of survival.

PA/A ratio> 0.9 or higher PVR is associated with worse survival in PVDOMICS participants with COPD, ILD, or both, but higher PVR does not mediate the PA/A ratio‐survival relationship.

## A020 Changes in Cardiac Effort in Pulmonary Hypertension‐Interstitial Lung Disease: Insights from the ASCENT Trial

Dr. Daniel Lachant^1^, Senior Director, Clinical Development Ashley Galloway^2^, Senior MSL Dawn Fleming^2^, Chief Medical Officer Rajeev Saggar^2^, Director, Medical Affairs Savan Patel^2^, Dr. R. James White^1^

^1^University of Rochester Medical Center, Rochester, USA, ^2^Liquidia Technologies, Morrisville, USA

Rational: [NCT06129240], Cohort A, is a prospective, multicenter, open‐label trial evaluating the safety, tolerability, and efficacy of LIQ861, an inhaled dry powder formulation of treprostinil, in pulmonary hypertension associated with interstitial lung disease (PH‐ILD). Cardiac Effort, the number of heartbeats during the 6‐minute walk test (6MWT) divided by the 6‐minute walk distance (6MWD), has been shown to have less variability than 6MWD alone, tracks with physiologic improvement, and correlates with stroke volume. We explored changes in Cardiac Effort as a novel metric of improvement after treatment with LIQ861 in PH‐ILD. During all 6MWTs, heart rate monitoring was performed using the Fourth Frontier X2, a single‐lead dry electrode electrocardiogram. Cardiac Effort at Week 8 and Week 16 were compared to baseline. The median age is 69 years (range: 29‐80), with 48% being male. Baseline measurements included a PVR of 6.0 ± 2.9 WU, mPAP of 33 ± 8 mmHg, a FVC of 65.9 ± 20.7%, a diffusing capacity of 36.2 ± 13.9%, and a 6MWD of 298.1 ± 80.29 meters. At Week 8, the baseline mean Cardiac Effort of 2.35 ± 0.82 beats/m meaningfully reduced by 10.2% to 2.11 ± 0.71 beats/m (‐0.24 ± 0.36, n = 49, p < 0.001). Additionally, at Week 16, the baseline mean Cardiac Effort of 2.27 ± 0.72 beats/m significantly reduced by 10.6% to 2.03 ± 0.60 beats/m (‐0.24 ± 0.42, n = 41, p < 0.001). The median dose of LIQ861 was 132.5 and 159 mcg QID at Weeks 8 and 16, respectively. Cardiac Effort is an easy way to provide insight into physiological stress during the 6MWT. In this open‐label prospective study, treatment with drug LIQ861 was associated with a meaningful reduction in Cardiac Effort (≥ 10%) at both Weeks 8 & 16 in patients with PH‐ILD. This reduction likely reflects improved stroke volume and oxygen delivery.

## A021 Safety and Exploratory Efficacy Data of LIQ861 Dry Powder Inhaled Treprostinil in PH‐ILD patients: ASCENT to Week 24

Dr. Rajan Saggar^1^, Dr. Ashwin Ravichandran^2^, Dr. Nicholas Kolaitis^3^, Dr. Akshay Muralidhar^4^, Dr. Jeremy Feldman^5^, Dr. Patricia George^6^, Dr. Ricardo Restrepo‐Jaramillo^7^, Dr. John Ryan^8^, Director, Medical Affairs Savan Patel^9^, Sr. Director, Clinical Development Ashley Galloway^9^, Chief Medical Officer Rajeev Saggar^9^, Dr. R. James White^10^, Dr. Valerie McLaughlin^11^

^1^UCLA Health, Los Angeles, USA, ^2^Ascension St. Vincent, Indianapolis, USA, ^3^UCSF Health, San Francisco, USA, ^4^Arizona Pulmonary Associates, Phoenix, USA, ^5^Summit Health Oregon, Bend, USA, ^6^National Jewish Health, Denver, USA, ^7^University of South Florida Health, Tampa, USA, ^8^University of Utah Health, Salt Lake City, USA, ^9^Liquidia Technologies, Morrisville, USA, ^10^University of Rochester Medical Center, Rochester, USA, ^11^University of Michigan, Ann Arbor, USA

[NCT06129240], Cohort A, is a prospective, multicenter, open‐label trial evaluating the safety, tolerability, and efficacy of LIQ861 (YUTREPIA™), an inhaled dry powder formulation of treprostinil, in PH‐ILD). Eligible patients had PH‐ILD with mPAP ≥ 21 mmHg, PCWP ≤ 15 mmHg, and PVR ≥ 3 WU. LIQ861 was dosed QID and titrated based on tolerability and clinical response. The primary endpoint was safety & tolerability. Exploratory endpoints included change from baseline in 6MWD, Dyspnea‐12, EmPHasis‐10, and simplified cough scores. 54 patients were enrolled (mean age 68.5 ± 8.9 years; 48.1% male). ILD etiologies were idiopathic interstitial pneumonias (48.1%), autoimmune ILDs (35.2%), hypersensitivity pneumonitis (1.9%), CPFE (9.3%), and other (5.6%). Baseline mean mPAP, PCWP, and PVR were 33.4 ± 8.4 mmHg, 8.6 ± 3.3 mmHg, and 6.0 ± 2.9 WU; 6MWD was 298 ± 80 m. PDE5 inhibitors were used in 13%. Median LIQ861 doses were 132.5 mcg, 159.0 mcg, and 185.5 mcg QID at Weeks 8, 16, and 24, respectively. At Week 24, median change from baseline in 6MWD was +41.0 m. 54.1% of patients improved ≥ +40 m and 40.5% improved ≥ +50 m. Dyspnea‐12 improved from 10.3 to 9.1 and EmPHasis‐10 from 23.0 to 19.9. No changes in simplified cough scores were noted from baseline to 24 weeks. Most common treatment‐related TEAEs was cough in 48.1% [92% mild] and headache in 18.5% [90% mild]. No treatment‐related SAEs occurred. Fifteen patients (27.8%) discontinued, none for drug‐related reasons. LIQ861 was well tolerated over 24 weeks, with most patients titrating to ≥ 185.5 mcg QID (comparable to ~21 breaths nebulized treprostinil). Most common TEAE was mild cough with no change in simplified cough score. In patients with PHILD, LIQ861 improved exercise capacity (+41 m) from baseline over 24 weeks.

## A022 Inclusion of all WHO Pulmonary Hypertension (PH) Groups in a Specialty PH Clinic: Outcomes From the Queen's University PH Registry

Dr Henry Lu^1^, Shannan Davis^1^, Dr Joshua Durbin^1^, Brooke Ring^1^, Dr Charles Hindmarch^1^, Dr Stephen Archer^1^

^1^Queen's University, Kingston, Canada

Pulmonary hypertension (PH) is a progressive, life‐threatening disease that leads to right heart failure and early mortality. Although the World Health Organization (WHO) recognizes five PH groups, most clinical trials and registries focus on Group 1 (pulmonary arterial hypertension, PAH) and Group 4 (chronic thromboembolic PH). This excludes the most common causes of PH: Group 2 (left heart disease), Group 3 (lung disease), or mixed etiology PH, resulting in underreported outcomes. We report findings from the Queen's University PH Registry (n = 360), which includes all WHO groups: 10% G1, 39.7% G2, 5.8% G3, 8.3% G4, and 36.1% mixed. Median ages at presentation/death were lowest in G1 (61, 66 years) compared to 70‐81.5 years in other Groups. Median follow‐up was longest in G1 (27 months) vs. 18‐23 months in other Groups. G1 and G4 also had the lowest mortality rates (6.2, 4.2 deaths/100 person‐years), compared to 11.4 (G2), 19.4 (G3), and 11.2 (mixed). Accordingly, one‐, three‐, and five‐year survival was highest for G1 (93%, 77%, 60%) and G4 PH (92%, 87%, 87%), vs. G2 (90%, 77%, 57%), G3 (84%, 49%, 25%), and mixed (90%, 77%, 60%). Hazard ratios for death (G1 as reference) were 1.56 (G2), 2.95 (G3), 1.70 (mixed), and 0.59 (G4). Therapy was guideline‐concordant, with 80% of G1 on PAH‐targeted therapies (42% macitentan, 39% sildenafil, 39% tadalafil), 51% of G2 on SGLT2 inhibitors, and 100% of G4 on oral anticoagulants (and 43% of G4 on riociguat). Local five‐year survival in G1 (70%) and G4 (87%) was non‐inferior to international registries, including the French PAH registry (52%) and the Giessen Registry (62%). Similarly, G2/3 survival was comparable to US PVDOMICS. This demonstrates high mortality for all PH Groups and suggests access to PH specialty care for all forms of PH may improve outcomes, highlighting the value of more inclusive registry/trial design.

## A023 Exposure of Pulmonary Epithelium to Diesel Exhaust Particles Results in Expression of EndoMT Transcription Factors in Co‐Cultured Pulmonary Arterial Endothelial Cells

Miss Janne Verhaegen^1^, Lucia Aversa^1^, Lynn Willems^1,2^, Allard Wagenaar^1^, Marion Delcroix^1,3^, Greetje Vande Velde^4^, Rozenn Quarck^1,3^

^1^Laboratory of Respiratory Diseases & Thoracic Surgery (BREATHE), Department of Chronic Diseases & Metabolism (CHROMETA), KU Leuven – University of Leuven, Leuven, Belgium, ^2^Department of Cell and Chemical Biology, Leiden University Medical Centre, Leiden, The Netherlands, ^3^Clinical Department of Respiratory Diseases, University Hospitals, Leuven, Belgium, ^4^Laboratory of Biomedical MRI, Department of Imaging and Pathology & MoSAIC, KU Leuven – University of Leuven, Leuven, Belgium

Chronic thromboembolic pulmonary hypertension (CTEPH) is a rare but severe complication of pulmonary embolism, characterized by persistent obstruction of pulmonary arteries due to fibro‐thrombotic material and microvasculopathy. Despite its clinical significance, the underlying mechanisms driving disease progression remain poorly understood. Air pollution has recently emerged as a potential contributor to CTEPH progression, by being associated with adverse outcome in CTEPH patients. However, the mechanisms through which air pollution influences disease progression remain unexplored. We hypothesized that exposure to air pollution could influence endothelial‐to‐mesenchymal transition (EndoMT) and contribute to the persistence of obstructing fibro‐thrombotic material in pulmonary arteries of CTEPH patients. To address this, we established an in vitro co‐culture model comprising human bronchial epithelial cells on a Transwell membrane in air‐liquid interface, reproducing a pseudostratified epithelium, combined with pulmonary arterial endothelial cells (PAEC) in the bottom well. The epithelium was exposed to diesel exhaust particles (DEP; 200 µg/mL) for 24 and 72 hours, after which RNA was extracted from PAEC, and EndoMT transcription factor mRNA expression was assessed by qRT‐PCR. Preliminary data in control PAEC showed time‐dependent regulation of EndoMT transcription factors. After short‐term exposure (24 h) of epithelium to DEP, no differences in mRNA expression were observed. However, after longer‐term exposure (72 h), Snail1 (P = 0.005), Slug (P < 0.0001), and Zeb2 (P = 0.0025) mRNA expression was significantly upregulated. Our findings indicate that prolonged exposure of epithelial cells to DEP results in enhanced expression of EndoMT transcription factors in co‐cultured PAEC. Ongoing studies using patient‐derived PAEC will further delineate whether air pollution‐driven EndoMT contributes to CTEPH progression.

## A024 Discovery of Novel Genetic Mechanisms Underlying Chronic Thromboembolic Pulmonary Hypertension

Dr Mizuki Momoi^1^, Dr Takahiro Hiraide^1^, Dr Yoshinori Katsumata^2^, Dr Atsushi Anzai^1^, Dr Masaharu Kataoka^3^, Dr Masaki Ieda^1^

^1^Department of Cardiology, Keio University School of Medicine, Tokyo, Japan, ^2^Institute for Integrated Sports Medicine, Keio University School of Medicine, Tokyo, Japan, ^3^The Second Department of Internal Medicine, University of Occupational and Environmental Health, Kitakyushu, Japan

The detailed pathogenic mechanisms of chronic thromboembolic pulmonary hypertension (CTEPH) remain unclear. We genetically investigated unexplored mechanisms underlying CTEPH. Whole exome and panel sequencing were conducted to identify both somatic and germline variants using DNA from peripheral blood cells in Japanese CTEPH patients. Clinical features and treatment responses were compared between variant‐positive and negative patients. Among 214 CTEPH patients, 20.1% had somatic variants in hematologic malignancy‐related genes, indicating clonal hematopoiesis. This frequency was higher than that in the general population. Clinically, variant carriers showed higher post‐treatment B‐type natriuretic peptide levels, higher rates of home oxygen therapy, shorter 6‐minute walk distances, and more frequent thrombus reformation events. RNA sequencing and serum analysis showed enrichment of blood coagulation and the neutrophil extracellular trap (NET) formation pathway in patients with the variant. In addition, NET‐forming cells were confirmed by immunofluorescence staining in thrombi from pulmonary arteries resected during pulmonary endarterectomy as well as in peripheral blood cells of patients with clonal hematopoiesis. Regarding germline variants, the allele frequency of the heterozygous RNF213 R4810K variant (3.2%)—an East Asian specific variant previously reported to be associated with pulmonary artery stenosis and pulmonary arterial hypertension—was markedly higher than that in the Japanese general population (0.84%). Variant carriers were younger, had more severe hemodynamics, and more frequently exhibited poor subpleural perfusion on pulmonary angiography, indicating microvascular dysfunction. They also showed higher pulmonary arterial pressure after catheter therapy, suggesting poor treatment response. These findings raise the possibility that CTEPH patients with a heterozygous RNF213 variant may have a distinct pathophysiology compared to typical CTEPH. We discovered novel somatic and germline mechanisms underlying disease development and contributing to poor treatment response in CTEPH. These factors may represent potential treatment targets for refractory cases.

## A025 Pulmonary Arterial Hypertension Alters Nephron Composition, Reprograms Metabolism, and Promotes Autoimmune Mechanisms in the Kidney

Miss Madelyn Blake^1^, Dr. Lynn Hartweck^2^, Jenna Mendelson^2^, Dr. Steeve Provencher^3^, Sandra Bonnet^3^, Dr. Sebastien Bonnet^3,4^, Dr. Kurt Prins^1^

^1^Gazes Cardiac Research Institute, Division of Cardiology, Department of Medicine, Medical University of South Carolina, Charleston, United States, ^2^Cardiovascular Division, Lillehei Heart Institute, University of Minnesota, Minneapolis, United States, ^3^Pulmonary Hypertension and Vascular Biology Research Groupe, CRIUCPQ, Laval University, Quebec City, Canada, ^4^Institute for Lung Health (ILH), German Center for Lung Research (DZL), Cardio‐Pulmonary Institute (CPI), Justus‐Liebig University Giessen, Giessen, Germany

Impaired kidney function in patients with pulmonary arterial hypertension (PAH) is associated with poor prognosis. However, current PAH therapies offer minimal benefit to renal function, and the underlying mechanisms of PAH‐related kidney injury remain poorly understood. To address this critical gap, we profiled the cellular and molecular landscape of human PAH kidneys using single nucleus RNA sequencing (snRNA‐seq). Nuclei were isolated from autopsy‐derived kidney tissue from five healthy controls and four PAH patients. snRNA‐seq was performed using the 10x Genomics platform and analyzed with Seurat v5. Compared to healthy controls, PAH kidneys exhibited a markedly altered cellular composition. Within the nephron, there was an increased relative abundance of thick ascending limb cells (control: 14.2%, PAH: 43.3%) and proximal tubule cells (control: 7.0%, PAH: 14.3%), alongside a reduction in principal cells (control: 38.2%, PAH: 17.1%). Outside of nephron segments, PAH kidneys showed elevated lymphocyte levels (control: 0.8%, PAH: 2.4%) and reduced endothelial cell populations (control: 12.7%, PAH: 8.2%). Molecularly, thick ascending limb and proximal tubule cells in PAH kidneys displayed a hypermetabolic phenotype, with upregulation of fatty acid metabolism, amino acid metabolism, and oxidative phosphorylation pathways. In contrast, principal and endothelial cells were enriched for oxidative stress and ferroptosis pathways. Additionally, PAH kidneys exhibited an autoimmune‐like profile, characterized by lymphocytic upregulation of T‐cell receptor signaling and NK cell activation, along with broad downregulation of MHC Class I signaling across multiple cell types. PAH nephropathy is marked by profound shifts in cellular composition, metabolic reprogramming, endothelial cell loss, and activation of autoimmune‐like pathways. It remains to be confirmed whether these findings are also observed in earlier disease stages. Nevertheless, these findings provide new insights into the pathophysiology of PAH‐associated kidney dysfunction and highlight potential therapeutic targets for this currently unaddressed complication of PAH.

## A026 Fontan‐Kreutzer Univentricular Circulation: Pulmonary Microvascular Reserve Quantification Through Selective Catheter‐Based Pulmonary Artery Digital Subtraction Angiography

MD, PhD, PVRI Ernesto Juaneda^1^, MD Alejandro Peirone^1^, MD Ignacio Juaneda^1^, MD, PhD Cristian Kreutzer^2^

^1^Private University Hospital of Córdoba, Córdoba, Argentina, ^2^Austral University Hospital, Pilar, Argentina

The Fontan‐Kreutzer (F‐K) procedure produces a circulation with non‐pulsatile flow, elevated systemic venous pressure, and low cardiac output. Data describing microvascular disease in living patients remain limited. To develop a method for quantifying pulmonary microvascular reserve.

Materials and Methods: A prospective study (January 2024–August 2025) included four fenestrated F‐K patients under routine follow‐up. Two had left and two right ventricular morphology; three had right‐sided and one a left‐sided extracardiac conduit. All had competent atrioventricular valves and preserved ejection fraction. Mean age was 22.5 years (15–32). Evaluations included oxygen saturation, lower limb venous Doppler echography, D‐dimer, and cardiac catheterization with mean pulmonary artery pressure (mPAP), wedge pressure (WP), transpulmonary gradient (TPG), pulmonary vascular resistance index (PVRI), and selective catheter‐based pulmonary artery digital subtraction angiography (DSA). DSA analysis determined capillary phase, dividing each lung base into nine areas. Contrast density was categorized as black, grey, or white, scored 2, 1, and 0 respectively. A score of 18 indicated preserved microvascular reserve; < 18 indicated decreased reserve. The score index was total score/18, with values of 1 considered preserved and < 1 decreased. Oxygen saturation averaged 89.5% (89–92). Doppler echography was normal in all. Mean D‐dimer was 382 ng/ml (140–650), > 500 ng/ml in 2/4 patients. Hemodynamics: mPAP 16.75 mmHg (5–19), WP 10.25 mmHg (9–12), TPG 6.5 mmHg (4–9), PVRI 4.65 U/m² (2.79–6.79). Mean right lung score was 10 (7–12), left lung 11 (5–14). Score index: right 0.55 (0.38–0.66), left 0.59 (0.24–0.77). Selective catheter‐based pulmonary artery DSA quantified pulmonary microvascular reserve by analyzing nine lung areas. All patients showed decreased reserve. Findings suggest microvascular occlusion due to distal, silent, multifactorial thrombosis. Therapy was switched from aspirin to apixaban.

## A027 Genetic Variants of Group 1 Paediatric Onset Pulmonary Arterial Hypertension

MD, PhD, PVRI Ernesto Juaneda^1,3^, Biotechnoloy Graduate Sofia Savy^2^, MD Victoria Damonte^1^, Biology, PhD Laura Larovere^1^, Biology, PhD Carola Grosso^1^, Genetic Graduate, PhD Silene Silvera^1^, MD Florencia Pabletich^3^, MD Juan Diaz^3^, MD Ignacio Juaneda^1,3^, MD Alejandro Peirone^1,3^, Chemical Bioquimic Graduate, PhD Juan Pablo Nicola^2^

^1^Children´s Hospital, Córdoba, Argentina, ^2^Chemist Science Faculty, National University of Córdoba, Córdoba, Argentina, ^3^Private University Hospital of Córdoba, Córdoba, Argentina

Pulmonary arterial hypertension (PAH) is a progressive vascular disease with increased right ventricular afterload that conducts to heart faillure. Pulmonary arterioles endotelial and muscular cells molecular signals may be altered when pathogenic genetic variants are present.

Objective: genetic variants description of group 1 paediatric‐onset PAH in Argentine patients. Material and Methods: Between February 2019‐March 2024, 16 children were diagnosed with paediatric PAH, group 1. Screening included functional class (FC), 2D‐Doppler echocardiography, blood tests, and elective cardiac catheterization. After informed consent, blood samples were collected (biobank 78.5%), and a PAH‐targeted exome panel was performed. Data were analyzed retrospectively. Paediatric‐onset diagnosis mean age 4.87 ± 5.27 years, 8 female, 8 males, FC: II: 3, IIIa:4 and IIIb:9 and phenotype: 9 idiopathic, 3 persistent fetal circulation of newborn (PFCNB), 3 associated with congenital heart disease (CHD) and 1 portopulmonary hypertension, 2 associated to trisomy 21, 1 trisomy 18 and 1 cleft palate. Pathogenic genetic variants were found in 5/16 (31.25%): KDR, FOXF1, GGCX and 2 patients NFU1. Cardiac catheterization mean pulmonary artery pressure 53.56 ± 16.88 mmHg, pulmonary vascular resistance index 14.53 ± 8.81 mmHg/m2 and wedge pressure < 15 mmHg in all of them. Specific PAH treatment was stablished according risk determinants in all patients through current therapeutic targets. Follow‐up 6.42 ± 5.22 years; fatal outcome in 6/16 (37.5%): 3 with genetic variants (KDR, FOXF1, compound heterozygous NFU1), 1 idiopathic, 1 CHD and 1 portopulmonary hypertension; survival 10/16 (62.5%): 1 GGCX, 1 heterozygous NFU1, 6 idiopathic, 2 PFCNB. Genetic variants were found in 31.25% of this paediatric‐onset PAH cohort. Fatal outcomes occurred with KDR, FOXF1, and compound heterozygous NFU1, while survival was seen with GGCX and heterozygous NFU1. The presence of pathogenic genetic variants may contribute to PAH by disrupting signaling in pulmonary arteriolar endothelial and smooth muscle cells, consistent with their previously described pathophysiology.

## A028 A Randomized, Double‐Blind, Placebo‐Controlled Study of Treprostinil Palmitil Inhalation Powder (TPIP) in Patients With Pulmonary Arterial Hypertension (PAH)

Ekkehard Grünig^1^, Michael J. Cuttica^2^, Hiromi Matsubara^3^, Gerald O'Brien^4^, Bipin Mistry^4^, Zhaoyu Yin^4^, Kevin Mange^4^, Amy Boutet^4^

^1^Thoraxclinic at University Hospital Heidelberg, Heidelberg, Germany, ^2^Feinberg School of Medicine, Northwestern University, Chicago, USA, ^3^National Hospital Organization Okayama Medical Center, Okayama, Japan, ^4^Insmed Incorporated, Bridgewater, USA

TPIP is a dry powder investigational formulation of treprostinil palmitil, a treprostinil prodrug, enabling prolonged lung exposure and long‐acting vasodilation with once‐daily dosing. This phase 2 study (NCT05147805) assessed the efficacy, safety, and tolerability of TPIP versus placebo in patients with World Health Organization (WHO) Group 1 PAH over 16 weeks. Patients were randomized 2:1 to TPIP (80‐640 μg once daily) or placebo, stratified by WHO functional class (II vs III) and number of baseline PAH medications. The primary endpoint was the change in pulmonary vascular resistance (PVR) at 16 weeks, approximately 24 hours post‐dose. Secondary endpoints included safety and change in 6‐minute walk distance (6MWD) and N‐terminal pro‐B‐type natriuretic peptide (NT‐proBNP) levels, approximately 24 hours post‐dose. Patient characteristics were similar across TPIP (n = 69; 75.4% reached the maximum dose of 640 μg) and placebo (n = 33) groups. At week 16, TPIP showed a 35% placebo‐adjusted reduction from baseline in the primary efficacy endpoint PVR (P < 0.001); a 35.5‐m placebo‐adjusted improvement in 6MWD (P = 0.003) and 60% placebo‐adjusted reduction in NT‐proBNP levels (P < 0.001) were also observed. TPIP was generally well tolerated with no new safety concerns; all cough events were mild to moderate. TPIP added to background therapy resulted in a statistically significant improvement in the primary endpoint (PVR) and showed numerical improvement in 6MWD and other prespecified efficacy measures approximately 24 hours after the last dose. Safety outcomes were consistent with those expected for the inhaled prostanoid class.

## A029 Novel Insights From Functional Respiratory Imaging (FRI) Analysis in a Phase 2 Randomized, Double‐Blind, Placebo‐Controlled Study of Treprostinil Palmitil Inhalation Powder (TPIP) in Patients With Pulmonary Arterial Hypertension (PAH)

Ioana Preston^1^, Robert P. Frantz^2^, Patrick Muchmore^3^, Ben Lavon^3^, Jan De Backer^3^, Louis Holdstock^4^, Jia Li^4^, Zhaoyu Yin^4^, Gerald O'Brien^4^

^1^Lahey Hospital and Medical Center, Burlington, USA, ^2^Mayo Clinic, Rochester, USA, ^3^FLUIDDA, Inc., New York, USA, ^4^Insmed Incorporated, Bridgewater, USA

TPIP is a dry powder investigational formulation of treprostinil palmitil (a treprostinil prodrug), aiming to enable prolonged lung exposure and long‐acting vasodilation with once‐daily dosing. Results from the phase 2 study (NCT05147805) showed that TPIP met the primary endpoint (pulmonary vascular resistance) and was generally well tolerated in patients with PAH. To evaluate effects of TPIP on pulmonary blood vessel volume with FRI, a novel technique using 3‐dimensional reconstructions of high‐resolution computed tomography images. In this exploratory analysis, FRI parameters were calculated at screening and week 16 in the full computed tomography (CT) analysis set (all patients who had scans). Macroscopic pulmonary vessel volumes ≤ 5 mm² (BV5) and ≥ 10 mm² (BV10) in cross‐sectional areas were analyzed and presented as a proportion of total vessel volume (‐PR) and as a ratio (BV510RATIO). P values < 0.05 were considered nominally significant and not adjusted for multiplicity. Thirty‐four patients had baseline and week 16 FRI measurements. A nominally significant treatment effect of TPIP versus placebo was observed on change from baseline in BV5 at week 16 of 21.44 mL (19.36% of baseline mean in the placebo group; P = 0.02), with a trend towards a treatment effect on BV510RATIO of 0.98 (22.09% of baseline mean in the placebo group; P = 0.05). The analysis also appeared to show a trend towards treatment effect on BV10 of −9.35 mL (−30.68% of baseline mean in the placebo group; P = 0.13). Changes in BV5PR and BV10PR appeared to be directionally consistent, with trends toward treatment effect of 5.40% and −4.45%, respectively (P = 0.10 for both). Treatment with TPIP in patients with PAH can result in changes across all FRI parameters directionally consistent with peripheral vasodilation (increased BV5) and can reduce dilatation of proximal vessels (reduced BV10). Further evaluation of FRI in a larger trial may provide additional insights.

## A030 Study Design of a Phase 3 Trial of Treprostinil Palmitil Inhalation Powder (TPIP) in Patients With Pulmonary Arterial Hypertension (PAH)

Ekkehard Grünig^1^, Raymond Benza^**2**^, Murali Chakinala^3^, Jean Elwing^4^, Robert P. Frantz^5^, Ioana Preston^6^, Louis Holdstock^7^, Jia Li^7^, Kevin Mange^7^, Gene Sullivan^7^, Zhaoyu Yin^7^, Gerald O'Brien^**7**^

^1^Thoraxklinik Heidelberg gGmbH at University Hospital Heidelberg, Heidelberg, Germany, ^2^Sentara Health, Norfolk, USA, ^3^Washington University, St. Louis, USA, ^4^University of Cincinnati, Cincinnati, USA, ^5^Mayo Clinic, Rochester, USA, ^6^Lahey Hospital and Medical Center, Burlington, USA, ^7^Insmed Incorporated, Bridgewater, USA

PAH remains a progressive and life‐threatening condition despite available therapies. Currently approved prostacyclin pathway therapies for PAH are effective but limited by complex delivery systems, short half‐lives, and adverse event profiles that restrict dosing, impact adherence, and limit maximum therapeutic benefit. TPIP demonstrated efficacy and was generally well‐tolerated, without new safety concerns, in a phase 2b PAH trial (NCT05147805). This phase 3, randomized, double‐blind, placebo‐controlled, multicenter trial will evaluate safety and efficacy of once‐daily (QD) TPIP in patients with PAH over 24 weeks. Approximately 300 patients will be randomized 1:1 to receive TPIP or matching placebo QD via oral inhalation. Randomization will be stratified by geographic region (Europe, Japan, North America, rest of world), World Health Organization (WHO) functional class (II, III, IV), sotatercept use (Yes/No), and baseline 6‐minute walk distance (6MWD; ≤ 350 m and > 350 m). Key inclusion criteria include diagnosis of WHO Group 1 pulmonary hypertension; New York Heart Association (NYHA)/WHO functional class II‐IV; PAH confirmed by right heart catheterization at or < 90 days before screening (mean pulmonary arterial pressure > 20 mmHg at rest, pulmonary capillary wedge pressure ≤ 15 mmHg, and pulmonary vascular resistance ≥ 5 WU); and 6MWD ≥ 150 m and ≤ 450 m on 2 tests performed ≥ 4 hours apart at screening. Treatment will be initiated at 80 μg QD, titrated over 4 weeks to the patient's highest tolerated dose. Dose increases are allowed through week 16. Primary endpoint is change from baseline in 6MWD. Additional endpoints include proportion of patients with improvement in WHO functional class, hemodynamics, change from baseline in N‐terminal pro‐B‐type natriuretic peptide concentration, time to first clinical worsening event, and safety and tolerability. This phase 3 trial aims to further evaluate efficacy, safety, and tolerability, of TPIP, administered QD, in a broader group of patients with PAH already receiving background therapies.

## A031 Comparison of CT‐Based Pulmonary Vascular Biomarkers in Predicting Right Ventricular Dysfunction and All‐Cause Mortality in the Framingham Heart Study

Dr. Andrew Synn^1^, Dr. Farbod Rahaghi^2^, Dr. Connie Tsao^1^, Dr. George Washko^3^, Dr. Raul San Jose Estepar^3^, Dr. George O'Connor^4^, Dr. Murray Mittleman^1^, Dr. Mary Rice^1^

^1^Beth Israel Deaconess Medical Center, Boston, USA, ^2^UCLA, Los Angeles, USA, ^3^Brigham and Women's Hospital, Boston, USA, ^4^Boston University Medical Center, Boston, USA

Non‐invasive image‐based biomarkers have the potential to improve diagnosis, prognostication, and phenotyping of pulmonary vascular disease. However, multiple different volumetric metrics have been proposed, and little is known about their relative performance in predicting outcomes. We sought to compare the associations of three promising vascular biomarkers with all‐cause mortality and right ventricular function in a general population cohort. The sample consisted of 2,470 participants of the Framingham Heart Study who underwent chest CT (2008‐2011) with volumetric quantification of the pulmonary vasculature using automated image analysis. From these, we calculated the total blood vessel volume (TBV), small vessel (cross‐sectional area< 5mm2) volume (BV5), and pre‐acinar vessel (area 5‐20mm2) volume (BV5‐20). The three vascular biomarkers of interest were: BV5/TBV (pruning), BV5‐20/TBV (pre‐acinar dilation), and TBV. We constructed multivariable Cox proportional hazards models (adjusted for age, sex, smoking status/pack‐years, height, weight, and study cohort) to investigate the association of each metric with all‐cause mortality over 10 years of follow‐up. In a subset of 881 participants who also underwent cardiac MRI, we used multivariable linear regression models to examine cross‐sectional associations of each biomarker with right ventricular ejection fraction (RVEF). In adjusted models, we found that only BV5/TBV was associated with mortality: each standard deviation of more severe pruning (lower BV5/TBV) was associated with a 1.5‐fold higher rate of death (95% CI: 1.21‐1.86, p < 0.001). Similarly, only BV5/TBV was associated with RVEF on MRI. RVEF was 1.0% lower (95% CI: 0.5‐1.6, p < 0.001) per standard deviation of more severe pruning. In contrast, neither BV5‐20/TBV nor TBV were significantly associated with mortality or RVEF. In this study comparing three common image‐based volumetric biomarkers of pulmonary vasculopathy, only pruning of the small vessels on CT (BV5/TBV) was associated with greater risk of death and reduced right ventricular function in a general population cohort.

## A032 Prognostic Value of Right Atrial Pressure to Cardiac Output Slope (RAP/CO Slope) in Patients Undergoing Exercise Right Heart Catheterization for Suspected or Manifest Pulmonary Hypertension

**A sub analysis of the multicenter PEX‐NET Collaboration.**

Dr. Sc. Med. Julian Müller^1,2^, PD Dr. med. Philipp Douschan^3^, Dr. med. Julian Mayer^1,2^, PD Dr. med. Mona Lichtblau^1,2^, Prof. Mads J. Anderson^4^, Prof. Joan Albert Barberà^5^, Prof. Isabel Blanco^5,6^, Prof. Sergio Caravita^7,8^, Prof. Robin Condliffe^9^, Prof. Michele D'Alto^10^, Dr. med. Benjamin Egenlauf^11^, Prof. Ralf Ewert^12^, Prof. Nazzareno Galiè^13^, Prof. Ekkehard Grünig^11^, Dr. med. Alexander Heine^12^, Dr. med. Simon Herkenrath^14^, Prof. Philippe Herve^15^, Dr. med. Brian A. Houston^16^, Prof Steven Hsu^17^, Prof. Marc Humbert^15^, Dr. med. Krzysztof Kasperowicz^18^, Prof. Marcin Kurzyna^18^, Dr. med. Gregory D. Lewis^19^, Prof. Susanna Mak^20^, Prof. Rajeev Malhotra^21^, Prof. Bradley A. Maron^22,23^, Dr. med. Colm McCabe^24,25^, Prof. Rudolf K. F. Oliveira^26^, Prof. Horst Olschewski^27,28^, Prof. Stephan Rosenkranz^14^, Prof. Laurent Savale^15^, Prof. Rogerio Souza^29^, Prof. David Systrom^30^, Prof. Ryan J. Tedford^16^, Prof. Khodr Tello^31,32^, Prof. Anton Vonk‐Noordegraaf^33^, Prof. Gabor Kovacs^3^, Prof. Silvia Ulrich^1,2^

^1^Department of Pulmonology, University Hospital Zurich, Zurich, Switzerland, ^2^Faculty of Medicine, University of Zurich, Zurich, Switzerland, ^3^Division of Respiratory Medicine, Lung Research Cluster, Medical University Graz, Graz, Austria, ^4^Department of Cardiology, Aarhus University Hospital, Aarhus, Denmark, ^5^Department of Pulmonary Medicine, Hospital Clinic‐IDIBAPS, University of Barcelona, Biomedical Research Networking Center on Respiratory Diseases (CIBERES), Barcelona, Spain, ^6^ERN‐LUNG, Hospital Clínic, Barcelona, Spain, ^7^Department of Cardiology, Ospedale San Luca, IRCCS Istituto Auxologico Italiano, Milan, Italy, ^8^Department of Management, Information and Production Engineering, University of Bergamo, Dalmine, Italy, ^9^University of Sheffield, Sheffield, UK, ^10^Department of Cardiology ‐ Monaldi Hospital, Naples, Italy, ^11^Centre for Pulmonary Hypertension, Thoraxklinik Heidelberg gGmbH at Heidelberg University Hospital, Translational Lung Research Center Heidelberg, German Center for Lung Research, Heidelberg, Germany, ^12^Ernst Moritz Arndt University Greifswald, Greifswald, Germany, ^13^University of Bologna, Bologna, Italy, ^14^University of Cologne, Cologne, Germany, ^15^Université Paris‐Saclay, Inserm UMR_S 999 (HPPIT), Service de Pneumologie et Soins Intensifs Respiratoires, ERN‐LUNG, Hôpital Bicêtre, Le Kremlin‐Bicêtre, France, ^16^Medical University of South Carolina, Charlestown, USA, ^17^Johns Hopkins University School of Medicine, Baltimore, USA, ^18^Department of Pulmonary Circulation, Thromboembolic Diseases and Cardiology, Centre of Postgraduate Medical Education, European Health Centre Otwock, Otwock, Poland, ^19^Massachusetts General Hospital, Harvard Medical School, Boston, USA, ^20^Division of Cardiology, Department of Medicine, Sinai Health, University of Toronto, Toronto, Canada, ^21^Heart and Vascular Institute, Massachusetts General Hospital, Harvard Medical School, Boston, USA, ^22^Division of Cardiovascular Medicine, University of Maryland School of Medicine, Baltimore, USA, ^23^University of Maryland Institute for Health Computing, Bethesda, USA, ^24^Royal Brompton Hospital, London, UK, ^25^National Heart and Lung Institute, Imperial College London, London, UK, ^26^Federal University of São Paulo, UNIFESP, São Paulo, Brazil, ^27^Faculty of Medicine, Sigmund Freud Private University, Vienna, Austria, ^28^Dep. Pneumology, Intensive Care Medicine and Sleep Medicine, Charité University Medicine, Berlin, Germany, ^29^Instituto do Coração – Hospital das Clínicas da Faculdade de Medicina da Universidade de São Paulo, São Paulo, Brazil, ^30^Brigham and Women's Hospital, Harvard Medical School, Boston, USA, ^31^Justus Liebig University, Deutsches Zentrum für Lungenforschung, Giessen, Germany, ^32^Institute for Lung Health, Giessen, Germany, ^33^Amsterdam UMC location Vrije Universiteit Amsterdam, Department of Pulmonary Medicine, Amsterdam, The Netherlands

This study investigates the prognostic value of the right atrium pressure to cardiac output relationship between rest and end‐exercise (RAP/CO slope) in patients undergoing right heart catheterization for suspected or confirmed pulmonary hypertension (PH). This analysis was conducted on the retrospective arm of the Pulmonary Hemodynamics during Exercise Network Clinical Research Collaboration (PEX‐NET), a multicenter database involving 23 PH centers. All patients with data at rest and during exercise in the same body position and available RAP/CO slopes were included. Cox proportional hazard models adjusted for age, sex and hemoglobin were analyzed.

Results: 856 patients (median (Q1, Q3), age 59 (46, 69), 67% female) were included, 336 with resting PH. The median observation time was 6.5 (3.7, 10.8) years, 114 (13%) patients died. Overall, higher RAP/CO slopes were associated with increased risk of death (hazard ratio 1.08, 95%CI: 1.03‐1.13, p < 0.001). In patients with resting PH, those with a RAP/CO slope ≥ 1.0 mmHg·min·L⁻¹ had a 2.2‐fold higher risk of death (hazard ratio: 2.20; 95% CI: 1.07‐4.51; p = 0.032) compared to RAP/CO slope < 1.0 mmHg·min·L⁻¹. In the present study, the RAP/CO slope, reflecting downstream venous congestion, right ventricular (RV) diastolic properties and RV contractile reserve during exercise, emerged as a significant prognostic marker across a continuum of patients with varying stages of RV‐afterload strain. A RAP/CO slope ≥ 1 mmHg·min·L⁻¹ was identified as particularly predictive in manifest PH. The additive prognostic value of the RAP/CO slope is explained as it incorporates RV‐diastolic dysfunction and thus may serve to identify PH‐patients with increased risk.

## A033 Dynamin 2 Plays a Fundamental Role in Mitochondrial Fission and Its Dysregulation by the miR‐124a‐3p‐STAT3‐DNM2 Pathway Contributes to Pulmonary Arterial Hypertension

Dr. Asish Das Gupta^1^, Dr. Kuang‐Hueih Chen^1^, Dr. Danchen Wu^1^, Dr. V. Siddartha Yerramilli^1^, Dr. Patricia Lima^2^, Ms. Ashley Martin^1^, Dr. Rachel Bentley^1^, Mr. Benjamin Ott^2^, Mr. Jeffrey Mewburn^1^, Dr. Lian Tian^3^, Dr. Ruaa Al‐Qazazi^1^, Mr. Isaac Emon^1^, Mr. Pierce Colpman^1^, Ms. Lindsay Jefferson^1^, Mr. Curtis Noordhof^2^, Mr. Oliver Jones^2^, Dr. Charles Hindmarch^2^, Dr. Stephen Archer^1^

^1^Department of Medicine, Queen's University, Kingston, Canada, ^2^Translational Institute of Medicine (TIME), and the Queen's Cardiopulmonary Unit (QCPU), Department of Medicine, Queen's University, Kingston, Canada, ^3^Strathclyde Institute of Pharmacy and Biomedical Sciences, University of Strathclyde, Glasgow, United Kingdom

Mitochondrial fission, mediated by dynamin‐related protein 1 (Drp1) is increased in PAH; however, Drp1 and its binding partners appear insufficient to complete fission. The large GTPase, dynamin 2 (DNM2), interacts with Drp1 at the mitochondrial fission sites to complete mitochondrial fission. We assessed whether DNM2 interacts with Drp1 to mediate mitotic fission and accelerate cell cycle progression, permitting the hyperproliferative phenotype of pulmonary arterial smooth muscle cells (PASMC) in patients and rodents with pulmonary arterial hypertension (PAH). We also assessed DNM2's value as a therapeutic target. Mitochondrial morphology and protein colocalization were assessed using super‐resolution STED microscopy, protein interactions by immunoprecipitation and transcriptomic changes by RNA‐seq. DNM2 was quantified in PASMC and lungs from PAH patients and monocrotaline (MCT)‐ and SU5416/hypoxia (Su/Hx)‐induced PAH rats. siDNM2's effects on cell proliferation, cell cycle progression and apoptosis were assessed by flow cytometry. siDNM2 was nebulized to MCT‐ and Su/Hx‐PAH rats and disease regression was quantified by cardiac catheterization. DNM2 is increased in PAH PASMC in humans and rodents. DNM2 interacts with Drp1 via its GTPase domain, permitting mitochondrial translocation. siDNM2 inhibits fission. siDNM2 also downregulates the Regulator of Cell Cycle (RGCC) thereby causing cell cycle arrest and apoptosis. Conversely, augmenting DNM2 in normal PASMC induces fission and accelerates proliferation. In PAH PASMC, decreased miR‐124‐3p upregulates DNM2, and restoring miR‐124‐3p decreases DNM2, inhibits proliferation and induces apoptosis. Second, STAT3, which is also negatively regulated by miR‐124‐3p, regulates DNM2. siSTAT3 reduces DNM2 and mitochondrial fission in PAH. Nebulized siDNM2 regresses experimental PAH. DNM2's GTPase domain binds Drp1, mediating mitochondrial translocation to create tighter mitochondrial constriction. DNM2's upregulation in PAH reflects both miR‐124‐3p deficiency and STAT3 activation. A miR‐124‐3p‐STAT3‐DNM2‐Drp1‐RGCC pathway accelerates mitotic fission and links mitochondrial and nuclear division in PAH. DNM2 is a novel antiproliferative therapeutic target in PAH.

## A034 The Role of Endothelial Prdm16 in a Mouse Model of Experimental Pulmonary Arterial Hypertension

Miss Alexia Mahy^1^, Ellen Caluwé^2^, Mirthe Willekens^1^, Petra Vandervoort^1^, Séverine Remy^3^, Laurent Tesson^3^, Marleen Lox^1^, Hannelore Kemps^1^, Frédéric Perros^4^, Rozenn Quarck^5^, Aernout Luttun^1^

^1^Center for Molecular and Vascular Biology, Department of Cardiovascular Sciences, KU Leuven, Leuven, Belgium, ^2^Cardiology, Department of Cardiovascular Sciences, KU Leuven, Leuven, Belgium, ^3^Centre de Recherche en Transplantation et Immunologie UMR1064, INSERM, Université de Nantes, Nantes, France, ^4^Laboratoire CarMeN, UMR INSERM U1060/INRA U1397, Université Claude Bernard Lyon 1, Pierre‐Bénite, France, ^5^Laboratory of Respiratory Diseases and Thoracic Surgery, Department of Chronic Diseases and Metabolism, KU Leuven, Leuven, Belgium

Pulmonary arterial hypertension (PAH) is a rare but severe cardiopulmonary disease characterized by pulmonary vascular remodeling resulting in increased pulmonary vascular resistance, right heart failure, and death, if left untreated. Current treatments only provide palliative relief, underscoring the need to explore novel targets to cure PAH. We demonstrated that endothelial but not smooth muscle cell PRDM16, an arterial‐restricted transcription factor, is indispensable for arterial flow recovery in the systemic circulation by preserving arterial endothelial function upon peripheral artery disease in mice. Considering the crucial role of the endothelium in PAH, we hypothesized that endothelial PRDM16 deficiency would exacerbate PAH. After establishment of an efficient regime for specific Prdm16 deletion in endothelial cells, our goal was to implement and validate the Sugen/hypoxia (SuHx) mouse model. Hereto, wild‐type mice were exposed to Su/Hx, Su alone, Hx alone, or a vehicle for three weeks, and right ventricular systolic pressure (RVSP) and hypertrophy (RVH) were evaluated. Unexpectedly, RVSP and RVH were similar in mice exposed to SuHx and hypoxia alone, suggesting that in our hands mice exposed to SuHx did not develop more severe pulmonary hypertension (PH) compared to mice exposed to hypoxia alone. Therefore, to study the role of endothelial PRDM16 in PH, we subjected EC‐deficient PRDM16 mice and wild‐type littermates to hypoxia for 1 or 3 weeks. Loss of endothelial PRDM16 did not alter RVSP, RVH or pulmonary vascular remodeling. These findings suggest that, in contrast to the systemic circulation, PRDM16 may play a role in a non‐endothelial compartment during P(A)H, e.g. in arterial smooth muscle cells or epithelial cells where it is also prominently expressed. To further investigate the involvement of PRDM16 in PAH, we will initially resort to mice and rats with an ubiquitous heterozygous deficiency for PRDM16, the latter of which we have successfully generated by CRISPR/Cas9 technology.

## A035 Evaluation of Disease Remission Definitions in Pulmonary Arterial Hypertension Clinical Trials

Dr. Jason Weatherald^1^, Dr. Yuri Matusov^2^, Dr. Sandeep Sahay^3^, Dr. Jidapa Kraisangka^4^, Dr. Thomas Cascino^5^, Dr. Charles Fauvel^6^, Dr. Shili Lin^7^, Priscilla Correa‐Jacque^8^, Dr. Raymond Benza^8^

^1^University Of Alberta, Edmonton, Canada, ^2^Cedars‐Sinai Medical Center, Los Angeles, USA, ^3^Houston Methodist Hospital, Houston, USA, ^4^Mahidol University, Salaya, Thailand, ^5^University of Michigan, Ann Arbor, USA, ^6^Rouen University Hospital, Rouen, France, ^7^Ohio State University, Columbus, USA, ^8^Eastern Virginia Medical School, Old Dominion University, Norfolk, USA

The concept of disease remission in pulmonary arterial hypertension (PAH) has emerged with new therapies targeting vascular remodeling. Proposed definitions of remission are based on normalization/near‐normalization of hemodynamics, symptoms, risk, and right heart function. These remission definitions have not yet been validated so their utility as endpoints in trials or in clinical practice are uncertain. We used a harmonized dataset of individual patient data from 7 PAH clinical trials submitted to the FDA and applied modified versions of the complete (CR) and partial remission (PR) definitions (Rahaghi et al. Lancet Respir Med 2025) based on available variables. CR was defined as achieving mPAP < 20 mmHg and PVR < 2 WU and WHO FC 1 and PR was defined as achieving mPAP ≤ 35 mmHg with PVR < 5 Wood units, WHO FC 1 or 2, and NT‐proBNP < 300 ng/L at follow‐up. The primary outcome was clinical worsening (CW), as defined in each parent trial, at 1 year. Of 3927 trial participants, 1796 had follow‐up hemodynamics, WHO FC, and NT‐proBNP available. Median time to follow‐up assessment of CR and PR was 112 days. No patients achieved CR and only 59 achieved PR (3.3%) at follow‐up. For patients achieving PR (vs no PR), the absolute risk difference in 1‐year CW was ‐14.1% (95%CI ‐25.0 to ‐4.3%) and relative risk was 0.39 (95%CI 0.15‐0.99). The odds ratio for 1‐year CW was 0.35 (95%CI 0.12‐0.98, p = 0.045) adjusted for age, sex, baseline PVR and baseline WHO FC. No patients in contemporary PAH trials achieved CR whereas a low proportion achieved PR. Achieving PR was associated with lower CW risk at 1‐year, lending some validity to proposed definitions. Further validation of remission constructs in real‐world populations and prospective trials of novel therapies are warranted.

## A036 Platelet Activation and Macrophage Recruitment Drive Vascular Dysfunction in a Murine Model of Sickle Cell Pulmonary Hypertension

Dr Daniel Colon Hidalgo^1^, PhD Christina Lisk^1^, Nathan Dee^1^, PhD Melissa Lucero^1^, Delaney Swindle^1^, Janelle Posey^1^, MD Cassidy Delaney^1^, PhD David Irwin^1^, MD Eva Nozik^1^

^1^University Of Colorado, Aurora, USA

Pulmonary hypertension (PH) is a severe and life‐limiting complication of sickle cell disease (SCD). Chronic hemolysis and hypoxia promote release of cell‐free hemoglobin and reactive oxygen species (ROS), generating an oxidizing extracellular milieu that contributes to vascular injury. Platelet activation and immune cell recruitment are increasingly recognized as drivers of SCD‐associated PH (SCD‐PH), but the mechanisms linking extracellular redox imbalance to platelet dysfunction and inflammation remain poorly defined. Transgenic SCD (Berkeley, Berk‐SS) and wild‐type (WT) mice were maintained under normoxic or chronic hypoxic conditions. Platelet activation was assessed by flow cytometry for P‐selectin and activated integrin αIIbβ3. Platelet–leukocyte aggregates (PLAs) and macrophage phenotypes were evaluated by flow cytometry and lung histopathology. Hemodynamics were determined by right heart catheterization to obtain right ventricular systolic pressure (RVSP), ejection fraction, and pulmonary vascular remodeling indices. Compared to WT controls, Berk‐SS platelets exhibited markedly elevated P‐selectin, activated αIIbβ3, and increased PLAs. Lung sections from hypoxic Berk‐SS mice demonstrated prominent accumulation of CD169⁺ macrophages, absent in WT mice, suggesting redox‐driven macrophage recruitment. Proteomic analysis of lung macrophages identified hypoxia‐induced dysregulation of platelet‐related proteins, including platelet‐activating factor acetylhydrolase 2 (PAFAH2) and PECAM1/CD31, implicating altered platelet–macrophage signaling in vascular inflammation. Strikingly, depletion of macrophages with clodronate significantly improved RV contractility, PA–RV coupling, and ejection fraction relative to vehicle controls, supporting a pathogenic role of macrophage–platelet interactions in SCD‐PH.

These findings identify platelet activation and macrophage recruitment as key contributors to pulmonary vascular dysfunction in SCD‐PH. Ongoing studies aim to define the upstream triggers of these interactions and their potential as therapeutic targets to prevent vascular remodeling in SCD.

## A037 Revealing The Role of Interleukin 9 in The Pathogenesis of Pulmonary Hypertension As a New Immunomodulatory Therapy

Doctor Sandra Medrano Garcia^1,2,3^, Doctor Yajuan Zhang^4^, Prof. Dr. Magdalena Huber^4^, Prof. Dr. Dr. Werner Seeger^1,2,3^, Prof. Dr. Rajkumar Savai^1,2,3^, Prof. Dr. Soni Pullamsetti^1,2,3^

^1^Institute for Lung Health (ILH), Justus Liebig University, Giessen, Germany, ^2^Max Planck Institute for Heart and Lung Research, Member of the German Center for Lung Research (DZL), Member of the Cardio‐Pulmonary Institute (CPI), Bad Nauheim, Germany, ^3^Department of Internal Medicine, Justus‐Liebig University Giessen, Member of the DZL, Member of CPI, Germany, ^4^Institute for Medical Microbiology and Hygiene, Philipps‐University Marburg, Germany

T cell populations are divided into several subpopulations based on their cytokine profiles, including a newer subtype of Th cells linked to tumorigenesis: Th9 cells, which predominantly produce the cytokine IL‐9. Pulmonary hypertension (PH) is characterized by perivascular accumulation of T cells that likely contributes to vascular changes. In PH, CD4 + T cells (T‐helper, Th) accumulate and communicate with pulmonary artery cells and the surrounding environment via soluble cytokines and cell‐derived molecules. However, it is not yet known which specific T cell subpopulations are involved in the pathogenic mechanism. Additionally, the role of Th9 cells and the cytokines they release (e.g., IL‐9) in PH pathology has not been investigated. The impact of Th9 on PH was studied in vitro to assess functional changes in pulmonary arterial adventitial fibroblasts (PAAFs), pulmonary arterial smooth muscle cells (PASMCs), and pulmonary microvascular endothelial cells (PMVECs). Th9 cells were also mapped and validated in lung tissues and cells from patients with idiopathic pulmonary arterial hypertension (IPAH). Our data show that Th9 and IL‐9+ cells are increased in the perivascular compartment of lung tissue from IPAH patients, as well as in the perivascular compartment after 35 days in the MCT rat model and after 5 weeks of hypoxic exposure in mouse lungs. In addition, IL‐9 induces proliferation and migration of human donor and IPAH PASMCs and mouse control PASMCs, proliferation of IPAH PAAFs, and angiogenesis and proliferation of human and mouse PMVECs. In summary, our study demonstrates for the first time a role for IL‐9+ cells in PH pathology and a proliferative, migratory, and angiogenic effect on pulmonary vascular cells, although the underlying mechanisms require further investigation.

## A038 Pulmonary Hypertension in Interstitial Lung Diseases (PH‐ILD) in Greece: Epidemiology, Clinical Features, and Management from a Multicenter Retrospective Study

MD, MSc Athanasios Zacharias^1^, MD, MSc, PhD Afroditi Boutou^1^, Msc Sofia Zafeiri^2^, MSc Panagiotis Papapetrou^2^, MSc Antonis Georgiou^2^, MSc Eirini Kefalidou^2^, MD, PhD Anastasia Anthi^3^, MD, PhD Effrosyni Dima^3^, MD, PhD Frantzeska Frantzeskaki^4^, MD Thomas Chrysochoidis^1^, MD Panagiotis Gourgiotis^1^, MD, PhD Panagiotis Kariofillis^5^, MD, MMedSc, PhD Eftychia Demerouti^5^, MD, PhD Ioanna Mitrouska^6^, MD Vaia Stamatopoulou^6^, MD, PhD Eleni Stagaki^7^, MD, PhD George Giannakoulas^1^, MD, PhD Iraklis Tsagkaris^4^, MD, MSc, PhD Georgia Pitsiou^1^

^1^Aristotle University of Thessaloniki, Thessaloniki, Greece, ^2^Galenica S.A., Kifisia, Greece, ^3^Evangelismos Hospital, Athens, Greece, ^4^National and Kapodistrian University of Athens, Athens, Greece, ^5^Onassis Hospital, Athens, Greece, ^6^University Hospital of Heraklion, Heraklion, Greece, ^7^Sotiria Chest Hospital, Athens, Greece

To describe the epidemiology, comorbidities, lung function, treatment patterns, and clinical outcomes of patients with PH‐ILD in Greece. A retrospective study was conducted using anonymized data from all seven nationally recognized reference centers for PH‐ILD in Greece. Adult patients (≥ 18 years) with confirmed diagnosis of both pulmonary hypertension (ICD‐10: I27.0/I27.2) and interstitial lung disease (ICD‐10: J84 series) between January 2022 and December 2024 were included. Patients diagnosed solely based on transthoracic echocardiography were excluded. Demographic characteristics, comorbidities, lung function, treatments, and clinical outcomes were analyzed.

Sixty‐nine adult patients were identified. Mean age at diagnosis was 65.9 years (SD: 10.9), and 39% were female. The most common ILD subtypes were connective tissue disease‐associated ILD (CTD‐ILD, 43%) and idiopathic pulmonary fibrosis (IPF 33%). Frequent comorbidities included hypertension/coronary artery disease (42%), diabetes (19%), and gastroesophageal reflux disease (19%). Mean pulmonary vascular resistance (PVR) was 6.47 WU, with 54% ≥ 5 WU. Average 6‐minute walk distance (6MWD) at diagnosis was 275.3 meters (SD: 100.6), with 53% walking < 300 meters. Twelve patients (17%) had died by data collection, with higher mean PVR among deceased (8.66 WU) versus survivors (6.02 WU). Lung function was impaired: mean FEV1 1.76 L (69% predicted), FVC 2.23 L (70% predicted), total lung capacity 3.25 L (59% predicted), and DLco 33% predicted, reflecting restrictive physiology with markedly reduced gas transfer. Regarding management, 74% required long ‐term oxygen therapy and 49% had at least one hospitalization for PH‐ILD. Treatments varied, including PH‐targeted therapies (inhaled treprostinil, PDE5 inhibitors, endothelin receptor antagonists), antifibrotics, or supportive care. This multicenter study demonstrates that PH‐ILD is a complex, multifactorial disease with a severe and often aggressive clinical course. Profound functional and hemodynamic impairment, multiple comorbidities, frequent hospitalizations, and high oxygen therapy requirements highlight the urgent need for earlier recognition and optimized management.

## A039 Still More Than Just Breathless: An Update on the Real‐World Symptom Burden in Pulmonary Hypertension in the Modern Era

Dr Christopher Chew^2^, Mr Shaun Clayton^1^, Ms Mary Ferguson^1^, Dr Stephen John Wort^2^, Dr Iain Armstrong^1^

^1^Pulmonary Hypertension Association UK, Sheffield, UK, ^2^National Pulmonary Hypertension Service at the Royal Brompton Hospital, London, UK

Novel therapies for pulmonary hypertension (PH) have significantly improved survival in the modern era. However, their impact on symptom burden and morbidity has been less well‐characterised. This study aimed to provide an update on the real‐world symptom burden in a large UK‐based community patient cohort. We distributed an open invitation to a cross‐sectional, online survey through Pulmonary Hypertension Association UK's social media platforms. The survey collected data on demographics, PH type, and the prevalence and impact of 13 common PH symptoms experienced in the last 12 months. Participants were also asked to rank their top three most impactful symptoms.

232 patients responded and were predominantly female (83%) with a mean age of 59. Respondents represented the full spectrum of PH diagnoses, with IPAH (31%) and CTEPH (18%) the most common. 9% of patients did not know their PH subtype. A high burden of overlapping symptoms was evident, with 94% of respondents reporting three or more symptoms. While shortness of breath on exertion was the most impactful symptom, fatigue was a close second, reported by over 85% of patients and ranked as one of the top three most impactful symptoms by 59%. Symptoms varied the most with weather changes, and least with menstrual cycles. Exploratory analyses suggested that reported symptoms did not vary by PH category but differed based on age and gender. Despite receiving specialist care in the modern era, a real‐world UK cohort of patients with PH continues to experience a substantial and persistent symptom burden despite contemporary treatment. Debilitating fatigue was nearly as significant as breathlessness, consistent with earlier studies on symptom burden in PH. This highlights an ongoing major unmet clinical need for more routine assessment of fatigue and the development of interventions targeted at this pervasive, debilitating symptom.

## A040 Heavy Hearts and Labouring Lungs: Patient Experiences of Life With Pulmonary Hypertension and Chronic Lung Disease

Dr Iain Armstrong^1^, Mr Shaun Clayton^1^, Ms Mary Ferguson^1^, Dr Stephen John Wort^2^, Dr Christopher Chew^2^

^1^Pulmonary Hypertension Association UK, Sheffield, UK, ^2^National Pulmonary Hypertension Service at the Royal Brompton Hospital, London, UK

The National Pulmonary Hypertension Service (NPHS) in the UK was primarily designed to meet the needs of patients with prototypical pulmonary arterial hypertension (PAH). However, patients with pulmonary hypertension associated with chronic lung disease (PH‐CLD) make up an increasing proportion within the NPHS. Our current limited understanding of the lived experiences of patients with PH‐CLD compromises our ability to provide truly appropriate and holistic care. We present the initial findings of a mixed‐methods study, conducted by the Pulmonary Hypertension Association UK (PHA UK), which aims to address this critical knowledge gap. Patients with PH‐CLD were recruited through an open invitation through the PHA UK's social media platforms. Semi‐structured interviews exploring their lived experiences were conducted, recorded, and transcribed verbatim; a detailed thematic analysis is currently underway. Following interviews, participants completed a suite of PROMs (emPHasis‐10, Dyspnoea‐12, EQ‐5D‐5L, FROM‐16, Fatigue Severity Scale) and provided a self‐reported WHO Functional Class. Quantitative data from PROMs were analysed using descriptive statistics to characterise the cohort's health status and symptom burden. 19 patients have been included, with diagnoses of COPD (n = 6), connective‐tissue‐related interstitial lung disease (n = 7), pulmonary sarcoidosis (n = 5) and congenital lung disease (n = 1). Initial PROM data reveals a meaningful impact on quality of life and significant symptom burden. The cohort reported a median emPHasis‐10 score of 30 (out of 50) and a substantial degree of breathlessness and fatigue, with a mean Dyspnoea‐12 score of 15 (out of 36) and a mean Fatigue Severity Scale score of 4.1 (out of 7).

Initial quantitative data PROM from this large qualitative study demonstrates a substantial burden of disease in PH‐CLD. The forthcoming qualitative analysis results will provide crucial context to these findings, helping to define what holistic, patient‐centred care should look like for this distinct and growing population.

## A041 False PROMises: The Evidence Gap in Patient‐Reported Outcomes in Pulmonary Hypertension with Interstitial Lung Disease (PH‐ILD)

Dr Christopher Chew^2^, Dr Stephen John Wort^2^, Dr Iain Armstrong^1^

^1^Pulmonary Hypertension Association UK, Sheffield, UK, ^2^National Pulmonary Hypertension Service at the Royal Brompton Hospital, London, UK

Patient‐reported outcome measures (PROMs) have had an increasing role in clinical trials, regulatory and health technology assessments. However, their actual value in truly reflecting the patient experience and priorities, particularly in orphan diseases, is unclear. For instance, in the INCREASE trial, inhaled treprostinil improved six‐minute walk distance but, in contrast, did not alter the chosen patient‐reported outcome measure (PROM) in patients with Pulmonary Hypertension associated with Interstitial Lung Disease (PH‐ILD). To understand this apparent disconnect in PH‐ILD, we examined the prior use of PROMs, and existing foundational evidence on lived patient experiences.

We performed two concurrent evidence syntheses. First, a systematic review was conducted to identify and assess the application, justification, and measurement properties of all PROMs used in PH‐ILD interventional trials. Second, a scoping review mapped all existing primary qualitative research to determine the extent to which the PH‐ILD lived experience has been formally investigated. Our systematic review revealed a haphazard and poorly explained application of various PROMs across past trials. Instrument selection was inconsistent, poorly justified and critically, no population‐specific Minimal Clinically Important Difference (MCID) has been established for any PROM in PH‐ILD. The complementary scoping review identified only three, limited studies that conducted primary qualitative research into the patient experience in PH‐ILD. The choice of PROMs in interventional trials in PH‐ILD to date has been inconsistent, with a lack of foundational validation studies in this population. This is compounded by a paucity of qualitative research into the lived experience of patients with PH‐ILD. Consequently, the patient voice is not being reliably captured, rendering PROM selection arbitrary and interpretation unreliable. Future research must prioritise building outcomes from the patient experience upwards to ensure they are meaningful for this group.

## A042 Distinct Translational Dysregulation in Endothelial Cells Reveals a Unique Molecular Signature of Chronic Thromboembolic Pulmonary Hypertension

Ms Miriam Peracaula^1^, Dr. Daniel Aguilar^2^, Ms Àngela Vea^3^, Dra Cristina Rodríguez^3^, Mr Miquel Gratacós‐Aurich^1^, Dr Jeisson Osorio^3,4^, Dra Isabel Blanco^3,4^, Dr Victor I. Peinado^3,4,5^, Dr Joan Carles García‐Pagan^6,7^, Dr Héctor García Calderó^6,7^, Dra Virginia Hernández Gea^6,7^, Dra Ana Paula Dantas^8^, Dra Maribel Diaz‐Ricart^9,10^, Dra Fatima Crispi^11,12^, Dr Joan Albert Barberà^3,4^, Dra Olga Tura‐Ceide^1,3,4,13^

^1^Translational Research Group on Cardiovascular Respiratory Diseases (CAREs), Dr. Josep Trueta University Hospital de Girona, Santa Caterina Hospital de Salt and the Girona Biomedical Research Institute (IDIBGI‐CERCA), 17190 Girona, Spain, ^2^Centro de Investigación Biomédica en Red de Enfermedades Hepáticas y Digestivas (CIBEREHD), Barcelona, Spain, ^3^Department of Pulmonary Medicine, Hospital Clínic‐Institut d'Investigacions Biomèdiques August Pi I Sunyer (IDIBAPS), University of Barcelona, Villarroel, 170, 08036, Barcelona, Spain, ^4^Biomedical Research Networking Centre on Respiratory Diseases (CIBERES), 28029, Madrid, Spain, ^5^Department of Experimental Pathology, Institut d'Investigacions Biomèdiques de Barcelona (IIBB), CSIC‐IDIBAPS, Rosselló, 161, 08036, Barcelona, Spain, ^6^Barcelona Hepatic Hemodynamic Laboratory, Liver Unit, Hospital Clínic, Clínic Barcelona, Health Care Provider of the European Reference Network on Rare Liver Disorders (ERN‐RareLiver), CSUR Centro de referencia del Sistema Nacional de Salud en Enfermedad Hepática Vascular Compleja en adultos. Barcelona, Spain. AGAUR SGR2021 01115, ^7^Fundació de Recerca Clínic Barcelona‐Institut d'Investigacions Biomèdiques August Pi i Sunyer (FRCB‐IDIBAPS), Barcelona, Spain. Centro de Investigación Biomédica en Red Enfermedades Hepáticas y Digestivas (CIBEREHD), Madrid, Spain. Medicine Department, Faculty of Medicine, and health Science, Universitat de Barcelona, Barcelona, Catalonia, Spain, ^8^Cardiovascular Institute, Hospital Clinic, Institut d'Investigacions Biomèdiques August Pi i Sunyer (IDIBAPS), University of Barcelona, 08036 Barcelona, Spain, ^9^Hematopathology, Centre Diagnòstic Biomèdic (CDB), Hemotherapy‐Hemostasis Department, Hospital Clinic, Institut d'Investigacions Biomèdiques August Pi i Sunyer (IDIBAPS), University of Barcelona, 08036 Barcelona, Spain, ^10^Barcelona Endothelium Team (BET), 08036 Barcelona, Spain, ^11^BCNatal|Fetal Medicine Research Center, Hospital Clínic and Hospital Sant Joan de Déu, Institut d'Investigacions Biomèdiques August Pi i Sunyer (IDIBAPS), University of Barcelona, 08036 Barcelona, Spain, ^12^Centre for Biomedical Research on Rare Diseases (CIBER‐ER), Instituto de Salud Carlos III, 28029 Madrid, Spain, ^13^Department of Biological Sciences, Faculty of Science, University of Girona, 17003 Girona, Spain

Pulmonary arterial hypertension (PAH) and chronic thromboembolic pulmonary hypertension (CTEPH) are rare, severe forms of pulmonary hypertension marked by elevated mean pulmonary arterial pressure (mPAP) and poor survival outcomes, with a 20% mortality rate at 3 years. While PAH is characterized by progressive obliterative vasculopathy, CTEPH results from persistent obstruction of pulmonary arteries by fibrotic thrombotic material, triggering vascular remodeling, right ventricular hypertrophy, and eventual heart failure. This study aimed to identify and compare the molecular dysregulations in endothelial colony‐forming cells (ECFCs) derived from PAH and CTEPH patients relative to healthy‐controls, with a particular focus on uncovering distinct transcriptomic profiles and ultrastructural features that could elucidate disease‐specific mechanisms and therapeutic targets. Additionally, the effect of Riociguat, the only approved treatment for inoperable or persistent post‐surgical CTEPH was evaluated. Transcriptomic profiling via gene expression analysis and ultrastructural examination by transmission electron microscopy (TEM) were performed on ECFCs from PAH, CTEPH patients, and controls. The impact of Riociguat on gene expression and mitochondrial ultrastructure was assessed in CTEPH‐ECFCs. Distinct gene expression patterns were observed across the groups. Notably, CTEPH‐ECFCs exhibited a significant upregulation of genes involved in protein translation, including ribosomal proteins and elongation factors (RPS11, RPS2, RPL23, RPL23A, RPL12, RPL4, RPL21, EEF2, RPS4Y1), which was absent in PAH‐ECFCs and controls‐ECFCs. TEM revealed structural abnormalities in mitochondria‐associated endoplasmic reticulum membranes in CTEPH‐ECFCs. Riociguat treatment did not substantially reverse these translational gene expression changes or mitochondrial structural defects. These findings reveal dysregulated protein biosynthesis as a key pathophysiological hallmark that differentiates CTEPH from PAH. This distinct molecular signature underscores the limitations of current Riociguat therapy and highlights the urgent need for novel targeted treatments addressing the unique translational and mitochondrial dysfunctions in CTEPH. These insights enable development of CTEPH‐specific biomarkers and therapies.

## A043 Clinical Targeting of Fibrosis by 89Zr‐labelled anti‐PDGFRβ Fibrobody® as Visualized by Total Body PET‐CT

Drs Sylvia Klomp^1^, Prof. dr. Harm Jan Bogaard^1^, Prof. dr. Guus van Dongen^2^, Dr. Bart Nijmeijer^2^, Rychon van Dasselaar^2^, Joey Muns^2^, Dr. Yvet Noordman^2^, Dr. Xiaoqing Sun^1^, Prof. dr. Ronald Boellaard^3^, Dr. Ben Zwezerjnen^3^, Prof. dr. Daniëlle Vugts^3^, Prof. dr. Anton Vonk Noordegraaf^1^

^1^Department of Pulmonary Medicine, Amsterdam Umc, Amsterdam, The Netherlands, ^2^LinXis Biopharmaceuticals, Amsterdam, The Netherlands, ^3^Department of Radiology and Nuclear Medicine, Amsterdam UMC, Amsterdam, The Netherlands

Pulmonary arterial hypertension (PAH) is a fatal progressive lung disease, characterized by increased pulmonary vascular resistance attributed to vasoconstriction, and fibrotic remodeling of pulmonary arterioles and right ventricle by cells expressing platelet derived growth factor receptor beta (PDGFRβ). Thus, PDGFRβ may serve as a target for both staging and treatment of PAH. Therefore, the VHH‐based construct SP02SP26‐ABD (Fibrobody®) was developed. It displays high expression and species cross‐reactivity towards PDGFRβ, and can be functionalized with diagnostic or therapeutic payload. In the preclinical SuHx rat model of PAH, and in patient samples, we confirmed high expression of PDGFRβ in remodeled regions of lungs and hearts with co‐expression of fibrosis‐related markers collagen and αSMA. In the SuHx model, using an 89Zr‐based radioactive tracer approach for PET‐CT imaging, SP02SP26‐ABD was confirmed to selectively accumulate in the affected organs. The preclinical evidence that PDGFRβ is a suitable marker for remodeling in PAH, and SP02SP26‐ABD and a suitable vector for PDGFRβ targeting in vivo. Now, we will investigate by PET‐CT imaging whether SP02SP26‐ABD is capable of selectively targeting fibrotic remodelling in the lungs and heart of PAH patients, using the same radioactive tracer approach. The study is a two‐phased prospective observational study. In phase one, patients will be dosed and subjected to total body PET‐CT scanning at multiple time points post‐dosing, to examine tracer protein dose and time‐dependency of biodistribution and to determine the most suitable imaging time point. Patients will also be monitored for safety, and pharmacokinetic analysis will be performed. Through dosimetry, the first phase will define the scanning protocol. In the second study phase, PAH and non‐PAH carriers of the BMPR2 gene mutation, as well as healthy family members (DOLPHIN‐GENESIS cohort) will be subjected to the defined protocol, to evaluate whether SP02SP26‐ABD is a suitable vector for diagnosis (and potentially treatment) of PAH.

## A044 Effects of Sotatercept on Echocardiographic (ECHO) Parameters of Right Ventricular (RV) Function: Results from the Randomized, Placebo‐Controlled, Double‐Blind, Phase 3 HYPERION Study of Adults Within 1 Year of Pulmonary Arterial Hypertension (PAH) Diagnosis and at Intermediate‐to‐High Risk of Mortality

Vallerie McLaughlin^1^, Marius Hoeper^2,3^, David Badesch^4^, H. Ardeschir Ghofrani^5,6,7^, Simon Gibbs^8^, Mardi Gomberg‐Maitland^9^, Ioana Preston^10^, Rogerio Souza^11^, Aaron Waxman^12^, Yaru Shi^13^, Wei Fu^13^, Chih‐Chin Liu^13^, Barry Miller^13^, Samuel Kim^13^, Michela Brambatti^13^, Alexandra Cornell^13^, Marc Humbert^14,15,16^

^1^Department of Internal Medicine, Division of Cardiovascular Medicine, University of Michigan, Ann Arbor, USA, ^2^Department of Respiratory Medicine and Infectious Diseases, Hannover Medical School, Hannover, Germany, ^3^Member of the German Center for Lung Research, Biomedical Research in EndStage and Obstructive Lung Disease Hannover, Hannover, Germany, ^4^Pulmonary Hypertension Program, University of Colorado, Anschutz Medical Campus, Aurora, USA, ^5^Department of Internal Medicine, Justus‐Liebig University Giessen, Giessen, Germany, ^6^Universities of Giessen and Marburg Lung Center and Institute for Lung Health, Giessen, Germany, ^7^Member of the German Center for Lung Research, Giessen, Germany, ^8^National Heart and Lung Institute, Imperial College London, London, United Kingdom, ^9^Division of Cardiovascular Medicine, George Washington University School of Medicine and Health Sciences, Washington, DC, USA, ^10^Pulmonary and Critical Care Medicine, Lahey Hospital and Medical Center, Burlington, USA, ^11^Divisão de Pneumologia, Instituto do Coração, Hospital das Clínicas da Faculdade de Medicina da Universidade de São Paulo, São Paulo, Brazil, ^12^Pulmonary and Critical Care Medicine, Brigham and Woman's Hospital, Harvard Medical School, Boston, USA, ^13^Merck & Co., Inc., Rahway, USA, ^14^Université Paris‐Saclay, Faculté de Médecine, Pulmonary Hypertension: Pathophysiology and Novel Therapies, Le Kremlin‐Bicêtre, France, ^15^INSERM UMR_S 999 “Pulmonary Hypertension: Pathophysiology and Novel Therapies”, Hôpital Marie Lannelongue, Le Plessis‐Robinson, France, ^16^Department of Respiratory and Intensive Care Medicine, Publique Hôpitaux de Paris, Hôpital Bicêtre, ERN‐LUNG, Le Kremlin‐Bicêtre, France

Sotatercept is a first‐in‐class activin signaling inhibitor approved for PAH. Sotatercept improves the balance between anti‐ and pro‐proliferative signaling in the pulmonary vasculature, aiming to reverse the vascular remodeling associated with PAH. HYPERION (NCT04811092) evaluated sotatercept in patients (< 1 year since diagnosis) at intermediate‐to‐high risk of mortality and met its primary objective of a reduction in time to clinical worsening events. As RV dysfunction is the principal determinant of morbidity/mortality in PAH, we assessed ECHO data on RV function from HYPERION.

Participants with WHO functional class II/III PAH at intermediate‐to‐high risk of mortality (REVEAL Lite 2 risk ≥ 6 or COMPERA 2.0 risk ≥ 2) on stable background therapy were randomized to sotatercept (n = 160) or placebo (n = 160) Q3W. Centrally read ECHO parameters are reported as observed from participants with paired baseline and Week 24 values. Most participants were women (72.5%), median age was 60 years, and most (72.2%) were receiving double background therapy. Relative to placebo, sotatercept showed improvements (median change from baseline) in systolic pulmonary artery (PA) pressure (sPAP, mmHg; −18.61 [n = 62] versus +0.16 [n = 102]), RV area at end‐diastole (RVA‐ED, cm²; −4.55 [n = 140] versus −1.36 [n = 144]), RV fractional area change (RVFAC, %; +6.49 [n = 140] versus +0.99 [n = 144]), and tricuspid annular plane systolic excursion (TAPSE)/sPAP (mm/mmHg; +0.13 [n = 55] versus +0.01 [n = 93]). No notable between‐group changes from baseline were seen for TAPSE or estimated right atrial pressure. In patients with PAH (< 1 year since diagnosis) at intermediate‐to‐high risk of mortality, add‐on sotatercept demonstrated improvements in multiple (Week 24) ECHO indices of RV function, including TAPSE/sPAP, a marker of RV–PA coupling and possible RV remodeling. The improvements observed in RV function may explain some of the benefits seen with sotatercept in HYPERION and further support early initiation of sotatercept as add‐on treatment following PAH diagnosis.

## A045 Pulmonary Arterial Hypertension in Latin America: Patient Characteristics, Therapy, and Survival — A Systematic Review and Meta‐Analysis of Regional Cohorts and Registries

Dr. Marcos Fernandes Garcia, Dr. Katherine Julian, Dr. Tim Lahm, Prof. Rogerio Souza

^1^University of Colorado Anschutz Medical Campus, Aurora, United States

There is limited data on pulmonary arterial hypertension (PAH) in Latin America and Caribbean (LAC). To address this, we conducted a systematic review and single‐arm meta‐analysis to characterize patient demographics, clinical features, treatments, and survival across LAC cohorts and compared these findings with international registries. We searched databases in English, Spanish, and Portuguese without date restrictions. Eligible studies enrolled adults with right heart catheterization (RHC)–confirmed PAH in LAC. Study‐level data on demographics, clinical and hemodynamic variables, treatments, and survival were pooled using random‐effects models. Sixteen studies from eight countries (1999–2025) including 2,553 patients (79% incident) were analyzed. Patients were young (mean age 43.6 years), predominantly female (81.5%), and had heterogeneous etiologies: idiopathic (40.3%), congenital heart disease–associated (25.6%), connective tissue disease–associated (20.6%), and schistosomiasis‐associated (15.3% in Brazilian cohorts). Overall, 51% were in WHO functional class III/IV, with a six‐minute walk distance of 406 m. Hemodynamics showed mean pulmonary arterial pressure 55.8 mmHg, right atrial pressure 9.8 mmHg, cardiac index 2.63 L/min/m², and brain natriuretic peptide 228 pg/mL, reflecting an intermediate‐risk profile. Treatment patterns, while heterogeneous due to variable timing of treatment initiation, were primarily dominated by monotherapy (54.7%), with limited use of dual (32.7%) and triple therapy (4.3%). Pooled survival was 93.9% at 1 year, 87.5% at 2 years, and 83.0% at 3 years. This first comprehensive characterization of PAH in LAC establishes a regional benchmark and exposes key regional disparities. Patients are young predominantly female, and disproportionately affected by CHD‐ and schistosomiasis‐associated PAH. Survival was similar to international registries, but may reflect younger, healthier patients rather than comparable PAH care. Key priorities moving forward include earlier recognition and referral, systematic use of risk assessment tools, access to CHD repair programs, schistosomiasis screening, and expanded access to RHC and PAH‐specific therapies.

## A046 ORAL ABSTRACT: Distinct RV vs. LV Macrophage Stress Responses Under Uniform Hypoxia

Dr Sue Gu^1,2^, Alyse Staley^3^, Dr. Claudia Mickael^1,2^, Linda Sanders^1,2^, Dr. Tim Lahm^4,5^, Dr. Kurt Stenmark^2^

^1^Division of Pulmonary, Allergy and Critical Care Medicine, Department of Medicine, University of Colorado Anschutz Medical Campus, Aurora, United States of America, ^2^Cardiovascular Pulmonary Research Laboratory, University of Colorado School of Medicine, Department of Pediatrics‐Critical Care Medicine, Aurora, United States of America, ^3^University of Colorado Anschutz Medical Campus Cancer Center Bioinformatics Core Facility, Aurora, United States of America, ^4^Division of Pulmonary, Critical Care and Sleep Medicine, Department of Medicine, National Jewish Health, Denver, United States of America, ^5^Rocky Mountain Regional Veterans Affairs Medical Center, Aurora, United States of America

Right ventricular (RV) function is the key determinant of survival in pulmonary hypertension (PH), yet RV immunobiology is incompletely defined. Left‐ventricular (LV) injury studies implicate CCR2⁺ macrophages in monocyte recruitment and remodeling, but whether RV macrophages deploy similar or distinct programs is unknown. We hypothesized that under uniform hypoxia the RV and LV mount different macrophage stress responses. C57BL/6 mice were exposed to hypobaric hypoxia (18,000 ft) for 3, 7, or 21 days (n = 4/timepoint, balanced by sex). RV and LV were dissected, enzymatically digested, and viable cells isolated by flow cytometry. Fixed cells underwent 10x Genomics Chromium Fixed RNA Profiling. Data were integrated and clustered (Seurat); macrophage subtypes were identified by canonical markers. Cluster‐level differential expression (MAST hurdle), RV–LV contrasts across time, GO‐based GSEA, and ligand–receptor analyses quantified transcriptional programs and intercellular communication. Despite uniform hypoxia, RV and LV macrophages diverged in a time‐dependent manner. At 3 days, RV macrophages upregulated interferon and chemokine modules (Ifit1/2/3, Isg15, Rsad2, Irf7; Ccl2/Ccl7), antigen‐processing nodes (Tap1, Cd274), and a CCR2 recruitment signature, while LV induction was muted. At 7 days, RV retained interferon tone and expanded antigen presentation/immune interaction pathways (Ciita, H2‐DMa/b1, Ctss) with early matrix engagement (Fn1, Tgfbi); LV remained comparatively homeostatic. At 21 days, RV transitioned to ECM/integrin programs (Fn1, Col1a1/2, Postn, Ltbp2/3), coincident with stronger RV macrophage‐to‐fibroblast/endothelial communication; LV networks remained attenuated. Collectively, RV responses reflect combined pressure/afterload and oxidative stress, driving CCR2‐mediated recruitment and matrix remodeling, whereas LV showed a different, relatively homeostatic hypoxic response. These findings demonstrate that under uniform hypoxia, RV macrophages mount a distinct immune‐metabolic response driven by biomechanical and oxidative stress, in contrast to the relatively quiescent LV. RV‐specific macrophage activation emerges as a key determinant of ventricular remodeling and a potential therapeutic target in pulmonary hypertension.

## A047 Single Nucleus Rna Profiling of Right Ventricular Adaptation to Congenital Pressure Overload

Md Michael Smith^1^, MD Jason Boehme^1^, MD, PhD Sanjeev Datar^1^, MD Jonathan Hyde^1^, MD John Hwang^1^, MD Naveen Swami^2^, MD, PhD Emin Maltepe^3^, MD Gary Raff^4^, Carsten Knutsen^1^, PhD Daoqin Zhang^1^, MD Jeffrey Fineman^1^, MD Cristina Alvira^1^

^1^Division of Pediatric Critical Care, Department of Pediatrics, University Of California, San Francisco, San Francisco, United States, ^2^Division of Pediatric Cardiothoracic Surgery, Department of Surgery, University of California, San Francisco, United States, ^3^Division of Neonatology, Department of Pediatrics, University of California, San Francisco, San Francisco, United States, ^4^Division of Pediatric Cardiothoracic Surgery, Department of Surgery, University of California, Davis, Davis, United States

Patients with congenital heart disease‐associated pulmonary arterial hypertension (CHD‐PAH) have superior right ventricular (RV) function and improved outcomes compared to patients with other forms of PAH. In utero, the RV is the dominant ventricle, but with the perinatal transition the RV is typically offloaded. A better understanding of the mechanisms underlying RV adaptation to congenital pressure overload in CHD may reveal novel therapeutic targets for supporting RV function. In an ovine model of CHD‐PAH created via fetal implantation of an aortopulmonary graft (shunt lambs), we have previously demonstrated the development of an adaptively hypertrophied RV with enhanced physiologic performance. In the present study, we performed single nucleus RNA sequencing of RV harvested from four‐week‐old shunt (n = 2), age‐matched control (n = 2), and unoperated fetal lambs (n = 2) to better understand cell type‐specific transcriptional adaptations to congenital pressure overload. A total of 74,864 nuclei were isolated and sequenced according to the standard 10x Genomics workflow (29,661 shunt, 22,034 control, and 23,169 fetal). Cardiomyocytes were the predominant cell type, composing 73.5% of fetal, 64.2% of shunt, and 45.3% of control nuclei (p < 0.001). Cardiomyocyte transcriptomes were further evaluated in single nucleus trajectory analysis. A clear continuum was evident across pseudotime, with fetal cardiomyocytes the most naïve, control the most mature, and shunt intermediate. Genes identified to be significantly regulated over the course of this fetal‐shunt‐control trajectory were subjected to Gene Ontology enrichment analysis. Cardiac muscle contraction (GO:0055007) and response to calcium ion (GO:0051592) were among the top biological processes enriched by genes differentially regulated across pseudotime (adjusted p < 0.01). This initial profiling of the RV's response to congenital pressure overload highlights transcriptional evidence for an adaptive persistence of the fetal RV phenotype marked by a relative increase in the proportion of cardiomyocytes and transcriptional changes to support RV contractile function and calcium handling in CHD‐PAH.

## A048 Exploring the Role of Neutrophil‐Percentage‐to‐Albumin Ratio (NPAR) in Pulmonary Arterial Hypertension (PAH)

Professor Habil Andreea Varga^1^, Professor Habil Ioan Tilea^1^, Asistant Prof. Liviu Cristescu^1^, PhD student Dragos Gabriel Iancu^1^, Lecurer Razvan Gheorghita Mares^1^, Assist prof. Andreea Diana Moldovan^1^, PhD student Bianca Larisa Abalasei^1^

^1^George Emil Palade, University Of Medicine, Pharmacy, Science And Technology, Targu Mures, Romania

Systemic inflammation and congestion are established drivers of adverse outcomes in pulmonary arterial hypertension (PAH). The neutrophil percentage‐to‐albumin ratio (NPAR), a composite biomarker reflecting both inflammatory burden and nutritional–congestive status, has not been previously evaluated in PAH. We aimed to assess the prognostic value of NPAR for overall mortality, defined as in‐hospital and 90‐day post‐discharge death. We analysed 282 consecutive PAH hospitalisations recorded between 2014 and 2024 in a single tertiary referral centre in Romania. The cohort was predominantly female (n = 171, 61%). NPAR was benchmarked against established prognosticators: log‐transformed NT‐proBNP, WHO functional class (FC I–II vs. III–IV), and six‐minute walking distance (6MWD). NPAR values ranged from 9.9 to 40.1 (mean 17.8 ± 5.7). Higher NPAR correlated moderately with WHO functional class (Spearman r = 0.32, p = 0.007), but not with NT‐proBNP or 6MWD, suggesting that it reflects a distinct biological axis. In univariable logistic regression, elevated NPAR independently predicted overall mortality (OR 1.19 per unit, 95% CI 1.08–1.30, p = 0.003) with good calibration (Hosmer–Lemeshow p = 0.901). In multivariable backward regression, only NPAR and WHO functional class (III–IV) remained significant predictors, yielding a robust overall model fit (ANOVA p < 0.001). Receiver‐operating characteristic (ROC) analysis demonstrated strong discriminative ability for NPAR (AUC 0.878, p < 0.001). A cut‐off of 21.5 (Youden index) provided 80.0% sensitivity and 86.6% specificity. Comparative ROC analysis confirmed that NPAR performed non‐inferiorly to WHO functional class and 6MWD, and significantly outperformed NT‐proBNP (p = 0.032 for AUC difference). NPAR, an inexpensive and readily available composite biomarker, is strongly associated with short‐term mortality in PAH and provides complementary prognostic information to functional and biochemical indices. Validation of the proposed cut‐off (21.5) in larger, independent cohorts is warranted prior to clinical translation.

## A049 PHYRE‐RO: Romania's First National Pulmonary Hypertension Registry — A Clinical Infrastructure for Benchmarking and Quality Improvement

Professor Habil Ioan Tilea^1^, Professor Habil Andreea Varga^1^, Assistant Professor Liviu Cristescu^1^, Lecturer Razvan Gheorghita Mares^1^, PhD Student Dragos Gabriel Iancu^1^, Assistant Professor Diana Andreea Moldovan^1^

^1^George Emil Palade University Of Medicine, Pharmacy, Science And Technology Of Targu Mures, Romania, Targu Mures, Romania

Clinical registries are central to advancing pulmonary hypertension (PH) research, benchmarking care, and informing health policy. Worldwide initiatives have shaped prognostic modelling, validated risk stratification, and provided essential epidemiological evidence. Despite increasing PH burden, Romania has lacked a national registry, limiting standardisation of care and participation in global collaborations. We established PHYRE‐RO, Romania's first national PH registry. It is a cloud‐hosted, ISO‐27001 compliant, GDPR‐by‐design platform, interoperable via HL7/FHIR standards. Structured electronic case report forms (eCRFs) support longitudinal data collection at 3, 6, 12, and 24 months. Governance is ensured by a national Steering Committee, with centre‐level data ownership and aggregated reporting under Ministry of Health oversight. Ethical approval, e‐consent, pseudonymisation, role‐based access, and audit trails guarantee compliance, transparency, and security. Clinical Scope:The initial focus is on Group 1 pulmonary arterial hypertension (PAH) and Group 4 chronic thromboembolic pulmonary hypertension/disease (CTEPH/CTEPD), in both adult and paediatric populations. Core datasets include demographics, comorbidities, diagnostics (RHC, imaging, biomarkers), and therapies. Patient‐reported outcomes comprise EmPHasis‐10 as the core PH‐specific HRQoL instrument, CAMPHOR for comprehensive profiling, PAH‐SYMPACT in PAH research, and EQ‐5D‐5L for generic HRQoL and economic analyses WHO functional class and 6MWD are recorded as clinician‐assessed endpoints. Other outcomes include hospitalisations, transplantation, BPA, PEA, and survival. Key performance indicators assess diagnostic timeliness, multidisciplinary team review, therapy escalation, and clinical outcomes.

Innovation/Impact: PHYRE‐RO operationalises ESC/ERS risk frameworks, embedding automated data validation, dashboards, and national reporting. Benchmarkable against international registries yet tailored to Romanian healthcare, it aims to enable earlier diagnosis, equitable referral, risk‐appropriate therapy, improved functional status, and reduced mortality. Crucially, the registry is designed for registry‐based clinical trials, strengthening Romania's role in global PH research.:PHYRE‐RO is a milestone for Romanian cardiology and pulmonology, creating the first national PH registry and establishing a robust clinical research and quality‐improvement infrastructure aligned with international standards.

## A050 Prognostic Value of the Hemoglobin‐to‐Red Cell Distribution Width Ratio for Early Readmission in Pulmonary Arterial Hypertension and Chronic Thromboembolic Pulmonary Hypertension

Professor Habil Ioan Tilea^1^, Professor Habil Andreea Varga^1^, PhD student Dragos Gabriel Iancu^2^, Lecturer Razvan Gheorghita Mares^1^, Assistant Professor Liviu Cristescu^1,2^, Assistant Professor Diana Andreea Moldovan^1,2^

^1^George Emil Palade University Of Medicine, Pharmacy, Science And Technology Of Targu Mures, Romania, Targu Mures, Romania, ^2^Doctoral School, George Emil Palade University Of Medicine, Pharmacy, Science And Technology Of Targu Mures, Romania, Targu Mures, Romania

Early readmission in pulmonary arterial hypertension (PAH) and chronic thromboembolic pulmonary hypertension (CTEPH) increases morbidity, costs, and reduces quality of life, while readmission risk prediction remains suboptimal. The red cell distribution width (RDW) has been linked to prognosis in pulmonary hypertension (PH), while the haemoglobin‐to‐RDW ratio (HRR; haemoglobin [g/dL] divided by RDW‐CV [%]) has emerged as a robust prognostic marker in heart failure. However, the prognostic role of HRR in PH has not been previously investigated. We retrospectively analysed 80 patients (54 PAH, 26 CTEPH) with 468 hospitalisations at a national PH referral centre. HRR was derived from admission blood counts. The primary endpoint was 30‐day readmission. Logistic regression with patient‐level clustering was performed separately for PAH and CTEPH. Models were adjusted for NT‐proBNP, 6MWD, WHO functional class, TAPSE, and eGFR. Predictive performance was assessed by ROC analysis. Readmitted patients had significantly lower HRR compared with those not readmitted. In univariate analysis, HRR predicted 30‐day readmission in PAH (OR 0.056, 95%CI 0.007–0.438, p = 0.006) and CTEPH (OR 0.045, 95%CI 0.005–0.433, p = 0.007). After adjustment, HRR remained independently associated with readmission in CTEPH (OR –0.415, 95%CI –0.724‐0.106, p = 0.009), together with NT‐proBNP (p = 0.028) and eGFR (p = 0.002). In PAH, NT‐proBNP (p = 0.024) was the strongest predictor, while HRR lost significance. ROC analysis showed fair discrimination for HRR in both groups (AUC:0.664 in PAH, cut‐off≤ 0.73; AUC:0.673 in CTEPH, cut‐off≤ 0.81). In PAH, 6MWD (AUC:0.729, p < 0.001) and NT‐proBNP (AUC:0.707, p = 0.007) were stronger predictors, whereas in CTEPH, TAPSE (AUC:0.647, p = 0.020) and HRR (AUC:0.673, p = 0.004) were more relevant.

Lower HRR was associated with increased 30‐day readmission in both PAH and CTEPH, with independent prognostic value in CTEPH. As a simple biomarker from routine blood counts, HRR may complement established risk factors to improve early risk stratification and guide closer follow‐up in PH.

## A051 Dissecting the Role of NDUFA4L2 in Pulmonary Arterial Smooth Muscle Cells Exposed to Chronic Hypoxia and Pulmonary Hypertension

Mr Sandipan Jash^1^, Mr Arjhang Adeli^1^, Miss Vidya Lakshmi^1^, Dr Fenja Knoepp^1^, Dr Oleg Pak^1^, Prof Dr Werner Seeger^1^, Prof Dr Norbert Weissmann^1^, Dr Luca Giordano^1^, Prof Dr Natascha Sommer^1^

^1^Excellence Cluster Cardio‐Pulmonary Institute (CPI), Universities of Giessen and Marburg Lung‐Center (UGMLC), Member of the German Center for Lung Research (DZL), Justus‐Liebig University, Giessen, Germany, ^2^Institute for Lung Health (ILH), Giessen, Germany

Pulmonary hypertension (PH) is caused by vascular remodelling of the pulmonary arteries (PA) induced by resistance to apoptosis and hyperproliferation of pulmonary arterial smooth muscle cells (PASMC). This phenotype is sustained by decreased mitochondrial respiration (MR) and increased anaerobic glycolysis, leading to a metabolic adaptation (MA). The mitochondrial NADH dehydrogenase 1□ subcomplex subunit 4‐like 2 (NDUFA4L2) is upregulated in several cell types exposed to chronic hypoxia (CH); however, its role in the MA of the PASMC exposed to CH and PH remains unclear. RNAseq, western blot (WB) and immunostaining of the pulmonary arteries (PA) were used to quantify NDUFA4L2 in mice exposed to CH (10% O₂) and in lung biopsies of patients with idiopathic PH (IPAH). qPCR and WB were used to detect NDUFA4L2 expression in isolated mouse and human PASMC exposed to CH (1% O₂), and high‐resolution respirometry to examine MR. The functional role of NDUFA4L2 in PASMC during CH was tested by siRNA‐mediated downregulation, followed by cell proliferation (CelPro) and scratch assays, and WB to explore proliferative (pAKT, pERK), apoptotic (BAX, cleaved PARP, cleaved caspase‐3) and metabolic (PDHK1, LDHA, PC, OXPHOS) markers. NDUFA4L2 protein was upregulated in the lung homogenate and PA of mice exposed to CH, and in the lung biopsies from IPAH patients. In vitro, NDUFA4L2 mRNA and protein were upregulated in mouse and human PASMC, along with decreased MR. Silencing NDUFA4L2 in PASMC exposed to CH decreased CelPro and wound healing, increased protein levels of apoptotic markers and restored mitochondrial complex I and III subunits, and pyruvate carboxylase. Our results support a direct role of NDUFA4L2 in the pathogenesis of CH‐induced PH. It controls mitochondrial function, apoptosis, and CelPro of the PASMC during CH. Future investigations using NDUFA4L2 KO mice will clarify its role in the pathophysiology of PH.

## A052 Limited Agreement Between Non‐Invasive and Invasive CPET Metrics in Chronic Pulmonary Embolism

Dr. Beatraiz Deberaldini‐Marinho^1^, Dr. S. Mehdi Nouraie^1^, Dr. Michael Risbano^1^

^1^University of Pittsburgh Medical Center, Pittsburgh, USA

Persistent exertional symptoms after acute pulmonary embolism (PE) are frequent and may reflect chronic thromboembolic pulmonary disease (CTEPD). Non‐invasive cardiopulmonary exercise testing (CPET) is often employed to screen for physiologic impairment, but algorithms such as SEARCH and FOCUS rely on indirect estimates of dead space ventilation and stroke volume augmentation. These surrogates have not been rigorously validated against invasive CPET (iCPET), the gold standard for physiologic phenotyping. We sought to assess diagnostic concordance between non‐invasive CPET algorithms and iCPET‐derived measures. We retrospectively evaluated 42 post‐PE patients who underwent iCPET with concurrent imaging (ventilation–perfusion scintigraphy and/or CT pulmonary angiography). Non‐invasive measures of dead space ventilation (VD/VT at rest > 0.38 [SEARCH]; VE/V̇CO₂ slope or nadir ≥ 30 [FOCUS]) were compared with iCPET‐derived VD/VT at peak exercise > 0.28. Surrogates for stroke volume augmentation (O₂ pulseAT:rest ≤ 2.37 [SEARCH]; peak O₂ pulse < 80% predicted [FOCUS]) were compared with iCPET stroke volume ratio (SVAT:rest < 28%). FOCUS criteria for elevated dead space demonstrated moderate sensitivity (VE/V̇CO₂ slope 75.0%, nadir 70.8%) but limited specificity (50.0%). SEARCH thresholds for VD/VT performed less well (sensitivities and specificities 50–58%). Stroke volume surrogates showed poor agreement with invasive measures: SEARCH (sensitivity 29.2%, specificity 41.7%) and FOCUS (sensitivity 79.2%, specificity 41.7%). Direct comparison revealed significant discordance between SEARCH and FOCUS stroke volume surrogates (p = 0.0012), indicating classification of distinct physiologic subgroups. Non‐invasive CPET algorithms show moderate ability to detect elevated dead space but fail to reliably identify impaired stroke volume augmentation compared with iCPET. These findings highlight the limitations of algorithm‐based screening and reinforce the value of iCPET for accurate physiologic characterization in post‐PE patients.

## A053 Discordance in Exercise Pulmonary Hypertension Definitions After Pulmonary Embolism: Slope vs. ΔmPAP/ΔCO

Fellow Jasmine Vakhshoorzadeh^1^, Beatriz Deberaldini Marinho^1^, S. Mehdi Nouraie^1^, Dr. Michael Risbano^1^

^1^University Of Pittsburgh Medical Center, Pittsburgh, USA

Persistent exertional symptoms after pulmonary embolism (PE) are common and may reflect chronic thromboembolic pulmonary disease (CTEPD). Invasive cardiopulmonary exercise testing (iCPET) detects exercise pulmonary hypertension (ePH) in patients with normal resting hemodynamics. Current guidelines define ePH as mPAP/CO slope > 3 mmHg·min·L⁻¹, but it remains unclear whether slope or rest‐to‐peak ΔmPAP/ΔCO more accurately reflects physiologic burden. We retrospectively analyzed 39 symptomatic patients > 3 months after acute PE (27 with chronic PE by VQ, 12 with resolved PE). CTEPH was excluded. All underwent incremental iCPET. ePH was defined both by mPAP/CO slope and by ΔmPAP/ΔCO (rest–peak). In chronic PE, ePH prevalence was 3.7% (1/27) by slope versus 22.2% (6/27) by ΔmPAP/ΔCO. In resolved PE, prevalence was 8.3% (1/12) and 16.7% (2/12), respectively. Patients identified only by ΔmPAP/ΔCO had lower peak VO₂, watts, pulmonary vascular compliance, and distensibility compared with non‐ePH chronic PE, indicating physiologically meaningful disease that was missed by slope criteria alone. Detection of ePH in post‐PE patients is highly definition‐dependent. A ΔmPAP/ΔCO criterion identifies a greater proportion of patients with impaired vascular reserve than slope alone, reducing misclassification and aligning with prior evidence that exercise‐only pulmonary vascular abnormalities in CTEPD are clinically relevant. These findings emphasize that slope‐based criteria may underestimate disease burden, with potential consequences for diagnosis, patient selection for advanced therapies, and clinical trial design. Incorporating ΔmPAP/ΔCO into research frameworks—and validating it against prospective outcomes—may improve early recognition of pulmonary vascular dysfunction after PE. Refining ePH definitions in this at‐risk population is essential to ensure timely diagnosis, avoid missed opportunities for treatment, and advance consensus on exercise‐based hemodynamic standards.

## A054 Fibronectin–Integrin α5β1 Axis Orchestrates Pathological RV Remodeling in Pulmonary Hypertension

Miss Sarah‐Eve Lemay^1^, Yann Grobs^1^, Manon Mougin^1^, Sandra Breuils‐Bonnet^1^, Andréanne Pelletier^1^, Justin Bellavance^1^, Jamal Nabhanizadeh^2,3^, Roxanne Desbiens^1^, Alice Bourgeois^1^, Sandra Martineau^1^, Mélanie Sauvaget^1^, Soni Savai Pullamsetti^2,3^, Rajkumar Savai^2,3,4^, François Potus^1^, Steeve Provencher^1^, Olivier Boucherat^1^, Sébastien Bonnet^2,3^

^1^Pulmonary hypertension research group, Quebec Heart and Lungs Institute, Laval University, Quebec, Canada, ^2^Max Planck Institute for Heart and Lung Research, member of the German Center for Lung Research (DZL), member of the Cardio‐Pulmonary Institute (CPI), Bad Nauheim, Germany, ^3^Institute for Lung Health (ILH), Justus Liebig University, Giessen, Germany, ^4^Frankfurt Cancer Institute (FCI), Goethe University, and German Cancer Consortium (DKTK), Frankfurt, Germany

Right ventricular (RV) function is a key prognostic factor in pulmonary arterial hypertension (PAH). In response to pressure overload, the RV undergoes structural remodeling driven by extracellular matrix (ECM) accumulation and cardiomyocyte hypertrophy. Integrins are members of the cell adhesion receptors superfamily. Integrins transduce extracellular cues to cardiac cells through ECM binding, activating pro‐hypertrophic and pro‐fibrotic signaling pathways. We hypothesized that integrins may represent a potential therapeutic target for RV dysfunction in PAH. Using publicly available transcriptomic datasets, we identified the “ECM‐receptor interaction” pathway as significantly enriched in RVs from PAH patients and animal models. Among its components, only ITGA5 and fibronectin were consistently and significantly upregulated across all datasets. Western blot analysis confirmed increased levels of integrins α5 and β1 in RVs from PAH patients and models, correlating with disease severity. In rat cardiomyocytes, α5β1 inhibition reduced phenylephrine‐induced hypertrophy, reversed established hypertrophy induced by MCT treatment (F‐actin labeling), and enhanced contractility (sarcomere shortening, time to peak). In human RV fibroblasts (FB), inhibition of α5β1 prevented TGFβ1‐induced activation of control RVFB and decreased proliferation and activation of RVFB derived from PAH patients (WB PCNA, αSMA, COL1; Ki67). In pulmonary artery banding rats and monocrotaline‐injected rats, α5β1 inhibition improved RV function (CO, TAPSE, ejection fraction, strain; assessed by echocardiography, RHC, and cardiac MRI) and decreased RV hypertrophy and fibrosis. Ex vivo, inhibition of α5β1 attenuated cardiomyocyte hypertrophy and RV fibrosis and enhanced contractile work in human precision‐cut RV slices (biomimetic work loops with modulations in preload and afterload). Our findings suggest that integrin α5β1 plays a central role in RV dysfunction in PAH. Ongoing studies using phosphoproteomics and spatial transcriptomics aim to further define the underlying mechanisms.

## A055 Olaparib: A Potential New OPTION for Pulmonary Arterial Hypertension Therapy

Miss Sarah‐Eve Lemay^1^, Yahe Xu^1^, Pascale Blais‐Lecours^1^, Chanil Valasarajan^2^, Sandra Breuils‐Bonnet^1^, Andréanne Pelletier^1^, Magalie Boucher^1^, Sandra Martineau^1^, Soni Savai Pullamsetti^1,2,5,8^, Rajkumar Savai^1,2,5,7,8^, Olivier Boucherat^1^, François Potus^1^, John Granton^3^, Jason Weatherald^4^, Sébastien Bonnet^1,2,5,6,8^, Steeve Provencher^1,6^

^1^Pulmonary hypertension research group, Quebec Heart and Lungs Institute, Laval University, Quebec, Canada, ^2^Department of Internal Medicine, Universities of Giessen and Marburg Lung Center (UGMLC), DZL, Justus Liebig University, member of the German Center for Lung Research (DZL), Giessen, Germany, Giessen, Germany, ^3^Division of Respirology, Temerty Medicine, Faculty of Medicine, University of Toronto, Toronto, Canada, ^4^Department of Medicine, Division of Respirology, University of Calgary, Calgary, Canada, ^5^Max Planck Institute for Heart and Lung Research, Department of Lung Development and Remodeling, German Center for Lung Research (DZL), Bad Nauheim, Germany, ^6^Equally contributing corresponding author, ^7^Frankfurt Cancer Institute (FCI), Goethe University, and German Cancer Consortium (DKTK), Frankfurt, Germany, ^8^Institute for Lung Health (ILH), Giessen, Germany

Pulmonary arterial hypertension (PAH) is characterized by progressive narrowing of the distal pulmonary arteries (PAs), leading to right ventricular (RV) failure and premature death. Despite cytotoxic inflammatory environment that induces DNA damage, PA smooth muscle cells (PASMCs) adapt and continue to proliferate. Persistent activation of the DNA damage response (DDR) is a key driver of this abnormal behavior. Among DDR‐associated proteins, poly (ADP‐ribose) polymerase (PARP) enzymes promote aberrant DNA repair, survival, and proliferation. PARP can be inhibited by Olaparib, an orally available approved for the treatment of ovarian cancer. PARP inhibition also improves RV function in preclinical PAH models. To evaluate the efficacy of Olaparib in PAH in vitro and ex vivo preclinical models, and in a Phase 1b clinical trial. In PASMCs and PA endothelial cells isolated from PAH patients, Olaparib reduced cell proliferation (MCM2 expression and Ki67) and resistance to apoptosis (Survivin expression and Annexin V). PAH‐PASMCs with high baseline expression of DDR‐associated proteins (RAD51, CHK2, BRCA1) showed reduced responsiveness to Olaparib compared to PAH‐PASMCs with low expression of these proteins. In rat RV cardiomyocytes, Olaparib enhanced contractility (sarcomere shortening, time to peak). Ex vivo, Olaparib improved established vascular remodeling in human precision‐cut lung slices from PAH patients. In a Phase 1b clinical trial (OPTION, NCT03782818) involving 17 patients with severe PAH, Olaparib improved hemodynamics in 6 patients, stabilized hemodynamics in 3 patients. However, post‐baseline hemodynamics worsened in 3 patients and were unavailable in 5 patients due to premature discontinuation (mostly due to unrelated disease worsening). Olaparib exhibits PA anti‐remodeling effects in preclinical models of PAH, which are influenced by baseline DDR activation. In PAH patients, Olaparib was associated with a heterogeneous hemodynamic response. Ongoing studies aim to explore whether baseline DDR activity and additional predictive biomarkers can predict the hemodynamic response in humans.

## A056 Targeting Membrane‐Associated Human Lysyl‐tRNA Synthetase 1 (KARS1) Inhibits Monocyte/Macrophage Migration and Improves Lung Vascular Remodeling in PAH Rodent Models

Dr. Nam Hoon Kwon^3^, Ina Yoon^4,5^, Nu‐Ri Im^3^, Hyungjoo Park^3^, Daeyoung Han^6^, Junjeong Choi^4^, Yong‐Sung Kim^7^, Byung Woo Han^8^, Pilhan Kim^9^, Hyunbo Shim^10^, Hyung‐Kwan Kim^11,12^, Jun‐Bean Park^11,12^, Sunghoon Kim^3,5,6,13^, Dr David Kennard, David Kennard^MRC Laboratory of Medical Science^

^1^Critical Care Medicine and Pulmonary Branch, National Heart, Lung and Blood Institute, National Institutes of Health, Bethesda, USA, ^2^Critical Care Medicine Department, Clinical Center, National Institutes of Health, Bethesda, USA, ^3^Zymedi, Co. Ltd., Incheon, Republic of Korea, ^4^Yonsei Institute of Pharmaceutical Sciences Research, College of Pharmacy, Yonsei University, Incheon, Republic of Korea, ^5^Department of Integrative Biotechnology, Yonsei University, Incheon, Republic of Korea, ^6^Institute for Artificial Intelligence and Biomedical Research (AIBI), Medicinal Bioconvergence Research, Center, College of Pharmacy, Yonsei University, Incheon, Republic of Korea, ^7^Department of Molecular Science and Technology, Ajou University, Suwon, Republic of Korea, ^8^Research Institute of Pharmaceutical Sciences, College of Pharmacy, Seoul National University, Seoul, Republic of Korea, ^9^Graduate School of Medical Science and Engineering, Korea Advanced Institute of Science and Technology (KAIST), Daejeon, Republic of Korea, ^10^Department of Life Science, Ewha Womans University, Seoul, Republic of Korea, ^11^Cardiovascular Center, Seoul National University Hospital, Seoul, Republic of Korea, ^12^Division of Cardiology, Department of Internal Medicine, Seoul National University College of Medicine, Seoul, Republic of Korea, ^13^College of Medicine, Gangnam Severance Hospital, Yonsei University, Seoul, Republic of Korea

Innate and adaptive immune responses play a central role in pulmonary arterial hypertension (PAH) development and progression yet are not targeted by current therapies. In pre‐clinical models, depleting or blocking the migration of pro‐inflammatory interstitial monocytes/macrophages improves lung vascular remodeling. Lysyl‐tRNA synthetase (KARS1) is normally a cytosolic enzyme that ligates lysine to tRNA for protein translation. Following phosphorylation, KARS1 translocates to the plasma membrane and enhances cancer cell migration. In this study, we examined the role of membrane‐KARS1 in pathologic migration of monocytes/macrophages and demonstrated its potential as a therapeutic target in PAH. A human single‐chain variable fragment phage display library was screened for clones binding the N‐terminal extracellular epitope of membrane‐KARS1. Lead antibodies were affinity‐optimized to generate ZMA001, a human monoclonal antibody targeting membrane‐KARS1. ZMA001 binding and effect on monocyte/macrophage migration was characterized in vitro. Membrane‐KARS1 expression was examined by immunohistochemical staining of lung tissue and flow cytometry of circulating immune cells. Therapeutic efficacy of ZMA001 was assessed in PAH animal models. ZMA001 bound human (KD = 71 pM) and rat (892 pM) N‐KARS11‐207aa with high affinity. Membrane‐KARS1 was detected in monocytes/macrophages where it promoted laminin‐mediated migration. Phorbol ester, LPS, and/or IFN‐γ increased membrane levels of KARS1 in monocytes and macrophages. ZMA001 blocked laminin‐induced migration of monoctye/macrophage cell lines where binding induced endocytosis of membrane‐KARS1. PAH patients had higher proportions of membrane‐KARS1+ lung monocytes/macrophages and blood monocytes compared to healthy controls. ZMA001 improved survival, reduced RV systolic pressure and hypertrophy, reversed vascular remodeling, and decreased pro‐inflammatory cytokines and chemokines in PAH pre‐clinical models. Membrane‐KARS1 regulates laminin‐mediated migration of inflammatory monocytes/macrophages. ZMA001 blocks monocyte/macrophage migration in vitro and in vivo, improving inflammatory vascular remodeling in PH animal models. ZMA001, targeting a unique inflammatory mechanism, may offer a new approach to PAH treatment that complements current vasodilator‐based regimens.

## A057 Characterization of Pulmonary Artery Changes in Subjects With Known or Suspected Pulmonary Hypertension by Updated Hemodynamic Criteria

Dr Tyler Couch^1^, Dr Gerald J. Beck^2^, Dr Erika Berman Rosenzweig^3^, Dr J. Jeffrey Carr^4^, Dr Serpil C. Erzurum^5^, Dr Robert P. Frantz^6^, Dr Paul M. Hassoun^7^, Dr Nicholas S. Hill^8^, Dr Evelyn M. Horn^9^, Dr Jason K. Lempel^10^, Dr Jane A. Leopold^11^, Dr Monica Mukherjee^12^, Dr Hui Nian^13^, Dr David N. Ray^1^, Dr Franz P. Rischard^14^, Dr Kevin T. Schwalbach^1^, James G. Terry^15^, Dr Anna R. Hemnes^1^

^1^Division of Allergy, Pulmonary and Critical Care Medicine, Vanderbilt University Medical Center, Nashville, USA, ^2^Department of Quantitative Health Sciences, Cleveland Clinic, Cleveland, USA, ^3^Department of Pediatrics and Medicine, New York Medical College, Westchester Medical Center, Valhalla, USA, ^4^Departments of Radiology, Biomedical Informatics, and Cardiovascular Medicine, Vanderbilt University Medical Center, Nashville, USA, ^5^Lerner Research Institute, Cleveland Clinic, Cleveland, USA, ^6^Department of Cardiovascular Medicine, Mayo Clinic, Rochester, USA, ^7^Division of Pulmonary and Critical Care Medicine, Johns Hopkins University, Baltimore, USA, ^8^Division of Pulmonary, Critical Care, and Sleep Medicine, Tufts Medical Center, Boston, USA, ^9^Perkin Heart Failure Center, Division of Cardiology, Weill Cornell Medicine, New York, USA, ^10^Imaging Institute, Cleveland Clinic, Cleveland, USA, ^11^Division of Cardiovascular Medicine, Brigham and Women's Hospital, Harvard Medical SChool, Boston, USA, ^12^Division of Cardiology, Johns Hopkins University, Baltimore, USA, ^13^Department of Biostatistics, Vanderbilt University Medical Center, Nashville, USA, ^14^Division of Pulmonary, Allergy, Critical Care and Sleep Medicine, University of Arizona, Tuscon, USA, ^15^Department of Radiology and Radiologic Sciences, Vanderbilt University Medical Center, Nashville, USA

Prior studies report association between PA size and mPAP in patients with moderate‐severe PH. It remains unclear whether these associations are present in mild PH and whether the association between PA size and mPAP differs based on PH etiology. We measured PA diameter and PA/Aorta (PA/A) ratio on chest CT for subjects with PH and matched controls in the PVDOMICS cohort. Association with mPAP for subjects undergoing right heart catheterization (RHC) was assessed using multivariable linear regression adjusting for age, sex, BMI, and time since PH diagnosis. Diagnostic performance was evaluated using receiver operating characteristic analysis. 1058 subjects underwent CT (364 WSPH group 1/comparators, 241 group 2/comparators, 259 group 3/comparators, 71 group 4/comparators, 38 group 5/comparators, 85 healthy controls), with 960 undergoing RHC (189 with mPAP ≤ 20 mmHg, 771 with mPAP > 20). PA diameter was associated with mPAP (r² = 0.29, p < 0.001). Change in mPAP between 25th and 75th percentile PA diameter was smaller for WSPH group 3 subjects (7.7 mmHg) than for other groups (8.0‐16.4 mmHg) (p < 0.001). ROC analysis of PA diameter for mPAP > 20 mmHg revealed AUC of 0.824 in allcomers and 0.901 in subjects without left heart, parenchymal lung, or thromboembolic disease. In this latter group of subjects, diameter of 30.5 mm identified by Youden's index was 88.1% sensitive and 83.3% specific for detection of mPAP > 20 mmHg. Similar associations and diagnostic performance were observed for PA/A. PA size on CT associates with mPAP and possesses good discriminatory ability for detecting PH using updated hemodynamic criteria in subjects with known or suspected PH, including in those with mild disease. The degree of change in mPAP for a given increase in PA size tends to be smaller in WSPH group 3 subjects than in other subgroups.

## A058 The Sigma‐1 Receptor Agonist Pre084 Improves Cardiopulmonary Function and Remodelling in an Experimental Model of Pulmonary Arterial Hypertension

Dr Daniel Morales‐Cano^1,2^, Marta Villegas‐Esguevillas^1,2^, Bianca Barreira^1,2^, Dr Rui Adão^1,2^, Ana Hernández‐García^1,2^, Dr María Sancho^2,3^, Dr Jorge Navarro^2,4^, Dr Jesús Ruiz‐Cabello^2,5,6^, Dr Francisco Pérez‐Vizcaíno^1,2^, Dr Belén Climent^2,4^, Dr Angel Cogolludo^1,2^

^1^Department of Pharmacology and Toxicology, School of Medicine, Complutense University of Madrid, Spain, ^2^Ciber Enfermedades Respiratorias (CIBERES), Spain, ^3^Department of Physiology, Faculty of Medicine, Complutense University of Madrid, Spain, ^4^Department of Physiology, Faculty of Pharmacy, Complutense University of Madrid, Spain, ^5^Center for Cooperative Research in Biomaterials (CIC biomaGUNE), Basque Research and Technology Alliance, Spain, ^6^6NMR and Imaging in Biomedicine Group, Department of Chemistry in Pharmaceutical Sciences, Pharmacy School, Complutense University of Madrid, Spain

Sigma‐1 receptors (S1R) are potential pharmacological targets for neurodegenerative diseases and cardiac pathologies, such as cardiac hypertrophy. S1R modulate several cellular functions by interacting with various proteins, including ion channels. Pulmonary arterial hypertension (PAH) is a severe disease characterised by exacerbated pulmonary vasoconstriction and pathological remodelling of the pulmonary vasculature, leading to right ventricular (RV) hypertrophy and eventual heart failure. Herein, we evaluated the therapeutic potential of the S1R agonist PRE084 in the hypoxia plus SU5416 (Hpx/Su) rat model of severe PAH. We found that treatment with PRE084 significantly improved the attenuated activity of Kv1.5 channels, reduced hypertrophy, and restored the resting membrane potential to physiological levels in pulmonary artery smooth muscle cells from Hpx/Su animals. This is associated with attenuated pulmonary vasoconstriction. Treatment with PRE084 also improved endothelium‐dependent relaxation and attenuated pulmonary vascular remodelling in Hpx/Su rats. Notably, PRE084 ameliorated RV systolic pressure, RV dysfunction, hypertrophy, and fibrosis in Hpx/Su rats, as assessed through haemodynamic, echocardiographic, and histological evaluations. These effects were associated with the normalization of p‐Akt expression and inositol levels in the RV. In summary, PRE084 markedly improved both the functional and morphological parameters characteristic of PAH, thereby attenuating disease progression. Collectively, our study suggests that targeting S1R may represent a promising therapeutic approach for the treatment of PAH.

## A059 Role of Nicotinic Receptors in Pulmonary Vascular Dysfunction and Pulmonary Infiltration of Immune Cells Induced by Cigarette Smoke

Ana Hernández‐Gacía^1^, Bianca Barreira^1,2^, Rosa Andreu‐Martínez^3^, Dr Francisco Pérez‐Vizcaíno^1,2^, Dr María José Calzada^3^, Dr Edgar Fernández‐Malavé^4^, Dr Angel Cogolludo^1,2^

^1^Department of Pharmacology and Toxicology, School of Medicine, University Complutense of Madrid, MADRID, Spain, ^2^Ciber Enfermedades Respiratorias (CIBERES), Spain, ^3^Department of Medicine, School of Medicine, University Autonoma of Madrid, Spain, ^4^Department of Immunology, Ophthalmology and ENT, Complutense University of Madrid, Spain

Pulmonary hypertension (PH) is a frequent complication of chronic obstructive pulmonary disease (COPD), a chronic respiratory pathology mainly associated with cigarette smoking. We have previously reported that cigarette smoke (CS) extract induces pulmonary vascular dysfunction and might contribute to PH development. In the present study, we analysed the potential role of nicotinic receptors in pulmonary dysfunction and pulmonary infiltration of inflammatory cells induced by CS. Pulmonary vascular reactivity assays were performed in isolated pulmonary arteries (PA) from C57‐BL6 Wt or alpha‐7 nicotinic receptor (α7 nAChR) KO mice. PA were incubated during 24 h with CS extract in the absence or in the presence of the nAChR antagonists (methyllycaconitine, MLA, or α‐bungarotoxin, α‐bt). In another experiments, mice untreated or treated with MLA were exposed for two weeks to air or CS. Lungs from these animals were harvested for flow cytometry analysis. We found that exposure to CS extract leads to PA endothelial dysfunction and to attenuated relaxation induced by Riociguat. These effects were prevented by MLA or α‐bt. Moreover, exposure to CS extract did not alter the relaxation to acetylcholine or Riociguat in α7‐nAChR KO mice. In animals exposed to CS, a similar impairment in acetylcholine‐ or Riociguat‐induced relaxations were observed in vehicle‐treated but not in MLA‐treated mice. No significant differences were found in lung lymphoid and myeloid subsets apart from Tγδ lymphocytes, which were significantly augmented in CS‐exposed animals. This increment was not reversed by MLA treatment. In summary, our data strongly suggests that nicotinic receptors, particularly α7‐nAChR, are involved in the pulmonary vascular dysfunction induced by CS. Furthermore, increased abundance of pulmonary Tγδ lymphocytes by CS, as previously reported for COPD patients, does not appear to involve α7‐nAChR.

## A060 Unclogging the Drain: Development and Modeling Diseases of the Pulmonary Vein

Dr. David Frank^1^, Dr. Prashant Chandrasekaran^1^, Dr. Susan Lin^2^, Dr. Mudit Gupta^1^, Dr. Sylvia Michki^1,3^, Dr. Benjamin Smood^4^, Hongbo Wen^1^, Dr. Lisa Young^3^, Dr. Maria Basil^2^, Dr. Todd Kilbaugh^5^

^1^Division of Cardiology, Department of Pediatrics, Penn Cardiovascular Institute, Penn‐CHOP Lung Biology Institute, University of Pennsylvania, The Children's Hospital of Philadelphia, Philadelphia, USA, ^2^Division of Pulmonary and Critical Care Medicine, Department of Medicine, Penn‐CHOP Lung Biology Institute, University of Pennsylvania, Philadelphia, USA, ^3^Division of Pulmonology and Sleep Medicine, Department of Pediatrics, Penn‐CHOP Lung Biology Institute, The Children's Hospital of Philadelphia, Philadelphia, USA, ^4^Division of Cardiovascular Surgery, Department of Surgery, The University of Pennsylvania, Division of Cardiothoracic Surgery, Department of Surgery, Children's Hospital of Philadelphia, Philadelphia, USA, ^5^Resuscitation Science Center, The Children's Hospital of Philadelphia, Department of Anesthesiology, Perelman School of Medicine at the University of Pennsylvania, Philadelphia, USA

Pulmonary veins deliver oxygenated blood to the left heart and, while traditionally viewed as passive conduits, possess notable regenerative capacity following injury. However, they are also susceptible to severe and often fatal diseases, including pulmonary vein stenosis (PVS) and pulmonary veno‐occlusive disease (PVOD). Despite advances in interventions, PVS still results in 30% infant mortality, and PVOD is uniformly fatal without lung transplantation, highlighting the urgent need for defining new therapeutic targets. To elucidate mechanisms and potential targets for these diseases, we employed a single‐cell RNA sequencing (scRNA‐seq) human PVOD and PVS tissue alongside age‐matched controls. For the latter, we banded the common inferior vein and allowed the neonatal swine to grow into the band, developing angiographically and hemodynamically confirmed PVS. Integration of scRNA‐seq data from PVOD and age‐matched controls and human PVS and swine PVS were performed. Key findings were confirmed using immunohistochemistry (IHC) or RNA fluorescent in situ hybridization (RNA‐FISH). Single‐cell transcriptomic analysis of PVOD revealed disease‐specific venous and capillary endothelial cell (EC) populations absent in controls. Notably, we observed dysregulation of the integrated stress response (ISR) and its key transcription factor, ATF4. Several vascular signaling components with existing therapies were upregulated and confirmed by IHC, including endothelin (EDN1), a potent vasoconstrictor, and BRD4, a pro‐mitogenic epigenetic regulator. BMP6, a ligand for both BMPR2 and ACVR2, was significantly elevated, correlating with increased phospho‐SMAD2 and expression of the TGF‐β target gene, SERPINE1 (PAI1). In PVS, we identified a distinct COL15A1+ venous EC population present only in diseased tissue. Interestingly, we also detected activation of multiple targetable pathways, including aberrant expression of arginine vasopressin (AVP), a vasoconstrictive peptide typically restricted to the brain.

In summary, our findings define novel, targetable mechanisms underlying pulmonary vein disease and introduce a translational large animal model that enables further mechanistic studies and therapeutic development.

## A061 PAH Patient Perspectives on AI Survival Assessments: Current Practices, Barriers, and Opportunities

Arpit Mathur^1^, Katelyn Morrison^1^, Udhirna Krishnamurthy^1^, Jonathan Lai^1^, Sang Wan Lee^2^, Priscilla Correa‐Jaque^3^, Dr. Shili Lin^2^, Dr. Sandeep Sahay^4^, Dr. Megan Griffiths^5^, Dr. Manreet Kanwar^6^, Dr. Allen Everett^7^, Dr. Raymond Benza^3^, Dr. Adam Perer^1^

^1^Carnegie Mellon University, Pittsburgh, USA, ^2^Ohio State University, Columbus, USA, ^3^Sentara College of Health Sciences, Chesapeake, USA, ^4^Houston Methodist, Houston, USA, ^5^UT Southwestern Medical Center, Dallas, USA, ^6^University of Chicago, Chicago, USA, ^7^Johns Hopkins University, Baltimore, USA

AI‐based survival assessments are increasingly used to inform clinical decision‐making in Pulmonary Arterial Hypertension (PAH). While clinician‐facing tools show promise for triage and treatment planning, little is known about how patients themselves interpret and would use survival risk information within shared decision‐making (SDM). To understand (1) how people living with PAH interpret AI‐based survival assessments and (2) how patient‐facing survival information could support care management, motivation, and treatment discussions. We conducted semi‐structured interviews with 18 participants: 8 PAH support‐group leaders, 8 patients, and 2 caregivers. Sessions were grounded with a design probe: an interactive web dashboard (PHORA) that visualizes longitudinal clinical data, survival risk scores, and counterfactual “what‐if” scenarios. Interviews were recorded, transcribed, and analyzed via multi‐coder thematic analysis with iterative affinity diagramming. Participants reported substantial information needs outside clinic visits and described barriers to SDM, including medical jargon, time constraints, and difficulty interpreting medical data. Most participants had not seen formal survival scores, but relied on proxies (e.g., 6‐minute walk distance, WHO functional class). When interacting with the probe, participants articulated three high‐value uses for patient‐facing survival assessments: (i) contextualizing clinical numbers to personally meaningful ranges; (ii) tracking temporal trends to recognize improvement or deterioration; and (iii) analyzing treatments by relating medication changes to survival trajectories. Survival information carried psychological weight, suggesting the need for careful framing. Patients want transparent, comprehensible survival assessments to participate more confidently in SDM and to sustain motivation for medication adherence, diet, and exercise. We contribute the first in‐depth qualitative account of PAH patient perspectives on survival risk outputs and offer design directions for patient‐facing decision support. Patient‐centered survival dashboards may complement clinician tools to strengthen SDM and ongoing self‐management in PAH.

## A062 Profound Loss of Regenerative Lung Microvascular Endothelial Cells in a Rodent Model of Pulmonary Veno‐Occlusive Disease: Role of Endothelial‐To‐Mesenchymal Transition?

Ms. Elham Salehisiavashani^1,2^, Dr. Yupu Deng^1^, Dr. Pramod Sahadevan^1^, Ms. Elmira Safaie Qamsari^1,2^, Dr. Nicholas D. Cober^1,2^, Dr. Jalil Azami^1^, Dr. Kenny Schlosser^1^, Dr. Duncan J. Stewart^1,2,3^

^1^Ottawa Hospital Research Institute, Ottawa, Canada, ^2^Faculty of Medicine, University of Ottawa, Ottawa, Canada, ^3^University of Ottawa, Heart Institute, Ottawa, Canada

Pulmonary veno‐occlusive disease (PVOD) is a rare and fatal subset of pulmonary arterial hypertension (PAH), characterized by occlusive remodeling in all three vascular compartments: venous, capillary, and arterial. While PVOD can be sporadic or caused by EIF2AK4 mutations, it is more commonly associated with exposure to alkylating agents, in particular mitomycin C (MMC). PVOD has a poor survival and lacks any effective therapy apart from lung transplantation. We performed single‐cell RNA‐seq (scRNAseq) using dissociated cells from lungs from MMC‐treated rats to explore the cell and molecular mechanisms of PVOD. A rat PVOD model was established using two daily injections of MMC (2.5 mg, IP). After five weeks, we assessed right ventricular (RV) systolic pressure (RVSP) and hypertrophy (Fulton index), vascular remodeling (H&E, Mason trichrome), and immunofluorescent (IF) staining for endothelial cells (ECs, CD31) and smooth muscle cells (SMC)/myofibroblasts (αSMC). MMC‐treated rats exhibited marked elevation in RVSP and Fulton Index, with severe arterial, venous, and alveolar capillary remodeling. Flow cytometry showed an 8‐fold reduction in the proportion of lung ECs, which was confirmed by scRNAseq, demonstrating with near total loss of regenerative ‘general’ capillary (gCap) ECs, which harbor the endothelial stem/progenitor cells, while the proportion of structural alveolar (aCap ECs or aerocytes), that form the air‐blood barrier, was preserved. RNA velocity and pseudo‐time analyses identified vectors that originated from an ‘intermediate’ cell cluster, exhibiting both endothelial and stromal cell features, converging on an SMC/myofibroblast cluster. IF staining confirmed widespread loss of ECs with the accumulation of myofibroblast‐like cells within remodeled veins, arteries, and capillaries, consistent with endothelial‐to‐mesenchymal transition (EndMT). These findings suggest that EndMT may be a central mechanism underlying lung microvascular EC loss, generating myofibroblast‐like cells that further contribute to occlusive vascular remodeling. This work points to EndMT as a potential therapeutic strategy for the treatment of PVOD.

## A063 Effect of E‐Cigarette Aerosol on Pulmonary Endothelial Barrier Function in Isolated Mouse Lungs

Dr Oleg Pak^1^, David Spiegelberg^1^, Thorben Pape^2^, Karin Quanz^1^, Janik Deutscher^1^, Prof. Hossein A. Ghofrani^1^, Prof. Friedrich Grimminger^1^, Prof. Werner Seeger^1^, Prof. Ralph Theo Schermuly^1^, Benjamin Seeliger^2^, Prof. Norbert Weissmann^1^, Natascha Sommer^1^

^1^Excellence Cluster Cardio‐Pulmonary Institute, University of Giessen and Marburg Lung Center (UGMLC), member of the German Center for Lung Research (DZL), Institute for Lung Health (ILH). Justus‐Liebig‐University, Giessen, Germany, ^2^Division of Respiratory Medicine and Infectious Diseases, Hannover Medical School and German Center for lung research, Hannover, Germany

Although marketed as a safer alternative to traditional cigarettes, the toxic effects of specific e‐cigarette components, particularly nicotine and flavorings, and effect of inflammatory co‐stimuli remain poorly understood. We investigated whether short‐term exposure to e‐cigarette vapor (ECV) containing different ingredients induces endothelial barrier dysfunction in the pulmonary vasculature. Isolated perfused and ventilated mouse lungs were exposed intratracheally to ECV for five minutes every 60 minutes over a three‐hour period. ECV was generated from three liquids: the base solution (55% propylene glycol, 35% glycerin, 10% water), the base with nicotine (18 mg/ml), and the base with ‘cherry’ flavor, using a Joyetech eVic Primo Mini (0.5 Ω, 30 W). Endothelial permeability was assessed by measuring capillary filtration coefficient (Kfc, lung weight gain after a hydrostatic maneuver), fluid retention, and wet‐to‐dry weight ratio. The effect of the Tie2 agonist PMC403 on capillary leakage was also evaluated. Exposure to nicotine‐ or flavor‐containing ECV significantly increased Kfc compared to control lungs ventilated with normoxic gas or base ECV alone. Nicotine‐ and flavor‐containing ECV also caused significant fluid retention, whereas the base solution did not. The wet‐to‐dry weight ratio showed a trend towards an increase in lungs exposed to nicotine‐containing ECV. Perfusion with LPS (6 µg/ml) also increased endothelial permeability, edema formation, and wet‐to‐dry ratio, but did not further enhance the effects of ECV inhalation. Treatment with the Tie2 agonist PMC403 (10 µg/ml) effectively prevented ECV‐ and LPS‐induced barrier disruption. Repetitive inhalation of nicotine‐ or flavor‐containing ECV, but not the base solution, significantly increases pulmonary endothelial permeability and fluid retention in mouse lungs. Tie2 activation with PMC403 mitigates this barrier dysfunction and may represent a potential protective strategy.

## A064 Long‐Term Vascular Sequelae of COVID‐19: Persistent Endothelial Damage and Cardiovascular Risk 4‐years Post‐Infection

Postdoctoral Researcher Noelia Prieto‐Martínez^1^, Reseach Technician and Lecturer Anna Vert Company^1,2^, Reseach Technician and Project manager Neus Luque^1^, PhD student Sanna Ayyoub^1^, Physician and Associate Professor Saioa Eizaguirre^1,3^, Physician and Associate Professor Marc Bonnin^1,3^, Principal Investigator/Group Leader and Substitute Lecturer Olga Tura‐Ceide^1,3,4,5^

^1^Department of Pulmonary Medicine, Dr. Josep Trueta University Hospital de Girona, Santa Caterina Hospital de Salt and the Girona Biomedical Research Institute (IDIBGI), Girona (17190), Spain, ^2^Department of Biology, Faculty of Sciences, University of Girona, C/Maria Aurèlia Campany 69, Campus Montilivi, Girona (17003), Spain, ^3^Department of Medical Sciences, Faculty of Medicine, University of Girona, Girona, Spain, ^4^Biomedical Research Networking Centre on Respiratory Diseases (CIBERES), Madrid, Spain, ^5^Department of Pulmonary Medicine, Servei de Pneumologia, Hospital Clínic‐Institut d'Investigacions Biomèdiques August Pi I Sunyer (IDIBAPS), University of Barcelona, Villaroel, 170, Barcelona, Spain

The highly transmissible SARS‐CoV‐2 has caused a global pandemic with major mortality and economic impact, damaging the endothelium and causing cardiovascular complications. Endothelial colony‐forming cells (ECFCs) are released into the blood to aid vascular repair. Due to their high angiogenic capacity, ECFC number and function closely reflect endothelial health and serve as biomarkers of vascular injury and long‐term sequelae. This study aimed to longitudinally assess ECFC counts and determine whether the significant increase observed up to 12‐months post‐SARS‐CoV‐2 infection in our previous studies persists after 4‐years. 29 post‐COVID‐19 patients were recruited at 3, 6, and 12‐months, as well as 4‐years after infection, along with 31 healthy controls matched for age, sex, BMI, smoking history, and comorbidities. ECFCs were isolated from peripheral blood mononuclear cells (PBMCs) in both patients and controls and cultured under specific conditions until colonies emerged. Patients initially recruited during 2020–2021 showed a significant increase in ECFCs at 3‐months (82.8% vs. 48.4%; P < 0.05), 6‐months (87% vs. 48.4%; P < 0.05), and 12‐months (85% vs. 48.4%; P < 0.05) post‐SARS‐CoV‐2 infection. Follow‐up of the same cohort in 2024‐2025, 4‐years after infection, confirmed a persistent abnormal increase in ECFC production, observed in 88.2% of post‐COVID‐19 patients vs 48.4% of healthy controls (P < 0.05). The average number of colonies was also significantly higher in patients than controls at 3‐months (2.79 ± 2.41 vs. 1.23 ± 1.86; P < 0.05), 6‐months (2.78 ± 2.0 vs. 1.23 ± 1.86; P < 0.05), 12‐months (2.9 ± 2.61 vs. 1.23 ± 1.86; P < 0.05), and remained elevated at 4‐years (2.48 ± 1.78 vs. 1.23 ± 1.86; P < 0.05), indicating ongoing endothelial damage. Furthermore, troponin levels remained significantly elevated up to 4‐years post‐infection (P < 0.01), in line with our previous findings from 3 to 12‐months. These findings reveal persistent endothelial dysfunction and elevated cardiovascular risk up to 4‐years post‐infection, underscoring the need for long‐term monitoring and prevention.

## A065 Quantification of Right Ventricular Remodelling in Pulmonary Hypertension Using 3D Echocardiography

Mr Jop Schneijdenberg^1^, Dr. Annemien van den Bosch^1^, Dr. Daniel Bowen^1^, Bernardo Raposo Loff Barreto^1^, Gerard van Burken^1^, Dr. Johan Bosch^1^, Dr. Karin Boomars^2^

^1^Erasmus MC, Cardiovascular Institute, Thorax Center, Department of Cardiology, Rotterdam, The Netherlands, ^2^Erasmus MC, Cardiovascular Institute, Thorax Center, Department of Pulmonary Medicine, Rotterdam, The Netherlands

In pre‐capillary pulmonary hypertension (PH), increased pulmonary vascular resistance leads to a compensatory rise in pulmonary arterial pressure. Consequently, the right ventricle (RV) undergoes structural and functional remodelling, which could lead to fatal RV failure. This study aims to investigate the potential of 3D echocardiography (3DE) to gain insight in RV remodelling in patients with pre‐capillary PH. RV‐focused 3DE images were obtained from 18 pre‐capillary PH patients and 50 healthy controls, and converted to dynamic 3D meshes (4D RV‐Function 3, TomTec, Unterschleissheim). A dedicated method was developed in a custom software application (RV‐Dynamics) to decompose RV contraction in longitudinal, radial, and anteroposterior motion directions, and a novel approach was implemented to quantify regional endocardial curvature. These analyses allowed for detailed assessment of both functional and morphological RV remodelling.

Functional analysis revealed that radial contraction was most impaired in PH patients, showing significant reduction compared to healthy controls (P < 0.001). In contrast, longitudinal contraction exhibited no significant difference between both groups. Curvature was reduced the most in the inferior free wall in PH patients compared to controls. Additionally, significant end‐systolic septal flattening was quantified (P < 0.001). 3DE provides unique value in quantifying functional and morphological RV remodelling in pre‐capillary PH patients. Whereas radial contraction was evidently reduced in PH patients, longitudinal contraction was preserved, underlining the importance of multidirectional functional assessment. Morphological alterations were most evident in the inferior free wall and septum, emphasising the need for regional quantification. Together, these findings demonstrate the unique value of 3DE for detailed characterization of RV remodelling in PH, which may contribute to improved phenotyping and risk stratification.

## A066 Superoxide‐Driven Peroxynitrite Formation Mediates S‐Nitrosylation of Adenylyl Cyclase Isoform 6: Linking Hypoxia to Pulmonary Vasoconstriction

Phd Candidate Saeid Maghsoudi^1^, Mr Musstafa Smeir^1^, Ms Gabriela Todescan^1^, Mr Vikram Bhatia^1^, Mr Ahmed Al‐Khaffaf^1^, Ms Martha Hinton^1^, Mrs Shyamala Dakshinamurti^1^

^1^University of Manitoba, Winnipeg, Canada

Persistent pulmonary hypertension of the newborn (PPHN), affecting 1–6/1000 births, is a life‐threatening failure of pulmonary arterial relaxation characterized by sustained constriction of pulmonary arterial smooth muscle cells (PASMCs). A central mediator of vasodilation, the adenylyl cyclase (AC)–cAMP pathway, is disrupted in hypoxia. Neonatal pulmonary arteries express mainly AC isoform 6, plus ACs 3, 7 and 9. We reported that in hypoxia, AC6 is selectively inhibited by s‐nitrosylation of cysteine 1004 within its catalytic C2 subunit, a residue critical for Gαs interaction and ATP turnover. This modification blocks AC6 activation and cAMP generation, promoting vasoconstriction. We hypothesized that reactive nitroso species (sNO) generated from nitric oxide and superoxide drive AC6 s‐nitrosylation during hypoxia, and disrupt catalytic subunit (C1–C2) interaction. First passage PASMC from newborn piglets (< 24 h old), and PASMC primary cultured from newborn piglets raised in 10% O2 for 72 h (PPHN) or from age‐matched normoxic controls, were cultured to confluence then exposed to normoxia (21% O₂) or hypoxia (10% O₂) for 72 h with or without nitroso donor GSNO, superoxide dismutase (SOD) or peroxynitrite scavenger FeTTPs. Superoxide measured by dihydroethidium (DHE), NO/sNO by diaminofluorescein (DAF), quantified by FACS; protein s‐nitrosylation by biotin‐switch labeling; AC activity measured by Tb (III)‐norfloxacin assay; cAMP generation by HTRF‐Gαs kit. Hypoxia increased intracellular superoxide and protein s‐nitrosylation. AC6, eNOS and Gαs, but not Gαi, were s‐nitrosylated by GSNO, or by hypoxia in vivo or in vitro. sNO generation was superoxide‐dependent, attenuated by SOD or FeTTPs. AC activity and cAMP generation were attenuated by hypoxia or by sNO donors; and restored by scavenging superoxide or peroxynitrite. Hypoxia enhances pulmonary arterial superoxide production, which scavenges nitric oxide to form peroxynitrite, s‐nitrosylating AC6; this disrupts catalytic subunit interactions and suppresses cAMP synthesis. AC6 s‐nitrosylation represents a novel target for drug discovery.

## A067 Atrial Septostomy With AFR Device as a Bridge to Transplantation in a Patient With Complex Congenital Heart Disease and Combined Pulmonary Hypertension

Dr. Juan Duarte‐Torres^1,2^, Dr. Pablo Meras‐Colunga^2^, Dr. Jose Ruiz‐Cantador^2^, Dr. Carlos Merino‐Argos^2^, Dr. Ines Ponz‐de Antonio^2^, Dr. Adriana Gonzalez‐Chaverri^2^, Dr. Luz Polo‐Lopez^2^, Dr. Angel Aroca‐Peinado^2^, Dr. Santiago Jimenez‐Valero^2^, Dr. Enrique Balbacid‐Domingo^2^, Dr. Raul Moreno‐Gomez^2^

^1^Hospital Universitario 12 de Octubre, Madrid, Spain, ^2^Hospital Universitario La Paz, Madrid, Spain

Combined post‐ and precapillary pulmonary hypertension (CpcPH) associated with congenital left heart disease and diastolic dysfunction has limited therapeutic options. We present a case of percutaneous atrial septostomy with an atrial flow regulator (AFR) as a bridge to transplantation in a patient with complex congenital heart disease. A 39‐year‐old man with a history of neonatal aortic coarctation repair and bicuspid aortic valve valvuloplasty presented with progressive heart failure and severe CpcPH. At diagnosis (2009), right heart catheterization showed mPAP 55 mmHg, LVEDP 45 mmHg, and PVR 4 WU. He also had paroxysmal atrial fibrillation and was treated with anticoagulation, sildenafil monotherapy, and diuretics. Due to progressive NYHA class III–IV symptoms and limited therapeutic options, percutaneous atrial septostomy with implantation of an 8 mm AFR device was performed in 2020 to decompress left‐sided filling pressures (mPAP 40 mmHg, LVEDP 24 mmHg, PVR 4.5 WU). A continuous left‐to‐right shunt was established. Pre‐procedural NT‐proBNP was 1053 pg/mL. The patient experienced significant clinical and hemodynamic improvement, oscillating between NYHA class II and III for three years following the procedure. NT‐proBNP decreased to approximately 326 pg/mL during the first year after septostomy. He remained free from hospitalizations and with good outpatient stability for 2.5 years, until recurrent atrial fibrillation triggered decompensated heart failure. Ultimately, progressive deterioration led to listing and successful heart transplantation in 2023. Percutaneous atrial septostomy with AFR can provide meaningful and sustained symptomatic benefit, reduce filling pressures, and improve biomarkers as a palliative or bridge therapy in patients with CpcPH secondary to left heart disease, even in the setting of complex congenital cardiac anatomy.

## A068 Management of an ASD With Pulmonary Hypertension and Exertional Desaturation: A Treat‐and‐Repair Case

Dr. Juan Duarte‐Torres^1^, Dr. Teresa Segura‐de la Cal^1^, Dr. Fernando Sarnago‐Cebada^1^, Dr. Irene Martín‐de Miguel^1^, Dr. Alejandro Cruz‐Utrilla^1^, Dr. Eva Gutiérrez‐Ortiz^1^, Dr. Berenice Brown‐Arriola^1^, Dr. Pilar Escribano‐Subías^1^

^1^Hospital Universitario 12 De Octubre, Madrid, Spain

In patients with congenital heart disease–associated pulmonary arterial hypertension (PAH), the “treat‐and‐repair” approach — targeted therapy followed by defect closure — is a challenging but feasible option in carefully selected cases. We present a patient with severe PAH and exercise‐induced desaturation who underwent successful fenestrated ASD closure guided by advanced functional testing. A 23‐year‐old woman presented with severe PAH due to an ostium secundum atrial septal defect (ASD) and partial anomalous pulmonary venous drainage (PAPVD, right upper pulmonary vein to SVC). Baseline findings included erythrocytosis, resting oxygen saturation 91% (79% with exercise), and severe ventilatory inefficiency. Right heart catheterization (RHC) showed mean PAP 41 mmHg, PCWP 3 mmHg, RAP 2 mmHg, Qp/Qs 1.83, and PVR 6.5 WU. Cardiac MRI revealed a severely dilated RV with an RVEF of 40% and a 42 mm pulmonary artery aneurysm. NT‐proBNP was 369 pg/mL. Subjectively, the patient was in NYHA functional class III. After targeted PAH therapy (tadalafil and ambrisentan), the patient improved clinically (NYHA I, SaO₂ 99%), with NT‐proBNP 249 pg/mL. Repeat RHC showed mean PAP 32 mmHg, PCWP 8 mmHg, Qp/Qs 2.45, and PVR 2.34 WU. Despite improved peak VO₂ and ventilatory efficiency, exertional desaturation persisted (~80%). Advanced functional testing, including exercise RHC and stress echocardiography, revealed no pathological rise in PVR, preserved RV contractile reserve, and only mild transient shunt reversal.

Given these findings, surgical fenestrated ASD closure was performed, while PAPVD was left unrepaired due to minimal impact. At 6‐month follow‐up, the patient was asymptomatic and active, with improved RV dimensions, resolution of tricuspid regurgitation, NT‐proBNP 123 pg/mL, and normalized ECG axis. This case illustrates the value of treat‐and‐repair with fenestrated closure, guided by advanced functional assessment, in a patient with PAH and persistent exertional desaturation after targeted therapy.

## A069 SOX17 Drives Resilience of the Pulmonary Arterial Endothelium Under Shear Stress

Msc Beau Neep^1^, Msc Laura E. De Heus^2^, Msc Lion Raaz^3^, Msc Xiaoke Pan^1^, PhD Robert Szulcek^4^, PhD Petra Knaus^3^, PhD Marie Jose Goumans^6^, PhD Christopher Rhodes^5^, MD PhD Martin R Wilkins^5^, MD PhD Harm Jan Bogaard^1^, MD PhD Jurjan Aman^1^

^1^Dept. of Pulmonary Medicine, Amsterdam UMC, Amsterdam, The Netherlands, ^2^Applied Stem Cell Technologies, Twente University, The Netherlands, ^3^Institute of Chemistry and Biochemistry, Signal Transduction lab, Freie Universität Berlin, Germany, ^4^Freie Universitat Berlin, Berlin, Germany, ^5^Imperial College London, National Heart and Lung Institute, London, United Kingdom, ^6^Cardiovascular Cell Biology, Leids Universitair Medisch Centrum (LUMC), Leiden, The Netherlands

Pulmonary arterial hypertension (PAH) is a fatal lung disease characterized by pulmonary vascular remodeling. Genetic variations in SOX17 have been associated with PAH development. Although SOX17 is a known embryonic transcription factor involved in arterial differentiation, its function in the adult vasculature is unclear. Given its arterial expression pattern, we hypothesized that SOX17 drives adaptation to high flow and that impaired SOX17 expression predisposes to vascular injury.

SOX17 expression was higher in healthy pulmonary artery endothelial cells (PAECs) as compared to microvascular endothelial cells. Exposure of PAEC to physiological fluid shear stress (15 dyn/cm2 up to 72 h) enhanced expression of SOX17, but not related SoxF members SOX7 or SOX18. SOX17 depletion using short hairpin RNA (shRNA) reduced elongation in the direction of flow, indicating disturbed shear adaptation. SOX17 depletion induced intercellular gap formation under static conditions, which was significantly exaggerated upon exposure to physiological shear stress. Regenerative capacity was disturbed, indicated by reduction in Ki67‐positive cells in wounding assays and decreased expression of CyclinE1. In line with the impaired barrier, SOX17 depletion reduced expression levels of the junction proteins VE‐cadherin and β‐catenin, and disturbed organization of these proteins in adherens junctions. This was reproduced in a 3D vessel‐on‐a‐chip. Cut&Run analysis performed after shear stress revealed that SOX17 directly binds to the promotor of mentioned junction proteins. RNAseq following deadCRISPR modulation of the SOX17 promotor (activation/repression), revealed the guidance cue Semaphorin 3 G (SEMA3G) as downstream target, with direct binding of SOX17 binding to its promotor (Cut&Run). Treatment of PAEC with recombinant SEMA3G restored VE‐cadherin at adherens junctions, and rescued barrier disruption following SOX17 depletion. SOX17 is crucial for the maintenance of endothelial barrier integrity under fluid shear stress. SOX17 drives endothelial maintenance via parallel genetic programs. Impaired SOX17 expression sensitizes the arterial endothelium, contributing to pulmonary vascular remodeling.

## A070 Discovery of an Immunothrombotic Signature in the Pulmonary Endothelium of CTEPH Patients

Dr. Wouter Venema^1,2^, Dr. Giovanni Maroli^3,4^, Prof. Dr. Soni Savai Pullamsetti^3,4^, Ms. Xiaoke Pan^1,2^, Prof. Dr. Harm Jan Bogaard^1,2^, Dr. Jurjan Aman^1,2^

^1^Department of Pulmonary Medicine, Amsterdam University Medical Centre, Amsterdam, The Netherlands, ^2^Amsterdam Cardiovascular Sciences, Pulmonary Hypertension and Thrombosis, Amsterdam, The Netherlands, ^3^Max‐Planck Institute for Heart and Lung Research, Bad Nauheim, Germany, ^4^Department of Internal Medicine, Justus Liebig University, Giessen, Germany

Chronic thromboembolic pulmonary hypertension (CTEPH) is a severe and increasingly recognized form of pulmonary arterial hypertension (PAH). CTEPH patients suffer from persistent thrombi formation and incomplete resolution in the pulmonary arteries or distal microvasculature. A causal molecular pathway or genetic predisposition is not known, therefore we aimed to identify new leads in the development of CTEPH. We combined RNA sequencing and H3K27Ac ChIP assay in pulmonary artery endothelial cells (PAECs) from CTEPH patients compared to control PAECs to identify genes that are differentially expressed in the CTEPH endothelium. After integrating both datasets, pathway enrichment analysis revealed a common signature that is functionally linked to immunothrombosis. This signature included an upregulated inflammatory trait related to leukocyte recruitment (CXCL2), increased complement activation (C1R/C1S, C7) and disturbed thrombolysis (SERPINE1). The link between inflammation and the complement system in CTEPH was illustrated by the upregulation of C1QTNF1 (also known as CTRP1). C1QTNF1 is associated with hypertension and cardiac disease, but also the regulation of platelet aggregation. Furthermore, TNFSF10, also known as Tumor Necrosis Factor‐related Apoptosis‐inducing Ligand (TRAIL), was increased in CTEPH. These observations point towards an integrated molecular network that links inflammation and the complement system to the thrombosis in CTEPH. Unraveling how these persistent thrombi are formed is essential for understanding the disease mechanism and the possibility to find novel pharmacological therapies in CTEPH.

## A071 Evaluating the Effect of Increasing Dose of Sildenafil on Pulmonary Haemodynamics in ILD‐PH Using Remote Monitoring

Dr Rebecca Burney^1,2^, Dr Marcelle Paula‐Ribeiro^2^, Dr Ze Ming Goh^1,2^, Dr Sophie Herbert^3,4^, Dr Ee Ling Heng^3,4^, Professor S John Wort^3,4^, Dr Colm McCabe^3,4^, Dr Heba Nashat^3,4^, Professor David Kiely^1,2^, Dr Roger Thompson^1,2^, Professor Alex Rothman^1,2^, Dr Laura C Price^3,4,5^, Professor Robin Condliffe^3,4,5^

^1^Department of Clinical Medicine, University Of Sheffield, Sheffield, UK, ^2^Pulmonary Vascular Disease Unit, Royal Hallamshire Hospital, Sheffield, UK, ^3^National Pulmonary Hypertension Service, Royal Brompton Hospital, London, UK, ^4^National Heart and Lung Institute, Imperial College London, London, UK, ^5^Both authors contributed equally

Pulmonary hypertension can complicate interstitial lung disease (ILD‐PH) and is associated with reduced quality‐of‐life and increased mortality. International guidelines recommend consideration of phosphodiesterase‐5 inhibitor (PDE5i) therapy. Prior studies, in heterogeneous populations, have not demonstrated consistent improvements in exercise capacity. However, benefits may exist in subgroups identified by detailed haemodynamic evaluation. We monitored remote cardiopulmonary haemodynamics and physical activity in treatment‐naïve patients with fibrotic ILD‐PH initiated on sildenafil. Diuretics and oxygen therapy were optimised at baseline. Pulmonary artery pressure monitors (cardioMEMS, Abbott) and insertable cardiac monitors (LinQ, Medtronic) were implanted as part of the FIT‐PH study (FIT‐PH/IRAS269248/STH20422). 6MWT, cardiac MRI/echocardiography, PFT's and NT‐proBNP were performed at baseline and on maximum‐tolerated sildenafil dose.

Following implantation, a sildenafil test dose was administered with pulmonary artery pressure and heart rate measured every 20 minutes over two hours. Thereafter, patients underwent a two‐week monitoring phase before initiating sildenafil 25 mg TDS if not hypotensive. This was titrated monthly as tolerated, up to 100 mg TDS. Pulmonary arterial pressure and flow, heart rate and physical activity were downloaded daily. Systemic blood pressure and oxygen saturations and requirement were also recorded. To date, six participants have been enrolled across two sites with a mean age of 66 ± 3.4 years (SD), male:female ratio 2:1, mPAP 40 ± 9 mmHg, PVR 6.7 ± 2 WU, RAP 7.2 ± 4.6 mmHg, PAWP 8.8 ± 4.9 mmHg, NTproBNP 1278.5 ± 1234.4 ng/L, DLco 22.3 ± 4%, and 6MWD 115 m ± 112 m. 3 participants were on long term oxygen therapy. These cases represent the first cardioMEMS implants in patients with ILD‐PH, with no device‐related complications. Early results have consistently demonstrated improved pulmonary haemodynamics in response to the sildenafil test dose. Longer‐term response to increasing doses of sildenafil will be presented. This study aims to provide new insights into the role of remote monitoring as a study endpoint, and of PDE5i therapy in carefully selected individuals with ILD‐PH.

## A072 VEGFR2 is Essential for Microvascular Maintenance in the Adult Lung

Dr Xiao‐Qing Sun^1^, Wen‐Jun He^1^, Bayan Akhras^1^, Xiaoke Pan^1^, Yosuke Wada^2^, Norbert Weissmann^3^, Anton Vonk‐Noordergraaf^1^, Harm‐Jan Bogaard^1^, Jurjan Aman^1^

^1^Department of Pulmonary Medicine, Amsterdam UMC, VU University Medical Center, Amsterdam Cardiovascular Sciences (ACS), Amsterdam, The Netherlands, ^2^First Department of Internal Medicine, Shinshu University School of Medicine, Japan, ^3^University of Giessen and Marburg Lung Center (UGMLC), Justus‐Liebig University Giessen and Member of the German Center for Lung Research (DZL), Giessen, Germany

Increasing evidence points toward a role of microvascular loss in development of chronic lung diseases, highlighting the dependence of parenchymal integrity and lung function on vascular integrity. Vascular endothelial growth factor receptor 2 (VEGFR2, KDR) is essential for vascular development and integrity, yet its role in maintaining the adult lung remains unclear. This study investigates the role of VEGFR2 in lung vascular maintenance in health and disease (emphysema). We hypothesized that VEGFR2‐mediated vascular integrity is a key determinant of lung health. VEGFR2 expression and activation were assessed in lung tissue and lung microvascular endothelial cells (MVECs) from emphysema patients versus matched controls, and in a cigarette smoke mouse model. Endothelial‐specific VEGFR2 knockout mice were exposed to hypoxia for 4 weeks, followed by evaluation of vascular/alveolar integrity and emphysema progression at 3 and 9 months using echocardiography and histological analyses. In a meta‐analysis of 11 scRNAseq database, KDR was specifically expressed in the lung microvascular endothelium (aCaps), and was suppressed by risk factors like ageing and smoking. In line with this, VEGFR2 expression and activation were markedly reduced in lung MVECs isolated from emphysema patient lungs, and in mouse lung tissues after chronic smoke exposure. In human lung MVECs, VEGFR2 was activated by shear stress, while VEGFR2 deletion induced inter‐endothelial gaps and impaired barrier integrity under shear stress. In vivo, endothelial VEGFR2 knockout mice exhibited persistently elevated right ventricular systolic pressure (RVSP) with RV hypertrophy after hypoxia, whereas control mice recovered to normal within 3 months. In the adult lung, VEGFR2 is critical for microvascular integrity. Ongoing studies will evaluate whether microvascular loss contributes to injury to the lung parenchyma. Targeting vascular repair via VEGFR2 signalling may offer a novel therapeutic approach for chronic lung diseases like emphysema.

## A073 Micropeptide‐Encoding lncRNAs LNC16 and LNC19 Regulate Translation Pathways Under Hypoxia in the Lungs

Miss Zahraa S. Msheik^1,2^, Dr. Chanil Valasarajan^1,2^, Dr. Fatemeh Khassafi^1,2^, Dr. Anoop V. Cherian^1,2^, Prof. Dr. Marek Bartkuhn^1,3^, Prof. Dr. Rajkumar Savai^1,2^, Prof. Dr. Werner Seeger^1,2^, Prof. Dr. Soni Savai Pullamsetti^1,2^

^1^Department of Internal Medicine, Universities of Giessen and Marburg Lung Center (UGMLC), Institute for Lung Health (ILH), Cardio‐Pulmonary Institute (CPI), Member of the German Center for Lung Research (DZL), Gießen, Germany, ^2^Department of Lung Development and Remodelling, Max Planck Institute for Heart and Lung Research, Bad Nauheim, Germany, ^3^Biomedical Informatics and Systems Medicine, Justus Liebig University, Gießen, Germany

Hypoxia, a condition characterized by insufficient oxygen levels in the tissues, plays a crucial role in various pathological events in the lungs. Recent studies have shown that in addition to coding genes, long non‐coding RNAs (lncRNAs) are dysregulated under hypoxia conditions. lncRNAs can regulate several signaling pathways by acting as RNA‐decoys, miRNA sponges, and splicing modulators. Notably, recent studies have shown that some of these lncRNAs can be further translated into micro‐peptides, opening up novel therapeutic options. In this project, we aim to delineate the role of two lncRNAs with high coding potential (LNC16 and LNC19) in the modulation of the hypoxia‐driven signaling pathways and their contribution to the pathogenesis of chronic lung diseases. Screening for dysregulated lncRNAs after 24hrs of hypoxia (hox) exposure in human pulmonary arterial smooth muscle cells (hPASMCs) identified several candidates. Among these, LNC16 and LNC19, selected from the 10 most significantly upregulated lncRNAs, exhibited notable coding potential, suggesting their possible translation into micro‐peptides. This was further confirmed by generating antibodies that specifically recognized the endogenous peptides derived from LNC16 and LNC19. Screening the selected lncRNAs in different lung diseases revealed that LNC16 was significantly upregulated in COPD, while LNC19 was significantly upregulated in both COPD and IPF. Further investigation of the cellular localization of LNC16 and LNC19 revealed that LNC16 is distributed equally between nuclear and cytoplasmic compartments, while LNC19 is predominantly expressed in the nucleus. Furthermore, silencing LNC16 induced significant apoptotic resistance in the hox‐exposed hPASMCs, whereas LNC19 knockdown led to a significant anti‐proliferative phenotype. Transcriptomic analysis revealed that silencing LNC16 and LNC19 dysregulated pathways associated with protein translation in the hox‐exposed hPASMCs. Specifically, their knockdown disrupted a key component of the cap‐dependent translation, eIF2α. These findings indicate a potential role for LNC16 and LNC19 in regulating translation under hypoxic conditions in the lungs.

## A074 Wnt5a‐mediated Crosstalk Between Adipocytes and Cardiomyocytes Regulates Right Ventricular Function in Group 2 PH

Assistant Professor Vineet Agrawal^1^, Zoe Zanetta^1^, Mr. Faris Abusharkh^1^, Sumner Gardner^1^, Dr. Fubiao Shi^1^, Elizabeth Kobeck^1^, Dr. James West^1^, Anna Hemnes^1^

^1^Vanderbilt University Medical Center, Nashville, USA

Obesity is recognized to be an important upstream risk factor in right ventricular (RV) dysfunction due to heart failure (Group 2 PH), but the precise mechanisms that drive obesity‐associated cardiac remodeling are not well understood. Our previous work has shown that increased expression of the natriuretic peptide clearance receptor (NPRC) may drive RV remodeling in response to high fat diet. In this study, we hypothesized that knockdown of NPRC prevents and reverses experimental Group 2 PH. NPRC knockout transgenic mice were generated to induce global, cardiomyocyte‐specific, or adipocyte‐specific knockout of NPRC. RV dysfunction due to Group 2 PH was induced by the 2‐hit stress of L‐NAME and high fat diet. Outcomes measured included echocardiography, exercise endurance, invasive catheterization, and histologic assessment. Differential gene expression in adipocyte NPRC knockout visceral adipose tissue was investigated via bulk RNA sequencing and validated by RT‐qPCR and culture of H9C2 cells with or without Wnt5a treatment. Adipocyte‐mediated Wnt ligand release was interrupted in vivo via LGK974 injection in mice subjected to L‐NAME and high fat diet. Global inducible NPRC knockout prevented and reversed structural, hemodynamic, echocardiographic, and exercise tolerance features of Group 2 PH. This effect was found to be driven by adipocyte‐specific, not cardiomyocyte‐specific NPRC expression. Bulk RNA sequencing of adipocyte‐specific NPRC knockout visceral adipose tissue identified downregulation of several secretory pathways, including Wnt pathways. Wnt5a was identified as one of the most downregulated genes by RNA sequencing and validated by qPCR. Wnt5a increased cell hypertrophy in vitro, and LGK974 treatment in vivo decreased circulating Wnt5a levels and improved cardiac remodeling in Group 2 PH. Our study identifies a novel crosstalk mechanism between cardiomyocytes and adipocytes in obesity‐associated HFpEF driven by natriuretic peptide‐mediated inhibition of release of pro‐hypertrophic stimuli in adipocytes.

## A075 Expression of Endocytic Molecules and Transmembrane Channel Genes in In Vitro Models of Dysregulated BMP/TGF‐β‐signaling

Mr. Nikolaos Lotsios^1^, Dr. Chrysi Keskinidou^1^, Prof. Ioanna Dimopoulou^1^, Prof. Anastasia Kotanidou^1^, Prof. David Langleben^2^, Prof. Stylianos E. Orfanos^3^, Dr. Alice G. Vassiliou^1^

^1^First Department of Critical Care Medicine & Pulmonary Services, School of Medicine, National and Kapodistrian University of Athens, Evangelismos Hospital, 106 76 Athens, Greece, ^2^Center for Pulmonary Vascular Disease, Azrieli Heart Center and Lady Davis Institute, Jewish General Hospital, McGill University, Montreal, QC H3T 1E2, Canada, ^3^School of Medicine, National and Kapodistrian University of Athens, 115 27 Athens, Greece

Over 70% of heritable pulmonary arterial hypertension (hPAH) involves mutations in the bone morphogenetic protein receptor type II (BMPR2) gene. BMPR2 is internalized in the cytoplasm via clathrin‐coated pits (CCPs), regulating the transcriptional response to BMP‐signaling. Clathrin‐mediated endocytosis (CME) controls cellular interactions, intercellular signaling pathways, and homeostasis. While BMPR2 is one of the principal regulators of vascular remodeling, the key driving force in PAH, dysregulated vasoconstriction may also occur. Mutations in the ATP‐binding cassette subfamily B member 8 (ABCC8) gene mainly result in reduced activity of the ATP‐sensitive potassium channel. Impaired ABCC8/SUR1 function could promote pulmonary vasoconstriction by reducing K‐ATP channel activity. Indeed, novel ABCC8 mutation variants have been identified and linked to PAH onset, even though the specific role of the SUR1/Kir6.2 channel in the pathophysiology of PAH is still unclear. Interestingly, elevated mRNA expression levels of ABCC8 have been described in lung tissues of patients with BMPR2‐associated hPAH. We evaluated the mRNA expression of ABCC8 and CLTC before and after BMP9 exogenous administration in AQP1‐, BMPR2‐, and TGFB1‐silenced human pulmonary microvascular endothelial cells (HPMEC‐ST1.6 R). In all three experimental models, CLTC mRNA expression decreased significantly (AQP1‐silenced: p < 0.001; BMPR2‐silenced: p < 0.05; TGFB1‐silenced: p < 0.001). Administration of exogenous BMP9 did not restore CLTC mRNA expression (AQP1‐silenced: p < 0.001; BMPR2‐silenced: p < 0.05; TGFB1‐silenced: p < 0.001). ABCC8 mRNA expression decreased in the AQP1‐silenced HPMECs (p < 0.05), while it tended to increase in the BMPR2‐silenced cells (p = 0.08), and it remained stable in the TGFB1‐silenced cells (p=ns). In all experimental models, exogenous BMP9 administration did not affect ABCC8 mRNA expression. All comparisons were against the non‐transfected control cells. Our preliminary data highlight the broad effect of disrupted BMP‐signaling on key molecules implicated in PAH. The interplay between impaired BMP signaling, reduced clathrin‐mediated trafficking, and disrupted ABCC8 expression could modulate both vascular remodeling and vasoconstriction in the setting of PAH.

## A076 Gut Mycobiome Modification With Fluconazole Reduces Pulmonary Arterial Hypertension Severity

Ms Rashmi Raveendran^1^, Elizabeth Ngo^1^, Benjamin Kremer^1^, Ryan Moon^1^, Dr. Sasha Prisco^1^

^1^Cardiovascular Division, Department of Medicine, Lillehei Heart Institute, University of Minnesota, Minneapolis, United States of America

Pulmonary arterial hypertension (PAH) is a progressive pulmonary vasculopathy characterized by chronic inflammation. Recent studies have highlighted the gut microbiome as a potential therapeutic target to attenuate systemic inflammation. However, little is known about the role of the intestinal fungi, or mycobiome, in PAH. Thus, we investigated the effects of modulating the gut mycobiome in two preclinical PAH rodent models, monocrotaline (MCT) and Sugen‐hypoxia (SuHx). Two weeks following MCT injection (60 mg/kg) and three weeks of hypoxia (10% O2) in the SuHx model, rats received a daily oral gavage of fluconazole (20 mg/kg) until day 24 in the MCT group and until day 42 in the SuHx group (after three weeks of normoxia). Echocardiography, closed‐chest invasive pressure‐volume loops, and histological analyses of lung and jejunum were completed. Stool metagenomics and internal transcribed sequencing defined the micro‐ and mycobiome, respectively. Deconvolution of bulk RNA‐sequencing data of lung tissue delineated the immune cell populations impacted. Fluconazole treatment (MCT‐F, SuHx‐F) mitigated PAH disease severity compared to vehicle‐treated rats (MCT‐V, SuHx‐V) as demonstrated by reduced right ventricular (RV) systolic pressure (p < 0.0001 MCT‐F vs. MCT‐V, p = 0.0005 SuHx‐F vs. SuHx‐V), effective arterial elastance (p < 0.0001 MCT‐F vs. MCT‐V, p = 0.0003 SuHx‐F vs. SuHx‐V), and percent medial wall thickness of the pulmonary arterioles (p < 0.0001 MCT‐F vs. MCT‐V, p = 0.03 SuHx‐F vs. SuHx‐V) and increased pulmonary artery acceleration time (p < 0.0001 MCT‐F vs. MCT‐V, p = 0.001 SuHx‐F vs. SuHx‐V). Fluconazole augmented RV function. Fluconazole altered the fecal bacterial and fungal communities, and normalized jejunal villi length and the number of goblet cells per villus. Deconvolution of bulk‐RNA sequencing of lung tissue revealed that fluconazole diminished pulmonary interstitial macrophage and mast cell abundance, which were validated by immunofluorescence. Modulating the intestinal mycobiome reduced PAH severity and augmented RV function, suggesting the gut mycobiome may contribute to PAH pathogenesis.

## A077 Pressure‐Based Estimates for Right Ventricular Ejection Fraction in Response to Treatment of Pulmonary Arterial Hypertension

Tharsiga Kanagasingam^1^, Jonas Lund^1^, Christoph Sinning^1^, Gunnar Lund^1^, Hans Klose^1^, Lars Harbaum^1^

^1^Universitätsklinikum Hamburg‐Eppendorf, Hamburg, Germany

Right ventricular (RV) dysfunction is a key determinant of outcomes in pulmonary arterial hypertension (PAH). A recently proposed single‐beat method enables estimation of RV ejection fraction (RVEF) from pressure signals obtained during right heart catheterisation (RHC). This method is based on two components, the predicted maximal RV pressure (Pmax) and end‐systolic pressure (ESP). RVEF can then be approximated as 1 − (ESP/Pmax). We evaluated the feasibility of pressure‐derived RVEF in patients with PAH and its response to targeted therapy. Two independent groups of PAH patients were included. In the first group, patients underwent same‐day cardiac magnetic resonance imaging (CMR) and RHC. In the second, patients had RHC before and within 24 months after initiation of therapy. Pmax was derived using a four‐parameter Weibull peak fit, and ESP was defined by approximating the point of maximal time‐varying RV elastance. All participants provided informed consent (NCT04654650). In the first group of patients (N = 36), CMR‐derived RVEF ranged from 12–68% (mean ± SD: 42 ± 15) and pressure‐derived RVEF from 7–68% (34 ± 17). The two methods correlated moderately (r = 0.59, p < 0.001), with a mean bias of 7% and limits of agreement from −24 to 38%. In the second group (N = 26), targeted therapy increased pressure‐derived RVEF from 35 ± 15% to 48 ± 19% (p = 0.012). Of the two components, Pmax remained unchanged (p = 0.6), while ESP decreased from 57 ± 22 to 47 ± 21 mmHg (p = 0.012). Pressure‐derived RVEF showed acceptable agreement with CMR and was responsive to targeted therapy in our PAH cohort, primarily through reductions in ESP. These findings support its use as a practical and treatment‐sensitive measure of RV function, although interpretation should acknowledge methodological limitations.

## A078 Bone Marrow Immunometabolic Dysfunction Links Clonal Hematopoiesis and the IRG1/Itaconate Axis in Pulmonary Arterial Hypertension

Dr. Golnaz Hesami^1,2^, Dr. Siavash Mansouri^1,2^, Tessa Schmachtel^3^, Dr. Chanil Valasarajan^1,2^, Samuel Olapoju^1^, Dr. Stefan Günther^2^, Dr. Baktybek Kojonazarov^1^, Prof. Dr. Andreas Weigert^4^, Prof. Dr. Sebastien Bonnet^5^, Prof. Dr. Stephen Y. Chan^6^, Prof. Dr. Michael Rieger^3^, Prof. Dr. Werner Seeger^1^, Prof. Dr. Rajkumar Savai^1,2^, Prof. Dr. Soni Savai Pullamsetti^1,2^

^1^Universities of Giessen and Marburg Lung Center (UGMLC), Institute for Lung Health (ILH), Cardio‐Pulmonary Institute (CPI), Member of the German Center for Lung Research (DZL), Giessen, Germany, Giessen, Germany, ^2^Max Planck Institute for Heart and Lung Research, Bad Nauheim, Germany, ^3^Department of Medicine, Hematology/Oncology Goethe University Hospital, Frankfurt, Germany, ^4^Institute of Biochemistry I, Goethe‐University, Frankfurt, Germany, ^5^Pulmonary Hypertension and Vascular Biology Research Group of Quebec Heart and Lung Institute, Department of Medicine, Laval University, Canada, ^6^Center for Pulmonary Vascular Biology and Medicine, Pittsburgh Heart, Lung, and Blood Vascular Medicine Institute, Division of Cardiology, Department of Medicine, University of Pittsburgh School of Medicine, Pittsburgh, USA

Pulmonary arterial hypertension (PAH) is a progressive and fatal cardiopulmonary disorder associated with aging, in which bone marrow (BM)‐derived monocytes and macrophages play major pathogenic roles. However, the contribution of their progenitors, hematopoietic stem cells (HSCs), remains poorly understood. Here, we investigate how HSC‐driven mitochondrial metabolism influences PAH pathogenesis and mediates crosstalk among the BM, heart, and lung. We first assessed the prevalence of clonal hematopoiesis of indeterminate potential (CHIP), a condition driven by somatic HSC mutations that promote proinflammatory myelopoiesis. CHIP‐associated mutations were detected in 29% of cases, most frequently in DNMT3A, followed by TET2 and JAK2. Given the critical role of mitochondrial metabolism in CHIP‐HSC clonal expansion, DNMT3A‐CHIP macrophages exhibited elevated succinate dehydrogenase (SDH) activity, resulting in altered mitochondrial metabolism and a proinflammatory phenotype. Consistent with these findings, matrix‐assisted laser desorption/ionization mass spectrometry imaging (MALDI‐MSI) of BM samples from PAH patients revealed dysregulation of tricarboxylic acid (TCA) cycle metabolites. Accordingly, we focused on itaconate, a TCA‐derived metabolite and potent endogenous inhibitor of SDHA. Regression analysis in a cohort of approximately 1,900 PAH patients demonstrated that lower plasma itaconate levels significantly correlated with worsening hemodynamic parameters. In an ex vivo co‐culture model, wild‐type human heart organoids exposed to CHIP‐DNMT3A macrophages displayed increased collagen deposition, which was significantly attenuated by itaconate treatment. Therapeutic administration of an itaconate derivative in monocrotaline rats mitigated PH. Moreover, Irg1 knockout (Irg1⁻/⁻) mice, deficient in the enzyme responsible for itaconate production, developed a spontaneous PH‐associated phenotype. Irg1⁻/⁻ mice showed expanded myeloid populations in the heart and lung, linked to enhanced lymphoid‐primed multipotent progenitor (MPP‐Ly) population and increased egress of BM cells, consequent to activation of purinergic and interferon signaling. Collectively, our findings reveal that CHIP‐associated metabolic alterations and deficient IRG1/itaconate signaling drive hematopoietic remodeling and pathological myeloid expansion through immunometabolic dysregulation, contributing to PAH pathogenesis.

## A079 Seralutinib Targets Fibrotic Pathways in IPF: Evidence From Single‐Cell Transcriptomics

Dr. Rui Benfeitas^1^, Dr. Robin Osterhout^2^, Mr. Eduardo Garcia^2^, Dr. Ravikumar Sitapara^2^, Dr. Vanessa Pitozzi^1^, Dr. Sidra Hoffman^2^, Dr. Lawrence Ho^2^, Dr. Zhaoqing Ding^2^, Dr. Luís Magalhães^1^, Dr. Silvia Parolo^3^, Dr. Richard Aranda^2^, Prof. Gísli Jenkins^4^, Dr. Toby M. Maher^5^, Dr. Jean‐Marie Bruey^2^, Daniel Bachteler, Dr. Roham T. Zamanian^6^

^1^Chiesi Farmaceutici S.p.A., Parma, Italy, ^2^Gossamer Bio, Inc., San Diego, CA, United States, ^3^Fondazione the Microsoft Research ‐ University of Trento Centre for Computational and Systems Biology (COSBI), Rovereto, Italy, ^4^National Heart and Lung Institute, Imperial College London, London, United Kingdom, ^5^Keck School of Medicine of USC, Los Angeles, CA, United States, ^6^Stanford University School of Medicine, Stanford Medicine, Stanford, CA, United States

Seralutinib, an inhaled receptor tyrosine kinase inhibitor under development for pulmonary hypertension, including pulmonary arterial hypertension and interstitial lung disease, reduced circulating proteins associated with extracellular matrix (ECM) remodeling in the phase 2 TORREY trial (NCT04456998). To further investigate its antifibrotic potential, we identified seralutinib‐associated signatures in a human precision‐cut lung slice (PCLS) fibrosis model and mapped them to scRNAseq profiles from idiopathic pulmonary fibrosis (IPF) patient lungs. Seralutinib‐associated signatures were identified from circulating proteins in TORREY and RNA‐seq analysis of PCLS cultures stimulated with pro‐fibrotic cocktail and treated with 10 μM seralutinib. Signatures were mapped to seven public scRNAseq datasets (80 IPF; 58 controls; 486,114 cells). ECM‐related proteins decreased by seralutinib in TORREY, including COL1A1, COL6A3, COL24A1 and ITGB1, were upregulated in IPF myofibroblasts, fibroblasts, AT2 and endothelial cells. Top enriched pathways included ECM organization, epithelial‐mesenchymal transition (EMT), and collagen turnover. In the PCLS model, seralutinib significantly reversed fibrotic patterns, downregulating 262 genes associated with ECM, EMT and epithelial repair pathways. This gene signature is significantly elevated across many IPF cell populations including fibroblasts, AT2, secretory cells and macrophages. Seralutinib downregulated pathways elevated in IPF disease‐relevant cell populations, suggesting a broad, multi‐cellular impact on fibrosis, thus supporting the development of seralutinib as a novel inhaled therapy for fibrotic lung diseases. These data have been previously presented at the ERS Congress 2025.

## A080 Seralutinib Decreases Endotrophin (PRO‐C6) Production, a Mediator of Fibrosis and Inflammation, in an In vitro Model of Pulmonary Fibrosis

Dr. Zhaoqing Ding^1^, Dr. Mark Kuipers‐Skarsfeldt^2^, Dr. Robin Osterhout^1^, Dr. Filipa B. Simões^2^, Dr. Jannie Marie Bülow Sand^2^, Dr. Lawrence S. Zisman^1^, Dr. Sidra Hoffman^1^, Dr. Robert F. Roscigno^1^, Dr. Richard Aranda^1^, Dr. Lawrence Ho^1^, Dr. Ravikumar Sitapara^1^, Mr. Eduardo Garcia^1^, Dr. Anna R. Hemnes^3^, Dr. Roham T. Zamanian^4^, Dr. Bruno Guedes Baldi^5^, Prof. Rogério Souza^5^, Dr. Tomás Pulido^6^, Prof. Dr. Morten Karsdal^2^, Dr. Jean‐Marie Bruey^1^, Daniel Bachteler, Prof. Toby M. Maher^7^

^1^Gossamer Bio, Inc., San Diego, CA, United States, ^2^Nordic Bioscience, Herlev, Denmark, ^3^Vanderbilt University, Vanderbilt University Medical Center, Nashville, TN, United States, ^4^Stanford University School of Medicine, Stanford Medicine, Stanford, CA, United States, ^5^Instituto do Coração, Hospital das Clínicas da Faculdade de Medicina da Universidade de São Paulo, São Paulo, Brazil, ^6^Instituto Nacional de Cardiología Ignacio Chávez, México, D. F., México, ^7^Keck School of Medicine of USC, Los Angeles, CA, United States

Pulmonary hypertension (PH) is characterized by progressive vascular remodeling, contributed in part by extracellular matrix (ECM) deposition. In the phase 2 TORREY study (NCT04456998) treatment with seralutinib, an inhaled tyrosine kinase inhibitor, was associated with a reduction of ECM components in the plasma, such as COL6A3, highlighting its anti‐fibrotic effect. A cleavage product of COL6A3, endotrophin (PRO‐C6), derived from activated fibroblast has been shown to promote fibrosis and inflammation, which in turn propagates further tissue remodeling in diseases associated with cardiac and hepatic fibrosis. This study aims to investigate the effect of seralutinib on production of PRO‐C6 in pulmonary fibrosis. Primary healthy lung fibroblasts (NHLFs) were cultured using the Scar‐in‐a‐jar model and exposed to a pro‐fibrotic cytokine cocktail (FC) for 12 days. FC, seralutinib (10‐1000 nM), and treatment controls were administered on days 0, 4, and 8. On day 12, cell culture supernatants were collected to quantify NordicPRO‐C6™ levels. Stimulating NHLFs with the FC increased PRO‐C6 by 2.3‐fold at day 12 compared to unstimulated cells. By day 12, seralutinib treatment reduced PRO‐C6 levels in a concentration‐dependent manner (1.3‐ to 2.3‐fold), with 1000 nM seralutinib reducing PRO‐C6 levels to those observed in unstimulated cells. Seralutinib decreased PRO‐C6 production in an in vitro model of pulmonary fibrosis providing a compelling rationale to further investigate its potential as a surrogate biomarker of pulmonary fibrosis and its role in the pathophysiology of vascular remodeling in PH. These data have been previously presented at the ERS Congress 2025.

## A081 Trial in Progress: SERANATA, a Phase 3 Randomized, Placebo‐Controlled Trial to Evaluate Inhaled Seralutinib for Treatment of Pulmonary Hypertension Associated With Interstitial Lung Disease (PH‐ILD)

Dr. Oksana Shlobin^1^, Dr. Sidra Hoffman^2^, Dr. Lawrence Ho^2^, Dr. Richard Aranda^2^, Dr. Ayodeji Adegunsoye^3^, Prof. Roberto Badagliacca^4^, Prof. Dr. Jürgen Behr^5^, Dr. Robert P. Frantz^6^, Prof. Dr. Hossein‐Ardeschir Ghofrani^7,8^, Dr. Kristin B. Highland^9^, Dr. Vallerie V. McLaughlin^10,11^, Dr. Lana Melendres‐Groves^12^, Dr. Lucilla Piccari^13^, Prof. Dr. Stephan Rosenkranz^14^, Dr. Rajan Saggar^15^, Dr. Francisca Gavilanes^2^, Dr. Luís Magalhães^16^, Mr. Glauco Cappellini^16^, Mr. Kartik Raghupathi^2^, Daniel Bachteler, Prof. Vincent Cottin^17^

^1^Inova Fairfax Hospital, Falls Church, VA, United States, ^2^Gossamer Bio, Inc., San Diego, CA, United States, ^3^University of Chicago, Chicago, IL, United States, ^4^University of Rome, Rome, Italy, ^5^Ludwig‐Maximilians University, Munich, Germany, ^6^Department of Cardiovascular Medicine, Mayo Clinic, Rochester, MN, United States, ^7^Department of Internal Medicine, Justus‐Liebig‐University Giessen and Marburg Lung Center (UGMLC); Institute for Lung Health, Cardio‐Pulmonary Institute; Member of the German Center for Lung Research (DZL), Giessen, Germany, ^8^Department of Medicine, Imperial College, London, United Kingdom, ^9^Cleveland Clinic, Cleveland, OH, United States, ^10^Department of Cardiovascular Medicine, University of Michigan, Ann Arbor, MI, United States, ^11^Frankel Cardiovascular Center, Ann Arbor, MI, United States, ^12^University of New Mexico, Albuquerque, NM, United States, ^13^Hospital del Mar, Barcelona, Spain, ^14^University of Cologne, Cologne, Germany, ^15^University of California Los Angeles, Los Angeles, CA, United States, ^16^Chiesi Farmaceutici S.p.A., Parma, Italy, ^17^Centre Hospitalier Universitaire de Lyon, Lyon, France

Seralutinib is an inhaled tyrosine kinase inhibitor targeting PDGFR, CSF1R, and c‐KIT pathways that drive inflammation, proliferation, and fibrosis, key to pulmonary vascular remodeling in pulmonary hypertension. In the phase 2 TORREY study in adults with pulmonary arterial hypertension (PAH), inhaled seralutinib significantly reduced pulmonary vascular resistance (PVR) versus placebo and was well tolerated. Based on similarities in the pulmonary vascular changes in PAH and pulmonary hypertension associated with interstitial lung disease (PH‐ILD) and demonstrated clinical efficacy in PAH, there is a strong rationale to assess seralutinib in PH‐ILD. SERANATA is a phase 3, randomized, double‐blind, placebo‐controlled study to evaluate the efficacy and safety of two doses of seralutinib in 480 adults with PH‐ILD (NCT07181382, EUDRA‐CT 2025‐521777‐13‐00). Eligible patients are ≥ 18 years with a diagnosis of WHO Group 3 PH with fibrosing ILD confirmed by RHC with PVR ≥ 4 WU, pulmonary capillary wedge pressure ≤ 15 mmHg, and mean pulmonary artery pressure ≥ 25 mmHg. Patients are randomized 1:1:1 to twice daily seralutinib 90 mg, 120 mg, or placebo administered by a dry powder inhaler during a 24‐week double‐blind period. The primary endpoint is change from baseline to week 24 in six‐minute walk distance. Key secondary endpoints include time to clinical worsening, change in NT‐proBNP and change in absolute FVC from baseline to week 24. Safety endpoints include adverse events, changes in laboratory parameters, pulmonary function and oxygenation, and exacerbations of ILD. Patients continuing into the long‐term extension remain on their respective seralutinib dose or switch from placebo to seralutinib 90 mg or 120 mg in 1:1 randomized fashion for continued blinded evaluation of safety and efficacy. Exploratory hemodynamic, echocardiographic, and lung HRCT substudies are being conducted to further evaluate the effects of seralutinib on both the pulmonary vasculature and pulmonary fibrosis.

## A082 Seralutinib Shows Significant Anti‐Fibrotic Effects: Evidence From Patient‐Derived Models

Dr. Ravikumar Sitapara^1^, Dr. Silvia Cantoni^2^, Mr. Eduardo Garcia^1^, Ms. Valentina Violi^2^, Dr. Zhaoqing Ding^1^, Dr. Robin Osterhout^1^, Dr. Lawrence S. Zisman^1^, Dr. Sidra Hoffman^1^, Dr. Lawrence Ho^1^, Dr. Francisca Gavilanes^1^, Dr. Richard Aranda^1^, Dr. Daniela Miglietta^2^, Dr. Tomás Pulido^3^, Dr. Bruno Guedes Baldi^4^, Prof. Rogério Souza^4^, Dr. Jean‐Marie Bruey^1^, Daniel Bachteler, Prof. Vincent Cottin^5^

^1^Gossamer Bio, Inc., San Diego, CA, United States, ^2^Chiesi Farmaceutici S.p.A., Parma, Italy, ^3^Instituto Nacional de Cardiología Ignacio Chávez, México, D. F., México, ^4^Instituto do Coração, Hospital das Clínicas da Faculdade de Medicina da Universidade de São Paulo, São Paulo, Brazil, ^5^Centre National de Référence des Maladies Pulmonaires rares, Service de Pneumologie, Hospices Civils de Lyon, Hôpital Louis Pradel, Lyon, France

Fibrosis contributes to both pulmonary vascular and parenchymal remodeling in pulmonary hypertension (PH) and interstitial lung disease (ILD). Seralutinib is a potent inhaled PDGFRα/ß, CSF1R and c‐KIT kinase inhibitor in clinical development for PAH and PH‐ILD. In the phase 2 TORREY study (NCT04456998) in Group 1 PH patients, seralutinib decreased circulating fibrotic biomarkers, including collagen 1a1. To test whether seralutinib inhibits established fibrosis in idiopathic pulmonary fibrosis (IPF) patient‐derived precision‐cut lung slices (PCLS) and lung fibroblasts (HLF).

PCLS from two IPF patient donors were cultured and treated for 4 days with vehicle, seralutinib (0.1‐10 μM), or nintedanib (1 μM). Supernatants were collected and pro‐fibrotic markers evaluated. The antifibrotic effect was also studied in IPF patient‐derived lung fibroblasts with the same treatments. Statistical significance was determined by one‐way ANOVA with nominal p < 0.05. PCLS from both IPF donors constitutively released pro‐fibrotic markers. Seralutinib dose‐dependently decreased pro‐collagen 1a1, fibronectin, MMP3, TIMP1 and MCP1. This reduction either matched or exceeded that of nintedanib at clinically relevant concentrations in both IPF PCLS. In the HLF assay, seralutinib significantly inhibited the release of pro‐collagen 1a1 and MCP1 in a dose‐dependent manner. Seralutinib also inhibited αSMA mRNA and protein expression, a marker of myofibroblast activation.

These data support investigation of seralutinib as a potential inhaled treatment option for diseases characterized by underlying pulmonary vascular and interstitial fibrosis, including PH and other ILDs.

These data have been previously presented at the ERS Congress 2025.

## A083 Seralutinib Demonstrates In vitro Reduction of Vascular Inflammatory Drivers Underlying Pulmonary Hypertension

Dr. Zhaoqing Ding^1^, Dr. Elisa Schiavi^2^, Dr. Silvia Cantoni^2^, Dr. Ravikumar Sitapara^1^, Mr. Eduardo Garcia^1^, Ms. Giulia Nuozzi^2^, Dr. Jessica Burroughs‐Garcia^2^, Dr. Robin Osterhout^1^, Dr. Lawrence S. Zisman^1^, Dr. Sidra Hoffman^1^, Dr. Richard Aranda^1^, Dr. Daniela Miglietta^2^, Prof. Dr. Stephan Rosenkranz^3^, Dr. Anna R. Hemnes^4^, Dr. Adam J. Byrne^5^, Dr. Jean‐Marie Bruey^1^, Daniel Bachteler, Dr. Cormac McCarthy^6^

^1^Gossamer Bio, Inc., San Diego, CA, United States, ^2^Chiesi Farmaceutici S.p.A., Parma, Italy, ^3^Department of Cardiology ‐ Internal Medicine III, Heart Center, University Hospital Cologne; Cologne Cardiovascular Research Center (CCRC), University of Cologne, Cologne, Germany, ^4^Vanderbilt University, Vanderbilt University Medical Center, Nashville, TN, USA, ^5^University College Dublin, School of Medicine, Dublin, Ireland, ^6^University College Dublin, School of Medicine, St. Vincent's Hospital, Dublin, Ireland

In pulmonary hypertension (PH), immune cell accumulation around the pulmonary vasculature correlates with disease severity, underscoring its role in vascular remodeling. Seralutinib, a potent inhaled tyrosine kinase inhibitor, significantly reduced pulmonary vascular resistance in the phase 2 TORREY study (NCT04456998) and was associated with reduced circulating inflammation related proteins in patients (MIP1α and CSF2) and in a preclinical model of pulmonary arterial hypertension (TNFα and MCP1). This study assesses the impact of seralutinib on in vitro models of vascular inflammation. Seralutinib's (8 nM–10 μM) effect on vascular inflammation was tested on primary human cells (Eurofin BIOMAP; n = singlet mix of 2–6 healthy donors) that model different biological responses, such as venular endothelial cells (VECs) stimulated under Th1 (TNFα, IFNγ, IL‐1β), Th2 (IL‐4, histamine) conditions, or co‐cultured with peripheral blood mononuclear cells (PBMCs) and stimulated with superantigen (SAg±anti‐IgM) or TLR4 ligand. Supernatant or cell pellets were measured for protein expression of inflammation related molecules by ELISA after 24–72 hours.

In VEC alone or in co‐cultures with PBMCs, seralutinib in a dose‐dependent manner significantly reduced pro‐inflammatory cytokines (IL‐8, TNFα, IL‐17A/F), chemokines (CXCL9), adhesion (VCAM1), and integrin molecules (SELE). Seralutinib demonstrated broad anti‐inflammatory effects across multiple in vitro vascular inflammation models. These findings, combined with clinical biomarker data, highlight seralutinib's potential for reducing the underlying inflammation in PH, and merit further investigation. These data have been previously presented at the ERS Congress 2025.

## A084 AI‐Driven Risk Prediction in Pulmonary Arterial Hypertension: Imaging‐Based Alternatives to Invasive Models

MS Max Cartright^1^, Enterprise Analytics Consultant Priscilla Correa‐Jaque^2^, MD Charles Fauvel^3^, MD David G. Kiely^4^, MD Raymond L. Benza^2^, PhD Shili Lin^1^

^1^Department of Statistics, The Ohio State University, Columbus, United States, ^2^Sentara College of Health Sciences, Sentara Health, Norfolk, United States, ^3^Cardiology Department, Rouen University Hospital, Rouen, France, ^4^University of Sheffield Medical School, Sheffield, United Kingdom

Pulmonary arterial hypertension (PAH) is a progressive condition where accurate risk stratification is essential for guiding therapy and improving survival. Current models such as REVEAL 2.0 and the European 3‐strata incorporate invasive hemodynamic data from right heart catheterization, which limits accessibility and increases procedural risk. Cardiovascular magnetic resonance (CMR) imaging offers a non‐invasive alternative, yet its integration into risk prediction frameworks remains underexplored. To assess whether CMR‐derived imaging metrics can replace invasive parameters in established PAH risk models without reducing prognostic accuracy. We analyzed 343 patients with confirmed PAH from the UK ASPIRE registry. Forty‐nine CMR variables were consolidated into 12 biologically coherent “mega‐imaging” features. These were substituted for invasive measures within existing risk models. Predictive performance for one‐year survival was evaluated using AUC, sensitivity, and specificity. Replacing invasive variables with mega‐imaging features preserved or improved model performance. For REVEAL 2.0, AUC increased from 0.75 to 0.77; for the European 3‐strata, AUC rose from 0.60 to 0.72. While improvements were not statistically significant, trends consistently favored imaging‐based models, suggesting potential clinical benefit. CMR‐derived imaging features can effectively substitute invasive hemodynamic measures in PAH risk stratification models without compromising predictive accuracy. This imaging‐driven approach offers a safer, cost‐effective, and scalable alternative, supporting broader adoption of comprehensive risk assessment in routine practice.

Keywords: Pulmonary Arterial Hypertension; Risk Stratification; Cardiovascular Magnetic Resonance; Artificial Intelligence; Non‐Invasive Prognostic Models.

Impact Statement: This study demonstrates that AI‐driven integration of CMR imaging can replace invasive measures in PAH risk models without loss of predictive power. By enabling safer, cost‐effective, and scalable risk assessment, this approach has the potential to transform clinical practice and improve accessibility for patients worldwide.

## A085 Transcriptomic Signatures of Pulmonary Vascular Remodeling in COPDGene

Dr. Vasile Foris^1,2^, Dr. Raul San Jose Estepar^3^, Dr. Farbod N. Rahaghi^4^, Jose S. Carbera^1^, Dr. Raymond Chad Wade^5^, Dr. James Michael Wells^5^, Dr. Jeong H. Yun^1,6^, Aabida Saferali^1^, Dr. Craig P. Hersh^1,6^, Dr. Edwin K. Silverman^1,5^, Dr. Michael H. Cho^1,5^, Dr. Peter J. Castaldi^1,7^, Dr. Adel Boueiz^1,6^

^1^Channing Division of Network Medicine, Department of Medicine, Brigham and Women's Hospital, Harvard Medical School, Boston, USA, ^2^Division of Pulmonology, Department of Internal Medicine, Medical University of Graz, Graz, Austria, ^3^Surgical Planning Laboratory, Laboratory of Mathematics in Imaging, Department of Radiology, Brigham and Women's Hospital, Harvard Medical School, Boston, USA, ^4^Division of Pulmonary and Critical Care Medicine, Department of Medicine, University of California, Los Angeles, USA, ^5^Division of Pulmonary, Allergy, and Critical Care Medicine, University of Alabama at Birmingham, Birmigham, USA, ^6^Division of Pulmonary and Critical Care Medicine, Department of Medicine, Brigham and Women's Hospital, Boston, USA, ^7^Division of General Medicine and Primary Care, Brigham and Women's Hospital, Harvard Medical School, Boston, USA

CT‐derived measures of pulmonary artery (PA) morphology, including PA diameter, PA–to–aorta (PA/A) ratio, and pulmonary artery surface‐to‐volume ratio (PAs/v) obtained through automated segmentation, provide quantitative markers of vascular remodeling, characteristic of pulmonary vascular disease (PVD). Such traits predict adverse outcomes in COPD. However, the molecular mechanisms underlying these structural phenotypes remain poorly understood. We leveraged whole‐blood RNA sequencing (RNA‐seq) to identify transcriptomic signatures of PA metrics. We analyzed 3,704 participants from the COPDGene Phase 2 cohort with CT‐based PA measurements and RNA‐seq data. Differential gene expression was assessed using the voom/limma method, adjusting for relevant covariates. Pathway enrichment was evaluated using Gene Set Enrichment Analysis. Gene correlation networks, immune deconvolution, and integrative analyses were applied to link transcriptomic, genetic, and clinical features of PVD. A total of 4,607 genes were associated with PA diameter, 17 with PA enlargement (PA/A > 1), and 1,239 with PAs/v (FDR < 0.05). While each imaging metric exhibited a distinct molecular profile, shared upregulated genes reflected innate and adaptive immunity (GZMA, LY96), cell cycle regulation (CKS2), fatty acid synthesis (ELOVL6), and redox regulation (GLRX). Shared downregulated genes were enriched for actin binding, calcium ion binding, and tyrosine kinase activity (ABLIM1, SMARCD2, PTK7, CCNI2). Trait‐specific pathway analysis revealed enrichment in 1,889 pathways for PA diameter, 258 for PA enlargement and 127 for PAs/v. Vascular remodeling mediators (PDGF, EGFR) predominated for PA diameter and enlargement, whereas eicosanoid, glycosphingolipid, and ceramide signaling pathways were common across traits (FDR < 0.05). Integrative network and cell‐type analyses highlighted immune activation and vascular signaling modules associated with vascular remodeling. Distinct and overlapping transcriptomic signatures of CT‐based PA metrics implicate immune activation and vascular signaling in pulmonary vascular remodeling in COPD. Validation in independent cohorts and multi‐omic integration will refine these signatures and their translational potential.

## A086 Heart Rate Variability (HRV) as a Prognostic Marker in Pediatric Pulmonary Arterial Hypertension (PAH)

Dr Prashant Raviprakash Bobhate^1^

^1^Kokilaben Dhirubai Ambani Hospital, Mumbai, India

Pediatric PAH risk stratification is challenging due to the varied age and functional capabilities of patients, highlighting the need for a non‐invasive, objective parameter. This study aimed to evaluate Heart Rate Variability (HRV) as a prognostic marker in pediatric PAH patients.

A prospective observational study tracked 50 pediatric patients with idiopathic PAH, stable on therapy, from March 2022 to May 2024. Patients underwent clinical evaluation, echocardiograms, NT‐proBNP, and Holter monitoring to measure HRV parameters: SDNN (standard deviation of normal‐to‐normal intervals), SDANN (standard deviation of mean normal‐to‐normal intervals), and RMSSD (square root of the mean square differences of successive RR intervals). Patients were followed up for a mean of 372\pm 182.1 days to assess for disease progression (worsening functional class, > 10\% decrease in 6‐minute distance, increase in NT‐proBNP, mortality, or need for transplant/shunt). Key Results: * 40% (20/50) of patients showed disease progression, including 10 deaths. * The progressive group had significantly lower HRV values compared to the non‐progressive group (all p = 0.001): * SDNN: 48.2\pm 32.6 ms vs. 86.7\pm 26.8 ms * SDANN: 38.6\pm 24.9 ms vs. 68.2\pm 24.9 ms * RMSSD: 17.4\pm 9.9 ms vs. 36.4\pm 13.8 ms * Specific cut‐off values for SDNN (\mathbf{63\text{ms}}), SDANN (\mathbf{49\text{ms}}), and RMSSD (\mathbf{20.9\text{ms}}) demonstrated good sensitivity (75‐86%) and specificity (65‐80%) in predicting progression.

HRV is significantly lower in pediatric PAH patients who experience disease progression. It shows promise as a non‐invasive, objective parameter for risk prediction, which could facilitate earlier therapeutic intervention.

## A087 A Preliminary Study on the Construction of Organoids Derived from Patients with Chronic Thromboembolic Pulmonary Hypertension

Research Fellow, Associate Professor Ran Miao^1^, Vice Director of Department of Pulmonary and Critical Care Medicine, China‐Japan Friendship Hospital, Director of Research Office, National Center for Respiratory Medicine Zhenguo Zhai^2^, Doctor of Medicine Zhu Zhang^2^, Doctor of Medicine Shuai Zhang^2^, Doctor of Medicine Jixiang Liu^2^

^1^National Center for Respiratory Medicine; State Key Laboratory of Respiratory Health and Multimorbidity; National Clinical Research Center for Respiratory Diseases; Institute of Respiratory Medicine, Chinese Academy of Medical Sciences; National Center for Respiratory Medicine Labs, Center of Respiratory Medicine, China‐Japan Friendship Hospital, Beijing, China, Beijing, China, ^2^National Center for Respiratory Medicine; State Key Laboratory of Respiratory Health and Multimorbidity; National Clinical Research Center for Respiratory Diseases; Institute of Respiratory Medicine, Chinese Academy of Medical Sciences; Department of Pulmonary and Critical Care Medicine, Center of Respiratory Medicine, China‐Japan Friendship Hospital, Beijing, P.R. China, Beijing, China

Current research on chronic thromboembolic pulmonary hypertension (CTEPH) is heavily dependent on animal models and 2D cell cultures, which suffer from significant limitations in recapitulating human pathological features. There is an urgent need for a translational disease model that can faithfully mimic the pathophysiological progression of CTEPH. This study aimed to establish a robust methodology for generating vascular organoids from patient‐derived pulmonary endarterectomy tissues and characterize their structural, cellular, and molecular properties. Tissue from pulmonary artery endarterectomy in patients with CTEPH was processed for organoid establishment. After washing, fragmentation, and Matrigel seeding, organoids were cultured with specific media. Passaging and subculturing followed. Immunohistochemistry with antibodies identified cell types. Vascular network formation was assessed via Matrigel‐based assays. The study successfully established self‐organizing vascular organoids that maintained 3D architectural integrity in culture. Immunohistochemical analysis revealed stratified structures composed of CD31+ endothelial cells, α‐SMA+ smooth muscle cells, and PDGFR β+ pericytes to recapitulating the vascular wall organization of native pulmonary arteries. Matrigel assays demonstrated robust tube formation capacity, with organoid‐derived cells forming patent capillary‐like networks. This study successfully constructed a vascular organoid model based on patient derived pulmonary artery endarterectomy tissue of patients with CTEPH, which faithfully recapitulates human pulmonary arterial architecture, cellular heterogeneity, and disease‐relevant gene expression profiles. This platform may provide a physiologically relevant tool for mechanistic studies of CTEPH progression, high‐throughput drug screening, and personalized therapeutic development, addressing critical gaps in current preclinical modeling approaches.

## A088 Functional Assessment of Pulmonary Gas Exchange in Pah Associated With Systemic Sclerosis Using Hyperpolarised 129Xe MRI

Dr Laura Saunders^1,3^, Dr Scarlett Strickland^1,2^, Dr Muad Almsaaid^1^, Dr Guilhem J Collier^1^, Dr Paul J C Hughes^1^, Dr Rebecca Burney^1,2^, Ian Smith^2^, Dr Laurie Smith^1^, Dr Graham Norquay^1^, Dr Jemima Pilgrim‐Morris^1^, Dr Helen Marshall^1,3^, Dr Alberto Biancardi^1^, Mack Caraher^1^, Shruti Kulkarni^2^, Felicity Hitchcock^2^, Dr Neil J Stewart^1,3^, Professor Andy Swift^1,2^, Professor Alex Rothman^1,2^, Dr Robin Condliffe^1,2^, Professor David Kiely^1,2^, Professor Jim Wild^1,3^, Dr A A Roger Thompson^1,2^

^1^The University of Sheffield, Sheffield, United Kingdom, ^2^Sheffield Teaching Hospitals, Sheffield, United Kingdom, ^3^Insigneo institute for in silico medicine, Sheffield, United Kingdom

Pulmonary arterial hypertension (PAH) is a major cause of mortality in systemic sclerosis (SSc) and early detection of pulmonary vasculopathy could potentially improve outcomes. Hyperpolarised 129Xe magnetic resonance imaging (129Xe MRI) is a non‐invasive technique which can assess pulmonary gas exchange, including uptake of xenon into the membrane (M:gas), xenon gas transfer to the capillaries (RBC:M) and alveolar‐to‐blood xenon transfer (RBC:gas). We hypothesised that 129Xe MRI metrics could differentiate SSc patients with PAH (SSc‐PAH) from those without (SSc‐noPAH) by detecting vasculopathy‐related impairments and heterogeneity in gas transfer.

SSc patients with suspected PAH were prospectively recruited at a single centre and underwent 1.5 T 1H and 129Xe lung MRI. Patients with extensive ILD were excluded. Dissolved‐phase 129Xe imaging metrics included RBC:M, M:gas and RBC:gas z‐scores (calculated relative to 62 healthy volunteers). RBC:M coefficient of variation (CV) and RBC:gas CV and M:gas CV assessed gas transfer heterogeneity. 1H‐MRI ultra‐short echo time assessed parenchymal abnormalities, quantified using normalised lung signal intensity (LSnorm). Right heart catheterisation (RHC) was performed on the same day. Of 36 patients (64 ± 7 years, 33 female), 13 had SSc‐noPAH and 23 had SSc‐PAH. RBC:M z‐score was lower in SSc‐PAH (‐4.34 ± 1.38 vs ‐2.51 ± 1.13, p < 0.001), as was RBC:gas z‐score (‐2.35 vs ‐1.40, p = 0.005) and M:gas z‐score was elevated (1.57 ± 1.38 vs 0.51 ± 0.74). SSc‐PAH showed greater gas transfer heterogeneity (RBC:M CV 0.47 ± 0.09 vs 0.39 ± 0.08, p = 0.023). LSnorm was higher in SSc‐PAH (0.035 ± 0.07 vs 0.029 ± 0.05, p = 0.044). RBC:M z‐score correlated with mean PAP (r = ‐0.706, p < 0.001) and pulmonary vascular resistance (r = ‐0.701, p < 0.001). In this ongoing study,129Xe MRI gas transfer metrics distinguished SSc‐PAH from SSc‐noPAH and correlated with haemodynamic assessments, suggesting utility as a non‐invasive tool to detect pulmonary vasculopathy in SSc patients without extensive ILD. These findings support further exploration of 129Xe MRI for early diagnosis and monitoring in SSc‐PAH.

## A089 The Epidemiology and Profile of Pulmonary Hypertension in sub‐Saharan Africa

Dr. Katarina Zeder^1,2,3^, Dr. Lanjing Wang^4^, Ms Veranyuy Ngah^1,5^, Dr. Armella Santi^1^, Dr. Eric W Robbins^2^, Prof. Dr. Gabor Kovacs^3^, Prof. Dr. Guoqing Diao^4^, Prof. Dr. Bradley A Maron^1,6^

^1^University of Maryland Institute of Health Computing, North Bethesda, USA, ^2^University of Maryland, School of Medicine, Division of Pulmonary, Critical Care and Sleep Medicine, Baltimore, USA, ^3^Department of Internal Medicine, Division of Respiratory Medicine, Lung Research Cluster, Medical University of Graz, Graz, Austria, ^4^Department of Biostatistics and Bioinformatics, George Washington University, Washington, USA, ^5^Division of Epidemiology and Biostatistics, Department of Global Health, Faculty of Medicine and Health Sciences, Stellenbosch University, Stellenbosch, South Africa, ^6^Department of Medicine, University of Maryland School of Medicine, Baltimore, USA

Non‐communicable diseases (NCDs) are on the rise in sub‐Saharan Africa (SSA), with cardiovascular NCD accounting for most deaths. Pulmonary hypertension (PH) is a highly morbid NCD; however, the prevalence and profile of PH in SSA is unknown. We performed a systematic literature search of all echocardiographic or right heart catheterization studies performed in SSA. We grouped studies based on disease populations and performed random‐effects meta‐analyses, meta‐regression analyses, and extrapolation. Extrapolation was based on regional data from the World Health Organization and Global Burden of Disease Study. A tricuspid regurgitation velocity (TRV) > 2.8 m/s and > 3.4 m/s were used to assess ‘at risk’ and ‘likely’ PH, respectively. This study adhered to PRISMA, MOOSE and GATHER guidelines. We included n = 105 reports, comprising 23,384 individuals and echocardiography was used in n = 103 (98.1%) studies. Most investigated cohorts included sickle cell disease (SCD; n = 24), human immunodeficiency virus (HIV; n = 19), rheumatic heart disease (RHD; n = 18), hypertensive heart failure (HHF; n = 7), and other heart failure (HF) syndromes (n = 18), while others, including chronic obstructive pulmonary disease (COPD), pulmonary tuberculosis (TB), kidney failure (CKD) and schistosomiasis were underreported, and PH prevalence data were extrapolated from other global regions. Estimated PH prevalence across the cohorts was strongly dependent on TRV threshold, referral reason for echocardiography particularly for HIV, and disease severity. When taking these factors into account, extrapolation indicated that 1.2% of the SSA population had ‘likely’ PH and 1.9% ‘at risk’ PH in 2021, with the largest contributor being RHD (31%), followed by other HF (25%), COPD (18%), CKD (14%), HIV (5%), SCD (4%), schistosomiasis (2%) and TB (1%). PH has a unique epidemiological profile in SSA. PH prevalence is substantial but varies by disease population, disease severity, and specificity of diagnostic criteria. Population health initiatives focusing on PH prevention, detection and management in SSA are warranted.

## A090 Combination Artificial Intelligence‐Machine Learning to Automate Pulmonary Hypertension Diagnosis: A Real‐World Evidence Cohort Study

Dr. Seyed M. Shams^1,2^, Dr. Mary E Maldarelli^1,3^, Dr. Colleen M. Ennett^1,2^, Prof. Dr. Bradley A Maron^1,4^, Dr. Katarina Zeder^1,3^

^1^University of Maryland Institute for Health Computing, North Bethesda, USA, ^2^University of Maryland Medical System, Enterprise Data & Analytics, Baltimore, USA, ^3^University of Maryland, School of Medicine, Division of Respiratory, Critical Care and Sleep Medicine, Baltimore, USA, ^4^University of Maryland, School of Medicine, Department of Medicine, Baltimore, USA

Pulmonary hypertension (PH) is a highly morbid disease and underdiagnosis is common outside of expert referral centers. Automated PH diagnosis using artificial intelligence (AI) in large healthcare systems may solve this problem. Analysis of patient‐level right heart catheterization (RHC) data is required but has not been reported previously. We performed a retrospective cohort analysis of all RHC studies (1/1/2016‐12/31/2024) performed at the University of Maryland Medical System, a Maryland statewide clinical network of 12 hospitals serving > 2 million patients. We developed an automated large language model (LLM)‐driven Pattern Repository method, featuring three task‐specific LLM agents for extracting unstructured RHC data, which was manually cross‐validated independently by two PH experts. To address data missingness, we used machine‐learning (ML) to develop formulae to calculate mean pulmonary artery pressure (mPAP) from systolic (sPAP) and/or diastolic (dPAP) PAP, using an 80/20 train‐test split. The study cohort included N = 11,029 unique patients and N = 17,292 RHC reports (age 66 ± 13.5 years; 43% female; 65% White, 30% Black/African American; mPAP, 28 ± 11 mmHg; 26% congestive heart failure). The precision for accurate mPAP, sPAP, and dPAP extraction by the LLM was 99.6%, 99.4%, and 99.4%, respectively, with a detection failure of 0.4%. A missing mPAP was noted in N = 507 patients (4.6%). When applying ML, the simple, linear equation: mPAP=1.51 + 0.43*sPAP+0.45*dPAP returned the highest R2 of 0.94 and lowest mean square error of 8.3 mmHg, which outperformed linear equations used currently (all p < 0.001). The ML‐derived formula identified N = 382 patients (75.3%) out of the 507 patients with mPAP> 20 mmHg, and reclassified patients from no diagnosis to a diagnosis of PH. We combined AI‐ML to extract and interpret RHC and automate PH diagnosis in a large and heterogenous patient population. This approach is an efficient and scalable solution to preventing under‐diagnosis of PH, particularly in areas with lack of access to PH expert care.

## A091 GLP‐1R Agonism Reprograms Mitochondrial Function and Proteomic Signatures in IPAH‐Derived Adventitial Fibroblasts

Mr Yen Cheng Chen^1^, Chien‐Nien Chen^1^, Chris Rhodes^1^, Min Fang^2^, Lan Zhao^1^

^1^NHLI, Imperial College london, London, UK, ^2^Analytical and Biological Sciences, Medicines and Healthcare Products Regulatory Agency, London, UK

Pulmonary arterial hypertension (PAH) is a progressive vascular disease characterised by aberrant cell proliferation, inflammation and metabolic dysfunction. Emerging evidence implicates disrupted glycoprotein metabolism and activation of the AGE–RAGE axis in PAH pathogenesis. Semaglutide, a glucagon‐like peptide‐1 receptor (GLP‐1R) agonist with proven cardiovascular and metabolic benefits, has potential as a disease‐modifying therapy in PAH. In this study, semaglutide treatment selectively inhibited pathological hyperproliferation in pulmonary arterial adventitial fibroblasts (PAAFs) derived from idiopathic PAH patients, without affecting donor‐derived cells. Global proteomic profiling revealed that semaglutide reprogrammed IPAH PAAFs proteome toward a donor‐like phenotype. Differential expression analysis identified 154 semaglutide‐regulated proteins, including key mediators of mitochondrial metabolism and signaling (e.g., GNB1, GSTM3, MGST1, PPP3CA, ADRM1, RAB35). Pathway enrichment highlighted modulation of oxidative phosphorylation, glycolysis/gluconeogenesis, amino acid metabolism, and apelin signaling. These findings indicate that semaglutide not only attenuates pathological fibroblast proliferation but also restores metabolic and proteomic homeostasis, supporting GLP‐1R agonism as a promising therapeutic strategy targeting vascular remodeling and metabolic dysfunction in PAH. We gratefully acknowledge that PAAFs were kindly provided by Prof. Soni Savai Pullamsetti (Max Planck Institute for Heart and Lung Research, Giessen, Germany).

## A092 Features of Right Heart Remodeling and Effects of Comprehensive Therapy in Patients with Pulmonary Hypertension

Rakhimova D.A., Alyavi A.L., Khalilova Kh.M., Sabirzhanova Z.T.

State Institution “Republican Specialized Scientific and Practical Medical Center of Therapy and Medical Rehabilitation”, TSMU, TR PCC

Abstract Dilorom Rahimova^1^

^1^Center Of Therapy, Tashkent citi, Uzbekistan

The study focuses on the assessment of endothelial function of peripheral vessels and central hemodynamics in patients with chronic obstructive pulmonary disease (COPD) and bronchial asthma complicated by pulmonary hypertension (PH). To evaluate the impact of comprehensive therapy on indicators of endothelial function in peripheral vessels. A total of 46 patients with chronic obstructive pulmonary disease (COPD) (Group 1) and 12 patients with bronchial asthma (BA) complicated by the development of cor pulmonale(Group 2) were examined. All patients were diagnosed with pulmonary hypertension (mean pulmonary arterial pressure > 25 mmHg).

For 10 days, patients received amlodipine tablets at a daily dose of 5–10 mg as part of standard therapy (according to GOLD and GINA guidelines, 2023), along with ozone therapy in the form of intravenous administration of ozonated saline solution (ozone concentration: 1000 mcg/L). Before treatment, significant endothelial dysfunction was observed, characterized by an increase in the total synthesis of nitric oxide metabolites (NOx). In Group 2, this hyperproduction was 14% lower compared to Group 1. Assessment of central hemodynamic parameters revealed signs of impaired diastolic function of the right ventricle, with a reduction in the early‐to‐late ventricular filling ratio. Furthermore, all patients demonstrated elevated mean pulmonary arterial pressure, with statistically significantly higher values observed in Group 1. Follow‐up Doppler echocardiography revealed a decrease in systolic pulmonary arterial pressure in both groups: by 7.3% in Group 1 and by 8.8% in Group 2. Additionally, the early‐to‐late filling ratio of the right ventricle increased by factors of 1.07 and 1.08, respectively (p < 0.05). It can be concluded that in patients with bronchial asthma complicated by cor pulmonale and pulmonary hypertension, the hyperproduction of stable NO metabolites is moderately expressed compared to patients with COPD complicated by corpulmonale and pulmonary hypertension.

## A093 Assessment of Pulmonary Hemodynamics and Endothelial Function in Patients With Chronic Obstructive Pulmonary Disease

Alyavi A.L., Rakhimova D.A., Аliahunova M. Yu., Khalilova Kh.M.

State Institution “Republican Specialized Scientific and Practical Medical Center of Therapy and Medical Rehabilitation”, TR PCC. Tashkent, Uzbekistan

Abstract Dilorom Rahimova^1^

^1^Center Of Therapy

To investigate the state of central and pulmonary hemodynamics as well as endothelial function (EF) in patients with chronic obstructive pulmonary disease (COPD). Patients with signs of right ventricular (RV) hypertrophy and dilation, confirmed by Doppler echocardiography, were divided into two groups:

• Group 1 – 16 patients with right ventricular dilation (RVD) (anterior RV wall thickness < 5 mm, anteroposterior RV diameter > 2.5 cm);

• Group 2 – 18 patients with right ventricular hypertrophy (RVH) (anterior RV wall thickness > 5 mm, anteroposterior RV diameter > 2.5 cm).

A control group of 20 healthy individuals was also included. Doppler echocardiographic assessment of structural and functional parameters of the right ventricle, pulmonary hemodynamics, and measurement of stable nitric oxide metabolites (NOx) levels in blood plasma were conducted.

Signs of endothelial dysfunction were identified in 97.4% of patients with right ventricular dilation (RVD) and in all patients with right ventricular hypertrophy (RVH). Moderate negative correlations were observed between the plasma level of stable nitric oxide metabolites (NOx) and mean pulmonary artery pressure. In Group 2 patients, a clear tendency toward a more pronounced reduction in right ventricular ejection fraction was noted. Simultaneous alterations were observed in parameters of diastolic filling and active relaxation function of the right ventricle. Systolic pulmonary artery pressure values exceeded the normal range in nearly half of the patients. The degree of pulmonary hemodynamic impairment, hemodynamic load on the right heart chambers, and the increase in right ventricular size and wall thickness in COPD patients are directly related to the severity of systemic endothelial dysfunction and the duration of the disease.

## A094 Psychosocial Factors and Risk of Venous Thromboembolism: A Cohort and Mendelian Randomization Study Based on the UK Biobank

Dr Bingzhang Zou^1,2^, Dr Zhu Zhang^1^, Dr Zhenguo Zhai^1^, Dr Chen Wang^1^

^1^National Center For Respiratory Medicine, China‐japan Friendship Hospital, Beijing, China, ^2^Chinese Academy Of Medical Sciences & Peking Union Medical College, Beijing, China

The burden of venous thromboembolism (VTE) is rising, yet its causal links with psychosocial factors and underlying biological pathways remain unclear. While psychological distress is known to affect cardiovascular disease, evidence for VTE is limited. This study used UK Biobank (UKB) data to examine psychosocial correlates of VTE and explore inflammatory mechanisms. Among 501,981 UKB participants (excluding 3,951 baseline VTE cases), 23 psychosocial variables were analyzed as exposures during a median 15.5‐year follow‐up. VTE events were identified via hospital ICD codes. Associations were tested using Cox models (adjusted for age, sex, and center) in discovery and validation cohorts, with Bonferroni correction. Mendelian randomization (MR) assessed causality for significant exposures using UKB genetic instruments and FinnGen VTE data via inverse‐variance weighted (IVW) estimates. Proteomic data (UKB‐PPP, n = 53,002) identified differential plasma proteins (Bonferroni P < 0.05) using limma, with Reactome enrichment and bootstrap mediation analysis (1,000 iterations). Twelve psychosocial factors showed significant associations with VTE. Protective factors included frequent social visits (HR = 0.75), confiding support (HR = 0.87), and leisure participation (HR = 0.88). Risk factors included mood swings (HR = 1.18), helplessness (HR = 1.15), loneliness (HR = 1.29), and seeking medical help for anxiety/depression (HR = 1.19). MR confirmed causal effects of loss of enthusiasm and anxiety/depression‐related consultation on VTE (IVW P < 0.01). Proteomics identified 450 VTE‐associated proteins enriched in IL‐6, TNF and platelet degranulation pathways. Inflammatory pathways mediated 39.6% of the effect of loss of enthusiasm and 35.4% of that of anxiety/depression on VTE. This study demonstrates that negative psychological states, including loss of enthusiasm and anxiety/depression, causally promote VTE by activating inflammatory pathways, which mediate 35‐40% of the total effect. These findings highlight a novel psycho‐immune mechanism in VTE pathogenesis and suggest that primary prevention could integrate psychological support with anti‐inflammatory strategies. Further research is warranted to validate the clinical translatability of these targets.

## A095 Generation and Application of Synthetic 3D Velocity Data to Simulate Patient‐Specific Hemodynamics in Pulmonary Hypertension

Dr Chloe Armour^1,2^, Dr Kaihong Wang^2^, Dr Deepa Gopalan^3^, Mr Ben Statton^4^, Prof Declan P O'Regan^4^, Prof Luke Howard^3^, Prof Martin R Wilkins^1^, Prof Xiao Yun Xu^2^, Prof Allan Lawrie^1^

^1^National Heart and Lung Institute, Imperial College London, London, United Kingdom, ^2^Department of Chemical Engineering, Imperial College London, London, United Kingdom, ^3^National Pulmonary Hypertension Service, Imperial College Healthcare NHS Trust, London, United Kingdom, ^4^Medical Research Council, Laboratory of Medical Sciences, Imperial College London, London, United Kingdom

Pulmonary hypertension (PH) is characterized by complex hemodynamic alterations, where wall shear stress (WSS) and vorticity have emerged as key predictors of pulmonary artery pressure. Primary hemodynamic parameters can be evaluated directly from 4D‐flow MRI, and computational fluid dynamics (CFD) models allow for further derivative hemodynamics to be simulated. However, accurate simulation results are dependent on inlet velocity data. This would ideally be extracted from 4D‐flow MRI, yet 4D‐flow MRI remains limited in clinical availability due to cost and technical limitations. To address this gap, we applied our previously validated methodology, developed in studies of ascending aortic aneurysm and type B aortic dissection, to PH cohorts. This enabled the generation of synthetic 3D inlet velocity data for CFD simulations, offering a practical and physiologically accurate alternative when 4D‐flow MRI data are unavailable. We analysed 2 cohorts based on disease aetiology: 30 group 1 pulmonary arterial hypertension patients and 33 group 2‐5 PH patients. Pulmonary flow dynamics were compared between cohorts, and over 400 synthetic inlet profiles were generated. The synthetic profiles were integrated into CFD models to assess their ability to replicate key hemodynamic metrics. Our results demonstrate that synthetic inlet profiles can effectively approximate flow conditions in the absence of 4D flow data, preserving critical features such as WSS distribution and vorticity patterns. This highlights the potential of data‐driven synthetic modelling to expand CFD applications in PH, particularly in resource‐limited settings. Future efforts will focus on refining profile generation algorithms and validating clinical correlations with invasive pressure measurements. This study contributes to our wider project of utilizing CFD models to personalize patient care and improve early diagnosis, risk stratification, and clinical management in individual PH patients.

## A096 Quantification of Emphysema on Chest Computed Tomography Informs COPD‐PH Phenotyping

Dr. Shelsey Johnson^1^, Dr. Raul San Jose Estepar, Dr. Bradley Wertheim, Dr. Pietro Nardelli, Dr. Katarina Zeder, Dr. Gabor Kovacs, Dr. George Washko, Dr. Farbod Rahaghi

^1^Division of Pulmonary and Critical Care Medicine, Brigham And Women's Hospital, Boston, United States, ^2^Department of Radiology, Brigham and Women's Hospital, Boston, United States, ^3^Division of Pulmonary and Critical Care Medicine, Brigham And Women's Hospital, Boston, United States, ^4^Department of Radiology, Brigham and Women's Hospital, Boston, United States, ^5^University of Maryland Institute for Health Computing, North Bethesda, United States, ^6^Division of Respiratory Medicine, Graz, Austria, ^7^Division of Pulmonary and Critical Care Medicine, Brigham And Women's Hospital, Boston, United States, ^8^Division of Pulmonary and Critical Care Medicine, University of Los Angeles, Lost Angeles, United States

Recent guidelines highlight the necessity of quantitative chest computed tomography (CT) data for phenotyping of PH associated with Chronic Obstructive Pulmonary Disease (COPD‐PH). We hypothesized that CT measured emphysema would vary widely in people with COPD‐PH but that more homogenous tissue destruction would be associated with more severe PH. Single center retrospective cohort study of COPD patients referred for lung transplantation. Right heart catheterization (RHC) and chest CT were performed within 1‐year of transplant. Non‐contrast enhanced CT images were analyzed using the Chest Imaging Platform; percentage of emphysema (defined as low attenuation area at ‐950 Hounsfield units, %LAA‐950) was calculated for each lobe. Within‐subject standard deviation (SD) of emphysema across six regions and upper‐to‐lower lobe ratios were calculated. Spatial heterogeneity was measured as the within‐subject %LAA‐950 SD across six lobes. From 2005 to 2025, 23 people with COPD requiring lung transplantation, all with RHC‐confirmed PH (mean pulmonary arterial pressure (mPAP) > 20 mmHg), were analyzed. Of those with pre‐ or combined pre‐and post‐capillary PH (n = 20 (87%)), median [IQR] mPAP, PVR and PCWP were 33 mmHg [6.5], 3.1 Wood units (WU) [1.7] and 14.5 mmHg [7.0], respectively. There was a wide range of emphysematous involvement; median SD [Q1,Q3] of %LAA‐950 across six lung regions was 8.7% [5.7,13.9]. The upper‐to‐lower lobe ratio (SDupper/SDlower) was 0.65 [0.3,0.9]. Neither metric was significantly correlated with PVR. However, there was an inverse relationship between emphysema heterogeneity and PVR; increasing tertiles of within‐patient SD of %LAA‐950 were associated with decreased PVR (4.2 to 3.2 to 2.7 WU; p = 0.08). In a COPD cohort requiring lung transplantation with confirmed PH, emphysematous destruction was widely variable. In subjects with more homogeneous involvement, we observed a directionally higher PVR. These data provide preliminary evidence supporting the role of quantitative CT data to inform COPD subgroups at greater risk for severe PH.

## A097 Gelsolin Regulates Phenotypic Switching of Pulmonary Artery Smooth Muscle Cells in Chronic Thromboembolic Pulmonary Hypertension

Yuhan Li^1,2^, Yunxia Zhang^1^, Zhenguo Zhai^1^

^1^National Center for Respiratory Medicine, China‐Japan Friendship Hospital, Beijing, China, ^2^Chinese Academy of Medical Sciences & Peking Union Medical College, Beijing, China

To investigate the expression pattern of Gelsolin in pulmonary artery tissues and pulmonary artery smooth muscle cells (PASMCs) from patients with chronic thromboembolic pulmonary hypertension (CTEPH), and to determine its influence on pulmonary vascular remodeling. Single‐cell RNA sequencing of pulmonary endarterectomy (PEA) tissues from CTEPH patients defined cellular composition and transcriptional profiles. Gelsolin expression and localization were analyzed by immunohistochemistry, immunofluorescence, and Western blot in lung tissues from four CTEPH patients after PEA and three healthy donors, as well as in primary PASMCs from both groups. Lentiviral transfection achieved Gelsolin knockdown or overexpression in PASMCs. PASMC migration, proliferation, and apoptosis were assessed by scratch/Transwell assays, EdU staining, and Annexin V‐APC/PI flow cytometry, respectively. Transcriptome sequencing after Gelsolin knockdown identified downstream genes and pathways validated by real‐time PCR and Western blot. Single‐cell transcriptomics identified three PASMC subtypes within CTEPH lesions—contractile, synthetic, and mixed types. KLF4, a marker of phenotypic switching, was upregulated in CTEPH PASMCs. Gelsolin expression was reduced in PEA tissues compared with pulmonary arteries from healthy donors and mainly localized to the smooth muscle‐rich vascular layer. Western blot confirmed markedly decreased Gelsolin levels in CTEPH‐derived PASMCs relative to healthy cells. These findings indicate that PASMCs in CTEPH undergo phenotypic transformation accompanied by reduced Gelsolin expression. Gelsolin silencing promoted PASMC proliferation and migration while inhibiting apoptosis, whereas its overexpression produced the opposite effects. RNA sequencing of PASMCs after Gelsolin knockdown revealed enrichment of cell adhesion, cytoskeletal regulation and PI3K‐Akt signaling genes. Gelsolin knockdown upregulated KLF4, a marker of phenotypic switching, and downregulated the contractile marker SM22a, as confirmed by real‐time PCR and Western blot.

In CTEPH, reduced Gelsolin expression in PASMCs promotes proliferation and migration, inhibits apoptosis, and drives pulmonary vascular remodeling. Gelsolin regulates PASMC phenotypic switching and may represent a therapeutic target for CTEPH.

## A098 Deep Phenotyping of Circulating Immune Cells in Pulmonary Arterial Hypertension Using Mass Cytometry

Assistant Professor Sasha Prisco^1^, Benjamin Kremer, Megan Larson, Dr. Martin Felices, Dr. Patricia Azevedo De Lima, Rashmi Raveendran, Joseph Johnson, Dr. Weston Upchurch, Professor E. Kenneth Weir, Professor Stephen Archer, Professor Thenappan Thenappan

^1^University Of Minnesota, Minneapolis, USA

Pulmonary arterial hypertension (PAH) involves perivascular macrophage accumulation that promotes chronic inflammation, which drives endothelial dysfunction and proliferation of smooth muscle cells and fibroblasts. These macrophages arise from recruited circulating monocytes rather than tissue‐resident macrophages. However, circulating immune cells in PAH remain poorly characterized. Comprehensively profile circulating immune cells in PAH patients and healthy controls using mass cytometry (CyTOF). We studied 11 PAH patients and 11 healthy controls. Peripheral blood mononuclear cells were analyzed by CyTOF. Demographics, comorbidities, six‐minute walk distance, NT‐proBNP, emPHAsis‐10 scores, and echocardiographic data were collected. Cell subset identification, clustering, and visualization were performed using the Astrolabe Cytometry Platform. Spearman correlation was used to assess relationships between immune cell abundance and disease severity. Whole blood from monocrotaline‐treated rats was analyzed by flow cytometry. Mean age was 49.6 ± 15.1 years, and 63.6% were female. Although overall immune cell abundance was similar between PAH and control, PAH patients had reduced non‐classical monocytes (FDR = 0.007). Subset analysis using 31 surface markers showed significantly lower levels of non‐classical monocytes expressing integrin (CD14‐CD16 + CD11c^hi CD66b^lo CD38^lo; p = 0.001, FDR = 0.059), pro‐inflammatory classical monocytes (CD14 + CD16‐CD123 IL3R^lo CD66b^lo CD28^lo; p = 0.002, FDR = 0.059), activated cytotoxic NK cells (CD56 + CD16 + CD57^hi CD20^hi; p = 0.001, FDR = 0.059), double‐positive T cells with high CCR4 expression (CD4 + CD8 + CD57^lo CD45RO^lo CCR4^hi; p = 0.002, FDR = 0.059), and late‐differentiated CD8+ effector memory cells with high CCR4 expression (CD8 + CD57^hi CD161^lo CCR4^hi; p < 0.001, FDR = 0.059) in PAH patients versus controls. Similarly, monocrotaline‐treated rats had less circulating non‐classical monocytes (CD43^hi His48^lo) compared to controls, with reductions of 10.7% (p = 0.09) and 18.9% (p = 0.005) at 20 and 24 days, respectively. There was no correlation between circulating immune cell numbers and severity of PAH, as assessed by REVEAL score, and right ventricular (RV) function. PAH patients have reduced circulating monocytes, NK cells, and T cells, likely reflecting their enhanced outmigration into perivascular lung tissue and RV.

## A099 Effect of High Dose Inhaled Treprostinil On Mortality In Patients Enrolled in the Open‐Label Extension of the INCREASE Trial

Oksana Shlobin^1^, Abubakr Bajwa^2^, Chunqin Deng^3^, Kyle Davis^3^, Meredith Broderick^3^, Sandeep Sahay^4^

^1^Inova Fairfax, Falls Church, United States, ^2^Mayo Clinic, Jacksonville, United States, ^3^United Therapeutics, Durham, United States, ^4^Houston Methodist Hospital, Houston, United States

In the 16‐week randomized, placebo‐controlled INCREASE study, treatment with inhaled treprostinil (iTRE) demonstrated a significant improvement in 6MWD among patients with PH‐ILD. Prespecified subgroup analysis showed greater improvements in 6MWD in patients achieving higher doses of iTRE. This analysis further explored the relationship between doses achieved and outcomes of patients enrolled in the open‐label extension (OLE). The primary objective of the OLE was to provide iTRE to eligible patients enrolled in the INCREASE trial. Secondary objectives aimed to assess the long‐term safety and efficacy of the drug. Patients enrolled in the OLE were initiated on iTRE at a dose of 3 breaths per session (bps) four times daily, which could then be titrated to a maximum dose of 15 bps four times daily. Here, we analyzed patients assigned iTRE in INCREASE who continued iTRE in the OLE. Outcomes between the following three groups were compared: ≤ 9, 10‐14, and ≥ 15 bps.

One hundred nineteen patients received iTRE in INCREASE, entered the OLE, and continued iTRE (≤ 9 bps: n = 34; 10‐14 bps: n = 53; ≥ 15 bps: n = 32). Of these, patients who achieved ≥ 15 bps had fewer deaths (≤ 9 bps: 9 (26.5%); 10‐14 bps: 13 (24.5%); ≥ 15 bps: 7 (21.9%)) and greater time to death (≤ 9 bps: 53 (12.6, 117.0) weeks; 10‐14 bps: 62 (4.9, 106.7) weeks; ≥ 15 bps: 79.6 (28.3, 95.4) weeks) when compared to the other groups. s: Patients receiving higher doses of iTRE experienced numerically fewer deaths and had a longer time to demise than those on lower doses in the OLE portion of the INCREASE trial. These findings warrant validation with a larger controlled dataset.

Sponsored by United Therapeutics

## A100 Precision‐Cut Lung Slice Co‐Culture Models for Investigating Macrophage‐Mediated Inflammation in Pulmonary Hypertension

Instructor Hui Zhang^1^, B Alexandre McKeon^1^, Ram Raj Prasad^1^, Sushil Kumar^1^, Suzette Riddle^1^, Kurt Stenmark^1^

^1^University Of Colorado, Aurora, USA

Inflammation drives pulmonary vascular remodeling in pulmonary hypertension (PH), with macrophage accumulation forming a key proinflammatory niche. How macrophages are recruited and sustain inflammation remains unclear. While precision‐cut lung slices (PCLS) preserve native lung architecture, conventional models lack immune infiltration. To address this limitation, we established novel PCLS ‐ immune cell co‐culture systems that incorporate macrophage recruitment and activation, providing an ex vivo platform for mechanistic investigation of immune‐mediated vascular inflammation in PH. PCLS from calves and/or humans with PH (PH‐PCLS) and healthy controls (CO‐PCLS) were used as ex vivo model systems. CO‐PCLS were exposed to hypoxia alone, hypoxia combined with the pro‐inflammatory cytokine IL‐1β, or co‐cultured with bone marrow derived macrophages (BMDMs) isolated from PH calves, using a transwell system (8 µm). Immunofluorescence staining followed by Z‐stack confocal imaging was performed to evaluate macrophage recruitment within the PCLS, with BMDMs labeled using CMFDA (green). Additionally, transmission electron microscopy (TEM) was employed to identify and characterize spatiotemporal localization of extracellular vesicles (EVs), key mediators of intercellular communication within the pulmonary vasculature in PH. PH‐PCLS exhibited increased expression of proinflammatory genes (e.g., IL‐6, CXCL12) compared to CO‐PCLS. Exposure of CO‐PCLS to hypoxia (1.5% O2) combined with IL‐1β or co‐culture with BMDMs (not hypoxia alone) for 48 hours induced PH‐associated inflammatory responses, as indicated by increased expression of IL‐6 and CXCL12. We also found that hypoxia enhanced BMDM infiltration into the PCLS, consistent with the gene expression results. Furthermore, TEM analysis of PCLS revealed the ultrastructure of the distal pulmonary artery, showing EVs enriched in the adventitial region and positioned near vascular cells such as fibroblasts and macrophages, suggesting potential EV‐mediated interactions among adventitial cells. These PCLS immune co‐culture models provide a platform to study macrophage‐mediated inflammation and offer potential for developing immune cell‐targeted therapeutic strategies in PH.

## A101 Incidence and Impact of Cardiac Arrhythmias on Hospitalization in Pulmonary Hypertension: A Retrospective Cohort Study From the VA‐CART Program

MD Abdulkadir Kiris^1,2,3^, Matthew Borgia^1,2,3^, PhD Melih Agraz^1,2,3^, MD Matthew Jankowich^1,3^, MD Wen‐Chih Wu^1,2,3^, MD Stephen W. Waldo^4,5,6^, PhD Mary E Plomondon^4,5,6^, MD Peng Zhang^1,2,3^, MD Gaurav Choudhary^1,2,3^

^1^Providence Veteran Affairs Medical Center, Providence, United States, ^2^Cardiovascular Research Center, Brown University, Providence, United States, ^3^Department of Medicine, Warren Alpert Medical School of Brown University, Providence, United States, ^4^CART Program, Office of Quality and Patient Safety Veteran Health Administration, Washington DC, United States, ^5^Rocky Mountain Regional Veterans Affairs Medical Center, Aurora, United States, ^6^Division of Cardiology, Department of Medicine University of Colorado, Aurora, United States

Right heart remodeling associated with pulmonary hypertension (PH) are expected to increase risk of arrhythmias. However, the extent to which PH and its hemodynamic subgroups influence supraventricular (SVA) and ventricular (VA) arrhythmia incidence and arrhythmia‐related clinical deterioration remains unknown. To determine the incidence of SVA and VA in a large PH cohort, and to evaluate the relationship of mean pulmonary artery pressure (mPAP) and PH subgroups on arrhythmia development, and arrhythmia‐related clinical deterioration requiring hospitalization.

Methods: In this cohort study, we included a total of 40,855 patients who underwent right heart catheterization between January 2014 and December 2019 from the Veteran Affairs Clinical Assessment, Reporting, and Tracking Program. After exclusion of patients with a prior arrhythmia diagnosis within the preceding 10 years, a final study population of 24,369 individuals were followed for up to 5 years. The primary outcome was the incidence of SVA and VA in the overall PH population (mPAP> 20 mmHg) and across PH subgroups (precapillary, postcapillary and combined). The secondary outcome was clinical deterioration requiring hospitalization attributable to SVA and VA.

17,654 patients had PH and 6,715 without PH served as controls. The incidence rate of SVAs and VAs was significantly higher in the PH group (per 100 person‐year: 13.09 vs 8.63 and 3.29 vs 1.93, respectively), with atrial fibrillation and flutter being more common. All PH hemodynamic subgroups showed elevated SVA and VA incidence, with the combined subgroup having the highest rates (16.11 and 4.48/100 person‐years, respectively). SVA‐related hospitalization was increased across all subgroups, while only those with combined PH had a significantly higher hazard rate of VA‐related hospitalization. PH, particularly the combined subgroup, is strongly associated with both SVAs and VAs and related hospitalizations, underscoring the need for arrhythmia screening and targeted management to improve clinical outcomes.

## A102 Addressing Common Challenges in PAH Risk Score Calculation, a Statistician's Perspective

Dr Dana Cella^1^, ChunQin Deng^1^, Youlan Rao^1^, Andrew Nelsen^1^, Derek Solum^1^

^1^United Therapeutics Corp, Research Triangle Park, United States

Risk score calculators such as REVEAL 2.0, REVEAL Lite 2, COMPERA 2.0, and the Non‐invasive French Criteria (i.e., functional class I or II, 6‐minute walk test > 440 m, and NT‐proBNP < 300 ng/L) are widely used in the management of pulmonary arterial hypertension (PAH). These tools are used to evaluate a patient's baseline mortality risk, to monitor their prognosis over time, and to inform treatment decisions. Although conceptually straightforward—assigning points to clinical parameters and combining them into a total score—their practical application raises methodological challenges.

From a statistician's perspective, we outline key considerations in the implementation of PAH risk scores as clinical trial endpoints. Specific challenges include handling missing data (when only a subset of parameters is available) and aligning the timing of parameter collection (whether all inputs must be obtained on the same date). The appropriate approach depends on the intended use of the risk score. For example, limited missing data may be acceptable for estimating an individual's prognosis, but stricter requirements are needed for longitudinal assessments or when risk score change is used as a clinical trial endpoint. Risk scores can be used to evaluate not only mortality and clinical worsening, but also clinical improvement. We provide practical guidance to promote rigorous and statistically sound use of PAH risk calculators in both routine care and clinical research.

## A103 Excellent Care Engagement and Improved Exercise Tolerance in a Collaborative Care Model for Methamphetamine Associated Pulmonary Arterial Hypertension

Ms. Dominique Ingram^1,3^, Ms. Laura Stolebarger^2^, Mr. Ulises Amaton^2^, Dr. Michael Incze^2^, Assistant Professor In Cardiology Katharine Clapham^1^

^1^Division of Cardiovascular Medicine, University of Utah, Salt Lake City, United States, ^2^Division of General Internal Medicine, University of Utah, Salt Lake City, United States, ^3^College of Pharmacy, University of Utah, Salt Lake City, United States

Methamphetamine is a synthetic stimulant and rapidly emerging public health concern in the United States that can result in severe cardiopulmonary sequelae such as pulmonary arterial hypertension. Collaborative care models that include co‐located addiction treatment, care management, and specialty care may help lead to improved care engagement and outcomes for individuals with methamphetamine associated pulmonary arterial hypertension (MA‐PAH). To evaluate a collaborative care model developed between the pulmonary hypertension clinic and an integrated primary care/addiction medicine clinic to enhance care and support for participants with MA‐PAH.

Investigators at the University of Utah's pulmonary hypertension and integrated primary care/addiction medicine clinic developed a collaborative care model for patients diagnosed with MA‐PAH. Patients were enrolled in a 12‐week cardiac rehabilitation program. Participants met with a contingency management team during these visits during which they had the opportunity to draw for the potential of winning a gift card. Patient surveys and clinical data were collected at baseline and completion. Seven participants (mean age 54.3 ± 7.4 years; 57.1% female) were enrolled, with five (71.4%) reporting stable housing. Five participants completed the program with endpoint data available. The average change in METs from baseline to final cardiac rehabilitation assessment was 0.568 (±1.57). The average visit attendance was 86% (±15.9%). All participants reported a decrease in methamphetamine use. There was no significant change in NT‐proBNP or echocardiographic tricuspid annular plane systolic excursion (TAPSE) values. Preliminary findings demonstrate that a collaborative multidisciplinary approach including cardiac rehabilitation and contingency management is feasible for patients with MA‐PAH. Participants reported reduction in methamphetamine use, maintained high visit attendance, and exhibited stable cardiac function over three months. These results suggest that integrated care models may improve engagement and exercise tolerance among individuals with MA‐PAH, supporting further evaluation in larger cohorts.

## A104 Pulmonary Hypertension in Children Living at High Altitude. Special Reference to Andean Population

Professor Gabriel Diaz^1^, PEDIATRIC CARDIOLOGIST ALICIA MARQUEZ^2^, Postdoctoral Research Fellow Carlos Eduardo Diaz^3^

^1^Universidad Nacional De Colombia, BOGOTÁ, COLOMBIA, ^2^Centro Policlínico Del Olaya, Clínica De La Mujer, Bogotá, Colombia, ^3^University of Pittsburgh, Pittsburgh, United State

Pulmonary Hypertension (PH) in Children at high altitude has special characteristics by the effect of hypobaric hypoxia. Globally, 140 million people live above 2.500 MASL, with 35‐40 million residing in the Andean region: Colombia, Ecuador, Peru and Bolivia. We considered important to know with precision the current Andean population. We analyzed the Andean Population through official registries of the four countries and a cohort of 85 children with PH, living in Bogota 2.640 MASL and undergoing long‐term follow‐up. The study assessed etiology, severity, outcomes and the role of the prolonged hyperoxia test (O2Test: FIO2 > 80% for up to 24 hours) in evaluating pulmonary vascular reactivity (PVR). The last official Registries were made in 2017, with important under‐registry. The higher Andean population above 2.500 MASL is in Colombia, and Bogotá its capital has the higher population in the world (Table 1‐2). The primary causes of PH were idiopathic and PH following CHD correction. The mean of mPAP and PR were: 69.2 mmHg and 15.8 WU. Among the 10 longest‐surviving patients, the mean survival was 22.8 years. Overall mortality was 29.4% (25 patients), with a lower mortality rate in those diagnosed before 3 years old. The O₂Test was negative in 79.2% (19/24) of deceased patients, compared to 22.9% (11/48) of survivors, showing significant difference (p = 0.000). Andean Population is important (28.57% of world population living above 2500 m). PH in children living at high altitude requires greater attention due to its prevalence, severity and the importance of PVR induced by hypobaric hypoxia. While mortality is lower in younger patients, severe pulmonary vascular disease can develop before 3 years old., including newborns. The O2Test is a valuable tool for assessing PVR at altitude and serves as a predictor of survival, emphasizing the importance of early detection and intervention.

## A105 Hemodynamic Parameters in Predicting Survival in Pulmonary Arterial Hypertension

Md Manreet Kanwar^1^, Charles Fauvel, Jason Weatherald, Thomas Cascino, Sandeep Sahay, Angela Toepp, Raymond Benza

^1^University Of Chicago, Chicago, USA

Hemodynamics are relevant when clinically assessing patients with PAH, especially for estimating prognosis. Yet, data on their prognostic implications remain inconsistent and controversial. A harmonized dataset was constructed from 7 randomized controlled trials obtained from the US Food and Drug Administration (FDA): AMBITION, ARIES1/2, FREEDOM, GRIPHON, PATENT and SERAPHIN. Hemodynamics were evaluated at baseline and follow‐up (week 16), initially with univariate analysis and then using a backward stepwise variable selection method to assess their impact of outcomes. Primary outcome analyzed was death at 1 year. Cox Proportional Hazards Regression models were used to assess outcomes and AUC to evaluate model fit. Baseline and follow‐up data was available for 3,463 patients. Majority were females (78.7%), with a mean age of 48.1 years, a mean 6MWD of 387 meters and a NYHA functional class II (56.6%) or III (36.6%) at baseline. Mean RA pressure was 8.33‐ and 8.19‐mm Hg, systolic PAP of 82.85‐ and 75.9‐mm Hg, mean PAP of 51.55‐ and 50.0‐mm Hg and cardiac index of 2.48 and 2.6 L/min/m2 at baseline and follow‐up, respectively. Using Cox Proportional Hazards Ration, significant predictors of 1‐year mortality at baseline included systolic BP (HR 0.96, 95% CI 0.93‐0.99, p = 0.02); NYHA FC (HR 1.99, 95% CI 1.11‐3.56, p = 0.01); stroke volume (HR 0.91, 95% CI 0.84‐0.96, p = 0.004), RA/PCWP ratio (HR 1.46, 95% CI 1.02‐2.1, p = 0.03). Significant predictors of 1‐year mortality at follow‐up included RAP (HR 1.11, 95% CI 1.02‐1.22, 95% CI 0.01); stroke volume (HR 0.61, 95% CI 0.43‐0.88, p = 0.007); stroke volume index (HR 1.54, 95% CI 1.08‐2.21, p‐0.01); cardiac efficiency (HR 0,95% CI 0.00‐0.81, p = 0.04). To our knowledge, this is the largest aggregated analysis of hemodynamic parameters in predicting survival in PAH. This analysis will be used to select features for derivation of machine‐learned hemodynamic PAH patient risk stratification model.

## A106 Pregnancy Outcomes in Patients With eisenmenger Syndrome: A Case Series Report

Dr. Ly Nguyen Thi Minh^1,2^, Dr. Hieu Nguyen Lan^1,2^, Prof. Viet Nguyen Lan^1,2^

^1^Hanoi Medical University, Ha Noi, Vietnam, ^2^Hanoi Medical University Hospital, Ha Noi, Vietnam

Eisenmenger syndrome (ES) is characterized by pulmonary arterial hypertension, resulting from high pulmonary vascular resistance, along with a bidirectional or reversed right‐to‐left shunt in patients with congenital heart disease. Pregnancy in patients with ES is associated with high maternal and fetal mortality and is therefore considered a contraindication. However, in clinical practice, pregnant women with ES who choose to continue their pregnancies despite high risks are still occasionally encountered. This study is a prospective follow‐up of three pregnancies in women with ES from April 2024 to March 2025 at Hanoi Medical University Hospital. Three ES patients were presented with large ventricular septal defects and severe pulmonary arterial hypertension. The mean maternal age was 33 years, and all cases were in their first pregnancy. At admission, the gestational ages were 10, 16, and 20 weeks, respectively. The systolic pulmonary pressure was approximately 90 mmHg, and the peripheral oxygen saturation level was about 90%. All patients were managed by a multidisciplinary team consisting of cardiologists and obstetricians, with monthly follow‐up visits and treatment with phosphodiesterase‐5 inhibitors. They were scheduled for admission between 31 and 33 weeks of gestation. Pulmonary surfactant was administered before delivery, and patients underwent cesarean section at 32 and 35 weeks of gestation. Neonatal birth weights were 2000 g, 1600 g, and 1800 g. All patients received intravenous iloprost perioperatively, and each was initiated on an endothelin antagonist (bosentan) immediately postpartum. The newborns achieved Apgar scores ranging from 8 to 9 and received immediate neonatal intensive care. All mothers and infants were discharged home in stable condition without complications. This study highlights the importance of multidisciplinary care and proactive delivery planning as vital factors in achieving successful outcomes for pregnancies in women with ES.

## A107 Dasatinib‐Associated Pulmonary Arterial Hypertension in a Patient With Monoallelic EIF2AK4 Pathogenic Variant

Dr Som Singh^1^, Dr Salil Kumar^2^, Dr Gabriela Chen^3^, Dr Anita Deswal^2^, Dr Saadia Faiz^4^, Dr Prithviraj Bose^5^, Dr Naveen Pemmaraju^5^, Dr Hagop Kantarjian^5^, Dr Hyeon‐ju Ali^2^, Dr Som Singh

^1^Department of Internal Medicine, University of Texas Health Sciences Center at Houston, Houston, USA, ^2^Department of Cardiology, The University of Texas MD Anderson Cancer Center, Houston, Texas, Houston, USA, ^3^Clinical Cancer Genetics, The University of Texas MD Anderson Cancer Center, Houston, USA, ^4^Department of Pulmonary Medicine, The University of Texas MD Anderson Cancer Center, Houston, USA, ^5^Department of Leukemia, The University of Texas MD Anderson Cancer Center, Houston, USA

Pulmonary arterial hypertension (PAH) associated with dasatinib is a rare treatment complication with prior French registry data demonstrating no cases with bone morphogenic protein receptor‐2 (BMPR‐2) mutations. A 26‐year‐old woman with a history of chronic myeloid leukemia was treated with imatinib for 11 years but due to relapse, dasatinib 100 mg daily was initiated. Five months later, she developed dyspnea on exertion and syncope. Transthoracic echocardiogram demonstrated right ventricular enlargement and an elevated right ventricular systolic pressure (50‐55 mmHg). Right heart catheterization (RHC) confirmed PAH pulmonary artery pressure (PAP) 74/35 (50) mmHg, pulmonary capillary wedge pressure (PCWP) 11 mmHg; pulmonary vascular resistance (PVR) 11 Wood units. Other causes of PAH were excluded with ventilation perfusion scan, computed tomography of the chest, and pulmonary function testing. Dasatinib was discontinued and sildenafil 10 mg three times daily was initiated. Due to persistent dyspnea, RHC was repeated a year later, which showed persistent severe PAH (PAP 67/23 (38) mmHg; PCWP 10 mmHg; PVR 8.5 Wood units). Ambrisentan 5 mg daily and selexipag 800 mcg twice daily were initiated with only mild improvement in symptoms. Repeat RHC 6 months later showed improvement in pulmonary pressures (PAP 31/12 (22) mmHg; PCWP 10 mmHg; PVR 2.1 WU). The patient was referred for genetic testing given persistence of PAH. She was found to have a monoallelic mutation in the eukaryotic translation initiation factor 2 alpha kinase 4 (EIF2AK4), consistent with a pathogenic variant for autosomal recessive PAH. This is the first case reported with dasatinib‐associated PAH in a patient with PAH‐associated genetic mutation. Dasatinib may have been a “second hit” that led to PAH in a genetically predisposed patient with heterozygous EIF2AK4 variant status. Future studies are needed to understand the role of genetic mutations in drug‐associated PAH and propose early risk stratification.

## A108 Characterizing Pulmonary Hypertension in a Pediatric Pulmonary Vein Stenosis Population

Mr. Zerubabell Daniel^1^, Dr. Maanasi Mistry^1^, Dr. Kimberlee Gauvreau^1^, Ms. Christina Ireland^1^, Ms. Sydney Moreland^1^, Dr. Mary Mullen^1^, Dr. Kathy Jenkins^1^

^1^Boston Children's Hospital, Boston, United States

Pediatric pulmonary vein stenosis (PVS) is a rare, progressive disease in which there is overlap with features of pulmonary hypertension (PH). We aim to distinguish factors indicating PH secondary to PVS versus those indicating pulmonary arterial hypertension (PAH). This is a retrospective study using the Boston Children's Hospital (BCH) PVS Registry to evaluate PH in pediatric patients with biventricular physiology and multi‐vessel PVS treated at BCH between 2017 and 2024. Demographics, comorbidities, and mean pulmonary artery pressure (mPAP) from cardiac catheterizations were analyzed. Of the eligible 126 patients, 102 had a known date of first catheterization. Of these 102 patients, median age at the time of PVS diagnosis was 5.4 months and an interquartile range of [3.6, 9.9]. The median number of catheterizations was 2 [1, 4]. For PVS diagnosis category, 71 patients (70%) had underlying congenital heart disease, 21 (21%) were premature, 9 (9%) had primary PVS, and 1 (1%) had isolated lung disease. At the time of first catheterization, median age was 9.8 months [6.2, 27.1], elapsed time from PVS diagnosis was 2.8 months [0.3, 9.7], and mPAP was 31 mmHg [26, 44]. Severity of PH at the first catheterization was distributed as follows: 5 (5%) none, 42 (41%) mild, 22 (22%) moderate, 33 (32%) severe. Slope of unadjusted mPAP change in mmHg/year was ‐0.4 and was similar when adjusted for either mPAP at first catheterization (‐0.4) or initial PH category (‐0.5). There was a trend towards more rapid decrease in mPAP from first catheterization in those with severe PAH compared to mild/moderate, with mean decrease 1.4 mmHg/year (p = 0.07). In our cohort, most patients with PVS concurrently had PH. Those with severe PH saw the most improvement in mPAP with ongoing PVS treatment. The intersection between PVS, PH, and PAH requires further study.

## A109 β3‐Adrenergic Receptor Agonists Modulate Endothelial Inflammation and Improve Cardiopulmonary Function in Pulmonary Arterial Hypertension

Ms Laura de la Bastida‐Casero^1^, Ms Yolanda Sierra‐Palomares^1^, Ms Bertha Garcia‐Leon^1^, Ms Lola Navarro‐Llinares^1^, Dr Maria Villalba‐Orero^2,3^, Dr Eduardo Oliver^1,3,4^

^1^Centro de Investigaciones Biológicas Margarita Salas (CIB), CSIC, Madrid, Spain, ^2^Universidad Complutense de Madrid, Madrid, Spain, ^3^Centro Nacional de Investigaciones Cardiovasculares (CNIC), Spain, ^4^Centro de Investigación Biomédica en Red Enfermedades Cardiovasculares (CIBERCV), Spain

Pulmonary arterial hypertension (PAH) is a rare and severe condition characterized by endothelial dysfunction and vascular remodelling, leading to a progressive increase in pulmonary arterial pressure and right ventricular (RV) overload. Over time, RV hypertrophy advances to heart failure and premature death. A hallmark of PAH is an aberrant endothelial dysfunction, where endothelial cells express adhesion molecules, promoting leukocyte recruitment and sustaining vascular inflammation and remodelling. The regulation of endothelial integrity and function becomes essential for a successful therapeutic strategy. In our study, we explored the therapeutic potential of β3‐adrenergic receptor (β3‐AR) agonists—Mirabegron and Vibegron, currently approved for overactive bladder—on hemodynamic, morphological, inflammatory and remodelling markers in PAH. Using a murine model of hypoxia+Sugen‐induced PAH, we observed via western blot and immunofluorescence that β3‐AR agonist treatment reduced the expression of E‐Selectin, VCAM‐1 and α‐SMA in lung tissue. These molecular changes were accompanied by improvements in RV systolic pressure (RVSP) and RV hypertrophy in treated mice. Complementary magnetic resonance imaging revealed a trend toward reduced RV dilatation and improved cardiac function. Additionally, positron emission tomography indicated a trend toward restored cardiac and pulmonary metabolism, supporting a comprehensive therapeutic effect on myocardial and pulmonary function and bioenergetics. These results were in concordance with previous works where we described a protective role of Mirabegron on the pulmonary endothelium in animal models and identified relevant diseases biomarkers with potential diagnostic and prognostic uses in PH patients. Altogether, these findings highlight the potential of β3‐AR agonists as modulators of endothelial dysfunction and inflammation in PH. Additionally, biomarkers of endothelial damage and inflammation could reflect disease severity and guide future precision medicine strategies, supporting the repositioning of β3‐AR agonist as a novel therapeutic approach for PH.

## A110 Quantification of Venous Vascular Structural Changes in PVOD Using CT Imaging

MD Esther J. Nossent^1^, PhD Raul San Jose Estepar^2,3^, MD, PhD Harm Jan Bogaard^1^, PhD Pietro Nardelli^2,3^, MD, PhD Anton Vonk Noordegraaf^1^, PhD Ruben San Jose Estepar^2,3^, MD, PhD Lilian J. Meijboom^4^, MD, PhD Farbod N. Rahaghi^5^

^1^Department of Pulmonary Medicine, Amsterdam UMC, Free University Amsterdam, Amsterdam Cardiovascular Sciences, Amsterdam, the Netherlands, Amsterdam, The Netherlands, ^2^Applied Chest Imaging Laboratory, Brigham and Women's Hospital, Harvard Medical School, Boston, MA, USA., Boston, USA, ^3^Department of Radiology, Brigham and Women's Hospital, Harvard Medical School, Boston, MA, USA., Boston, USA, ^4^Department of Radiology and Nuclear Medicine, Amsterdam UMC, Free University Amsterdam, Cardiovascular Sciences, Amsterdam, the Netherlands, Amsterdam, The Netherlands, ^5^Division of Pulmonary and Critical Care Medicine, UCLA David Geffen School of Medicine, Los Angeles, CA, USA, Los Angeles, USA

Pulmonary veno‐occlusive disase (PVOD) is a rare form of pulmonary arterial hypertension (PAH) characterized by pronounced remodeling of veins, pre‐septal venules, and capillary dilatation/proliferation; patients have poor responses to treatment and a dismal prognosis (1). Early recognition of PVOD is important for clinical decision making. Twenty five PVOD patients diagnosed at the Amsterdam UMC confirmed by histopathology and/or genetic testing (presence of pathogenic EIF2AK4 variants) were included in this study. For each subject, available CT imaging closest to diagnosis was examined and thinnest and least sharp reconstruction kernel was selected for vascular reconstruction. The PVOD cohort was compared to 47 patients with PAH and 37 control comparators selected from a cohort of patients at Brigham and Women's Hospital as previously described (2). Vascular reconstructions, vessel sizing and arterial/venous separation were performed using the Chest Imaging Platform (https://chestimagingplatform.org/). Vascular volumes were measured as a function of cross‐sectional area and compared using Wilcoxon Rank‐Sum Testing with two‐tailed p‐values. Hemodynamic measures were not significantly different between PVOD and PAH patients. Lung volume adjusted vascular volume analysis revealed a higher total venous volume in PVOD as compared to PAH and controls, with this increase driven largely by volume in vessels with less than 10mm2 cross sectional area 18.2[15.0‐20.2], vs PAH 12.4[10.5‐13.5] p < 0.0001 vs control 14.8[13.5‐15.8] p = 0.001. Our analysis revealed higher lung volume adjusted venous volumes in vessels less than 10mm2 in PVOD as compared to PAH patients and controls. This might be consistent with the observation of venous engorgement driven by lymphatic collaterals adjacent to the venous walls in the pulmonary circulation, as previously noted in PVOD. In this exploratory cohort, an increase in the size of this range of venous vessels on CT imaging differentiates PVOD from PAH.

## A111 The Prognostic Value of Treatment‐Associated Changes in the Alveolar‐To‐Arterial Gradient for Oxygen in Patients With Pulmonary Arterial Hypertension and a Lung Phenotype

Dr Andrea Baccelli^1^, Dr Rachel J. Davies^1^, Dr Gulam Haji^1^, Dr Francesco Lo Giudice^1^, Prof Luke S. Howard^1^

^1^National Pulmonary Hypertension Service, Hammersmith Hospital, Imperial College Healthcare Trust, London, United Kingdom

Patients with PAH and a lung phenotype, characterised by a reduced transfer factor for carbon monoxide (TLCO) and a smoking history, display an attenuated response to therapy and poorer survival compared with “classical” PAH. Pulmonary vasodilators may further disrupt ventilation–perfusion matching, worsening arterial oxygenation in these patients. We aimed to evaluate treatment‐related changes in exercise gas exchange and to determine their prognostic value in PAH with and without a lung phenotype. We analysed 178 incident treatment‐naïve patients with PAH (128 classical PAH; 50 PAH lung phenotype). The lung phenotype was defined by a TLCO < 45% and a smoking history ≥ 20 pack‐year. Cox regression models were used to identify independent predictors of survival. Patients with a lung phenotype were older (63 ± 12 vs 51 ± 16 years, p < 0.001) and had lower baseline TLCO (32 ± 8 vs 63 ± 16%pred, p < 0.001) and peak VO₂ (9.3 ± 3.0 vs 12.8 ± 3.9 mL·kg⁻¹·min⁻¹, p < 0.001). After a median follow‐up of 3 months, the A–a gradient showed no significant change in classical PAH but increased significantly in the lung‐phenotype group (+1.2 kPa, 95% CI 0.5 to 2.0; p = 0.035), with an adjusted between‐group difference of +1.7 kPa (95% CI 0.8 to 2.7; p < 0.001). The treatment‐associated change in A–a gradient independently predicted survival in the lung‐phenotype subgroup (HR 1.66, 95% CI 1.21–2.28; p = 0.002) but not in classical PAH. The association remained significant after adjustment for age, gender, PAH diagnosis and 4‐strata ESC/ERS risk status. In patients with PAH and a lung phenotype, a rising alveolar‐to‐arterial oxygen gradient after therapy identifies patients at higher risk of death independently of conventional risk scores. Gas‐exchange indices should be further explored for inclusion in multidimensional risk assessment of PAH with coexistent lung disease.

## A112 Pulmonary Hypertension Associated With Chronic Lung Disease in Spain: Results From the REHAR Registry

Md, Phd Lucilla Piccari^1,2^, Manuel López‐Meseguer^3,4,5^, Álvaro Cantero Acedo^3^, Isabel Blanco^4,6^, Joan Albert Barberà^4,6^, Virginia Luz Pérez‐González^7^, Alejandro Cruz‐Utrilla^5,8,9^, Gregorio Pérez Peñate^10^, Adrián Vizoso^11^, Amaya Martínez Meñaca^5,12^, Anna Herranz Blasco^1,2^, Raquel López Reyes^13^, Juan Antonio Domingo Morera^14^, Leyre Chasco Eguilaz^15^, Salud Santos Pérez^16^, Roberto Del Pozo^17^, Cristina Sabater Abad^18^, Alberto García Ortega^19^, Ernest Sala‐Llinas^4,20^, Agueda Aurtenetxe Pérez^21^, Xavier Pomares^22^, Andrés Tenes^23^, Javier Carrillo Hernández‐Rubio^24^, Luis Jara‐Palomares^4,25^, Diego A Rodríguez‐Chiaradía^1,2,4,26^

^1^Hospital Del Mar, Barcelona, Spain, ^2^Hospital del Mar Research Institute, Barcelona, Spain, ^3^Respiratory Department, Hospital Universitari Vall d′ Hebron, Barcelona, Spain, ^4^Centro de Investigación Biomédica en Red de Enfermedades Respiratorias (CIBERES), Instituto de Salud Carlos III (ISCIII), Madrid, Spain, ^5^ERN‐LUNG (European Reference Network on Rare Respiratory Diseases), Spain, ^6^Department of Pulmonary Medicine, Hospital Clínic‐IDIBAPS, University of Barcelona, Barcelona, Spain, ^7^Lung Transplant Unit, Department of Respiratory Medicine, Hospital Universitario 12 de Octubre, Madrid, Spain, ^8^Pulmonary Hypertension Unit, Department of Cardiology, Hospital Universitario 12 de Octubre, Instituto de Investigación Hospital 12 de Octubre (i + 12), Madrid, Spain, ^9^Centro de Investigación Biomédica en Red de Enfermedades Cardiovasculares (CIBERCV), Instituto de Salud Carlos III (ISCIII), Madrid, Spain, ^10^Unidad Vascular Pulmonar. Neumologia. Hospital Universitario de Gran Canaria Dr Negrín. FIISSC Canarias, Las Palmas de Gran Canaria, Spain, ^11^Perioperative Medicine Research Group, Hospital del Mar Research Institute, Barcelona, Spain, ^12^Servicio de Neumología, Hospital Universitario Marqués de Valdecilla, Instituto de Investigación Valdecilla (IDIVAL), Santander, Spain, ^13^Pulmonary Hypertension Unit, Respiratory Department. Hospital Universitari i Politècnic La Fe, Valencia, Spain, ^14^Servicio de Neumología. Hospital Universitario Miguel Servet, Zaragoza, Spain, ^15^Respiratory Department, Galdakao‐Usansolo University Hospital, Usansolo, Spain, ^16^Pulmonology Department, Pneumology Research Group, Institut d'Investigació Biomèdica de Bellvitge – IDIBELL, Universitat de Barcelona, Hospital Universitari de Bellvitge, L'Hospitalet de Llobregat, Spain, ^17^Respiratory Department, Hospital Juan Ramon Jimenez, Huelva, Spain, ^18^Servicio Neumología, Consorcio Hospital General Universitario de Valencia, Valencia, Spain, ^19^Respiratory Department, Doctor Peset University Hospital, Valencia, Spain. Foundation for the Promotion of Health and Biomedical Research in the Valencian Region (FISABIO), Valencia, Spain, ^20^Servicio de Neumología, Hospital Universitario Son Espases, Palma de Mallorca, Spain, ^21^Servicio de Neumología, Hospital Universitario Basurto, Bilbao, Spain, ^22^Department of Respiratory Medicine, Parc Taulí Hospital Universitari, Institut d'Investigació i Innovació Parc Taulí (I3PT‐CERCA), Universitat Autònoma de Barcelona, Sabadell, Spain, ^23^Respiratory Department, Hospital Universitario Ramón y Cajal and Instituto Ramón y Cajal de Investigación Sanitaria IRYCIS, Madrid, Spain, ^24^Pulmonology Department, Hospital Universitario Rey Juan Carlos, Madrid, Spain, ^25^Respiratory Department, Medical Surgical Unit of Respiratory Diseases, Hospital Virgen del Rocio, Sevilla, Spain, ^26^Department of Medicine and Life Sciences (MELIS) Universitat Pompeu Fabra (UPF), Barcelona, Spain

Pulmonary hypertension (PH) is frequent and impactful in respiratory disease, but its characteristics in the Spanish population are unknown. We analysed the clinical characteristics and outcomes of patients with chronic obstructive pulmonary disease (COPD), interstitial lung disease (ILD) and combined pulmonary fibrosis and emphysema (CPFE) enrolled in the Spanish Registry of PH Associated with Respiratory Disease (REHAR) between January 2002 and December 2024.

Of the 447 patients, 216 had COPD, 186 had ILD and 45 had CPFE. In each group, severe PH showed distinct features compared to non‐severe PH, notably worse hypoxemia. Survival was worse in ILD and CPFE compared to COPD (p = 0.028), and in severe PH compared to non‐severe PH in COPD and CPFE, both according to the 6th World Symposium on PH (6WSPH) criteria (p = 0.048 and p = 0.021, respectively) and to the European Society of Cardiology/European Respiratory Society (ESC/ERS) 2022 guidelines (p = 0.005 and p = 0.027, respectively). However, in ILD none of the two classifications was associated with worse survival (p = 0.086 and p = 0.270 for 6WSPH and ESC/ERS, respectively); no PVR threshold could discriminate survival. Comparing the 6WSPH and the ESC/ERS hemodynamic classifications, 31% of patients changed hemodynamic category, those switching category having specific profiles. Treatment with pulmonary vasodilators was infrequent and associated with reduced survival. This study highlights clinical differences in a Spanish population with COPD, ILD and CPFE and in severe PH compared to non‐severe PH, with hemodynamic compromise having a different impact on survival in COPD, ILD and CPFE. Our results underscore the significant heterogeneity within Group 3 PH.

## A113 Laminar Flow Impacts Endothelial Junctional Protein Expression and Barrier Function Through Krüppel‐like Factor 2

Associate Professor Dustin Fraidenburg^1^, Kelsey Holbert^1^, Feifei Zhang^1^, Yulia Epshtein^1^, Christian Ascoli^1^, Jeffrey Jacobson^1^

^1^University Of Illinois Chicago, Chicago, USA

Flow and shear stress may be particularly important in mechanisms underlying pulmonary vascular disease, yet are not often incorporated into in‐vitro preparations. Krüppel‐like factor 2 (KLF2) is induced by laminar flow and is a central regulator of numerous endothelial cell processes. We hypothesize that laminar flow induces KLF2 in pulmonary artery endothelial cells (PAEC) and regulates junctional protein expression as a key mechanism in endothelial barrier function.

PAECs were exposed to laminar shear stress at physiologic flow (16 dynes/cm2) or pathologic flow (32 dynes/cm2) for 20 hours. RNA Sequencing identified differentially expressed genes (DEGs) which underwent pathway analysis. Western blotting, immunofluorescence, and trans‐endothelial resistance measures were used to identify differences in junctional protein expression and endothelial barrier function. Murine isolated‐perfused lung (IPL) experiments measured the effects of flow on pulmonary edema formation. RNA sequencing identified 1185 DEGs under physiologic flow and 2552 DEGs under pathologic flow compared to static conditions. KLF2 was one of the most highly upregulated genes. STRING analysis revealed a cluster of genes associated with integrin binding. Integrin‐B4 was strongly upregulated and closely associated with KLF2 expression. PAECs align with the direction of flow but become disorganized after KLF2 knockdown with changes in VE‐cadherin and F‐actin localization. Transendothelial resistance increases in response to flow but is blunted with KLF2 knockdown. Pathologic high flow in a murine IPL preparation result in decreased lung KLF2 expression with increased wet:dry ratio and Evan's blue dye extravasation. Laminar flow at both physiologic and pathologic levels is associated with highly differential gene regulation compared to static conditions. KLF2, a flow responsive transcription factor, is an important regulator of PAEC junctional protein expression and barrier function. Endothelial cells are particularly sensitive to flow shear stress and these underlying mechanisms play an important role in pulmonary vascular disease.

## A114 Aerosolized Gene and Oligonucleotide Therapy Targeting MicroRNA‐224 Ameliorates Pulmonary Hypertension by Orchestrating the Bmp Pathway

Dr. med. Olympia Bikou^1,2^, Aymen Halouani^3^, Malik Bisserier^2,4^, Petros Avramopoulos^5^, Carlos G. Santos‐Gallego^2^, Catherine Swarts^2^, Erik Kohlbrenner^2^, Kiyotake Ishikawa^2^, Marc Humbert^6^, Roger J. Hajjar^7^, Antonio Lax^8^, Stefan Engelhardt^5^, Lahouaria Hadri^2^, Sebastien Bonnet^9^, Yassine Sassi^2,3^

^1^Department of Medicine I, LMU University Hospital, Munich, Germany, ^2^Icahn School of Medicine at Mount Sinai, New York, USA, ^3^Fralin Biomedical Research Institute at Virginia Tech Carilion, Virginia, USA, ^4^Department of Cell Biology and Anatomy & Physiology, New York Medical College, New York, USA, ^5^Institute of Pharmacology and Toxicology, Technical University of Munich (TUM), Munich, Germany, ^6^Université Paris‐Saclay, Inserm U999, Assistance Publique Hôpitaux de Paris, Service de Pneumologie et Soins Intensifs Respiratoires, Hôpital Bicêtre, Le Kremlin‐Bicêtre, Paris, France, ^7^Gene and Cell Therapy Institute, Massachusetts General Brigham, USA, ^8^Biomedical Research Institute Virgen de la Arrixaca, University of Murcia, Murcia, Spain, ^9^Pulmonary Hypertension Research Group, Université Laval, Quebec, Canada

Pulmonary arterial hypertension (PAH) is a severe vascular disease that leads to right heart failure and death. Currently there is no cure for PAH and new therapeutic options targeting the roots of the disease are desperately needed. MicroRNAs (miRs) have emerged in the last decades as key regulators in health and disease. We identified miR‐224 as a lung enriched miR and in silico approaches for PAH predicted miR‐224 among the miRs that target PAH related genes. In this study we aimed to investigate the role of miR‐224 in pulmonary vascular remodeling, to define the mechanism of miR‐224 action in PAH and to test the therapeutic effect of miR‐224 inhibition in PAH.

We found pulmonary miR‐224 levels to be increased in the lungs of PH‐diseased mice, rats, pigs and humans. MiR‐224 is enriched in human pulmonary artery smooth muscle cells (hPASMCs), its level is increased in hPASMCs isolated from patients with PAH, and its expression is necessary and sufficient to induce hPASMCs proliferation. An intra‐tracheally aerosolized adeno‐associated virus (AAV)1‐tough decoy‐miR‐224 (AAV1‐TuD‐224) significantly decreased cardiac and pulmonary vascular remodeling (decreased pulmonary arterial‐ and right ventricular pressure, cardiomyocyte hypertrophy and pulmonary arterial medial thickness) in PH diseased mice. A chemically modified antisense oligonucleotide specific for miR‐224 (LNA‐224) significantly protected mice and rats from cardiac and pulmonary vascular remodeling, and alleviated PAH. Mechanistically, we found that miR‐224 represses BMP/SMAD signaling by directly targeting four members of this pathway and demonstrated that miR‐224 inhibition restores the balance between the growth‐promoting TGF‐β pathway and the growth‐inhibiting BMP pathway in PAH. Our study demonstrate that miR‐224 plays a pivotal role in pulmonary vascular remodeling by targeting the BMP pathway. Aerosolized gene and oligonucleotide therapy targeting miR‐224 may have therapeutic value for PAH.

## A115 SOX17 Enhancer Variant Reduces Endothelial Progenitor Cells in Patients With Pulmonary Arterial Hypertension

Dr James Klinger^**1**^, Dr Jason Hong^2^, Dr Micheala Aldred^3^, Dr. Christopher Rhodes^4^, Dr. Olin Liang^1^

^1^Alpert Medical School Brown University, Providence, USA, ^2^David Geffen School of Medicine, Los Angeles, USA, ^3^Indiana University School of Medicine, Indianapolis, USA, ^4^Imperial College London, London, UK

Rare mutations in SOX17 gene and common variants in its enhancer are associated with increased risk of pulmonary arterial hypertension (PAH), but the mechanism by which impaired SOX17 signaling contributes to PAH is not known. SOX17 plays a key role in driving differentiation of CD34+ multipotent progenitor cells toward an endothelial rather than a hematopoietic lineage. Analysis of a recently generated single‐cell RNAseq atlas of human lung in PAH patients identified a cluster of endothelial cells expressing CD34. We examined whether common SOX17 enhancer variants affect the proportion of endothelial cells that express CD34. Single‐nucleus RNA sequencing (scRNAseq) was performed on lung samples from 42 PAH and 25 age‐ and sex‐matched control lungs from the Pulmonary Hypertension Breakthrough Initiative. Cells were annotated by mapping onto the Human Lung Cell Atlas using Azimuth. Clustering identified over 30 cell types. Differential gene expression analysis was conducted using a pseudo bulk approach with edgeR. Gene set enrichment analysis was performed using the Hallmark pathways. Endothelial cell clusters were examined for CD34 expression. Lung samples from patients and controls were screened for the previously identified PAH risk alleles, single nucleotide variants (SNV) rs13266183 and rs10103692. Differences in endothelial cell CD34 expression were compared between patients with and without SOX17 risk variants.

Results: SOX17 expression was significantly higher in CD34+ compared with CD34‐ endothelial cells. No difference in the proportion of endothelial cells expressing CD34 were seen between patients with and without rs13266183, but patients homozygous for rs10103692‐A, had significantly lower proportion of CD34+ endothelial cells than patients without the risk variant. PAH patients with SOX17 enhancer variants have fewer CD34+ endothelial cells. These findings suggest that impaired SOX17 expression results in decreased differentiation of CD34+ progenitor cells into endothelial cells and may explain in part the increased risk of PAH.

## A116 Succesful Treatment With Balloon Pulmonary Angioplasty of a Patient With Recurrent CTEPH Due to Klippel Trenaunay Syndrome

Md Halil Ataş^1^, MD Derya Kocakaya, Prof Bülent Mutlu, Prof Bedrettin Yıldızeli

^1^Marmara University, Istanbul, Turkey

A 25‐year‐old male was admitted for evaluation and management of suspected pulmonary hypertension. He had a significant past medical history of deep vein thrombosis (2017), pulmonary embolism (2019), and chronic thromboembolic pulmonary hypertension (CTEPH), for which he underwent pulmonary endarterectomy in 2020. On admission, the patient presented with progressive dyspnea of two years’ duration and was on a mask with an oxygen saturation of 92%. Cardiovascular examination revealed elevated jugular venous pressure, and bilateral pretibial edema (+2/ + 2). A grade 3/6 systolic murmur A large, tender, violaceous skin lesion (50 × 30 cm) was noted on the right thigh.The lesion was identified as a capillary, venous, and lymphatic complex malformation with soft tissue hypertrophy and angiokeratomas, later diagnosed as Klippel‐Trenaunay syndrome, in which recurrent embolic events can occur, and pulmonary embolism is a common presentation. Functional status was consistent with WHO functional class IV. His medications included rivaroxaban (20 mg once daily) and riociguat (2.5 mg three times daily) Echocardiography showed severe tricuspid regurgitation, elevated estimated systolic pulmonary artery pressure (sPAP 70 mmHg), and evidence of right heart enlargement with impaired function Lower extremity ultrasonography demonstrated thrombotic material in the superficial venous system and within the vasculature of the right thigh lesion, without evidence of acute deep vein thrombosis. Right heart catheterization confirmed severe pulmonary hypertension, with pulmonary artery systolic pressure of 95 mmHg, mean pulmonary artery pressure of 58 mmHg, and pulmonary vascular resistance of 9.1 Wood units. Selective pulmonary angiography demonstrated bilateral diffuse segmental lesions, consistent with chronic thromboembolic pulmonary hypertension (CTEPH). Besides medical therapies, further treatment for CTEPH was planned as staged balloon pulmonary angioplasty. After 4 sessions of BPA his current the mean pulmonary artery pressure is 26 mmHg and PVR was calculated as 5.31 Wu.

## A117 Balloon Pulmonary Angioplasty for Inoperable or Residual/Recurrent Chronic Thromboembolic Pulmonary Hypertension: A Single‐Center Experience

Md Halil Ataş^1^, Prof Bülent Mutlu, MD Derya Kocakaya, Prof Bedrettin Yıldızeli

^1^Marmara University

Balloon pulmonary angioplasty (BPA) has recently emerged as a promising interventional option for chronic thromboembolic pulmonary hypertension(CTEPH). This study aimed to present the experience with BPA at a tertiary referral center for CTEPH. A total of 108 consecutive patients (64, 59.3% female) who underwent 423 BPA sessions, were included in the study. All patients underwent a detailed examination, including 6‐minute walking distance (6MWD), and right heart catheterisation at baseline and during the last BPA session. The mean age of the study population was 55 ± 15 years. Of these, 77 patients were diagnosed with inoperable CTEPH, while 31 patients presented with residual or recurrent CTEPH following pulmonary endarterectomy (PEA). After BPA, the six‐minute walk distance (6MWD) improved from 325 ± 127 m to 368 ± 110 m (p < 0.001), and NT‐proBNP levels decreased from a median of 558 pg/mL to 280 pg/mL (p = 0.001). Hemodynamically, the mean pulmonary artery pressure decreased from 42.9 ± 13.4 mmHg to 34.2 ± 10.9 mmHg (p < 0.001), and pulmonary vascular resistance declined from 7.6 ± 4.7 to 4.8 ± 3.1 Wood units (p < 0.001). The cardiac index increased slightly from 2.5 ± 0.7 to 2.7 ± 0.6 L/min/m²; however, this change did not reach statistical significance (p = 0.3). Balloon pulmonary angioplasty led to improvements in haemodynamics and six‐minute walk distance, while decreasing the need for supplemental oxygen, with an acceptable risk–benefit profile in patients with inoperable CTEPH as well as those with residual or recurrent disease.

## A118 Perivascular Adipose Tissue is an Immune Hub Driving Complement‐Mediated Vascular Remodeling in Hypoxia‐Induced Pulmonary Hypertension

Instructor Min Li^1^, Brittany McKeon^1^, Camille Daigle^1^, Greta Krafsur^1^, Sushil Kumar^1^, Suzette Riddle^1^, Maria Frid^1^, Sue Gu^1^, Professor Kurt Stenmark^**1**^

^1^University Of Colorado, Aurora, USA

Vascular stiffness predicts outcomes in systemic and pulmonary hypertension and reflects extracellular matrix (ECM) remodeling. Our proteomic studies in a hypoxia‐induced PH calf model revealed early matrisome changes and robust upregulation of complement factors, most prominently in the main PA, and we have previously established that complement is a potent driver of immune‐vascular crosstalk in pulmonary vascular disease. Perivascular adipose tissue (PVAT) has emerged as a key source of adipsin (complement factor D) and C3, fueling alternative pathway activation and vascular inflammation. We hypothesized that in hypoxic PH, PVAT surrounding the main PA promotes stiffening by releasing adipsin, which activates complement and macrophages, leading to ECM remodeling through collagen crosslinking and protease induction. Vascular stiffness and collagen and elastin organization and immune cell infiltration were assessed in human MPAs and hypoxia‐exposed calf/rat MPAs by 2‐dimensional stress‐strain testing, trichrome and Verhoeff–Van Gieson (VVG) staining, and immunofluorescence. Gene expression of cytokines, chemokines, and complement factors were measured in PA wall cells, PVAT, and other fat depots. Conditioned media (CM) from these fat depots was applied to bone marrow derived macrophages (BMDMs). Human and bovine PH‐MPAs showed marked changes in vascular stiffening in response to hypoxia, in addition to collagen accumulation, elastin degradation, and immune infiltration. PVAT from PH animals exhibited increased Ccl2, adipsin, C3, and S100As compared to controls. CM from PH‐PVAT robustly activated BMDMs, upregulating complement receptors and profibrotic/ECM‐remodeling genes IL‐1β, TGFβ1, LOX, TGM2, and MMP2/9, whereas subcutaneous fat CM had smaller effects. PVAT functions as a local immune‐complement signaling niche in PH, with adipsin‐driven complement activation amplifying macrophage responses and ECM remodeling, thereby contributing to proximal PA stiffening.

## A119 Spatial and Molecular Profiling of Soluble and Matrix‐Bound Extracellular Vesicles in the Pulmonary Artery

Instructor Sushil Kumar^1^, Instructor Hui Zhang^1^, Instructor Ram R Prasad^1^, Brittany McKeon^1^, Xinbo Yu^1^, Suzette Riddle^1^, Christina Coughlan^1^, Anza Darehshouri^1^, Professor Kirk Hansen^1^, Professor Kurt Stenmark^1^

^1^University Of Colorado, Aurora, USA

Extracellular vesicles (EVs) are emerging as key mediators of intercellular communication in the pulmonary vasculature, influencing endothelial, smooth muscle, fibroblast, and immune cells, including macrophage interactions that drive vascular remodelling. Recent studies have implicated EVs in the propagation of inflammatory and fibrotic signals in pulmonary hypertension (PH), yet most work has focused on EVs isolated from biofluids or cultured cells. Their spatial localization, cellular sources, and biochemical diversity within the intact pulmonary artery wall remain poorly defined. Understanding the distribution and composition of both soluble EV and Matrix bound nanovesicles (MBV) is therefore critical for elucidating how extracellular communication networks are organized and altered in disease. We combined precision‐cut lung slices (PCLS) with transmission electron microscopy TEM to visualize EVs directly within human, bovine, and mouse pulmonary arteries. A new isolation workflow was developed to extract and compare soluble EVs and MBVs from the same tissue source. Vesicle populations were analyzed using nanoparticle tracking analysis (NTA), TEM, and quantitative proteomics. High‐resolution TEM revealed abundant EVs distributed throughout the pulmonary artery wall, with the greatest density in the adventitia, indicating this region as a major signalling niche. Both soluble EVs and MBVs showed similar size profiles but distinct proteomic signatures. MBVs were enriched in extracellular matrix and fibroblast‐associated proteins, whereas soluble EVs contained canonical exosome markers. Proteomic analysis of EVs revealed significant disease‐associated changes. In PH samples, we observed increased fibroblast‐derived proteins (PDGFRA) and complement proteins, including C3, C4, C5, C6, C7, C8A, and C1QA, suggesting enhanced inflammatory and remodeling processes. This study provides the first spatial and molecular analysis of EVs in intact pulmonary artery tissue. By distinguishing soluble from MBV subpopulations, it reveals new aspects of extracellular communication in the vascular wall and highlights disease‐related shifts that could inform EV‐based biomarkers and therapies for PH.

## A120 Adventitial Fibroblast Derived Complement Factor D Promotes Inflammation and Immune Cell Crosstalk in Pulmonary Hypertension

Instructor Ram Raj Prasad^1^, Instructor Sushil Kumar^1^, Associate Professor Cheng‐Jun Hu^1^, Instructor Hui Zhang^1^, Instructor Min Li^1^, Suzette Riddle^1^, Britanny McKeon^1^, Professor Kurt Stenmark^1^

^1^University Of Colorado, Aurora, USA

Pulmonary hypertension (PH) is a progressive and ultimately fatal vascular disease characterized by perivascular inflammation and remodeling driven by persistently activated adventitial fibroblasts that adopt a proinflammatory and metabolically reprogrammed phenotype. Emerging evidence implicates the complement system—including local intracellular complement activity (the “complosome”)—as a critical regulator of these processes. Our recent studies demonstrated that intracellular complement proteins C3, CFB, and particularly CFD are essential for maintaining fibroblast activation in PH. Within fibroblasts, CFD interacts with CFB to activate C3, generating C3a that signals through mitochondrial C3aR1 to modulate metabolic and inflammatory programs. However, these mechanisms have primarily been studied in isolated cell cultures, which fail to recapitulate the complex tissue microenvironment. To address this limitation, we employed precision‐cut lung slices (PCLS) to investigate CFD‐mediated inflammatory signaling and immune–stromal crosstalk in situ. Using human and bovine PCLS, we examined the impact of pharmacological CFD inhibition (ALXN2050) on complement activation and inflammatory signaling. In PCLS derived from bovines with established PH, ALXN2050 markedly reduced C3a production and suppressed expression of key proinflammatory mediators including CCL2, CXCL12, and IL6. In healthy human and bovine PCLS, combined IL‐1β stimulation and hypoxia induced a robust inflammatory transcriptional response (IL6, CCL2, CXCL12, C3), which was significantly attenuated by CFD inhibition. Furthermore, in a hypoxia‐induced co‐culture model of PCLS with bone marrow–derived macrophages (BMDMs), hypoxia promoted macrophage infiltration and upregulation of IL1B, CCL5, CXCL12, and C3—effects that were substantially blunted by ALXN2050 treatment. These findings establish fibroblast‐derived CFD as a key mediator of inflammation and immune cell recruitment in PH. Pharmacological inhibition of CFD effectively suppresses complement activation, inflammatory gene expression, and macrophage infiltration in native lung tissue, identifying CFD as a promising therapeutic target to disrupt the self‐sustaining inflammatory milieu that drives pulmonary vascular remodeling in PH.

## A121 Safer Awake Procedures: Reducing Risk Through Buffered Lidocaine and Psychosocial Preparation in Pulmonary Vascular Resistance Studies

Senior Health Play Specialist Kirsten Black^1^

^1^Great Ormond Street Hospital Nhs Trust, London, United Kingdom

This presentation introduces a new program designed to reduce sedation‐related risks in PVR studies by integrating health play specialist support into procedural care. While right‐heart catheterisation remains essential for managing pulmonary hypertension, sedation—especially general anaesthesia—can pose serious complications. To improve safety, the clinical team transitioned to awake procedures using local anaesthetic for patients aged 12 and older. Health play specialists were instrumental in this shift, providing developmentally appropriate education, emotional support, and coping strategies that enabled patients to complete procedures without sedation. After supporting the first patient, a play specialist identified a key barrier to compliance: the burn of lidocaine injections, caused by the low pH compared to human tissue. Advocating for change, they initiated a multidisciplinary effort to introduce buffered lidocaine—created by mixing lidocaine with sodium bicarbonate in a 10:1 ratio—to neutralize the pH and reduce injection pain. Since the program began, eight awake PVR studies have been completed: six using standard lidocaine and two with buffered lidocaine. Implementation involved collaboration among cardiologists, pharmacists, and nursing teams, with knowledge‐sharing from providers already using buffered lidocaine. Although the sample size is limited, the program has seen a significant drop in patient‐reported pain scores (from ~7.5 to ~3), reduced staff anxiety, and decreased reliance on sedation. Challenges included navigating scope‐of‐practice boundaries, coordinating communication across departments, and addressing the lack of standardized protocols. The program will continue and expand to more patients, with plans to extend buffered lidocaine use and play specialist support to other procedures under local anaesthetic—specifically dental services and interventional radiology for PICC and Hickman line placements. Next steps include formalizing practice guidelines and research on patient outcomes, staff morale, and cost‐effectiveness. This initiative highlights the critical role of play specialists in procedural success and demonstrates how advocacy can drive meaningful, patient‐centered change and improve outcomes.

## A122 Identification of Interleukin 7 Signaling As A Biomarker of Pulmonary Vascular Pathology in Preterm Infants

Prof. Dr. Jennifer Sucre, PhD Alida Kindt, Friederike Haefner, Annika Fomm, Dr. Motaharehsadat Heydarian, PhD von Toerne Christine, Dr. Stefanie Hauck, Dr. Kai Förster, Prof. Dr. Andreas Flemmer, Prof. Dr. Sophia Stoecklein, PhD Markus List, Prof. Dr. Mark Toshner, Prof. Dr. Med. Anne Hilgendorff

Cardiopulmonary complications have become the most prevalent morbidity in preterm infants longer term. Early, non‐invasive biomarkers indicating early‐stage pulmonary vascular disease (PVD) in preterms with chronic lung disease (BPD, bronchopulmonary dysplasia) are needed, to understand and monitor the pathology, characterized by a high risk for progression into pulmonary hypertension (PH). We prospectively included 190 preterm infants born < 32 weeks postmenstrual age in the AIRR (Attention to Infants at Respiratory Risks) study (DRKS00004600) after informed parental consent. Identification of pathologic pulmonary artery flow and right heart strain by cardiopulmonary magnetic resonance imaging (MRI) at near‐term age enabled us to identify plasma markers associated with PVD early after birth from proteomic screening. The differential regulation of IL‐7 and its receptor IL7R, observed together with markers of endothelial injury and remodeling, was confirmed by RNA scope analysis in lung tissue (Vanderbilt University) from preterms with and without BPD and/or BPD‐PH. Single cell analysis confirmed expression on endothelial cells and provided insight into IL‐7 driven interaction with epithelial and immune cell populations. Analysis of genetic data provided background information linking mutations to lung disease in preterm infants.

Showcasing the regulation of IL‐7R expression in infants with BPD indicates a role of IL7 signaling in PVD pathophysiology and outlines its potential as a disease marker and future therapeutic target.

## A123 Classifying the Unclassifiable: A Case of Diagnostic Uncertainty in Pulmonary Hypertension

Dr Cameron Forward^1,2^, Dr Sarah Cullivan^1,2^, Dr Brian McCullagh^1,2^

^1^National Pulmonary Hypertension Unit, Mater Misericordiae University Hospital, Dublin, Ireland ‐ HIC, ^2^School of Medicine, University College Dublin, Dublin, Ireland ‐ HIC

Correct classification of PH can be difficult when patients present with multiple potential mechanisms. Case Description We describe the case of a 41‐year‐old, never smoker male plumber who was referred to the Pulmonary Hypertension Unit with one‐year of dyspnoea. Clinical examination revealed no peripheral edema, clear lung auscultation and a prominent P2. 6MWD was reduced at 360 m. Echocardiogram was suggestive of PH. CT thorax did not demonstrate any interstitial abnormalities but mediastinal and bi‐hilar adenopathy was present. EBUS of stations 11 L and 7 showed multiple non‐caseating granulomas. No infiltration was seen on CMRI. V/Q imaging was negative. NT‐proBNP was 18 pg/ml. He proceeded to RHC which demonstrated pre‐capillary PH with a mPAP of 41 mmHg, PAWP of 6 mmHg, and a PVR of 7.5WU. He was commenced on dual oral therapy followed by selexipag. One‐year later he was transitioned to subcutaneous treprostinil and was referred for lung transplantation. PFT's demonstrated normal spirometry but severely reduced gas transfer with a DLCO of 33%. Genetic analysis was negative. PVOD is a rare cause of PH but is considered in patients with a DLCO < 40% and characteristic CT features. Treatment with PH targeted therapies is controversial and the cornerstone of management is prompt lung transplant referral. This case demonstrates a low DLCO and mediastinal adenopathy but does not demonstrate characteristic interstitial changes such as interlobular septal thickening and ground‐glass opacification. IPAH with a severely‐reduced DLCO is within the differential. However, this population is typically older and has a history of tobacco exposure both of which are not features in our case. Sarcoidosis associated PH is a rare diagnosis. The lack of parenchymal disease and infiltration on CMRI makes this diagnosis unlikely. This case highlights the difficulty in classifying PH in patients presenting with multiple confounding factors.

## A124 Reversible Pre‐Capillary Pulmonary Hypertension due to a long‐standing Arteriovenous Fistula

M.D. Laura Saldivar^1^, APRN‐CNP Emily Garber^2^, M.D. Khaled Boubes^3^, M.D. Elie Homsy^1^

^1^Department of Pulmonary, Critical Care & Sleep Medicine, The Ohio State University Wexner Medical Center, Columbus, United States ‐ HIC, ^2^Department of Pulmonary, Cardiology, the Ohio State University Wexner Medical Center, Columbus, United States ‐ HIC, ^3^Department of Pulmonary, Nephrology, the Ohio State University Wexner Medical Center, Columbus, United States ‐ HIC

Chronic kidney disease predisposes patients to the development of pulmonary hypertension (PH) via multiple potential mechanisms such as via endothelial dysfunction, systemic hypertension, and left to right shunting through arteriovenous (AV) fistulas or grafts. AV fistulas have been found to be superior to AV grafts in terms of maintaining patency, however unlike grafts fistulas can expand significantly over time which predisposes patients to high output heart failure and subsequent PH. PH is independently associated with increased mortality even amongst those without co‐morbid CKD. This report demonstrates a case of newly diagnosed pre‐capillary PH in a young patient presenting with shortness of breath and cough. The patient had an AV fistula placed 18 years prior to presentation for CKD related to reflux nephropathy and ultimately underwent transplantation. Pulmonary angiography was negative for PE but showed signs of right heart strain and pulmonary arterial dilation. Echocardiography demonstrated an elevated RVSP of 67 mmHg that was confirmed via right heart catheterization. This case was unique in that the patient's pre‐capillary PH was reversed after AV fistula ligation. Additionally, after ligation the patient also experienced improvement in renal graft function (Cr 1.43 to 1.2 mg/dL). This case demonstrates the effect that AV fistulas can have on cardiac function; clinicians should survey patients with AV fistulas for PH via screening echocardiography, and, if abnormal, should perform right heart catheterization for confirmation. Fistulas with outputs greater than 1.5‐2 L/min should be considered for revision or ligation to decrease the risk of development of PH.

## A125 Effect of Nasal Low‐Flow Oxygen Therapy vs. Ambient Air on 6‐Minute Walk Distance in Patients With Pulmonary Vascular Disease Revealing Exercise‐Induced Desaturation

**A Pragmatic Randomized‐Controlled Non‐Inferiority Cross‐Over Trial**

Mrs Carmen Wick^1,2^, Mona Lichtblau^1,2^, Alessandro Vella^1,2^, Esther Schwarz^1,2^, Julian Müller^1^, Michael Furian^1^, Anna Titz^1^, Silvia Ulrich^1,2^

^1^Department of Pulmonology, University Hospital Of Zurich, Zurich, Switzerland, ^2^Faculty of Medicine, University of Zurich, Zurich, Switzerland

Patients with pulmonary vascular disease, pulmonary arterial and distal chronic thromboembolic pulmonary hypertension (PAH/CTEPH), reveal exertional dyspnea frequently accompanied by oxygen desaturation. Ambulatory nasal low‐flow supplemental oxygen therapy (SOT) is often prescribed, but the weight of portable concentrators may counteract potential SOT‐benefits. This was a pragmatic randomized controlled non‐inferiority cross‐over study in which patients with PAH or CTEPH, who revealed exercise‐induced oxygen desaturation > 3%, performed two six‐minute walk distance (6MWD) tests one in ambient air and one with low‐flow (1‐2 l/min) nasal SOT by a portable back‐packed concentrator, in randomized order. Maximal 6MWD was measured as primary outcome. The trial is registered on clinicaltrials.gov (NCT06384534). Forty participants (age 62 ± 15 years, 21 female, 20 PAH, 20 CTEPH) were included. The mean difference in 6MWD between ambient air and SOT was 0 m (95% CI: –15 to 14 m, p  >  0.0001), confirming non‐inferiority of ambient air as the lower confidence bound did not exceed the predefined margin of ‐35 m. Secondary outcomes showed higher SpO2 and reduced dyspnea with SOT, particularly in PAH. 52% of patients preferred ambient air, 38% SOT, 5% preferred none, 5% were not assessed. Low‐flow nasal ambulatory SOT may not follow a one‐size‐fits‐all approach but rather requires personalized treatment tailored to individual patient's needs and response. Future research should focus on identifying SOT‐responders versus non‐responders, and on developing lightweight, comfortable but still powerful SOT devices adapted to patients’ needs.

## A126 Investigating the Effect of Senolytic Therapy on Cigarette Smoke–Induced Pulmonary Hypertension in Mice

Dr Mira Yasemin Gökyildirim^1^, M.Sc. Rahul Mirgal^2^, M.Sc. Vinita Sharma^2^, Dr. vet. med. Simone Kraut^2^, Dr. med. Serge Adnot^3^, Dr. rer. nat. Norbert Weissmann^2^, PhD Marija Gredic^2^

^1^Institute for Lung Health (ILH), Universities of Giessen and Marburg Lung Center (UGMLC), Member of the German Center for Lung Research (DZL), Justus‐Liebig‐University (JLU), Giessen, Germany, Giessen, Germany, ^2^Cardio‐Pulmonary Institute (CPI), Institute for Lung Health (ILH), Universities of Giessen and Marburg Lung Center (UGMLC), Member of the German Center for Lung Research (DZL), Justus‐Liebig‐University (JLU), Giessen, Germany, Giessen, Germany, ^3^INSERM U955, Département de Physiologie‐Explorations Fonctionnelles and FHU SENEC Hôpital Henri Mondor, AP‐HP, Créteil, France, Créteil, France

Chronic obstructive pulmonary disease (COPD) is a progressive, incurable condition and the third leading cause of death worldwide. In addition to respiratory tract pathology, up to 90% of COPD patients develop at least mild pulmonary hypertension (PH). Important pathogenic factors in emphysema and COPD include both structural damage to the elastin fibres of the lungs and increased senescence of the lung cells, which alters their secretion and impairs the lung tissue regeneration.

Previous studies have shown that treatment with the senolytic drug ABT263 eliminates senescent cells in the lungs. ABT263 treatment led to an improvement in lung structure and function after emphysema induction in young mice. However, elimination of senescent cells worsened pulmonary hemodynamics in rodent models of PH. In this study, we wanted to investigate whether the treatment with the senolytic drug ABT263 and the resulting elimination of senescent cells improve lung structure and function, and affect pulmonary vascular alterations after cigarette smoke exposure of mice. To explore this, we exposed wild‐type mice to cigarette smoke for 8 months. Afterward, they were re‐exposed to room air and treated with either ABT263 or a vehicle for another 7 weeks. Lung and heart function and morphology were examined to assess the effects of ABT263.

In contrast to the findings in young mice, no improvement in lung function was observed after ABT263 treatment. Interestingly, removal of senescent cells did not exacerbate PH in this animal model, based on hemodynamic measurements and echocardiographic assessment of right heart function and dimensions. In summary, our data demonstrate that the elimination of senescent cells in mice with fully established cigarette smoke‐induced lung pathology does not improve lung function. Moreover, our findings suggest that the role of senescence in the progression of PH may differ between the smoke model and other forms of PH in animal studies.

## A127 Functional Role of Kir2 Channels in Pulmonary Vascular Cells and Their Involvement in Pulmonary Arterial Hypertension

Señora Bianca Barreira‐Barba^1,2,3^, Daniel Morales Cano^1,2,3^, Laura Moreno^1,2,3^, Beatriz de Olaiz^4^, Rui Adão^1,2,3^, Angel Cogolludo^1,2,3^, Francisco Perez Vizcaino^1,2,3^, Maria Sancho^2,3,5,6^

^1^Department of Pharmacology and Toxicology, School of Medicine, Universidad Complutense de Madrid, Madrid, Spain, ^2^Centro de Investigación Biomédica en red Enfermedades Respiratorias (CIBERES), Madrid, Spain, ^3^Instituto de Investigación Sanitaria Gregorio Marañón (IiSGM), Madrid, Spain, ^4^Department of Thoracic Surgery, Hospital Universitario de Getafe, Getafe, Spain, ^5^Department of Physiology, School of Medicine, Universidad Complutense de Madrid, Madrid, Spain, ^6^Department of Pharmacology, University of Vermont, Burlington, USA

Strongly inward‐rectifying K+ (Kir2) channels are key regulators of vascular membrane potential (VM), contributing to endothelium‐dependent hyperpolarization and the active hyperemia response across various vascular systems, including the brain circulation. However, their functional role in pulmonary vascular cells is poorly characterized, with limited evidence primarily based on cultured bovine cells. Although no mutations in the genes encoding Kir2 subunits have been directly linked to Pulmonary Arterial Hypertension (PAH), previous research reports a modest downregulation of Kir subunit expression in the lungs of PAH patients. This study investigated the functional expression of Kir2 channels in pulmonary arteries (PAs), and their potential pathophysiological implications in PAH. Using the conventional whole‐cell configuration of the patch‐clamp technique, Ba2 + ‐sensitive Kir2 currents were recorded in freshly isolated smooth muscle (PASMC) and endothelial (PAEC) cells from rat PAs. Immunohistochemical analysis confirmed the expression of Kir2.1 and Kir2.2 subunits in both cell types. Functionally, Ba2+ (3‐100 μM) induced a concentration‐dependent contraction of PAs in a wire myograph, supporting a role for Kir2 channels in regulating pulmonary vascular tone. In PAH rats (SU5416 plus hypoxia model), Kir2 currents and Kir2.1/Kir2.2 protein expression were significantly reduced in PASMCs and PAECs. Accordingly, Ba2 + ‐induced PA contractions were attenuated, and the Kir2 blocker (100 μM) impaired ACh (1 nM–100 μM)‐induced endothelium‐dependent relaxations. Our findings indicate that Kir2 channels are functionally expressed in pulmonary arteries, where they play a key role in controlling VM and vascular tone. Furthermore the downregulation of Kir2 expression and function in PAH suggests these channels could represent novel therapeutic targets for this devastating disease.

## A128 Endothelial Progenitor Cells From Pulmonary Arterial Hypertension Patients Display Altered Sensitivity to Tlr Agonists

Ana Conde‐Yañez^1^, Rebeca Carrion‐Marchante^1^, Lourdes Baron‐Argos^1^, Señora Bianca Barreira‐Barba^1^, Patricia Sosa^1^, Alejandro Rodriguez‐Ogando^2^, Francisco Perez‐Vizcaino^1^, Maria Toledano^3^, Maria Jesus del Cerro^3^, Maria Alvarez‐Fuente^3^, Laura Moreno^1^

^1^Pharmacology & Toxicology Department, School of Medicine, Universidad Complutense de Madrid, Instituto de Investigación Sanitaria Gregorio Marañón(IiSGM). Ciber Enfermedades Respiratorias(CIBERES), Madrid, Spain, ^2^Pediatric Cardiology Section, “Gregorio Marañon” General University Hospital, Madrid, Spain, ^3^Pediatric Cardiology Section, Ramón y Cajal University Hospital, Madrid, Spain

In children, pulmonary hypertension (PH) is commonly associated with underlying cardiac or lung disease such as congenital heart disease (CHD) or bronchopulmonary dysplasia (BPD). Increasing evidence suggests that inflammation contributes to pulmonary arterial hypertension (PAH), as patients with advanced PAH show increased levels of inflammatory mediators such as IL‐6. Toll‐like receptors (TLRs) have also been implicated in PAH by modulating vascular inflammation and remodeling. Endothelial progenitor cells (EPCs) are crucial in vasculogenesis but their contribution to disease progression remains unclear. This study aims to characterize the potential dysfunction of innate immune receptors in EPCs isolated from paediatric patients with or without PH.

EPCs were isolated from 7 paediatric patients with CHD without PH (non‐PH) and 11 patients with different forms of PH: Idiopathic PAH (IPAH), CHD‐associated PH and PH associated with BPD. EPCs were incubated in basal medium or stimulated with different TLRs agonists and IL‐6 levels were quantified. EPCs responses were compared with those of commercial pulmonary arterial endothelial cells (HPAECs). EPCs from paediatric patients exhibited a phenotype comparable to that of HPAECs.

Under basal conditions, EPCs from IPAH patients secreted higher levels of IL‐6 than EPCs isolated from patients without PAH or with other forms of PH. Moreover, while the responses to TLR3 and TLR4 agonists of EPCs from non‐PH patients and patients with other forms of PH were comparable to those of HPAECs, EPCs from IPAH patients showed increased sensitivity to TLR activation.

These findings suggest that EPCs from children with IPAH display a proinflammatory phenotype and may actively contribute to the inflammatory milieu underlying PH progression. Further research in this field could provide valuable insights for the development of targeted therapeutic approaches for different forms of PH. Funded by ISCIII (PI19/01616, PI22/01302), CAM (S2022/BMD‐7409) and NextGenerationEU.

## A129 Initial 6‐Minute‐Walk Distance Z Score is not Associated With Survival In A Large Cohort Of Pediatric Patients With Pulmonary Arterial Hypertension

Dr. Elisa K. McCarthy^1^, Dr. Doff B. McElhinney^1,2^, Michelle T. Ogawa^3^, Esther Liu^3^, Dr. Jeffrey A. Feinstein^1^, Dr. Rachel Hopper^1^

^1^Department of Pediatrics (Cardiology), Stanford University School of Medicine, Palo Alto, USA, ^2^Department of Cardiothoracic Surgery (Pediatric Cardiac Surgery), Palo Alto, USA, ^3^Stanford Children's Health, Palo Alto, USA

Six‐minute walk distance (6MWD) is often considered in risk stratification for children with pulmonary arterial hypertension (PAH), with 6MWD < 350 m considered “high risk” in patients > 6 years old. Impact of age/height/sex on 6MWD presents unique challenges in using a universal cutoff.

We sought to evaluate 10‐year survival prediction of initial 6MWD Z‐score vs 350 m cutoff in pediatric PAH. We analyzed all first‐time 6MWD in pediatric PAH patients at a large tertiary referral center. Z‐scores were calculated for each 6MWD according to previously published Geiger reference equations based on sex, age, and height. Freedom from time‐related outcomes was estimated using the Kaplan‐Meier method, with 95% confidence intervals generated with complementary log‐log transformation and compared between groups using the log‐rank test. 105 patients aged 3 ‐ 18 years had an initial 6MWD within 3.3 + 3.9 years following initial diagnosis of PAH (51% congenital heart disease‐related, 39% idiopathic/heritable). Estimated 10‐year transplant‐free survival in those with initial 6MWD > 350 m was 45% (35, 55) and did not differ from those with initial 6MWD < 350 m (53% (66, 40)) (p = 0.85). Patients’ 6 MW distances were roughly evenly distributed between the three Z‐score groups: “normal” (Z > ‐2), “mild/moderately decreased” (‐2 > Z > ‐4), and “severely decreased” (Z < ‐4). Comparing 6MWD across categories, there was no difference in 10‐year transplant‐free survival (p = 0.23). Comparing “normal” to “decreased” (Z < ‐2) 6MWD, there is a suggestion of better 10‐year transplant‐free survival in the “normal” cohort (68% (55,81)) v. (38% (29, 47)), but not statistically significant (p = 0.08). This study presents an approach for characterizing the relationship between outcomes in pediatric PAH using standardized 6MWD data Z‐scores, rather than absolute values. While potentially better than a set cutoff for all children, 6MWD Z‐score does not reliably predict 10‐year transplant free survival, suggesting a need to incorporate serial performance and additional clinical metrics.

## A130 Long‐Term Survival and Prognostic Impact of the Time‐Dependent Changes in Outcome Measures Across The Pulmonary Hypertension Groups: A Single‐Center Cohort

Prof Cihangir Kaymaz^1^, MD Hacer Ceren Tokgoz^1^, MD Barkin Kultursay^2^, MD Seda Tanyeri^1^, MD Cagdas Bulus^1^, MD Dicle Sirma^1^, MD Metehan Kibar^1^, MD Can Erdem^1^, MD Aziz Vezir^1^, MD Ayse Nur Kucuk^1^, MD Ipek Akkus^1^, MD Seyma Zeynep Atici^1^, MD Seyma Cicek^1^, MD Mesut Ozkutukcu^1^, MD Oguz Donmez^1^, MD Omer Dogukan Ertan^1^, MD Ridvan Bolatarslan^1^, MD Mustafa Bulut^3^, MD Aykun Hakgor^4^, MD Ahmet Sekban^1^, MD, Professor of Cardiology Ibrahim Halil Tanboga^5^, MD Zubeyde Bayram^1^, MD, Professor of Cardiology Nihal Ozdemir^1^

^1^Kosuyolu Heart Research And Education Hospital, Istanbul, Turkey, ^2^Tunceli State Hospital, Tunceli, Turkey, ^3^Ahi Evren Thoracic and Cardiovascular Surgery Training and Research Hospital, Trabzon, Turkey, ^4^Medipol University Faculty of Medicine, Istanbul, Turkey, ^5^Nisantasi University Faculty of Medicine, Istanbul, Turkey

In this study, we evaluated the long‐term survival and prognostic impact of the time‐dependent changes in the outcome measures across the pulmonary hypertension (PH) groups and subgroups. Our single‐center and retrospective study comprised the 1213 patients with PH. All patients were under periodical follow‐up visits with 3 to 6 months intervals. Primary outcome measure was all‐cause mortality, and composite end‐points included the criteria utilized in SERAPHIN and GRIPHON studies. Multiparametric risk scores (MRSs) including REVEAL, REVEAL 2.0, REVEAL Lite 2, COMPERA, SPAHR and FPHN were also used. Median follow‐up was 552 days (1 to 7034 days). All MRSs showed sustained improvement from baseline (p < 0.001). Mortality was noted in 40.4% of patients. A trend for increased mortality co‐inciding the COVID‐19 Pandemic Era was followed by a decrease in mortality (p < 0.05). Five‐year survival for groups 1, 2, 3 and 4 were 56.9%, 40.6%, 27.4% and 51.9%, respectively (p < 0.001). Clinical deterioration were most frequently documented within first 6 to12 months, and separation among survival curves became evident at that time. Increased mortality was noted in CTD‐APAH (HR = 3.137, 95% CI: 2.118‐4.646, p < 0.001) and IPAH (HR = 1.608, p < 0.001) subgroups as compared to those with CHD‐APAH whereas survival curves were comparable across the CHD‐APAH subgroups (p = 0.699). Improvements in six‐minute walk distance (6MWD), functional class (FC), tricuspid annular plane systolic excursion (TAPSE) (p < 0.001, for all) and systolic pulmonary artery pressure (PASP) estimates (p = 0.043) were consistent across the all subgroups. The most powerful predictors for mortality were TAPSE/PASP ratio, TAPSE, FC, 6MWD, pericardial effusion and right atrial mean pressure (p < 0.001 for all). In our PH cohort, a decreasing trend in the mortality was evident, and periodical risk assessments provide reliable predictions for response to treatments even at early phases of follow‐up.

## A131 Importance of Lower Risk Status Attained at Early Period for Durability of Long‐Term Clinical Benefit in Pulmonary Arterial Hypertension: A Single‐Center Cohort

Prof Cihangir Kaymaz^1^, MD Hacer Ceren Tokgoz^1^, MD Barkin Kultursay^2^, MD Seda Tanyeri^1^, MD Cagdas Bulus^1^, MD Dicle Sirma^1^, MD Metehan Kibar^1^, MD Can Erdem^1^, MD Aziz Vezir^1^, MD Seyma Zeynep Atici^1^, MD Seyma Cicek^1^, MD Emre Kipritci^1^, MD Mustafa Kara^1^, MD Mesut Ozkutukcu^1^, MD Oguz Donmez^1^, MD Aykun Hakgor^3^, MD, Professor of Cardiology Ibrahim Halil Tanboga^4^, MD Zubeyde Bayram^1^, MD, Professor of Cardiology Nihal Ozdemir^1^

^1^Kosuyolu Heart Research And Education Hospital, Istanbul, Turkey, ^2^Tunceli State Hospital, Tunceli, Turkey, ^3^Medipol University Faculty of Medicine, Istanbul, Turkey, ^4^Hisar Intercontinental Hospital, Istanbul, Turkey

In this study, we analysed the time‐dependent changes in the multiparametric risk scores (MRSs) used in the risk stratification of pulmonary arterial hypertension (PAH). Our single‐center and retrospective study comprised the 1213 patients with PH. All patients were under periodical follow‐up visits with 3 to 6 months intervals. Primary outcome measure was all‐cause mortality, and composite end‐points included the criteria utilized in SERAPHIN and GRIPHON studies. Multiparametric risk scores (MRSs) including REVEAL, REVEAL 2.0, REVEAL Lite 2, COMPERA, SPAHR and FPHN were also used. Median follow‐up was 552 days (1 to 7034 days). Five‐year survival for groups 1, 2, 3 and 4 were 56.9%, 40.6%, 27.4% and 51.9%, respectively (p < 0.001). All MRSs showed significant improvement from baseline to first visits (p < 0.001 for all). From baseline to Visit 1, REVEAL score decreased rom 8.83 + 2.11 to 2.61 ± 4.30, REVEAL 2.0 decreased from 8.74 + 3.02 to 2.34 + 3.81,REVEAL Lite 2 score decreased from 8.20 ± 2.54 to 2.22 ± 3.63 (73% reduction), COMPERA score decreased from 1.86 ± 0.60 to 0.60 ± 0.98 (68% reduction), SPAHR score decreased from 1.69 ± 0.54 to 0.53 ± 0.84 (69% reduction), FPHN high‐risk proportion decreased from 33.8% to 3.6% (89% reduction). Moreover, improvements in each score from baseline to Visit 1, were found to sustain across the all follow‐up visits, indicating effective and durable treatment responses. Our results highlighted the consistency of serial risk assessments with validated MRSs in monitoring the treatment response in patients with PAH. All these MRSs demonstrated concordant improvement from baseline to first follow‐up visit, with 65 to 89% reductions in risk scores maintaining throughout the follow‐up. These results suggest the importance of lower risk status attained at early period with pro‐active combination therapies which might be associated with durability of long‐term clinical benefit and improved survival.

## A132 Pulmonary Artery Stenting in Proximal Chronic Thromboembolic Pulmonary Hypertension – an Evolving Approach

Dr Mike Seckeler^1^, Assistant Professor Michael Insel^1^, Assistant Professor Saad Kubba^1^, Lillian Hansen^2^, Professor Franz Rischard^1^

^1^University Of Arizona/Banner UMC‐Tucson, Tucson, USA, ^2^Banner UMC‐Tucson, Tucson, USA

Chronic thromboembolic pulmonary hypertension (CTEPH) can be treated with balloon pulmonary angioplasty (BPA) of small, distal vessels; larger vessels are treated with pulmonary thromboendarterectomy (PTE). For patients inappropriate for PTE, stenting large proximal vessels could be an alternative. We present three patients who had proximal large vessel stenting.

Patient 1: 75‐year‐old male with completely occluded left lower pulmonary artery (LLPA). It was recanalized and angioplasty performed, but re‐occluded within 2 months. LLPA was recanalized and stented to 9‐mm, serially dilated to 14‐mm in follow‐up procedures and is patent 11 months post‐implantation. Patient 2: 66‐year‐old male with completely occluded LLPA. It was recanalized and angioplasty performed, but re‐occluded within 6 weeks. It was recanalized and stented to 14‐mm when he re‐established care 16 months later. The stent occluded 3 months later due to anticoagulation noncompliance rather than procedural limitations; reintervention is pending consistent anticoagulation therapy. Patient 3: 77‐year‐old male with LLPA branch refractory to angioplasty alone, so was stented to 9‐mm. He had multiple re‐occlusions and each time it was recanalized and distal angioplasty performed with minimal sustained improvement. He had been on apixaban prior to stent placement but had too much bleeding and could not afford it, so was switched to dabigatran. Proximal vessel CTEPH is treated with open surgical PTE, but there may be a role for stenting to decrease morbidity and mortality in select patients. We hypothesize that stents in larger proximal vessels may be less prone to restenosis than smaller distal vessels, where stenting should likely be avoided. Further research is needed to: 1) refine patient selection for stenting, 2) understand the durability of stenting, 3) develop an optimal medical regimen to maintain stent patency, 4) determine if improving proximal stenosis induces growth of distal vessels, as it does in congenital heart disease.

## A133 Metabolomic Profiling Differentiates PAH‐like and ILD‐like Phenotypes in ILD‐PH

Dr Sarah Khan^1^, Matthew Lammi^1^, Sonye Danoff^1^, Aparna Balasubramanian^1^, Todd Kolb^1^, Monica Mukherjee^2^, J. Emanuel Finet^3^, Gabriele Grunig^4^, Christine Jellis^3^, Deborah Kwon^3^, Reena Mehra^5^, Margaret Park^3^, Anna Hemnes^6^, Jane Leopold^7^, Evelyn Horn^8^, Erika Rosenzweig^9^, Franz Rischard^10^, Robert Frantz^11^, Serpil Erzurum^12^, W. H. Wilson Tang^3^, Gerald Beck^12^, Nicholas Hill^13^, Paul Hassoun^1^, Stephen Mathai^1^, Catherine Simpson^1^, PVDOMICS Study Group

^1^Division of Pulmonary and Critical Care Medicine, Johns Hopkins University School of Medicine, Baltimore, USA, ^2^Division of Cardiology, Johns Hopkins University School of Medicine, Baltimore, USA, ^3^Heart, Vascular, and Thoracic Institute, Cleveland Clinic, Cleveland, USA, ^4^New York University Grossman School of Medicine, New York, USA, ^5^Division of Pulmonary, Critical Care, and Sleep Medicine, University of Washington, Seattle, USA, ^6^Division of Allergy, Pulmonary, and Critical Care Medicine, Department of Medicine, Vanderbilt University, Nashville, USA, ^7^Division of Cardiovascular Medicine, Department of Medicine, Brigham and Women's Hospital, Boston, USA, ^8^Division of Cardiology, Department of Medicine, Cornell University Medical Center, New York, USA, ^9^Department of Pediatrics, Westchester Medical Center, New York Medical College, Valhalla, USA, ^10^Division of Pulmonary, Allergy, Critical Care, and Sleep Medicine, University of Arizona College of Medicine, Tucson, USA, ^11^Division of Circulatory Failure, Department of Cardiovascular Medicine, Mayo Clinic, Rochester, USA, ^12^Cleveland Clinic Lerner Research Institute, Cleveland, USA, ^13^Pulmonary, Critical Care, and Sleep Division, Tufts University, Boston, USA

Interstitial lung disease‐associated pulmonary hypertension (ILD‐PH) is a clinically challenging entity that lies on a pathophysiologic spectrum between pulmonary arterial hypertension (PAH) and ILD. We compared the metabolomes of patients with ILD‐PH to those with PAH and ILD to characterize the PAH‐ILD axis and determine whether ILD‐PH patients can be classified based on their metabolomic similarity to PAH. Using untargeted metabolomics profiles from patients with PAH, ILD, and ILD‐PH in the large, U.S.‐based, NIH‐funded, multi‐center PVDOMICS cohort, we trained an anchor‐based classifier on PAH and ILD metabolomes using supervised PLS‐DA with a 75/25 train/test split, then applied the trained model to each ILD‐PH patient to assign a PAH similarity score. We compared PAH metabolomic similarity to expert‐adjudicated primary and secondary WSPH classifications in PVDOMICS. We implemented FELLA, a network‐based metabolomics enrichment method, to holistically characterize the biologic underpinnings of PAH, ILD‐PH, and ILD metabolomes.

We analyzed 244 patients with PAH, 54 with ILD, and 47 with ILD‐PH. The anchor‐based classifier demonstrated high train‐test performance with a c‐statistic of 0.81 and 78% agreement with expert‐adjudicated diagnoses. Among the ILD‐PH patients, 23 were classified as PAH‐like and 24 as ILD‐like (74% agreement with expert adjudication, Hedge's g estimate of 0.86). Patients with a PAH‐like phenotype had a median transplant‐free survival of 212 months compared with 79 months for those with an ILD‐like phenotype (log‐rank p‐value 0.019). FELLA enrichment revealed core glycolytic and bioenergetic pathways were enriched in both PAH and ILD‐PH. Comparison of diffusion Z‐scores between groups demonstrated subtle but biologically coherent differences in several reaction‐level nodes, including lactate, salicylate oxidoreductase, and phosphofructokinase, which were enriched in ILD‐PH. Patients with ILD‐PH demonstrate clinically relevant metabolomic phenotypes. Examination of the network biology underlying ILD‐PH may point to potential therapeutic targets in this vulnerable subgroup with lagging clinical outcomes.

## A134 Advance Extension Interim Analysis: Reveal Lite 2 and Compera 2.0 Risk Improvements With Ralinepag Treatment

Raymond Benza^1^, Cristian Edgardo Botta^2^, Colin Church^3^, Jean Elwing^4^, John Feenstra^5^, M. Patricia George^6^, Lana Melendres‐Groves^7^, Zeenat Safdar^8^, Hugo Hyung Bok Yoo^9^, Dana Cella^10^, Miluska Escudero^10^, Victoria Lacasse^10^, Dr Scott Seaman^10^, Derek Solum^10^, Marc Humbert^11^

^1^Sentara Health, Norfolk, USA, ^2^Hospital Dr. Jose Maria Cullen, Santa Fe, Argentina, ^3^Scottish Pulmonary Vascular Unit & Queen Elizabeth University Hospital, Clydebank, Scotland, ^4^University of Cincinnati College of Medicine, Cincinnati, USA, ^5^Wesley Pulmonary Hypertension Service ‐ Wesley Hospital, Auchenflower, Australia, ^6^National Jewish Health, Denver, USA, ^7^University of New Mexico Department of Medicine, Albuquerque, USA, ^8^Houston Methodist, Houston, USA, ^9^São Paulo State University Medical School, São Paulo, Brazil, ^10^United Therapeutics Corporation, Research Triangle Park, USA, ^11^University Paris‐Saclay, Paris, France

Ralinepag is a titratable, once‐daily, oral prostacyclin agonist in development for treatment of PAH. ADVANCE EXTENSION is an ongoing open‐label extension (OLE) of ADVANCE OUTCOMES, a phase 3 event‐driven study. This interim analysis (May 2025) assesses risk improvements following OLE ralinepag treatment. Only participants experiencing a clinical worsening event in ADVANCE OUTCOMES who enrolled in OLE are included. All OLE participants received ralinepag; treatment in ADVANCE OUTCOMES remain blinded. Participants with ≥ 3 REVEAL Lite 2 parameters, two being 6MWD, WHO FC, or NT‐proBNP, at OLE Baseline were included. For COMPERA 2.0, participants with 6MWD, WHO FC, and NT‐proBNP at OLE Baseline were included. Exposure, AEs, and dosing represent all OLE participants (n = 126). At OLE Baseline (n = 126), 24% were low risk, 25% intermediate risk, and 51% high risk by REVEAL Lite 2 with median (IQR) score of 8 (6,9). At Weeks 16, 28, and 52, 27% (27/100), 31% (28/91), and 31% (22/72) improved risk from OLE Baseline, respectively. At Weeks 16, 28, and 52, 39%, 46%, 44% were low risk, respectively. The median (IQR) score dropped to 6 (4,8) at Week 16 and was maintained through Week 52. By COMPERA 2.0, 4% were low risk, 39% intermediate‐low risk, 48% intermediate‐high risk, and 9% high risk at OLE Baseline (n = 117). At Weeks 16, 28, and 52, 41% (36/87), 42% (36/85), and 45% (29/64) improved risk from OLE baseline, respectively. At Weeks 16, 28, and 52, 22%, 24%, 33% were low risk, respectively.

The median ralinepag dose was 250 mcg at Weeks 16 and 28, and 300 mcg at Week 52; 74% were on dual background therapy. The median exposure in OLE was 67 weeks; safety was consistent with expected prostacyclin effects. Ralinepag treatment in the OLE produced early and sustained improvements in risk. Sponsored by United Therapeutics.

## A135 Interim Analysis of ARTISAN: mPAP Reduction and Right Ventricle Function Improvements with Early Treprostinil Therapy in Pulmonary Arterial Hypertension

Dr Raymond Benza^1^, Dr Kelly Chin^2^, Dr Daniel Lachant^3^, Dr Chad Miller^4^, Dr Robert Naeije^5^, Dr Aaron Waxman^6^, Dr James White^3^, Dr Ronald Zolty^7^, Nancy Daczkowski^8^, Dr Gil Golden^8^, Dr Kaitlyn Paxton^8^, Dr Franck Rahaghi^8^, Dr Yan Liu^8^, Dr Hiromi Matsubara^9^

^1^Sentara Health, Norfolk, United States, ^2^UT Southwestern Medical Center, Dallas, United States, ^3^University of Rochester Medical Center, Rochester, United States, ^4^Piedmont Healthcare, Atlanta, United States, ^5^Free University of Brussels, Brussels, Belgium, ^6^Brigham and Woman's Hospital, Boston, United States, ^7^University of Nebraska College of Medicine, Omaha, United States, ^8^United Therapeutics Corporation, Research Triangle Park, United States, ^9^NHO Okayama Medical Center, Okayama, Japan

Normalization of hemodynamics and improved right heart function are emerging treatment goals in pulmonary arterial hypertension (PAH). The ARTISAN study (NCT05203510) examines the effect of early and rapid treprostinil therapy on mean pulmonary artery pressure (mPAP) reduction to improve right ventricular (RV) function in PAH. ARTISAN is a prospective, multicenter, open‐label study of early initiate parenteral treprostinil with rapid dose titration to achieve mPAP of < 35 mmHg followed by potential transition to oral treprostinil. The CardioMEMS™ HF System enables remote monitoring of mPAP. This interim analysis conducted in August 2025 presents baseline and 12‐month data for mPAP, cMRI parameters, and NT‐proBNP. Variables are reported as mean (SD) unless denoted otherwise, with P values calculated using appropriate statistical methods. Of fifty‐two participants enrolled in ARTISAN, eighteen participants with centrally reviewed cMRI data were included in this interim analysis. Time since PAH diagnosis to treprostinil initiation was 3 (2) months, combined with ERA and/or PDE‐5 inhibitors therapy. The baseline REVEAL Lite 2 risk score was 7 (3) and mPAP was 52 (12)mmHg. At month 12, parenteral treprostinil dose was 91 (19)ng/kg/min (n = 9), while the oral treprostinil dose was 27 (10)mg total daily dose (n = 9). From baseline to month 12, significant hemodynamic and cardiac improvements were observed: mPAP decreased by 13 (11)mmHg (p = 0.0002), cardiac output increased by +1.0 (1.3)L/min (p = 0.0038), and RV stroke volume index increased by +8 (7)mL/m2 (p = 0.0001). RV Mass Index decreased ‐0.4 (8.0)g/m2 (p = 0.8297), and median (IQR) NT‐proBNP decreased by ‐580 (‐2503, ‐60)pg/mL (p = 0.0105). Six participants died, none related to drug or protocol. Preliminary data from ARTISAN suggest that early initiation of high‐dose treprostinil therapy represents a feasible strategy for reducing mPAP and improving RV structure and function in PAH. Treating to remission and reverse remodeling may be an achievable goal. Sponsored by United Therapeutics.

## A136 The Humanistic and Economic Burden of Pulmonary Arterial Hypertension in Latin America and the Caribbean: A Systematic Literature Review

Dr Victoria Wurcel^1^, Natalia Tassara^1^, Amanda Bittencour^2^, Haliton Alves^2^, Hafez Antonio^3^, Claudia Folino^1^, Jose Rincon^3^, Felype Castelhano^2^, Ashley Humphries^4^, Ina Zile^4^, Amy Cooper^4^, Dr Jacqueline Pavia

^1^MSD Argentina, Buenos Aires, Argentina, ^2^MSD Brazil, São Paulo, Brazil, ^3^MSD Mexico, Ciudad de Mexico, Mexico, ^4^Adelphi Values PROVE, Bollington, United Kingdom

Pulmonary arterial hypertension (PAH) is a chronic, progressive rare disease characterized by narrowing blood vessels in the lungs increasing arterial pressure and straining the heart. This systematic literature review (SLR) analyzed health related quality of life (HRQoL) and economic burden of PAH in Latin American and Caribbean (LatAm) population. Systematic literature searches were conducted in April 2025 across multiple databases, congresses, and grey literature in English, Portuguese, and Spanish to identify all relevant literature on PAH in LatAm. References were independently screened and subsequently extracted by two reviewers. The SLR identified 42 studies, with high HRQoL burden across LatAm: mean EQ‐5D‐5L scores for ARG, COL and BRA (0.7), Mexico (0.67). EQ‐5D‐VAS scores worsened with increasing World Health Organization Functional Class (WHO FC). Patients with higher WHO FC had greater healthcare resource utilization (mean annual per patient hospitalization days 0.2 in WHO FC I vs 1 in WHOFC IV; mean ICU length of stay 4.5 days in WHO FC III vs 12 in WHO FC IV), greater overall costs (USD 37,335 in WHO FC I vs 75,747 in WHO FC IV in Colombia), and PAH‐specific treatment costs. In Brazil, 85.2% of PAH patients reporting loss of productivity and associated earnings. Monotherapy was used in 72% of patients, combination was more likely in patients with higher WHO FC, or intermediate to high risk of mortality. Patients across LatAm received limited treatment beyond double therapy, contrary to international clinical guidelines. This study demonstrates the high economic and humanistic burden of PAH in LatAm, a region facing challenges in treatment availability and accessibility.

## A137 PHELEX: Pulmonary Hypertension Engine for Linked Experiments – A Web Application to Explore, Mine and Analyze Publicly Available Pulmonary Hypertension ‘Omics Datasets

Miss Tanvi Nandani^1^, Mr Benjamin P. Ott^2^, Miss Prusha Balaratnam^1^, Dr. Stephen L. Archer^1,2^, Dr. Charles C.T. Hindmarch^1,2,3^, Miss Tanvi Sandip Nandani

^1^Department of Medicine, Queen's University, Kingston, Canada, ^2^Queen's CardioPulmonary Unit, Queen's University, Kingston, Canada, ^3^Department of Biomedical and Molecular Sciences, Queen's University, Kingston, Canada

Pulmonary hypertension (PH) is a heterogeneous group of disorders characterized by an elevated mean pulmonary arterial pressure above 20 mmHg, leading to dyspnea, progressive right heart failure, and increased mortality. While treatments reduce symptoms and may improve survival, PH remains progressive and life‐threatening, decreasing quality of life while creating a significant economic burden for the patient and society. To develop a cure, better molecular understanding of PH is required. Efforts to understand the molecular processes driving PH have expanded through large‐scale “omics” data. The Gene Expression Omnibus (GEO) currently hosts 1,165 records related to all forms of pulmonary hypertension with 5,765 unique samples (2,282 microarray, 2,535 bulk and 154 single cell sequencing, 466 protein profiling, and 129 genomic samples) from 8 species. The cost of profiling the biological samples using high‐throughput technologies currently stands at around $1,000/sample. We estimate that >$5.76 million has been spent on generating these data. These data sets are often used for exploration of a single molecular pathway, whereas they could be extensively re‐mined to address diverse questions. Making these data more accessible requires bioinformatics expertise, software tools, and data‐processing pipelines. To address this, we created PHELEX, a web‐based R Shiny application with a searchable dashboard that allows users to search and filter GEO data by sequencing platform, species, experiment and sample type, and view its associated metadata in a clear and accessible format. The dashboard integrates with an RNA Sequencing Analysis software that we have built, called Confidence© which enables differential gene expression and pathway analysis with DESeq. 2, NOISeq, edgeR, or Limma, allowing users to visualize and download analyzed data files. With PHELEX, which currently houses 110 terabytes of data related to PH (Groups 1–5), scientists can access and analyze PH datasets from GEO within a single application, democratizing public data and accelerating discovery.

## A138 Real‐World Safety Profile of Treprostinil Inhalation Solution (Tyvaso) for Pulmonary Arterial Hypertension Compared to Results from the INSPIRE Trial

Dr Astrid Kugener^1,2^, Shruti Lopchuk^2^, Frances Chaar‐Alvarez^2^, Vijay Subramanian^2^, Claire Thrasher^2^

^1^University Of North Carolina, Chapel Hill, Chapel Hill, USA, ^2^United Therapeutics, Research Triangle Park, USA

Inhaled nebulized treprostinil solution (Tyvaso) is approved for use in patients with pulmonary arterial hypertension (PAH). Yutrepia is treprostinil dry powder inhaler recently approved for patients with PAH based on evidence from the INSPIRE trial. Ongoing comparative assessments of available therapies are essential for ensuring patient safety and optimal treatment. To evaluate the safety profile of nebulized Tyvaso from post‐marketing data in comparison to clinical trial evidence of Yutrepia from the INSPIRE trial. An internal retrospective review of the United Therapeutics Corporation safety database from January 1st, 2024 to December 31st, 2024, was performed to identify adverse events experienced by patients receiving nebulized Tyvaso for an indication of PAH. The most common adverse events from the INSPIRE trial, defined as those considered related to Yutrepia and reported in ≥ 10% of patients, were assessed. A total of 4,138 solicited and spontaneous patient cases were identified with nebulized Tyvaso within the safety database and were compared to those reported from the 121 participants of the INSPIRE trial. Dose‐agnostic adverse event rates for nebulized Tyvaso vs Yutrepia, respectively, were 32% vs 53% for cough, 8% vs 18% for diarrhea, 22% vs 19% for dyspnea, 20% vs 34% for headache, 3% vs 10% for nasopharyngitis, 8% vs 10% for nausea, and 9% vs 18% for throat irritation. These post‐marketing real‐world data suggest favorable safety and tolerability with nebulized Tyvaso compared to available clinical trial evidence for Yutrepia in PAH. The occurrence of adverse events identified in the safety database is consistent with established labeling for nebulized Tyvaso and is under continuous surveillance.

## A139 Real‐World Safety Profile of Treprostinil Dry Powder Inhaler (Tyvaso DPI) for Pulmonary Arterial Hypertension Compared to Results From the INSPIRE Trial

Dr Astrid Kugener^1,2^, Shruti Lopchuk^2^, Frances Chaar‐Alvarez^2^, Vijay Subramanian^2^, Claire Thrasher^2^

^1^University Of North Carolina, Chapel Hill, Chapel Hill, USA, ^2^United Therapeutics, Research Triangle Park, USA

Treprostinil dry powder inhaler (Tyvaso DPI) is approved for use in patients with pulmonary arterial hypertension (PAH). Yutrepia is another treprostinil DPI recently approved for patients with PAH based on evidence from the INSPIRE trial. Ongoing comparative assessments of available therapies are essential for ensuring patient safety and optimal treatment. To evaluate the safety profile of Tyvaso DPI from post‐marketing data in comparison to clinical trial evidence of Yutrepia from the INSPIRE trial. An internal retrospective review of the United Therapeutics Corporation safety database from January 1st, 2024 to December 31st, 2024, was performed to identify adverse events experienced by patients receiving Tyvaso DPI for an indication of PAH. The most common adverse events from the INSPIRE trial, defined as those considered related to Yutrepia and reported in ≥ 10% of patients, were assessed. A total of 5,964 solicited and spontaneous patient cases were identified with Tyvaso DPI within the safety database and were compared to those reported from the 121 participants of the INSPIRE trial. Dose‐agnostic adverse event rates for Tyvaso DPI vs Yutrepia, respectively, were 43% vs 53% for cough, 9% vs 18% for diarrhea, 21% vs 19% for dyspnea, 22% vs 34% for headache, 3% vs 10% for nasopharyngitis, 8% vs 10% for nausea, and 11% vs 18% for throat irritation. These post‐marketing real‐world data suggest favorable safety and tolerability with Tyvaso DPI compared to available clinical trial evidence for Yutrepia in PAH. The occurrence of adverse events identified in the safety database is consistent with established labeling for Tyvaso DPI and is under continuous surveillance.

## A140 Cell Type‐Specific Function of SPARC in Chronic Hypoxia‐Induced Pulmonary Hypertension

Dr Christine Veith^1^, M.Sc. Vanessa Nebel^1^, DVM Simone Kraut^1^, PhD Magdalena Wujak^1^, M.Sc. Anis Čilić^1^, PhD Stefan Hadžić^1^, DVM Kathrin Malkmus^1^, M.Sc. Edma Loku^1^, PhD Cheng‐Yu Wu^1^, MD Werner Seeger^1^, MD, PhD Natascha Sommer^1^, PhD Grażyna Kwapiszewska, PhD Norbert Weissmann^1^

^1^Justus‐liebig University Giessen, Giessen, Germany

Pulmonary hypertension (PH) is a life‐threatening, devastate disease, characterized by obliteration of small pulmonary vessels due to pulmonary vascular remodeling, leading to elevated pulmonary arterial pressure and right heart hypertrophy. PH can be caused by chronic hypoxia, leading to hyper‐proliferation of pulmonary arterial smooth muscle cells (SMC) and apoptosis‐resistant pulmonary endothelial cells (EC). Previously, we provide evidence that Secreted protein acidic and rich in cysteine (SPARC) is elevated expressed in lungs from chronic hypoxic mice and patients with idiopathic pulmonary arterial hypertension (IPAH). Immunostainings primarily localized SPARC in the SMC and EC layer of the pulmonary vessel wall. In in vivo experiments, AAV‐mediated Sparc knockdown led to significantly improved hemodynamic and cardiac function in PH mice.

In this study, we aim to unravel the cell type‐specific in vitro and in vitro function of SPARC in chronic hypoxia‐induced PH pathogenesis. In vivo, tamoxifen‐inducible Sparc knockdown was induced in Acta2‐Cre/ERT2_tomato (SMC) or Tek‐Cre/ERT2 (EC) mice. Surprisingly, Sparc knockdown in Tek‐Cre/ERT2 mice improved hemodynamic and cardiac function in PH mice, whereas knockdown in Acta2‐Cre/ERT2_tomato mice did not partially protect from PH. In in vitro experiments, SPARC expression was induced by hypoxia/hypoxia‐inducible factor (HIF)‐2A or transforming growth factor (TGF)‐β1 in pulmonary arterial SMC but not in pulmonary EC. In loss of function experiments, SPARC silencing enhanced apoptosis and reduced proliferation in both cell types. In conclusion, our study provides evidence for a cell type‐specific function of SPARC in PH pathogenesis, most likely by different underlying molecular mechanisms.

## A141 Genomic Determinants of Therapy Response in Pulmonary Veno‐Occlusive Disease: Insights From Whole‐Genome Sequencing and GWAS

Dr Natalia Gallego‐zazo^1,2,3,4^, Dr. Lucia Miranda‐Alcaraz^1,2,3^, Dr. Monica Mora‐Gómez^1,2,3^, Dr. Alejandro Cruz‐Utrilla^5,6,7^, Dr. Maria Jesús Del Cerro Marín^8^, Organization Spanish PAH Consortium Spanish PAH Consortium^1,2,3,5^, Dr. Nuria Ochoa Parra^5,6,7^, Dr. Irene Martín de Miguel^5,6,7^, Dr. Eva Guitiérrez Ortiz^5,6,7^, Dr. Juan Andrés Jiménez‐Estrada^1,2,3^, Dr. Alejandro Parra^1,2,3^, Dr. Mario Cazalla^1,2,3^, Dr. Sergio Ramos^1,2,3^, Dr. Manuel Rodríguez‐Canó^1,2,3^, Dr. Cristina Silván Fuentes^1,2,3^, Dr. Valeria Vásquez‐Amell^1,2,3^, Dr. Pedro Arias Lajara^1,2,3^, Dr. Julián Nevado Blanco^1,2,3^, Dr. Vinicio de Jesús Pérez^9^, Dr. Pablo Lapunzina^1,2,3,4^, Dr. Pilar Escribano Subias^5,6,7^, Dr. Carlos Flores^2,10^, Dr. Jair Tenorio Castaño^1,2,3^

^1^Instituto de Genética Médica y Molecular (INGEMM), Instituto de Investigación del Hospital Universitario La Paz (IdiPaz), Hospital Universitario La Paz, Madrid, Spain, ^2^CIBERER, Centro de Investigación Biomédica de Enfermedades Raras en Red, Instituto de Salud Carlos III, Madrid, Spain, ^3^ERN‐ITHACA, European Reference Network on Rare Malformations Syndromes, Intellectual and Other Neuro‐Developmental Disorders, Paris, France, ^4^Escuela de Doctorado, Universidad Autónoma de Madrid, Madrid, Spain, ^5^Unidad Multidisciplinar de Hipertensión Pulmonar, Servicio de Cardiología, Hospital Universitario 12 de Octubre, Madrid, Spain, ^6^ERN‐LUNG, European Reference Network on Rare Lung Diseases (Pulmonary Hypertension), Frankfurt am Main, Germany, ^7^CIBERCV, Centro de Investigación Biomédica en Red de Enfermedades Cardiovasculares, Instituto de Salud Carlos III, Madrid, Spain, ^8^Unidad de Hipertensión Pulmonar Pediátrica, Servicio de Cardiología Pediátrica, Hospital Universitario Ramón y Cajal, Instituto de Investigación Biomédica del Hospital Universitario Ramón y Cajal (Irycis), Madrid, Spain, ^9^Department of Medicine, Stanford University School of Medicine, Stanford, California, USA, ^10^Hospital Universitario Nuestra Señora de la Candelaria, Santa Cruz, Spain

Pulmonary veno‐occlusive disease (PVOD) is a rare subtype of pulmonary arterial hypertension (PAH) with distinct clinical, histological, and genetic features, often misdiagnosed as idiopathic PAH. Its hallmark is intimal fibrosis of pulmonary venules, leading to luminal obstruction, severe hypoxemia, and unpredictable response to PAH‐specific therapies. Definitive diagnosis requires histology or detection of EIF2AK4 mutations, though high‐probability diagnosis relies on clinical, radiological, and functional findings. PVOD carries a particularly poor prognosis, with survival markedly shorter than PAH. Response to therapy is heterogeneous: while some patients show transient benefit, others develop pulmonary edema and rapid deterioration. A founder mutation (EIF2AK4:c.3344 C > T;p.Pro1115Leu) was identified in gipsy PVOD patients, revealing strikingly different outcomes despite the same genotype, underscoring the need for biomarkers of treatment response. Genetic association studies, including GWAS, may provide such predictors and enable precision medicine. Based on these observations, our objective was to conduct a comprehensive genomic association study (GWAS) of treatment response in patients with PVOD. Specifically, we aimed to compare individuals who tolerate PAH‐specific therapy with those who are intolerant, in order to identify potential genetic determinants of therapeutic response. To this end, we performed whole‐genome sequencing (WGS) in a cohort of 50 PVOD patients for whom detailed clinical data on vasodilator response had been systematically collected. Using genome‐wide association study approaches, we investigated whether specific genetic variants could serve as biomarkers to predict treatment response in this highly vulnerable and clinically challenging patient population. We propose a poster presentation to communicate the preliminary results of the studies currently being conducted. Identifying such biomarkers would represent a major step toward precision medicine in PVOD, enabling early stratification of patients and guiding therapeutic decision‐making to improve outcomes.

## A142 Detection of Cnvs Through Whole Genome Sequencing in Patients With Pulmonary Arterial Hypertension

Ms Mónica Mora‐Gómez^1,2,3,4^, MSc Lucía Miranda‐Alcaraz^1,2,3,4^, PhD Natalia Gallego‐Zazo^1,2,3^, MD Alejandro Cruz‐Utrilla^5,6,7^, MD María Jesús del Cerro‐Marín^8^, MD Spanish PAH Consortium Spanish PAH Consortium^1,2,3,5^, PhD Juan Andrés Jiménez‐Estrada^1,2,3^, MSc Tomás Valle^1,2,3^, MSc Mario Cazalla^1,2,3,4^, MSc Manuel Rodríguez‐Canó^1,2,3,4^, Ms Cristina Silván^1,2,3^, Mr Pedro Arias^1,2,3^, MD Valeria Vásquez‐Amell^1,2,3^, PhD Julián Nevado^1,2,3^, MD Pilar Escribano‐Subías^5,6,7^, MD Pablo Lapunzina^1,2,3^, PhD Jair Tenorio‐Castano^1,2,3^

^1^Institute of Medical and Molecular Genetics (INGEMM), La Paz University Hospital, Madrid, Spain, ^2^Network Centre for Biomedical Research on Rare Diseases (CIBERER), Carlos III Health Institute, Madrid, Spain, ^3^European Reference Network on Rare Malformations Syndromes, Intellectual and Other Neuro‐Developmental Disorders (ERN‐ITHACA), Paris, France, ^4^Doctoral School, Autonomous University of Madrid, Madrid, Spain, ^5^Multidisciplinary Pulmonary Hypertension Unit, Cardiology Department, 12 de Octubre University Hospital, Madrid, Spain, ^6^European Reference Network on Rare Lung Diseases (Pulmonary Hypertension) (ERN‐LUNG), Frankfurt, Germany, ^7^Network Centre for Biomedical Research on Cardiovascular Diseases (CIBERCV), Carlos III Health Institute, Madrid, Spain, ^8^Pediatric Pulmonary Hypertension Unit, Pediatric Cardiology Department, Ramón y Cajal University Hospital, Ramón y Cajal University Hospital Biomedical Research Institute (Irycis), Madrid, Spain

Pulmonary arterial hypertension (PAH) is a severe disease characterized by elevated pulmonary artery pressure, leading to heart failure and premature death if untreated. Genetic factors significantly contribute to PAH, and several genes have been linked to its development. The aim of this study was to identify copy‐number variation (CNV) in PAH‐related genes in patients with idiopathic or heritable PAH and a previous non‐conclusive genetic testing through whole exome sequencing. We analyzed a cohort of 158 individuals (101 PAH patients and 57 relatives) using whole genome sequencing (WGS). Variants were prioritized through a custom pipeline and classified according to ACMG guidelines. Nine CNVs were observed in nine patients, in coding and non‐coding region. Seven variants were identified in genes with definitive evidence: two duplications and four deletions in BMPR2, and one duplication in TBX4 in a patient with small patella syndrome. The remaining two were duplications observed in genes whose relationship with PAH is currently disputed: BMPR1A and BMPR1B. These results show the improvement in accuracy when analyzing CNVs by performing whole genome sequencing, enabling the diagnosis of 8.9% more patients.

## A143 DNA Methylation Signature in Pulmonary Arterial Hypertension, A Potential Molecular Diagnostic Tool

Dr Jair Tenorio^1,2,3,4^, PhD Natalia Gallego^1,2,3^, Mr Mario Cazalla‐Garcia^1,2,3^, Ms Lucia Miranda^1,2,3^, Ms Mónica Mora^1,2,3^, MD Alejando Cruz‐Utrilla^6^, Ms Nuria Ochoa^6^, Mr Manuel Jesús Rodriguez Canó^1,2,3^, Mr Sergio Ramos^1,2,3^, Ms Valeria Vazquez^1,2,3^, Mr Pedro Arias^1,2,3^, Ms Cristina Silvan^1,2,3^, MD, PhD María Jesús Del Cerro^1,2,3^, PhD Mathis Hildonen^1,2,3^, Mr Spanish PAH Consortium^1,2,3^, Mr METILPREC Consortium^1,2,3^, PhD Juan Andrés Estrada Jimenez^1,2,3^, MD Tomás Lopez‐Valle^1,2,3^, PhD Zeynep Tuemer^8^, PhD Julian Nevado^1,2,3,4^, MD Vinicio De Jesús Pérez^9^, PhD Pablo Lapunzina^1,2,3,4^, MD Pilar Escribano^6^

^1^Institute of Medical and Molecular Genetics (INGEMM) ‐ HOSPITAL UNIVERSITARIO LA PAZ, MADRID, Spain, Madrid, Spain, ^2^CIBERER, Madrid, Spain, ^3^ERN‐ITHACA, Belgium, Brussels, ^4^Universidad Camilo José Cela, Madrid, Spain, ^5^METILPREC Consortium, Madrid, Spain, ^6^Hospital Universitario 12 de Octubre, Madrid, Spain, ^7^Hospital Universitario Ramón y Cajal, Madrid, Spain, ^8^Department of Clinical Genetics, Kennedy Center, Copenhagen University Hospital, Copenhagen, Denmark, ^9^Stanford School of Medicine, Stanford University, Palo Alto, USA

Pulmonary arterial hypertension (PAH) is a rare, life‐threatening disorder characterized by increased pulmonary arterial pressure, leading to right heart failure and potential mortality. The incidence ranges from 15 to 50 cases per million annually. Clinical symptoms include fatigue, syncope, chest pain, and edema. While PAH has a multifactorial etiology, genetic and epigenetic contributions play a critical role in its pathogenesis. The diagnostic utility of DNA methylation (DNAm) patterns has emerged as a novel approach to classify and validate genetic variants of uncertain significance (VUS), particularly in genes like BMPR2, SOX17, and EIF2AK4. Using Illumina 950 K Methylation Microarray, we analyzed DNAm profiles from 23 patients with pathogenic variants in BMPR2 and 41 controls. Differentially methylated probes (DMPs) were identified, and a support vector machine (SVM) model was employed for classification. We identified 842 DMPs, with 752 hypomethylated and 90 hypermethylated CpG sites, predominantly outside well‐known PAH‐associated genomic regions. PCA revealed distinct DNAm profiles between patients and controls. SVM analysis achieved 90% specificity and 100% sensitivity in differentiating patients based on DNAm patterns, with promising results for subclassification among other PAH‐related genes. DNAm signatures offer a robust tool for validating VUS in PAH‐associated genes, improving diagnostic accuracy. Preliminary data highlight a unique epigenetic signature for BMPR2, distinct from SOX17 and EIF2AK4. Further validation with external cohorts and diverse gene sets is essential to enhance the utility of DNAm profiling as a molecular diagnostic adjunct in PAH.

## A144 Notch Transcription Factor Rbpj Modulates Smooth Muscle Cell Function in Pulmonary Hypertension

Ms Vanessa Nebel^1^, Anis Čilić^1^, Dr. Stefan Hadžić^1^, Akylbek Sydykov^1^, Dr. Benedetto Daniele Giaimo^2^, Benedikt Jenuwein^3^, Dr. Cheng‐Yu Wu^1^, Dr. Oleg Pak^1^, Prof. Dr. Natascha Sommer^1^, Prof. Dr. Marek Bartkuhn^3^, Prof. Dr. Norbert Weissmann^1^, Prof. Dr. Tilman Borggrefe^2^, Dr. Christine Veith^1^

^1^Universities of Giessen and Marburg Lung Center (UGMLC), Member of the German Center for Lung Research (DZL), Excellence Cluster Cardio‐Pulmonary Institute (CPI), Justus‐Liebig University, 35392 Giessen, Germany, ^2^Institute of Biochemistry, Justus‐Liebig University, 35392 Giessen, Germany, ^3^Institute for Lung Health (ILH), Biomedical Informatics and Systems Medicine, Justus‐Liebig University, 35392 Giessen, Germany

Pulmonary hypertension (PH) is a severe, life‐threatening disease, characterised by increased pulmonary vascular resistance and pulmonary arterial pressure, mainly caused by pulmonary vascular remodelling. Chronic pressure overload of the right ventricle leads to right heart hypertrophy, which can progress to right ventricular failure and death if left untreated. Chronic alveolar hypoxia significantly contributes to pulmonary vascular remodelling by, among other mechanisms, triggering the uncontrolled proliferation of pulmonary arterial smooth muscle cells (PASMC), ultimately resulting in the obliteration of small pulmonary arteries. The evolutionarily conserved Notch signalling pathway plays a crucial role in vascular development and homeostasis by regulating various aspects of smooth muscle cell behaviour. While the involvement of Notch receptors in PH pathogenesis is well examined, the role of downstream Notch signalling remains unclear. In this context, we aim to investigate whether the Notch transcription factor Recombination Signal Sequence‐Binding Protein Jκ (RBPJ) contributes to smooth muscle cell dysfunction in the pathogenesis of PH. Our in vivo data from mice with a tamoxifen‐inducible Rbpj knockout in Acta2+ cells indicate partial protection against chronic hypoxia‐induced pulmonary hypertension, as evidenced by reduced right ventricular systolic pressure, attenuated right heart hypertrophy and improved right heart function. Remarkably, loss of RBPJ almost completely prevents pulmonary vascular remodelling in these mice. In in vitro studies using primary human PASMC, RBPJ was identified as a regulatory factor affecting both proliferation and apoptosis. RNA‐Seq analysis revealed that loss of RBPJ predominantly affects PASMC metabolism, which may account for its impact on multiple signalling pathways implicated in PH, including hypoxia‐inducible factor (HIF) and endothelin‐1 (ET‐1). In conclusion, our data provide the first evidence that RBPJ contributes to chronic hypoxia‐induced pulmonary hypertension in mice by modulating smooth muscle cell function.

## A145 Utility of Electrocardiography in Screening for Patients With ILD‐Associated Pulmonary Hypertension

Dr Bismah Khan, Miss Cordelia Leff, Dr Sophie Herbert, Mr Carl Harries, Dr Colm McCabe, Dr Heba Nashat, Professor S John Wort, Professor Konstantinos Dimopoulos, Dr Laura Price^1^

^1^Royal Brompton Hospital, London, United Kingdom, ^2^Imperial College London, London, United Kingdom

Prior to ILD‐PH diagnosis, several non‐invasive parameters prompt referral for echocardiography and right heart catheterization (RHC) (1). ECG is an inexpensive safe tool to potentially contribute to the screening process, as suggested in patients with PAH (2). From the PULSE ILD‐PH registry, 12‐lead ECG were analyzed for right heart signs in patients with RHC‐confirmed ILD‐PH (mPAP> 20 mmHg) vs controls (ILD without PH, mPAP≤ 20 mmHg). Data analysis used R Studio. Of 110 patients, 86 had PH and 24 had no PH (controls). Median age was 59.0 (IQR 49.2‐67.0) years and 40 (36.4%) were male. At ECG, patients with ILD‐PH had right‐ventricular hypertrophy (RVH) in 28 (32.6%) vs 0 (0.0%) in controls (p = 0.003); right‐axis deviation (RAD) in 22 (25.6%) in PH vs 0 (0.0%) in controls (p = 0.003); right bundle‐branch block (RBBB): 13 (15.1%) in PH vs 3 (12.5%) in controls (p = 1.000); p pulmonale: 36 (41.9%) in PH vs 0 (0.0%) in controls (p < 0.001). Among ILD‐PH patients, one ECG sign was present in 15 (17.4%), two in 11 (12.8%), and ≥ 3 in 19 (22.1%). On logistic regression, C‐statistics were: RVH 0.663 (95% CI 0.613–0.713), RAD 0.628 (95% CI 0.582–0.674), RBBB 0.513 (95% CI 0.436–0.591), and P pulmonale 0.709 (95% CI 0.657–0.762). Only parameters with an AUC > 0.60 were considered (Composite built from: RVH, RAD, P pulmonale (AUC > 0.60).). The C‐statistic for patients having ≥ 1 of these parameters was 0.750 (95% CI 0.697–0.803), for ≥ 2 of the parameters 0.669 (95% CI 0.618–0.719), and for all 3 parameters 0.581 (95% CI 0.542–0.621). For patients with ≥ 1 of the selected ECG parameters, the sensitivity for PH was 50.0%, specificity 100.0%, PPV 100.0%, and NPV 35.8%. The presence of any right heart signs on ECG should prompt assessment with echocardiography, and referral to a PH centre for confirmation of ILD‐PH diagnosis.

## A146 Sotatercept in Pediatric Pulmonary Hypertension: Real World Experience From the PPHNet

MD, PhD Paul J Critser^1^, Nidhy P Varghese^2^, Dr Usha Krishnan^3^, MD MSc Eric. D Austin^4^, Rachel T Sullivan^5^, PharmD Meredith J. O'Neil^1,6^, MD Benjamin. S Frank^7^, Dunbar. D Ivy^7^, MD Catherine M. Avitabile^8^, MD, MSCE Andrea L. Jones^8^, PharmD, BCPPS Neelam D. Bhatt^9^, MD Delphine Yung^10^, Claire Parker^11^, Stephanie Handler^12^, DO Edward Kirkpatrick^12^, MD Patric D Evers^13^, MD Emily. P. Williams^14^, Megan Griffiths^15^, MD, PhD Mary P. Mullen^16^

^1^The Heart Institute, Cincinnati Children's Hospital Medical Center University of Cincinnati College of Medicine, Cincinnati, United States ‐ HIC, ^2^Department of Pediatrics, Division of Pulmonology, Baylor College of Medicine and Texas Children's Hospital, Houston, United States ‐ HIC, ^3^Division of Pediatric Cardiology, Department of Pediatrics, Columbia University Irving Medical Center, New York, United States ‐ HIC, ^4^Vanderbilt University Medical Center, Monroe Carrell Jr Children's Hospital, Nashville, United States ‐ HIC, ^5^Department of Pediatrics, Division of Cardiology, Vanderbilt University Medical Center, Monroe Carrell Jr Children's Hospital, Nashville, United States ‐ HIC, ^6^Division of Pharmacy, Cincinnati Children's Hospital Medical Center, Cincinnati, United States ‐ HIC, ^7^University of Colorado school of medicine, department of pediatrics, section of cardiology, Denver, United States ‐ HIC, ^8^Division of Pediatric Cardiology, The Children's Hospital of Philadelphia University of Pennsylvania Perelman School of Medicine, Philadelphia, United States ‐ HIC, ^9^Department of Pharmacy, Texas Children's Hospital, Houston, United States ‐ HIC, ^10^Department of Pediatrics, University of Washington School of Medicine, Seattle, United States ‐ HIC, ^11^Department of Nursing, University of California, San Francisco Benioff Children's Hospital, San Francisco, United States ‐ HIC, ^12^Children's Wisconsin, Medical College of Wisconsin, Milwaukee, United States ‐ HIC, ^13^Division of Pediatric Cardiology, Oregon Health and Sciences University, Portland, United States ‐ HIC, ^14^Children's Healthcare of Atlanta Cardiology, Emory University School of Medicine, Atlanta, United States ‐ HIC, ^15^University of Texas Southwestern, Dallas, United States ‐ HIC, ^16^Boston Children's Hospital, Harvard Medical School, Boston, United States ‐ HIC, ^17^Maria Fareri Children's Hospital, Department of Pediatrics at New York Medical College, Valhalla, United States ‐ HIC

Sotatercept, a ligand trap for TGF‐β family members, has been shown to improve exercise capacity and decrease pulmonary vascular resistance in adults with pulmonary arterial hypertension. There are currently limited data describing the use of sotatercept in children with pulmonary hypertension (PH). This is a multi‐center, retrospective cohort study of children and young adults ≤ 21 years of age with PH enrolled in the Pediatric PH Network (PPHNet) registry. Demographic data and clinical characteristics before and after sotatercept initiation were collected. Sixty‐one subjects received sotatercept as add on therapy for a median duration of 213 days (IQR 103, 238). Thirty‐six (59%) were female with median age 15.3 years (range 7.0 – 18.0) with Group 1 PH in 56 (92%), Group 3 PH in 10 (15%), and Group 5 PH in 1 (2%). WHO functional class improved in 51% and did not decrease in any subjects. Improvements were seen in NT pro‐BNP (‐394 pg/ml [IQR 18, 942], n = 25, p < 0.0001), six‐minute walk distance (32 meters [IQR ‐5, 77], n = 27, p < 0.0001), mean pulmonary artery pressure (‐18 mmHg [IQR ‐21, ‐14], n = 12, p = 0.0005, and pulmonary vascular resistance (‐4.9 WUi [IQR ‐10.1, ‐3.3], n = 12, p = 0.0005). Parenteral prostacyclin dose was reduced in 3 patients and transitioned to oral or inhaled prostacyclin in another 3 patients. Adverse events occurred in 28 (46%) of subjects with bleeding in 11 (18%), including one who was hospitalized for gastrointestinal bleed. Dose reduction or dose delay occurred in 9 (15%) and 8 (13%) patients respectively, with most commonly due to elevated hemoglobin. Two patients discontinued therapy. No patients were hospitalized for clinical worsening, underwent lung transplant, or died. Pediatric PH patients treated with sotatercept as add on therapy demonstrated improvement in functional class and other PH disease indicators. Sotatercept was generally well tolerated in children with PH.

## A147 Serial Pulmonary Artery Pulsatility and Compliance as Simple and Reproducible Echocardiographic Parameters to Guide Management in Pediatric Pulmonary Arterial Hypertension

Dr Usha Krishnan^1^, Dr. Katelyn Benson, Dr. Yun Zhang

^1^Columbia University Medical Center, New York, United States, ^2^Columbia University Medical Center, New York, United States, ^3^Columbia University Medical Center, New York, United States

Hypothesis: Serial pulmonary artery (PA) measurement by echocardiogram is an easily reproducible noninvasive parameter that can be trended over time to guide management of pulmonary arterial hypertension (PAH). Data from 13 patients with PAH ages (3 months–18 years) seen between 2013‐2025 were collected by retrospective chart review. Main and right PA (MPA, RPA) dimensions, pulsatility, compliance, and indirect measures of PAH severity were measured on echocardiograms at 4 time points (with corresponding hemodynamic studies) at baseline, 6‐12 months of therapy, escalation of disease and most recent visit. Largest and smallest MPA and RPA diameters in a cycle were charted and converted to Z‐scores using Boston Z‐score calculator. PA compliance and pulsatility were calculated. These were correlated with concurrently documented clinical status, WHO FC, hemodynamic data, exercise parameters and biomarkers (NTproBNP). There are statistically significant differences in PVR and PVR/SVR ratio across the 4 time points. Post‐hoc pairwise comparisons showed PVR significantly decreased from Time 1 to Time 2 (median change: ‐4.36 (‐16.24, ‐1.5); p = 0.01), and then increased from Time 2 to Time 3 (median change: 6.07 (4.3, 16); p < 0.05). PVR/SVR ratio significantly decreased from Time 1 to both Time 2 (median change: ‐0.39 (‐0.63, ‐0.11); p = 0.03) and Time 4 (median change: ‐0.36 (‐0.68, ‐0.1); p = 0.03). On average, a one‐unit increase in compliance of the MPA was associated with a 0.11‐unit decrease in PVR (p = 0.01). Other parameters of pulsatility trended along hemodynamics, and further analysis and larger numbers may provide more statistical significance. These preliminary data suggest that PA size, pulsatility and compliance correlate with hemodynamic, biomarker, and functional data in children with PAH. Echocardiographic evaluation of PA pulsatile function potentially represents a simple, reproducible and non‐invasive metric to assist in managing these complex patients and provide additional information on response to therapies.

## A148 Cardiopulmonary Exercise Testing in Pulmonary Hypertension: An Audit of Clinical Practice and Outcomes in Sheffield Pulmonary Vascular Disease Unit

Dr Ellis Cerrone, Dr Abdul Hameed, Dr Hamza Zafar, Dr Rosie Simmons^1^, Dr Athanasios Charalampopoulos^1^, Dr Sami Kimyongur^1^, Dr Ilakkiya Appavoo^1^, Dr Elisa Cerrone^1^, Dr Hamza Zafar^1^, Dr Abdul Hameed^1^, Professor David Kiely^1^, Professor Robin Condliffe^1^, Dr Judith Hurdman^1^, Professor Roger Thompson^1^, Professor Alex Rothman^1^

^1^Sheffield Teaching Hospitals, Sheffield, United Kingdom

Cardiopulmonary exercise testing (CPET) is an established tool to investigate the mechanisms of exercise intolerance in pulmonary hypertension (PH). By evaluating physiological responses during exercise, CPET can help differentiate PH‐related limitations from alternative causes. The Sheffield Pulmonary Vascular Disease Unit (SPVDU) offers a CPET service for diagnosis, follow‐up, and treatment guidance in patients with suspected or established PH. We conducted an audit of CPETs performed at SPVDU to assess service quality and clinical impact. Data were retrieved from CPET records and clinic letters and reviewed for test indications, and technical quality. We also evaluated which pathologies were identified, whether patients were discharged, whether CPET led to treatment changes, and the frequency of repeat testing. We analysed 323 CPETs. The most common indications were investigation of chronic thromboembolic disease (37%) and follow‐up of patients treated for pulmonary arterial hypertension (PAH) (31%). Tests were maximal in 67% of cases. Work rate selection was outside the target (duration between 8‐12 min) in 44%. Anaerobic threshold was achieved in 84% but was an estimate in 53%. Peak arterial blood gas was obtained in 66% of tests. In 46% of tests there was gas exchange abnormality, in 25% oxygen delivery impairment, in 21% deconditioning, in 12% ventilatory limitation, in 8% chronotropic incompetence, and in 6% dysfunctional breathing. Following CPET, 25% of patients were discharged, and 20% had treatment changes (escalation of PAH therapies, introduction of inhalers, or discontinuation of heart rate limiting medications). 17% of the patients had more than one CPET. There were no safety concerns (only one episode of asymptomatic ventricular tachycardia and no syncope). CPET is a safe test with a measurable impact on clinical decision‐making in PH patients, influencing both diagnosis and therapy. These findings highlight the importance of structured CPET services in optimising PH care.

## A149 The Cost of Delay: How a Treatable Tumor Led to ECMO in a 10‐Month‐Old

**Highlighting the Evidence Gap in Early Diagnosis and Support Systems for Vulnerable Pediatric Populations**

Dr. Kayla O'Sullivan^1,2^, Dr Delphine Yung^1,2^, Dr. Michael Lin^1,2^, NP Emma Jackson^2^, NP Meredith Riker^2^, Dr. Pamela Valentino^1,2^, Dr. Monique Radman^1,2^

^1^University of Washington School of Medicine, Seattle, United States, ^2^Seattle Children's Hospital, Seattle, United States

A 10‐month‐old Black girl presented with tachypnea and hypoxia. Radiographs showed cardiomegaly and echo identified supra‐systemic pulmonary hypertension (PH), large pericardial effusion, severe right ventricular (RV) dilation and dysfunction, and right‐to‐left PFO. Evaluation for PH uncovered multiple liver lesions via ultrasound and diffuse liver hemangiomas, worse on left lobe, on CTA. Infantile hepatic hemangiomas (IHH) were suspected. Propranolol and furosemide were initiated, and confirmatory MRI was scheduled. During anesthesia induction, the patient suffered cardiac arrest, requiring VA‐ECMO. She subsequently underwent left hepatic artery ligation and was decannulated two days later. After three days, RV size and function normalized, and PH resolved. Past history revealed normal echo at 6 months but liver lesions were identified, leading to hepatology referral which had not been scheduled. She had premature birth at 30 weeks, readmitted at 3 and 4 months, and five medical evaluations after 6 months for respiratory distress. Social history revealed housing insecurity, incarcerated father, maternal methamphetamine use, and four siblings.

IHH are benign endothelial tumors that proliferate rapidly until nine months and respond well to beta‐blockers. When diffuse and untreated, they can cause high output heart failure (HF), leading to PH. In infants with PH without lung disease or intracardiac shunt, systemic arterio‐venous shunts should be suspected. This patient's severe RV dysfunction and pericardial effusion indicated chronic high output HF. Anesthesia‐induced systemic vascular resistance drop precipitated cardiovascular collapse. Ligation of the hepatic artery is rarely indicated, but facilitated rapid reduction of the shunt. Earlier diagnosis and treatment might have prevented HF, PH, ECMO, and surgery. Social determinants of health, including race and poverty, contribute to delayed care and worse outcomes. Research is needed to identify effective, proactive suport systems for vulnerable families. Though potentially costly upfront, such interventions may reduce long‐term healthcare burdens and improve equity in outcomes.

## A150 Pulmonary Hypertension in Mexico: A Younger Phenotype With a Distinct Comorbidity Profile

Miss Alejandra Ortiz^1^, MD Nayeli Guadalupe Zayas Hernandez

^1^Autonomous University of Baja California, Mexicali, Mexico, ^2^National Institute of Cardiology “Ignacio Chávez”, Tlalpan, Mexico

This study provides a comprehensive analysis of a cohort of 359 patients with pulmonary hypertension (PH) from the National Institute of Cardiology “Ignacio Chávez” in Mexico. The research aimed to characterize the demographic, clinical, hemodynamic, and treatment profiles of this population, with a particular focus on the influence of comorbidities. The findings reveal a patient population that is significantly younger (mean age of 42.9 years) than those reported in international registries. A strong female predominance was observed (80.4%). The most common etiology was idiopathic pulmonary arterial hypertension (PAH). Over half the patients had at least one comorbidity, with hypothyroidism unexpectedly being the most prevalent (36.8%). Patients without comorbidities exhibited a more severe hemodynamic profile. Although monotherapy was the most used strategy, dual therapy was more frequent in patients with comorbidities, suggesting that complex clinical scenarios do not preclude the use of combination therapy. These findings highlight a distinct PH phenotype in the Mexican population, underscoring the need for national registries to develop tailored therapeutic guidelines that address the specific characteristics of this patient group.

## A151 Interstitial Lung Disease with Mean Pulmonary Artery Pressure >/=25 mmHg and Pulmonary Vascular Resistance > 3 WU: Interim Results From the PHINDER Study

David Zisman^2^, Steven Nathan^3^, Sandeep Sahay^4^, Meredith Broderick^1^, Dasom Lee^1^, Kevin Maher^1^, Dr Claire Thrasher^1^, Oksana Shlobin^3^

^1^United Therapeutics Corporation, Research Triangle Park, United States, ^2^Cleveland Clinic Florida, Weston, United States, ^3^Inova Fairfax Hospital, Falls Church, United States, ^4^Houston Methodist Hospital, Houston, United States

Despite the poor prognosis in interstitial lung disease (ILD) with pulmonary hypertension (PH), there are no evidence‐based guidelines to screen for PH in ILD. PHINDER (NCT05776225) aims to develop a PH screening tool in patients with ILD. The primary analysis will focus on patients with pre‐capillary PH, defined by the 2024 WSPH as mPAP> 20 mmHg, PVR > 2 WU, and PCWP < / = 15 mmHg. The interim analysis here describes findings in patients with ILD and PH as previously defined by the 2015 ESC/ERS Guidelines (herein called PH‐2015), with mPAP > /=25 mmHg, PVR > 3 WU, and PCWP < / = 15 mmHg. This definition was used in older studies of ILD‐PH, such as the INCREASE trial, which demonstrated safety and efficacy of inhaled treprostinil in this population. PHINDER is a prospective, multicenter study enrolling approximately 300 patients with ILD who undergo non‐invasive clinical assessments followed by right heart catheterization (RHC) to confirm the presence of PH. In this interim analysis, univariate logistic regression was performed on numerous variables from each assessment to determine their association with the presence of PH‐2015. Among 128 patients (mean age 71 years, 57% male, 82% white, 55% with IIP) 51% had PH‐2015 and 23% had normal hemodynamics (mPAP < /=20, PVR < / = 2, PCWP < / = 15) confirmed by RHC. The following were statistically significant for association with PH‐2015: supplemental oxygen use and flow rate, 6MWD, DLCO absolute and % predicted, FVC%/DLCO% ratio, RV pressure, tricuspid regurgitant velocity, RV/LV diameter ratio, pulmonary artery (PA)/aorta diameter ratio, and PA enlargement. These interim findings suggest that information from routine non‐invasive assessments, particularly supplemental oxygen requirements, 6MWD, PFTs, echo, and HRCT, may be useful in identifying patients with ILD and PH‐2015. Results from the fully enrolled PHINDER study will be used to develop a practical PH screening tool to aid in the detection of PH in patients with ILD.

## A152 Pulmonary Hypertension Screening in Interstitial Lung Disease: Interim Comparison of Clinician Suspicion vs Right Heart Catheterization in the PHINDER Study

Oksana Shlobin^1^, Maral DerSarkissian^2^, Matthew Hunsucker^3^, David Kiely^4^, Tejaswini Kulkarni^5^, Abhijit Raval^6^, Sandeep Sahay^7^, David Zisman^8^, Meredith Broderick^9^, Dasom Lee^9^, Kevin Maher^9^, Dr Claire Thrasher^9^, Mary Beth Scholand^10^

^1^Inova Fairfax Hospital, Falls Church, United States, ^2^Analysis Group, Los Angeles, United States, ^3^LeBauer Pulmonary Care, Greensboro, United States, ^4^University of Sheffield, Sheffield, England, ^5^University of Alabama at Birmingham, Birmingham, United States, ^6^AnMed Pulmonary & Sleep Medicine, Anderson, United States, ^7^Houston Methodist Hospital, Houston, United States, ^8^Cleveland Clinic Florida, Weston, United States, ^9^United Therapeutics Corporation, Research Triangle Park, United States, ^10^University of Utah, Salt Lake City, United States

Despite the poor prognosis of interstitial lung disease (ILD) with pulmonary hypertension (PH), there are no evidence‐based guidelines for PH screening in ILD. PHINDER (NCT05776225) aims to develop a PH screening methodology in patients with ILD. This interim analysis compared study clinicians’ suspicion of PH versus hemodynamically confirmed PH. PHINDER is a prospective, multicenter study where ILD patients undergo routine non‐invasive assessments followed by right heart catheterization (RHC) to confirm pre‐capillary PH (mPAP> 20 mmHg, PVR > 2 WU, PCWP < / = 15 mmHg). After reviewing all non‐invasive test results but prior to RHC, study clinicians are asked to rank their suspicion of PH as low, medium, or high. At the time of this interim analysis (May 2025), data were available for 189 patients, of whom 15% were deemed low suspicion for PH, 46% deemed medium suspicion, and 39% deemed high suspicion by their clinician prior to RHC. Parameters that trended along with and may have driven suspicion of PH, included 6‐minute walk distance, natriuretic peptide levels, supplemental oxygen requirements, peripheral edema or loud second heart sounds, FEV, FVC, DLCO, FVC/DLCO, RV pressure, tricuspid regurgitant velocity, pulmonary artery (PA) enlargement, PA/aorta diameter, and RV/LV diameter. Pre‐capillary PH was then confirmed by RHC in 32% of the low‐suspicion group (median mPAP 29 mmHg), 51% of the medium‐suspicion group (mPAP 26 mmHg), and 69% of the high‐suspicion group (mPAP 28 mmHg). A high level of discrepancy was observed between clinicians’ gestalt assessment of PH likelihood and RHC hemodynamics. Pre‐capillary PH was confirmed in almost a third of patients believed to have low likelihood for PH and was ruled out in almost a third of patients believed to have high likelihood for PH. These results underscore the need for more objective PH screening in patients with ILD.

## A153 Right‐Sided Hemodynamic Coupling in Pulmonary Hypertension (PH): RAP/PCWP Ratio Correlation With PAPi and RVSWI

Dr Athina Batsouli^1^, Doctor Panagiota ‐ Natalia Zimpounoumi ‐ Keratsa^1^, Doctor Athanasia Megarisiotou^1^, Doctor Sotirios Xydonas^1^, Doctor Efstathia Prapa^1^, Doctor Panagioula Niarchou^1^, Doctor Effrosyni Dima^2^, Professor Stylianos Orfanos^3^, Doctor Anastasia Anthi^2^

^1^Pulmonary Hypertension Center of “Evangelismos” Hospital, and Cardiology Department, Athens, Greece, ^2^Pulmonary Hypertension Center of “Evangelismos” Hospital and 1st Department of Critical Care Medicine and Pulmonary Services, Medical School, National and Kapodistrian University of Athens, “Evangelismos” Hospital, Athens, Greece, ^3^Critical Care, Medical School, National and Kapodistrian University of Athens, Athens, Greece

Right ventricular failure (RVF) is a major cause of morbidity and mortality in PH patients. The Right Atrial Pressure/Pulmonary Capillary Wedge Pressure (RA/PCWP) ratio correlates with right ventriculo‐arterial coupling and RVF, and has been thoroughly studied in heart failure patients. However, data on its hemodynamic impact in PH remains limited. We sought to investigate if RA/PCWP ratio could serve as a surrogate marker of right ventricular dysfunction in PH patients, by correlating it with Pulmonary Artery Pulsatility Index (PAPi) and Right Ventricular Stroke Work Index (RVSWI). 116 patients diagnosed in a referral center of PH over a period of 3 years, were enrolled. Right Heart Catheterization (RHC) under fluoroscopic guidance via internal jugular or femoral access using a Swan–Ganz catheter according to the standard protocol. RA/PCWP ratio, PAPi and Right Ventricle Stroke Work Index (RVSWI) were calculated for each patient. 116 consecutive patients were included in the study (62.9% females). The mean age of them was 66 ± 12,4 years and the mean calculated BSA was 1.87 ± 0.39m2. Mean RA/PCWP was 0.58 (SD = 0.24), mean PAPi was 7.47 ± 6.58 and mean RVSWI was 14.35 ± 6.07 g/m/beat/m². RA/PCWP was significantly and negatively correlated with PAPi (r = ‐.47; p < .001). RA/PCWP was significantly greater in patients with abnormal PAPi (M (SD) = 0.67(0.20) vs 0.52(0.24); p < 0.001). Furthermore, PAPi was significantly lower in patients with abnormal RA/PCWP (M (SD) = 5.09(2.66) vs 10.43(8.59); p < 0.001), while RVSWI did not differ significantly among patients with normal and abnormal RA/PCWP levels (p > 0.05). Moreover, the percentage of patients with abnormal PAPI levels was significantly greater in patients with abnormal RA/PCWP levels (65.1% vs 27.5%; p < 0.001). In this study, RA/PCWP was associated with lower values of PAPi, thus serving as a simple, integrative hemodynamic predictor of RVF in PH patients.

## A154 From Echocardiography to Right Heart Catheterization (RHC): RA Area, Pulmonary Artery Acceleration time and TAPSE/SPAP correlations with RVSWI, CPO, RA/PCWP and PVR/SVR ratio in Pulmonary Hypertension (PH)

Dr Athina Batsouli^1^, Doctor Panagiota‐Natalia Zimpounoumi‐Keratsa^1^, Doctor Sotirios Xydonas^1^, Doctor Athanasia Megarisiotou^1^, Doctor Panagioula Niarchou^1^, Doctor Effrosyni Dima^2^, Doctor Anastasia Anthi^2^, Professor Stylianos Orfanos^3^, Doctor Efstathia Prapa^1^, Doctor Athanasios Trikas^4^

^1^ Pulmonary Hypertension Center of “Evangelismos” Hospital, and Cardiology Department, Athens, Greece, ^2^Pulmonary Hypertension Center of “Evangelismos” Hospital and 1st Department of Critical Care Medicine and Pulmonary Services, Medical School, National and Kapodistrian University of Athens, “Evangelismos” Hospital, Athens, Greece, ^3^Critical Care, Medical School, National and Kapodistrian University of Athens, Athens, Greece, ^4^Cardiology Department, Athens, Greece

Right atrial area (RAarea), Pulmonary Artery Acceleration time (PAAT), and TAPSE/SPAP ratio are non‐invasive echocardiographic parameters indicative of right ventricular dysfunction. However, their correlation with invasive hemodynamic measurements such as RA/PCWP ratio, Pulmonary Vascular Resistance/Systemic Vascular Resistance ratio (PVR/SVR), Right Ventricle Stroke Work Index (RVSWI) and Cardiac Power Output (CPO) remains unclear. We sought to evaluate the correlations between RAarea, PAAT, TAPSE/SPAP and hemodynamic parameters (RVSWI, CPO, RA/PCWP, PVR/SVR ratio) in PH patients. We enrolled 71 consecutive PH patients diagnosed in our center.All patients underwent comprehensive echocardiographic assessment including RAarea,PAAT and TAPSE/SPAP calculation. RHC was conducted within one day, under fluoroscopic guidance. RA/PCWP ratio, RVSWI, CPO and PVR/SVR ratio were calculated for each patient. Data from 71 patients were analyzed (66% females). The mean age of patients was 62 ± 13 years and the mean calculated BSA 1.87 ± 0.39m2. Mean RA area was 19.75 ± 5.91 cm2, mean TAPSE/SPAP was 0.38 ± 0.15 mm/mmHg and mean PAAT was 73.65 ± 18.47 ms. RAarea values were significantly, positively correlated with RA/PCWP ratio(r = 0.32; p = 0.007).Also, lower TAPSE/SPAP values were significantly associated with greater RVSWI(r = ‐0.25; p = 0.038), CPO (r = ‐0.27; p = 0.026) and PVR/SVR(r = ‐0.48; p < 0.001) values. Furthermore, lower PAAT values were significantly associated with greater RVSWI (r = ‐0.37; p = 0.003) and CPO (r = ‐0.26; p = 0.039). RA area was significantly greater in patients with abnormal RA/PCWP (M(SD) = 20.91(6.64) vs 17.80(3.75); p = 0.024), while TAPSE/SPAP was significantly lower in patients with abnormal RVSWI(M(SD) = 0.36(0.14) vs 0.45(0.14); p = 0.019). In this study increased RA area was associated with higher RA/PCWP ratio. TAPSE/SPAP had a negative correlation with RVSWI, CPO and PVR/SVR ratio. PAAT had also a negative correlation with RVSWI and CPO. These echocardiographic parameters may serve as non‐invasive surrogates for invasive hemodynamic measurements, contributing to risk stratification and follow‐up of PH patients.

## A155 ECG‐Based Evaluation of Patients With Pulmonary Hypertension (PH): Can QTc Duration and P Wave Area Predict Invasively Measured Hemodynamic Parameters?

Dr Athina Batsouli^1^, Doctor Panagiota ‐ Natalia Zimpounoumi ‐ Keratsa^1^, Doctor Sotirios Xydonas^1^, Doctor Athanasia Megarisiotou^1^, Doctor Efstathia Prapa^1^, Doctor Panagioula Niarchou^1^, Doctor Effrosyni Dima^2^, Doctor Anastasia Anthi^2^, Professor Stylianos Orfanos^3^, Doctor Athanasios Trikas^1^

^1^Pulmonary Hypertension Center of “Evangelismos” Hospital, and Cardiology Department, Athens, Greece, ^2^Pulmonary Hypertension Center of “Evangelismos” Hospital and 1st Department of Critical Care Medicine and Pulmonary Services, Medical School, National and Kapodistrian University of Athens, “Evangelismos” Hospital, Athens, Greece, ^3^Critical Care, Medical School, National and Kapodistrian University of Athens, Athens, Greece, ^4^Cardiology Department, Athens, Greece

Electrocardiographic abnormalities are common in patients with pulmonary hypertension,reflecting right heart remodeling. This study investigates whether QTc duration and P wave area (PWA) could be used as predictors of invasively measured hemodynamic parameters. We sought to investigate the correlation between QTc duration, PWA and RA/PCWP ratio, Pulmonary Artery Pulsatility Index (PAPi), Right Ventricle Stroke Work Index (RVSWI) and Cardiac Power Output (CPO). We enrolled 72 consecutive PH patients. Right heart catheterization (RHC)was conducted according to the standard protocol.RA/PCWP ratio,RVSWI and CPO were calculated for each patient.Every patient underwent a12‐lead ECG at the same day of the RHC.The QT interval was measured manually. The corrected QT interval (QTc) interval was calculated using Bazett's and Fridericia's formula (QTc =QT/√RR and QTc= QT/RR1/3respectively).P wave area was calculated using the formula: P wave area = ½ P wave duration* P wave voltage. We used 4 ms*mV as a cut‐off for abnormal P Wave area values. Data from 72 patients were analyzed(66% females). Mean age was 62 ± 13years and mean calculated BSA was 1.87 ± 0.39m2. Mean QTc(Bazett) was 440.3 ± 37.64 ms, mean QTc (Fridericia)was 416.44 ± 26.7 ms and mean P wave area was 6.17 ± 2.53 ms*mV. Greater QTc (Fridericia) values were significantly associated with greater RVSWI (r = 0.33; p = 0.046) and greater CPO (r = 0.33; p = 0.046) values. Higher QTc (Bazett) values were significantly correlated with greaterCPO (r = 0.36; p = 0.031) values. PWA and QTc values (calculated with both Bazett and Fridericia formula) had a similar dispersion amongst patients, irrespectively of PAPi, RVSWI and RA/PCWP levels (p > 0.05). In our study increased QTc (Fridericia) values correlated with higher RVSWI and CPO. Increased QTc (Bazett) values correlated with higher CPO values. P wave area was not associated with RA/PCWP ratio, PAPi, RVSWI, or CPO. Additional data are required to clarify the association between these parameters and hemodynamic measurements.

## A156 Sotatercept in High‐Risk PAH Patients Under Triple Combination Therapy, in a Greek PAH centre

Dr Frantzeska Frantzeskaki^1^, MD Dimitrios Konstantonis^1^, MD Mihalis Rizos^1^, MD Maria Doumani^1^, Prof Iraklis Tsagkaris^1^

^1^Attikon University Hospital, Pulmonary Hypertension Clinic, Athens, Greece

Sotatercept, an activin signaling inhibitor, represents a novel therapeutic approach targeting pulmonary vascular remodeling in pulmonary arterial hypertension (PAH). Recent clinical trials have demonstrated significant improvements in hemodynamics, exercise capacity, and clinical outcomes when sotatercept is added to background therapy. However, real‐world evidence in high‐risk populations remains limited. To evaluate the short‐term clinical and biochemical effects of sotatercept in high‐risk PAH patients who remained symptomatic despite optimized triple combination therapy. In this prospective single‐centre study, four patients with high‐risk PAH received sotatercept in addition to established therapy. NT‐proBNP levels, 6‐minute walk distance (6MWD), and echocardiographic parameters—including tricuspid annular plane systolic excursion (TAPSE) and systolic pulmonary arterial pressure (SPAP)—were assessed at baseline and three months post‐initiation. At baseline, mean NT‐proBNP was 1447.2 pg/mL (range: 409–4226), decreasing markedly to 288.3 pg/mL (range: 131–797) after three months. Functional capacity improved, with mean 6MWD increasing from 389 m (range: 155–590) to 430 m (range: 228–505). Echocardiographic findings demonstrated enhancement in right ventricular performance (TAPSE: 21.5 mm to 23.3 mm) and a reduction in pulmonary pressures (SPAP: 80 mmHg to 72.5 mmHg). Hemoglobin and hematocrit levels remained stable throughout the study. One patient discontinued sotatercept due to severe hemoptysis. This real‐world experience supports the potential clinical benefit of sotatercept as an adjunctive therapy in high‐risk PAH patients unresponsive to maximal medical treatment. Improvements in hemodynamics, biomarkers, and exercise tolerance were observed within three months. Despite encouraging results, larger, long‐term studies are required to confirm the safety, durability, and optimal integration of sotatercept into advanced PAH treatment strategies.

## A157 PVR/SVR Ratio Strongly Correlates With PAPi and RVSWI in Pulmonary Hypertension Patients

Dr Panagiota‐natalia Zimpounoumi‐keratsa^1^, Doctor Athina Batsouli^1^, Doctor Athanasia Megarisiotou^1^, Doctor Sotirios Xydonas^1^, Doctor Efstathia Prapa^1^, Doctor Panagioula Niarchou^1^, Doctor Effrosyni Dima^2^, Professor Stylianos Orfanos^3^, Doctor Anastasia Anthi^2^

^1^Pulmonary Hypertension Center of “Evangelismos” Hospital, and Cardiology Department, Athens, Greece, ^2^Pulmonary Hypertension Center of “Evangelismos” Hospital and 1st Department of Critical Care Medicine and Pulmonary Services, Medical School, National and Kapodistrian University of Athens, “Evangelismos” Hospital, Athens, Greece, ^3^Critical Care, Medical School, National and Kapodistrian University of Athens, Athens, Greece, Athens, Greece

Pathophysiologically, PVR/SVR reflects the interplay between pulmonary and systemic vascular resistance, serving as a simultaneous marker of Right Ventricular–Pulmonary Artery (RV–PA) coupling and Right Ventricle pressure load. Therefore, PVR/SVR ratio's correlation with well‐established hemodynamic parameters, may yield insights into right ventricular failure (RVF) in patients with Pulmonary Hypertension (PH). We sought to investigate,PVR/SVR ratio association with Pulmonary Artery Pulsatility Index (PAPi) and Right Ventricular Stroke Work Index (RVSWI). We enrolled 116 patients diagnosed with PH in a referral center, over a period of 3 years.RHC was conducted under fluoroscopic guidanceaccording to the standard protocol,via internal jugular or femoral vein, using a Swan‐ Ganz catheter. All pressure measurements were performed at end‐expiration, and cardiac output (CO) was calculated with direct Fick method.PVR/SVR ratio, PAPi [Pulmonary Artery Systolic Pressure (PASP) – Pulmonary Artery Diastolic Pressure (PADP)/Right Atrial Pressure (RAP)] and Right Ventricle Stroke Work Index (RVSWI) [0.0136 × Stroke Volume Index × (Mean Pulmonary Artery Pressure (MPAP) ‐ Mean RAP) were calculated for each patient. 116 consecutive patients (62.9% females) were included in this study. Mean age was 66 ± 12.4 years and mean calculated BSA 1.87 ± 0.39m2. Mean PVR/SVR ratio was 0.26 (SD = 0.15), mean PAPi was 7.47 (SD = 6.58), and mean RVSWI was 14.35 (SD = 6.07) g/m/beat/m². PVR/SVR ratio was significantly and positively associated with RVSWI(r = .59; p < .001). It was also significantly lower in patients with abnormal PAPi (M(SD) = 0.23(0.13) vs 0.30(0.16); p = 0.009) and significantly greater in patients with abnormal RVSWI(M(SD) = 0.29(0.16) vs 0.19(0.09); p = 0.001). In this study, PVR/SVR ratio had a strong correlation with PAPi and RVSWI. These results underly the value of PVR/SVR ratio in hemodynamic assessment, risk stratification, and therapeutic management of PH patients.

## A158 Echocardiographic RVOT‐VTI Indices: A Game Changer for Diagnosis of Pulmonary Hypertension in Patients with Intermediate Echocardiographic Probability?

Dr Panagiota‐natalia Zimpounoumi‐keratsa^1^, Cardiologist Athina Batsouli^1^, Doctor Sotirios Xydonas^1^, Doctor Athanasia Megarisiotou^1^, Doctor Panagioula Niarchou^1^, Doctor Efrosini Dima^2^, Doctor Anastasia Anthi^2^, Professor Stylianos Orfanos^3^, Doctor Efstathia Prapa^1^, Doctor Athanasios Trikas^1^

^1^Pulmonary Hypertension Center of “Evangelismos” Hospital, and Cardiology Department, Athens, Greece, ^2^Pulmonary Hypertension Center of “Evangelismos” Hospital and 1st Department of Critical Care Medicine and Pulmonary Services, Medical School, National and Kapodistrian University of Athens, “Evangelismos” Hospital, Athens, Greece, ^3^Critical Care, Medical School, National and Kapodistrian University of Athens, Athens, Greece, ^4^Cardiology Department, Athens, Greece

Right Ventricular Outflow Track Velocity Time Integral (RVOT‐VTI) is a well‐studied echocardiographic marker, associated with invasively measured hemodynamic impairment in patients with established or severe Pulmonary Hypertension (PH). However, data on patients with intermediate echocardiographic probability of PH are lacking. We sought to investigate, the correlation between RVOT‐VTI, RVOT‐VTI indexed for BSA (RVOT‐VTIi) and a novel parameter introduced by our team, the product of RVOT‐ VTIi and TAPSE (RVOT‐VTIi x TAPSE) with mean Pulmonary Arterial Pressure (meanPAP) and Pulmonary Vascular Resistance (PVR). We enrolled 66 consecutive patients with intermediate echocardiographic probability of PH. RVOT‐VTI, RVOT‐VTIi and a novel parameter, VTIi×TAPSE, were calculatedby Pulse‐Wave Doppler Echocardiography.Within a day, the patients underwent Right Heart Catheterization (RHC) under fluoroscopic guidance according to the standard protocol. The correlation of calculated parameters with invasively measured mean PAP and PVR was studied. Statistical analysis was conducted using SPSS statistical software (version 27.0). Data from 66 patients (63.6% females, mean age 61 ± 11,9 years) were analyzed. Abnormal RVOT‐VTI values were observed in 18 patients (27.3%), abnormal PVR in 54 patients (81.8%) and elevated mean PAP in 56 patients (84.8%). The mean values of the measured parameters were: mean PVR 3.2 ± 1.52 Wood Units, mean PAP 28 ± 8.9 mmHg, mean RVOT‐VTI 16.9 ± 3.8 cm, mean RVOT‐VTIi 9.60 ± 2.55 and mean VTIi xTAPSE 193.1 ± 61.2.PVR and mean PAP were not significantly associated with RVOT‐VTI, RVOT ‐VTIi and VTIixTAPSE (r ranged from ‐.20 to ‐.10; p > .05). Although promising, a statistically important association between RVOT‐VTI indices and RHC parameters was not demonstrated in our study, thus implying that they are more sensitive echocardiographic markers once RV–pulmonary arterial (RV–PA) coupling deteriorates, typically in advanced PH. There is a need for larger studies focusing on RVOT‐VTI parameters in patients with intermediate echocardiographic probability for PH.

## A159 A Phase 1 Trial to Assess the Safety, Tolerability, and Pharmacokinetics of APL‐9796 in Healthy Adults

Mr Matthew Cardinal^1^, Rachel Gurrell^1^, Dr. Sanjay Aggwaral^1^, Stephanie Hopley^1^, Behnaz Ravanfar^1^, Kathryn Brown^2^, Patrick Eastham^2^

^1^Apollo Therapeutics Ltd, Cambridge, United Kingdom, ^2^Certara, Inc., Randor, United States

The zinc‐regulated transporter‐like, iron‐regulated transporter‐like protein 12 (ZIP12) is aberrantly expressed in patients with pulmonary hypertension (PH). APL‐9796 is a human ZIP12 monoclonal antibody inhibitor for subcutaneous (SC) administration being developed for the treatment of PH. AP13CP01 was a randomised, double‐blind, placebo‐controlled trial of APL‐9796 across multiple single ascending dose (SAD) cohorts (dose levels: 0.3, 1, 3, 6, 12 mg/kg). Healthy participants were followed up to Day 160 post‐dosing to monitor for treatment‐emergent adverse events (TEAEs), and to collect samples for pharmacokinetic (PK) and immunogenicity (anti‐drug antibody [ADA]) assessments. APL‐9796 was well tolerated, with no dose‐related trends in safety and tolerability. Overall, there was a total of 31 TEAEs; a similar proportion were reported for participants in the pooled APL‐9796 group (14 of 29 [48.3%]) and the placebo group (5 of 10 [50.0%]). There were no serious TEAEs, TEAEs leading to death, TEAEs leading to trial withdrawal, or TEAEs leading to drug discontinuation. There were no abnormalities in laboratory parameters that were deemed clinically significant; abnormalities were transient and did not appear to have any trend with increasing APL‐9796 dose. Incidence of ADA was relatively low (6.9%). Dose‐proportional PK was observed, with a mean t½ of 53 days. A population PK (PopPK) model was developed, to enable PK simulations to support selection of dosing regimens in the ongoing AP13CP02 Phase 2 multiple ascending dose (MAD) trial in Group 1 and Group 3 PH (NCT06846554). PopPK model simulations demonstrated that ≥ 1.0 mg/kg Q4W APL‐9796 is projected to achieve efficacious trough concentrations over a 6‐month treatment duration, based on EC50 estimates from nonclinical models. The safety and PK data from this Phase 1 trial supported the progression to the Phase 2 trial evaluating the safety and efficacy of APL‐9796 in patients with PH undergoing continuous haemodynamic monitoring via telemetry with CardioMEMS®.

## A160 Pericytes Regulate Vascular Pruning and Inflammation in Smoke‐Induced Emphysema Through a Cox4i2‐Dependent Mechanism: Therapeutic Potential of Mitochondrial ROS Inhibition

Miss Claudia Garcia^1^, Vidya Lakshmi^1^, PhD Stefan Hadzic^1^, PhD Claudio Nardiello^1^, David Glaser^1,2^, Professor Maik Hüttemann^3^, Professor Lawrence I Grossman^3^, Dr. Baktybek Kojonazarov^1,2^, Muchen Li^1^, Sandipan Jash^1^, PhD Janine Koepke^1^, PhD Marija Gredic^1^, PhD Cheng‐Yu Wu^1^, PhD Luca Giordano^1^, Professor Matthias Hecker^1^, Professor Christos Samakovlis^1,4,7^, Edma Loku^1^, Anis Cilic^1^, Julian Better^5^, Dr. Ulrich Matt^5^, PhD Learta Pervizaj‐Oruqaj^5^, Dr. Jochen Wilhelm^1,2^, Professor Susanne Herold^5^, PhD Slaven Crnkovic^1,2,6^, Professor Grazyna Kwapiszewska^1,2,6^, Professor Friedrich Grimminger^1^, Professor Marek Bartkuhn^1,2^, Professor Werner Seeger^1,2^, Professor Norbert Weissmann^1^, PhD Oleg Pak^1^, Professor Natascha Sommer^1^

^1^Cardiopulmonary Institute (CPI), Universities of Giessen and Marburg Lung Center (UGMLC), Member of the German Center for Lung Research (DZL), Justus‐Liebig University, Giessen, Germany, ^2^Institute for Lung Health (ILH), Justus Liebig University, Giessen, Germany, ^3^Center for Molecular Medicine and Genetics, Wayne State University School of Medicine, Detroit, USA, ^4^Department of Molecular Biosciences, Wenner‐Gren Institute, Stockholm University, S10691, Stockholm, Sweden, ^5^Department of Medicine V, Internal Medicine, Infectious Diseases and Infection Control, Universities of Giessen and Marburg Lung Center (UGMLC), German Center for Lung Research (DZL), Justus‐ Liebig University, Giessen, Germany, ^6^Otto Loewi Research Center, Lung Research Cluster, Medical University Graz, Graz, Austria, ^7^Science for Life Laboratory, Tomtebodavägen 232, 171 21, Stockholm, Sweden

Chronic obstructive pulmonary disease (COPD) is characterized by persistent inflammation and progressive lung remodelling, leading to emphysema and pulmonary hypertension (PH). Mitochondrial reactive oxygen species (mtROS) contribute to vascular inflammation and endothelial dysfunction in COPD. We previously identified cytochrome c oxidase subunit 4 isoform 2 (Cox4i2) as a key modulator of physiological mtROS release. Here, we investigated the role of Cox4i2 in cigarette smoke (SE)–induced emphysema and PH, and the therapeutic potential of mtROS neutralization by MitoQ. C57BL/6 J (WT) and Cox4i2‐/‐ mice were exposed to SE or room air for 3 or 8 months. PH and right ventricular function were assessed by echocardiography and hemodynamics, and lung mechanics by the forced oscillation technique. Inflammation was quantified by BAL cytospin and FACS, and apoptosis by FMT–µCT imaging. Transcriptomic alterations were analyzed by microarray and scRNA‐seq. Vascular remodelling was evaluated by microvascular casting and histology. Cox4i2‐/‐ mice developed significantly less emphysema but a similar degree of PH compared to SE exposed WT, associated by reduced apoptosis, inflammation, and nitrotyrosine formation. scRNA‐seq and tomato‐Cox4i2 reporter mice analyses revealed Cox4i2 expression in two cell subtypes: either in precapillary (Acta2High) or capillary (Acta2Low “classical pericytes”). Both cell subtypes showed reduced cigarette smoke extract (CSE) or SE‐induced mtROS release in vitro and in vivo respectively. Cox4i2‐/‐ Acta2High improved AEC2 viability while Acta2Low pericytes induced less neutrophil migration. Cox4i2‐/‐ also mitigated CSE‐induced inhibition of endothelial tube formation. Microvascular casting demonstrated reduced vascular volume in WT lungs after SE, which was attenuated in Cox4i2‐/‐ mice, albeit decreased vascular volume at baseline. MitoQ treatment 3 months post SE reduced inflammation and reversed emphysema and PH. Cox4i2 in pericytes regulates smoke‐induced inflammation and pulmonary vascular stability but not PH. In contrast, mtROS neutralization by MitoQ reversed both emphysema and PH, highlighting its therapeutic potential in COPD.

## A161 Histopathologic and Hemodynamic Evaluation of Macitentan 30 mg/kg and 3 mg/kg Doses in a Sugen/Hypoxia‐Induced Rat Model of Severe Pulmonary Arterial Hypertension

Dr Michael Hansen^1^, Lillian Sanna^2^, Anthony Davenport^3^, Fuyong Du^1^, David Langleben^4^, David Webb^5^, Sebastien Bonnet^6^

^1^Johnson & Johnson, Spring House, USA, ^2^Johnson & Johnson, Allschwil, Switzerland, ^3^Experimental Medicine and Immunotherapeutics, University of Cambridge, Cambridge, UK, ^4^Center for Pulmonary Vascular Disease, Division of Cardiology, Jewish General Hospital, McGill University, Montreal, Canada, ^5^Centre for Cardiovascular Science, University of Edinburgh, Edinburgh, UK, ^6^Pulmonary Hypertension Research Group, Centre de Recherche de l'Institut de Cardiologie et de Pneumologie de Québec, Quebec City, Canada

In the pivotal SERAPHIN study of macitentan 3 mg and 10 mg in human pulmonary arterial hypertension (PAH), a dose‐dependent effect on morbidity/mortality was observed. In a monocrotaline‐induced rat model of PAH, macitentan showed dose‐dependent improvements in hemodynamics and right ventricular (RV) hypertrophy. This study assessed histopathologic and hemodynamic effects of daily macitentan 30 mg/kg and 3 mg/kg in a Sugen/hypoxia rat reversal model of severe PAH. On Day 1, male Sprague Dawley rats were injected with semaxanib 60 mg/kg and exposed to hypoxia (~13–15% O2) until Day 22, then moved to normoxia (PAH rats). Control rats were maintained in normoxia. From Day 36–64, PAH rats received once‐daily macitentan (3 or 30 mg/kg) or vehicle (PAH‐vehicle). Assessments were conducted on Day 64. Comparison with control animals (n = 6) confirmed disease induction in PAH rats (n = 16/group). Lung histopathology showed macitentan 30 mg/kg reduced PAH disease severity versus PAH‐vehicle (mean sum lesion scores: 2.6 versus 6.1; P < 0.01), driven by decreased pulmonary artery hypertrophy and perivascular/interstitial inflammation; the mean proportion of fully muscularized arterioles was also reduced (78.6% versus 92.4%, P < 0.05). Macitentan 30 mg/kg reduced mean pulmonary arterial pressure (38 mmHg versus 59 mmHg, P < 0.01), RV systolic pressure (62 mmHg versus 109 mmHg, P < 0.05) and RV hypertrophy (Fulton index: 0.41 versus 0.58, P < 0.01) versus PAH‐vehicle. No statistically significant differences between macitentan 3 mg/kg and PAH‐vehicle were observed for the above parameters.

Macitentan 30 mg/kg, but not 3 mg/kg, significantly reduced pulmonary vascular and cardiac remodeling and improved hemodynamics in a rat model of severe PAH. The translational relevance of these findings is being explored in the ongoing UNISUS study (NCT04273945), a Phase 3 head‐to‐head superiority study of macitentan 75 mg versus macitentan 10 mg in patients with PAH.

## A162 Anti‐fibrotic Treatment of Pulmonary Venous Obstructive Disease

Julia Gaal^1^, Peter Hammer, PhD^1^, Gwang‐Bum Im, PhD^1^, Yunhye Kim, PhD^1^, Kerstin Saraci^1^, Cindy Zajac^1^, Tim Klouda, DO^1^, Daniel Diaz‐Gil, MD^1^, Christopher Baird, MD^1^, Kathy Jenkins, MD, MPH^1^, Juan Melero‐Martin, PhD^1^, Pedro J. del Nido, MD^1^, Ke Yuan, PhD^1^, Dr Ingeborg Friehs^1^

^1^Boston Children's Hospital, Harvard Medical School, Boston, United States

Pulmonary vein stenosis (PVS), typically arising at the veno‐atrial junction, which is lined by endocardium, has been reported to be associated with elevated shear stress. We have previously shown that endocardial endothelial cells (EECs) show a distinct susceptibility to fibrogenic activation through endothelial‐to‐mesenchymal transition (EndMT). Sodium‐glucose cotransporter 2 (SGLT2) inhibitors such as dapagliflozin have been shown to attenuate cardiac fibrosis. In this study, we aimed to investigate the role of pathological shear stress (WSS) in driving fibrogenic activation of EECs in PVS and to assess the therapeutic potential of dapagliflozin. Tissue specimens (n = 11) were obtained from pediatric patients with PVS during routine cardiac surgery, and fibrogenic EECs were isolated for culture. WSS was calculated from preoperative CT images. Tissue composition was analyzed using light microscopy, and EndMT by immunohistochemistry. EECs were exposed to pathological (40 dyn/cm²) or physiological WSS (7.5 dyn/cm²), ±0.5 µM dapagliflozin, for 48hrs. EndMT was evaluated for double‐positive NPR3⁺/vimentin⁺ (endocardial/mesenchymal marker) EECs and mean vimentin expression per 100 NPR3⁺ EECs. Data were analyzed using unpaired t‐tests and expressed as median (range) or mean ± SD. All patients presented with bilateral, multivessel disease, with median gradient of 18 mmHg (9–32) and pathological shear stress of 120 dyn/cm² (42–155). Resected tissue demonstrated fibroelastic remodeling with double‐positive EECs. Pathological shear stress significantly increased double‐positive EECs (17.46 ± 12.67% vs. 75.40 ± 26.01%, p < 0.0001) and mean mesenchymal expression (14.77 ± 7.32 vs. 35.75 ± 23.02, p < 0.01) compared to physiological shear stress. Dapagliflozin markedly reduced the percentage of double‐positive cells (75.40 ± 26.01% vs. 11.00 ± 5.93%, p < 0.0001) and mean vimentin expression (35.75 ± 23.02 vs. 11.34 ± 4.49, p < 0.001) under pathological shear stress. We demonstrate, for the first time, that pathological WSS drives EndMT of EECs, localized at the areas of PVS. Moreover, we show that dapagliflozin effectively suppresses EndMT under disease conditions. Targeting EEC‐driven fibroelastic remodeling may represent a promising therapeutic strategy for patients with PVS.

## A163 Proteomics Analysis Identifies Sub‐Phenotypes Amongst Clinically Similar Patients With Idiopathic Pulmonary Arterial Hypertension

Dr Megan Griffiths^1^, Dr Peifeng Ruan^1^, Dr Cedric Manlhiot^2^, Dr Eric Austin^3^, Dr William Nichols^4^, Dr Allen Everett^5^,

^1^University Of Texas Southwestern, Dallas, United States, ^2^The Hospital for Sick Children, University of Toronto, Toronto, Canada, ^3^Vanderbilt University Medical Center, Nasheville, United States, ^4^Cincinnati Children's Hospital Medical Center, Cincinnati, United States, ^5^Johns Hopkins University, Baltimoe, United States

Patients with idiopathic pulmonary arterial hypertension (IPAH) may be clinically similar, yet have different responses to therapy and outcomes. Proteomic profiles may identify molecular sub‐phenotypes amongst otherwise clinically similar patients with IPAH and identify novel pathways that may differentiate patients. A cross‐sectional, prevalent cohort of patients with IPAH (N = 120, 60 survivors/60 non‐survivors) was selected from the PAH Biobank with similar clinical, genetic hemodynamic and risk profiles. Plasma levels of 11,000 proteins (SomaScan 11 K Platform) were assayed. Spectral clustering was done using the top 10% most variable proteins. Survival by cluster was evaluated by Kaplan‐Meier analysis and Cox proportional hazards model, adjusted for enrollment age and sex. Pathway analysis identified major proteomic pathways differentiating each cluster. Spectral clustering identified 3 clusters, with 36%, 45% and 68% events over 5‐years in clusters 1, 2 and 3 respectively (p = 0.0025). The adjusted hazard ratio for death was 1.40 and 2.78 in clusters 2, 3 comparing to cluster 1. Cluster 3 subjects were older, but medications, six‐minute walk test and hemodynamics were similar across clusters. Most subjects were low or intermediate risk (ERS and REVEAL 2.0 score). Vascular Endothelial Growth Factor (VEGF) signaling (p = 9.30E‐04) was enriched between clusters 1 and 2, Wnt signaling was enriched between clusters 1 and 3 (p = 0.0014), and Neurotrophin signaling (p = 4.82E‐09) and VEGF signaling (p = 5.93E‐09) enriched between clusters 2 and 3. This study identified differential expression of 3 pathways across clusters of similar IPAH patients with differences in outcomes not predicted by clinical risk assessment. All three of these pathways, Wnt, VEGF and Neurotrophin, have been implicated in pulmonary vascular and right ventricular dysfunction and may identify patients at higher risk or at different stages of the IPAH disease process. Understanding the proteome may identify molecular phenotypes of IPAH associated with outcomes but missed by clinical classification.

## A164 Expression of The Human Thromboxane Receptor in Pulmonary Hypertension and Related Cardiopulmonary Conditions

Eamon P. Mulvaney^1^, Margaret E. Eng^1^, Helen M. Reid^1^, Frédéric Perros^2^, Pedro Mendes‐Ferreira^3^, Prof Therese Kinsella^1^

^1^ATXA Therapeutics Limited, UCD Conway Institute of Biomolecular and Biomedical Research, University College Dublin, Ireland, ^2^CarMeN Laboratory, INSERM U1060, INRAE U1397, Université Claude Bernard Lyon 1, France, ^3^RISE‐Health, Department of Surgery and Physiology, Faculty of Medicine of the University of Porto, Portugal

NTP42, a novel thromboxane (TX)A₂ receptor (TP) antagonist, is in clinical development for pulmonary arterial hypertension (PAH), and other pulmonary hypertension (PH) and cardiopulmonary diseases. Phase I studies in 92 healthy volunteers showed that NTP42 is safe, well‐tolerated, displaying favourable pharmacokinetic and pharmacodynamic properties, and direct evidence of TP target engagement. In preclinical models, NTP42 improved pulmonary haemodynamics and vascular remodelling. It also improved cardiac function and promoted beneficial right ventricular (RV) adaptation. Uniquely, these findings suggest both pulmonary‐specific and direct cardioprotective benefits for NTP42, highlighting its potential as a multi‐modal therapy in cardiopulmonary diseases. Thus, to explore this therapeutic potential of NTP42, TP expression was examined in lung and RV tissue from patients with distinct cardiopulmonary diseases. In PAH lungs, it was expressed in multiple cell types, including endothelial and smooth muscle (SM) cells of the pulmonary arterioles and within plexiform lesions. Expression was also evident in idiopathic pulmonary fibrosis (iPF) and Group 3 PH lungs, particularly in vascular SM, bronchiolar epithelium, and in aberrant epithelioid and inflammatory cells. Relative to control tissue, TP expression was increased in RV tissue in the PAH heart, with similar findings in RV tissues from dilated cardiomyopathy, a driver of Group 2 PH. Notably, TP expression was also increased in lungs and RV tissue from preclinical models of PAH, including the monocrotaline, Sugen‐hypoxia and BMPR null models, as well as pulmonary artery banding and ZSF‐1 heart failure models. Supporting these findings, interrogation of publicly‐available single‐cell RNA sequencing datasets confirmed elevated TP gene expression in key cardiac and pulmonary tissue in PAH and other cardiopulmonary conditions, including heart failure and iPF. In summary, these findings strengthen evidence for TP involvement in diverse cardiopulmonary diseases and highlight the potential of NTP42 as a promising therapeutic strategy for both the underlying pulmonary and cardiac dysfunctions.

## A165 Open‐label, Multicenter Study to Evaluate the Safety and Efficacy of Liposomal Treprostinil Inhalation Suspension (L606) in Subjects with Pulmonary Arterial Hypertension (PAH) and Pulmonary Hypertension Associated with Interstitial Lung Disease (PH‐ILD)

Dr Akshay Muralidhar, Dr Maria Triveri, Dr Ricardo Restrepo‐Jaramillo, Dr Jean Elwing, Dr Charles Burger, Dr Peter Yau, Ms Anna Leblond, Dr Sanjeev Khindri, Dr Rajeev Saggar

L606 is a novel investigational inhaled sustained‐release formulation of liposomal treprostinil designed to prolong lung retention, potentially improving efficacy and tolerability. L606 is administered twice daily by rapid nebulization with a compact hand‐held device. Open‐label study in 28 patients with PAH or PH‐ILD who transitioned from inhaled Tyvaso to L606 or were naïve to prior prostacyclin therapy. The objectives of the trial are to evaluate the safety and exploratory efficacy of L606 up to 48 weeks. 23 patients were in the transition group (18 PAH: 5 PH‐ILD), with 5 in the naïve group (PAH). 24 (85.7%) patients completed 48 weeks’ treatment. Three discontinued due to TEAE. The majority patients were female (75%), white (78.6%) with a mean age of 61 years. At baseline, 55.6% were NYHA FC II; 44.4% FC III with a median 6MWD of 395 m. In the transition group, 17/18 (94%) were on triple combination treatment. In the naïve group, 2/5 (40%) were on dual treatment. L606 mean dose at 48 weeks was 238.8 mcg BID, a daily dose comparable to at least 17 breaths QID of inhaled Tyvaso. Overall, 25 (89.3%) patients experienced at least 1 TEAE. Ten (35.7%) experienced treatment‐related TEAE. There was no related SAE. The most common related TEAE was cough (all mild) in 14.3% of patients. Following 48‐weeks of treatment with L606, there was a mean (SD) change from baseline of +29.4 m (64.3) with median change from baseline of +22.5 m in the 6MWD. 37.5% patients improved 6MWD by 30 m or more. Inhaled L606 was safe and well tolerated in both patients transitioning from inhaled Tyvaso or naïve to prior prostacyclin therapy over 48 weeks. Most TEAEs were mild. Cough (14.3%) was the most common drug‐related TEAE. There was a mean improvement of 29.4 m in 6MWD.

## A166 A Novel Digital Support Program for Home Based Prostacyclin Dose Increases

Ms Caitriona Minnock^1^, Ms Denise Lennon^1^, Dr Sarah Cullivan^1^, Dr Brian McCullagh^1^

^1^National Pulmonary Hypertension Unit, MMUH, Dublin 7, Ireland

Sub‐cutaneous (SC) treprostinil, a prostacyclin analogue, is used in the treatment of pulmonary arterial hypertension (PAH). While the dose of treprostinil is incrementally increased after initiation, the severity of a patient's condition can make visits to the PH centre for assessment and dose increases difficult. As a result, patients are admitted for dose increases in some PH centres. This may be disruptive to home life and an inefficient use of hospital resources. We sought to determine if an Advanced Nurse Practitioner (ANP) delivered Virtual Clinic (VC) with digital support could safely increase doses at home and achieve significant bed day savings for the Hospital. Digital for Care 2030 is a strategic plan to modernise and transform healthcare in Ireland. A “seedling” funding allocation from this strategy, supported this PH ANP delivered pilot phase. TPRO© eClinic Manager Platform, with Patient m Power© Bluetooth‐connected blood pressure, pulse oximetry and scales was used to support the VC program. Patients were followed (n = 7) over a period of 8 weeks. Two visits, VC and a telephone visit (TV), were conducted per dose increase to monitor effect. Dose titration was successfully achieved at home with close monitoring, medication counselling, effective patient education and tolerability assessment. The mean dose increase achieved was 10.18 (+/‐ 0.101) nanograms/kg/min over a mean of 40 (+/‐ 6) days. Patient satisfaction was positively expressed. No significant side effects were observed. Mean number of VC/TV conducted was 12.4 (+/‐2.6) per patient. No hospital admissions were required resulting in 50 bed days saved. Clinical data was uploaded accurately by patients. This program has been evaluated to ensure feasibility, safety and reduce hospital admissions whilst achieving target treprostinil doses. An unanticipated improvement in SC treprostinil cannula site pain and lifespan of cannula was reported by patients during the support program.

## A167 PHocus: A Global Phase 2 Study of Inhaled Mosliciguat in Adults With Interstitial Lung Disease‐Associated Pulmonary Hypertension (ILD‐PH)

Hossein Ardeschir Ghofrani^1^, Vincent Cottin^2^, Laura Price^3^, Rajan Saggar^4^, Rogerio de Souza^5^, Aaron Waxman^6^, Jennifer Cormier^7^, Carlos Sanmarco^7^, Dhiya Sani^7^, Dr Ubaldo Martin^7^, Marc Humbert^8^

^1^Department of Internal Medicine, Justus‐Liebig University Giessen and Marburg Lung Center (UGMLC), Giessen, Germany, ^2^National Reference Centre for Rare Pulmonary Diseases, University of Lyon, Lyon, France, ^3^Royal Brompton and Harefield Hospitals, London, United Kingdom, ^4^Division of Pulmonology, University of Southern California Health System, Los Angeles, USA, ^5^Pulmonary Medicine and Pulmonary Circulation Program, University of Sao Paulo, Sao Paulo, Brazil, ^6^Pulmonary Vascular Disease Program, Brigham and Women's Hospital, Boston, USA, ^7^Pulmovant, Inc., Waltham, USA, ^8^Pulmonary Respiratory Medicine, Université Paris‐Saclay, Paris, France

Evaluate safety and efficacy of once‐daily (QD) inhaled mosliciguat via dry‐powder inhaler (DPI) in ILD‐PH. Inhaled mosliciguat, an investigational soluble guanylate cyclase (sGC) activator, works independently of heme and nitric oxide and has been extensively characterized in a Phase 1 program including adult participants with PH. The Phase 2 PHocus study is evaluating mosliciguat as a potential treatment for ILD‐PH, a condition marked by high mortality and in need of therapeutic options. PHocus (NCT06635850) is a two‐part, randomized, double‐blind, placebo‐controlled, Phase 2 study in approximately 120 adult participants with ILD‐PH and pulmonary vascular resistance (PVR) ≥ 4 Wood units assessed by right heart catheterization (RHC). Part 1 is the blinded placebo‐controlled Treatment Period (24 weeks). Participants will be randomized 2:1 to QD inhaled mosliciguat or matched placebo and individually titrated to a maximally tolerated dose. The primary endpoint is change from baseline at Week 16 in PVR measured by RHC. Secondary endpoints include change from baseline at Week 16 in 6‐minute walk distance and N‐terminal pro‐brain natriuretic peptide. Pharmacokinetics of mosliciguat will also be assessed. Safety and tolerability of inhaled mosliciguat will be evaluated by the incidence and nature of treatment‐emergent adverse events (AEs), serious AEs, and AEs leading to study discontinuation. Functional improvement and time to clinical worsening will be assessed at Week 24. After Week 24, participants will have the opportunity to receive inhaled mosliciguat through an open‐label Extension Period (Part 2). Inhaled mosliciguat, a QD, sGC activator with targeted delivery to the lungs, has been extensively characterized in a Phase 1 program. The Phase 2 PHocus study, currently enrolling participants with ILD‐PH, is designed to generate clinical data to inform the therapeutic potential of inhaled mosliciguat via DPI in a population with high morbidity and mortality for whom therapeutic options are limited.

## A168 Association Between Arteriovenous Fistula and the Likelihood of Group 5 Pulmonary Hypertension in Patients with Chronic Kidney Disease

Dr Juan Carlos Hernandez Herrera^1^, Dra Leydi Sanchez Meneses^1^, Dr Nicolas Pacheco Ramirez^1^, Dr Julio Cesar Lopez Reyes^1^, Dra Nayeli Guadalupe Zayas Hernandez^1^, Dr Rodrigo Zebadua Torres^1^, Dr Mario Alejandro Diaz Pastrana^1^, Dra Arantxa Nava Suarez^1^

^1^Instituto Nacional De Cardiología. Ignacio Chávez, Tlalpan, México

Pulmonary hypertension (PH) is one of the complications observed in patients with end‐stage chronic kidney disease (CKD) and arteriovenous fistula (AVF). In these patients, an increase in blood flow through the fistula greater than 1.5 liters, or a fistula flow‐to‐cardiac output ratio greater than 20%, predisposes them to a high risk of developing PH. The objective was to determine the probability of group 5 PH in patients with CKD undergoing renal replacement therapy associated with AVF by means of transthoracic echocardiography. A descriptive and retrospective study was conducted, including 115 patients with CKD undergoing hemodialysis whose AVF was created between 2014 and 2025. The following variables were recorded: age, sex, etiology, date of initiation of hemodialysis, anatomical site of the fistula. To determine the probability of PH, echocardiographic data were used: tricuspid regurgitation velocity and parameters such as right ventricular diameter, TAPSE/PSAP ratio, and right atrial area. It was determined that in this population, 60% (n = 69) were male and 40% (n = 46) were female, with a mean age of 47.33 years. The most common etiology was undetermined (n = 41), followed by diabetic nephropathy (n = 30) and hypertensive nephropathy (n = 10). The most frequent anatomical sites for AVF were left brachiomedian (22.60%), left brachiocephalic (20.86%), and left radiocephalic (19.13%). Among the comorbidities in this population, the most common were systemic arterial hypertension (n = 78) and type 2 diabetes mellitus (n = 45). The average number of days from AVF creation to echocardiography was calculated to be 463.52 days. The calculated probability for suspected PH was: low probability 46.95% (n = 54), intermediate 26.95% (n = 31), high 17.39% (n = 20), and undetermined 8.69% (n = 10). These patients present with increased blood flow secondary to the AVF, which is associated with a higher risk of In our country, generating local evidence is essential to identify this complication early and improve the management of these patients.

## A169 Proteomics Defines Shared and Divergent Alterations in the Right Atrium and Right Ventricle in Porcine Right Heart Failure

Dr Sally Prins, Prof Kurt Prins^1^

^1^MUSC

Right heart failure (RHF) is a leading cause of morbidity and mortality in pulmonary arterial hypertension (PAH), and we currently lack effective therapies to combat this deadly condition. Proper right heart function is dependent on the synergistic actions of the right atrium and the right ventricle for the circulation of deoxygenated venous blood through the pulmonary vasculature and to ultimately fill the left ventricle. Recent studies demonstrate right atrial dysfunction is an emerging risk factor for heightened mortality in PAH, but the underlying pathobiology of right atrial dysfunction is poorly understood. In addition, a comparison of the chamber specific alterations in the diseased right atrium and right ventricle is lacking, which limits our ability to effectively treat both chambers to combat RHF. Crude mitochondrial enrichments (Abcam) from right atrial (n = 4 control and n = 4 PAB) and right ventricular (n = 4 control and n = 4 PAB) tissues were generated and subjected to TMT16plex proteomics. Calculated protein abundances were correlated with right ventricular ejection fraction or right atrial emptying fraction to determine which proteins were associated with right heart chamber function. In the right atrium, Wiki pathways associated with enhanced right atrial emptying fraction included the tricarboxylic acid (TCA) cycle, branched chain amino acid (valine, leucine, and isoleucine, BCAA) metabolism, and oxidative phosphorylation. In the right ventricle, higher right ventricular ejection fraction were dominated by oxidative phosphorylation, which suggested slightly different mechanisms of metabolic remodeling occurred when comparing the right atrium to the right ventricle. On the other hand, pathways associated with impaired chamber function were more concordant as ribosomal, integrin‐associated cellular adhesion, and glycolytic pathways. In conclusion, our data nominates pathways that may contribute to right heart failure via both right atrial and right ventricular compromise with shared alterations in oxidative phosphorylation, glycolysis, ribosomes, and integrin‐mediated adhesion.

## A170 Sotatercept – First Experience in Eisenmenger Patients

Prof Astrid Lammers^1^, Professor Gerhard‐Paul Diller^1^

^1^University Hospital Münster, Münster, Germany

Sotatercept, an activin signalling inhibitor, is approved for the treatment of adult patients with pulmonary arterial hypertension since 2024. Monitoring of the platelet levels as well as Hb is mandatory. Increased Hb levels have been observed which may lead to hyperviscosity syndrome and increased risk for thromboembolic events. Eisenmenger syndrom represents an extreme form of pulmonary hypertension with severe pulmonary vascular disease, which is characterized by a right‐to‐left‐shunt, hypoxaemia and erythrocytosis. Although Eisenmenger patients often survive for decades, physical exercise tolerance is very limited. Other advanced therapies for PAH have been proven to be beneficial in this patient group. We offered Sotatercept to selected Eisenmenger patients with moderate polycytaemia (Hb < 20 g/dl), no history of bleeding or thrombosis. We aimed to assess tolerability and safety. 2 patients with Eisenmenger physiology in FC 2 and 3 (both female, aged 29 and 31) have been treated with Sotatercept for 4 and 7 months respectively.

Treatment was well tolerated in both of the patients. One patient reported a temporary gingival bleed. Hb did not increase significantly in both patients, nor did we observe any other adverse serological changes. Transcutaneous oxygen saturation seemed to improve, as well as patients‘ excercise tolerance. NT‐ pro BNP decreased suggestive of less myocardial strain. Sotatercept appears to be well tolerated and leads to improved physiological markers and potential improvement of exercise tolerance, even in Eisenmenger patients. In particular Hb did not appear to raise signifincantly in this cohort with a physiologically higher baseline Hb, may be due to improved pulmonary blood flow. A planned study of Sotatercept of patients with Eisenmenger physiology with structured monitoring will hopefully reveal long‐term benefits of this therapy in this challenging patient cohort.

## A171 Evaluating the Impact of Digital Twin Forecasts on Decision‐Making in Pulmonary Arterial Hypertension

Dr Marcelle Paula‐Ribeiro^1^, Dr Ze Ming Goh^2^, Dr Mark Toshner^3^, Professor Alexander Rothman^1,2^, Professor Martin Wilkins^4^

^1^School of Medicine and Population Health, University of Sheffield, Sheffield, UK, ^2^Sheffield Pulmonary Vascular Disease Unit, Royal Hallamshire Hospital, Sheffield Teaching Hospitals NHS Foundation Trust, Sheffield, UK, ^3^Department of Medicine, Heart and Lung Research Institute, University of Cambridge, Cambridge, UK, ^4^Imperial College London, London, UK

Contemporary care of patients with pulmonary arterial hypertension requires repeated risk evaluation to guide therapeutic management. Digital Twin (DT) technologies offer the opportunity to personalise risk forecasts and simulate treatment effects. We aim to provide a first evaluation of the utility of DT forecasts in a pulmonary hypertension multidisciplinary team (MDT) decision making processes. We will perform a retrospective analysis of prospectively collected data from a phase IV randomized trial evaluating the dose–response and clinical efficacy of riociguat and selexipag using implanted technologies. At prespecified changes in treatment, a simulated MDT will be undertaken, including PH physicians, nurses, pharmacists, and imaging specialists. The MDT will be provided with anonymised patient data comprising haemodynamics (mean pulmonary arterial pressure [mPAP], cardiac output [CO], total pulmonary resistance [TPR]), cardiac MRI parameters and guideline‐based risk score. The MDT will first assign a risk estimate and record an initial management plan (Plan A) along with confidence rating and an expected change in clinical measures. Subsequently, the DT forecast will be disclosed, showing patient‐specific forecasts of haemodynamic trajectories (mPAP, CO, TPR), one‐year mortality risk, and simulated outcomes under alternative therapeutic strategies. The MDT will then provide an updated management plan (Plan B) and re‐rate confidence/expected benefit. All decisions will be documented, and MDT members will justify their confidence ratings.

Endpoints: Accuracy of the DT will be determined by accuracy of prediction of mPAP, CO and risk score. The utility of the DT will be evaluated by assessing confidence in MDT decisions and the proportion of cases where the management strategy changes after DT output disclosure. This evaluation will assess whether DT forecasts facilitate MDT‐guided patient management and improve decision confidence and guideline alignment. The proposed design provides a robust framework for safe, evidence‐based evaluation of DT technology in PAH management.

## A172 A Roadmap for Patient Digital Twins in Pulmonary Arterial Hypertension

Professor Steven Niederer^1,2^, Dr Maximilian Balmus^1,2^, Dr Marcelle Paula‐Ribeiro^3^, Dr Ze Ming Goh^4^, Dr Christopher Burr^1^, Professor Timothy Chico^5,6^, Professor Richard Clayton^7^, Dr Wenjia Bai^2^, Dr Camila Rangel‐Smith^1^, Professor David Wagg^1,5^, Dr Richard Wilkinson^8^, Sheng‐Ya Wang^2^, Professor Alexander Rothman^3,4^, Professor Martin Wilkins^2^

^1^The Alan Turing Institute, London, UK, ^2^Imperial College London, London, UK, ^3^School of Medicine and Population Health, University of Sheffield, Sheffield, UK, ^4^Sheffield Pulmonary Vascular Disease Unit, Royal Hallamshire Hospital, Sheffield Teaching Hospitals NHS Foundation Trust, Sheffield, UK, ^5^University of Sheffield, Sheffield, UK, ^6^British Heart Foundation Data Science Centre, UK, ^7^School of Computer Science and Insigneo Institute, University of Sheffield, Sheffield, UK, ^8^University of Nottingham, Nottingham, UK

Pulmonary arterial hypertension (PAH) is a progressive disease with limited survival despite therapeutic advances. Current management relies on episodic clinic visits, invasive procedures, and composite risk scores, providing only a partial view of disease trajectory. Patient digital twins (PDTs) offer a new paradigm by creating continuously updated, predictive representations of individual patients through the integration of mechanistic models, data‐driven approaches, and longitudinal patient information. We propose a roadmap for PDT development in PAH. The process begins with nominal cardiopulmonary models, constructed from established physiological principles and population‐level data, to capture core cardiovascular and pulmonary dynamics. When patients first present, these models are personalised using clinical measurements, imaging, haemodynamics, data from implanted sensors or wearables, and patient‐reported outcomes. Over time, each twin is continuously refined as new data are incorporated, producing a digital thread—a longitudinal record of forecasts, model states, and clinical decisions. Scaling across individuals generates a population of twins, enabling virtual cohorts, cross‐patient comparisons, and quantification of uncertainty.

Delivering PDTs in practice requires engineering solutions, including automated calibration to streamline personalisation, surrogate modelling to reduce computational cost, and secure workflows for integrating diverse clinical and community data. For clinical translation, we outline an evaluation strategy based on retrospective analysis of prospectively collected trial data, in which multidisciplinary teams record an initial management plan, reassess after disclosure of DT forecasts, and the impact is measured by accuracy of predictions, changes in management strategy, and decision confidence. Ultimately, the realisation of PDTs offers a framework that integrates patient data with physics‐ and physiology‐based models, enhancing patient monitoring, improving prediction, and enabling more personalised care in PAH.

## A173 AI‐Based Measurement of Pulmonary Artery to Ascending Aorta Ratio on Unenhanced CT for Pulmonary Hypertension: Diagnostic Accuracy of Diameter Vs. Volume

Mr Turki Nasser Alnasser^1,2,3^, Dr Alireza Hokmabadi^1,4^, Mr Khalid Alghamdi^1^, Mr Michael Sharkey^1,5^, Dr Krit Dwivedi^1,6^, Dr Smitha Rajaram^6^, Dr Christopher Johns^1,6^, Prof David G Kiely^1,4,7,8^, Dr Samer Alabed^1,4,6,7^, Prof Andrew J Swift^1,4,6,7^

^1^School of Medicine & Population Health, The University of Sheffield, Sheffield, United Kingdom, ^2^Radiological Sciences Department, College of Applied Medical Science, King Saud bin Abdulaziz University for Health Science, Riyadh, Saudi Arabia, ^3^King Abdullah International Medical Research Center (KAIMRC), Riyadh, Saudi Arabia, ^4^Insigneo Institute, Faculty of Engineering, The University of Sheffield, Sheffield, United Kingdom, ^5^3D Imaging Lab, Sheffield Teaching Hospitals NHSFT, Sheffield, United Kingdom, ^6^Department of Clinical Radiology, Sheffield Teaching Hospitals, Sheffield, United Kingdom, ^7^National Institute for Health and Care Research, Sheffield Biomedical Research Centre, Sheffield, United Kingdom, ^8^Sheffield Pulmonary Vascular Disease Unit, Sheffield Teaching Hospitals NHS Trust, Sheffield, United Kingdom

The aim of this study is to assess the diagnostic accuracy of a deep learning (DL) model for quantifying the pulmonary artery to ascending aorta ratio (PA/AAo), based on diameters and volumes, in detecting pulmonary hypertension (PH), compared with right heart catheterisation (RHC) and manually measured ground truth. A DL model was developed using unenhanced CT scans to automatically segment the pulmonary artery and ascending aorta. PA/AAo ratios were quantified using two post‐processing approaches: diametric and volumetric measurements. In a cohort of 97 patients, agreement between DL‐derived and manually obtained PA/AAo ratios, independently assessed by two observers, was evaluated using intraclass correlation (ICC). Within the same cohort, the diagnostic accuracy of both measurement approaches and manual measurements was determined against RHC. In an independent cohort of 425 patients (385 internal and 40 external), performance of the DL‐derived measurements was further assessed against RHC using diagnostic metrics, including sensitivity, specificity, positive and negative predictive values (PPV and NPV), and area under the curve (AUC). Inter‐observer agreement was excellent (ICC = 0.94 [0.91–0.96]). Agreement with the diameter‐based approach was good (Observer 1: ICC = 0.79 [0.70–0.85]; Observer 2: ICC = 0.79 [0.70–0.86]). Agreement with the volume‐based approach was moderate (Observer 1: ICC = 0.56 [0.41–0.68]; Observer 2: ICC = 0.55 [0.39–0.67]). The DL diameter‐based approach (AUC = 0.83) and volume‐based approach (AUC = 0.84) both outperformed manual measurements (Observer 1: AUC = 0.72; Observer 2: AUC = 0.74). In the 425‐patient cohort, the volume‐based approach achieved superior diagnostic accuracy compared with the diameter‐based approach (AUC 0.85 vs 0.73), with higher sensitivity (66% vs 41%) and specificity (89% vs 82%).

The diameter‐based approach showed higher correlation with manual measurements, whereas the volume‐based approach demonstrated greater diagnostic accuracy for PH diagnosis when benchmarked against RHC.

## A174 A Patient Perspective on Pulmonary Hypertension Monitoring

Dr Jamie Ingram^1^, Mrs Agnes Crozier^1^, Mr Jim Mearns^1^, Mr Andy Kerr^1^, Mrs Sarah Cooper^1^, Dr Martin Johnson^1^, Dr Melanie Brewis^1^, Dr Colin Church^1^

^1^Scottish Pulmonary Vascular Unit, Glasgow, Scotland

Pulmonary hypertension (PH) is a life‐shortening disease requiring close follow‐up to ensure treatment optimisation. Current guidelines recommend 3‐6 monthly review in stable patients. Cardiac MRI (CMRI) and right heart catheterisation (RHC) have recently shown added prognostic value in follow‐up risk stratification. We sought to explore the patient perspective on these investigations, alongside other routine follow‐up tests. A questionnaire was offered to all prevalent patients with pre‐capillary PH at the Scottish Pulmonary Vascular Unit outpatient clinic over a 4‐month period during 2024. Common PH investigations were scored out of 5 on a Likert scale with ‘5’ suggesting patients are ‘very happy’ to have that investigation. 180 patient responses were received. Age range 19‐86 (mean 62). The majority would undergo CMRI (89.9%) and RHC (68.6%) annually if recommended by their specialist. Percentages were similar when asked if patients would have these tests ‘at any point’ during follow‐up instead of annually. Patients favour PH specialist review around twice a year (mean 6.9 months, median 6 months). Mean scores out of 5 for individual investigations were: RHC 3.44/5, CMRI 4.14/5, CPET 3.37/5, 6MWT 4.02/5, Blood tests 4.65/5, CT 4.41/5, ECHO 4.50/5, ECG 4.49/5, PROs 4.40/5. Median scores were 5 for all investigations except RHC and CPET which scored 4. The most divisive tests were RHC, CPET and 6MWT, with percentages of patients rating these as less than 3 out of 5 being 29%, 37% and 17% respectively. Patients prefer 6‐monthly follow‐up with non‐invasive and non‐exertional tests. The majority of patients are willing to have regular CMRI and RHC but around one‐third would not want invasive haemodynamic reassessment. For those patients a CMRI may be a more accepted contribution to follow‐up risk profiling.

## A175 Real‐World Experience With Sotatercept: A Tale of Two Cities

Dr Naomi Habib^1^, MD Hamna Javed, MD Vijay Balasubramanian

^1^Norton Thoracic Institute, Phoenix, United States of America

Sotatercept is a first‐in‐class activin signaling inhibitor that was recently approved for the treatment of Group 1 pulmonary arterial hypertension. Mounting evidence has demonstrated the efficacy and safety of the drug within the context of clinical trials; however there remains a gap in real‐world evidence and experience. To this end, we describe a series of patients from 2 pulmonary hypertension centers in the western United States. We retrospectively reviewed cases of sotatercept initiation occurring in 2 large pulmonary hypertension centers in Fresno, California and Phoenix, Arizona. We evaluated patient demographics, hemodynamics, background therapies, as well markers of risk. In several cases patients also had repeat right heart catheterization which were also reported. Twenty‐four patients were evaluated. The age range of patients was between 23‐84, with a mean of 54. The group was largely female (17/24), with the predominant ethnicity white (15/24) and Hispanic (7/24). The majority of PAH was categorized as idiopathic (13/24), followed by connective tissue disease‐related (6/24) and methamphetamine‐related (4/24). The patients represented a range of risk categories with 6 low‐risk, 9 intermediate‐risk, and 9 high‐risk by REVEAL Lite 2.0. All patients but 1 were on background combination therapy, with 8 on dual, 15 on triple, and 11 on background parenteral prostacyclin. Of the 24 patients, only 3 required therapy discontinuation, 2 due to prohibitive thrombocytopenia and 1 due to severe epistaxis. Repeat hemodynamics in 7 cases demonstrated a mean 37% drop in pulmonary vascular resistance (PVR) and 58% increase in pulmonary arterial compliance (PAC). Cardiac output (CO) remained stable with a slight 1.3% increase across cases. Our series confirms real‐world efficacy and safety of sotatercept. No pericardial effusions were encountered. Repeat hemodynamics showed major improvement in PVR and PAC, with a stable CO in keeping with previous trials.

## A176 Pulmonary Arterial Hypertension in Transition: Age Shift and Survival Stratified by Age in the Giessen PH Registry

Dr Patrick Janetzko^1,2,3^, Jeeva Sam^1,2,3^, Meike Fünderich^1,2,3^, MD Nils Kremer^1,2,3^, MD Bruno Thal^1,2,3^, MD Zvonimir A. Rako^1,2,3^, MD, PhD Friedrich Grimminger^1,2,3^, MD Hossein A. Ghofrani^1,2,3^, MD Khodr Tello^1,2,3^, MD Werner Seeger^1,2,3^

^1^Universities Of Giessen And Marburg Lung Center (UGMLC), Giessen, Germany, ^2^Member of the German Center for Lung Research (DZL), Giessen, Germany, ^3^Institute for Lung Health (ILH), Cardio‐Pulmonary Institute (CPI), Giessen, Germany

Over the past decades, Group 1 pulmonary arterial hypertension (PAH) has evolved from a relatively homogeneous disorder predominantly affecting younger women to a markedly heterogeneous disease with a substantially higher median age at diagnosis. Corresponding trends have been observed in several large multicenter registries, reflecting a global shift in the epidemiology of PAH.

In this retrospective study, we confirm these findings in a large monocentric cohort from the Giessen PH Registry, comprising more than 5,000 patients. Our monocentric design offers the advantage of uniform diagnostic and therapeutic strategies, minimizes inter‐center variability, and ensures consistent long‐term follow‐up. Within the PAH subgroup of our registry, the median age at diagnosis increased from 44 years (1990–2000) to 62 years (2010–2020), illustrating the demographic transition in PAH over time. To assess the impact of this age‐related evolution on patient outcomes, we conducted a survival analysis across predefined age strata. Kaplan–Meier estimates revealed a distinct age‐related separation in long‐term survival, with 5‐, 10‐, and 15‐year survival rates of 72.9%, 59.9%, and 47.7% for patients < 45 years, 55.5%, 37.4%, and 30.9% for those aged 50–65 years, and 38.8%, 16.7%, and 3.5% for patients > 65 years, respectively. To elucidate the mechanisms underlying this divergence in mortality, we present analyses exploring factors such as comorbidities, sex differences, and therapeutic response. Hemodynamic follow‐up data obtained by right heart catheterization were included and stratified according to the applied PAH‐specific treatment regimen. Our data indicate that the progressive aging of the PAH population translates into a significant survival gradient and altered therapeutic outcomes across age groups. These findings emphasize the need for age‐tailored management strategies and underscore the importance of systematically including elderly patients in future clinical trials.

## A177 Role of Matrisome in Right Ventricular Stiffness and Dysfunction in Pulmonary Hypertension

Dr Peng Zhang^1,2^, PharmD Eric Wang^1^, Assistant Professor Richard Clements^3^, Assistant Professor Reza Avazmohammadi^4^, Professor Gaurav Choudhary^1,2^

^1^Providence VA Healthcare System, Providence, United States, ^2^Alpert Medical School of Brown University, Providence, United States, ^3^University of Rhode Island, Kingston, United States, ^4^Texas A&M University, College Station, United States

Increased right ventricular (RV) stiffness in pulmonary hypertension (PH) results in systolic and diastolic RV dysfunction. However, the mechanisms and extracellular matrix (ECM) remodeling underlying RV stiffness are poorly understood. This study was designed to determine the effect of PH on RV stiffness and function and alterations of RV matrisome that can regulate RV stiffness.

Following models were used: SU5416‐Hypoxia (SuHx) in Sprague–Dawley (SD) rats (4 and 7 weeks timepoints, n = 6 each) and Fischer rats (4 weeks timepoint, n = 6 each) and respective control rats (n = 6 each), and sham‐operated and pulmonary artery banding (PAB) rats (7 weeks, n = 6 each). Assessments included echocardiography, hemodynamics, and stiffness measurements, matrisome analysis, and snRNAseq from RV tissue. Matrisome analysis and snRNAseq were also performed using human RV tissues from transplant patients with or without RV dysfunction (n = 36). The PH models developed RV stiffness and dysfunction, which were more severe in the PH Fischer rats and was associated with reduced ventricular‐arterial (V‐A) coupling. Matrisome analysis from all the models showed an increase in collagen expression (Col1, Col2, Col3, Col4, Col5, Col6, Col8, Col14) that contributes to RV stiffening. The ECM proteins that critically regulate ECM structure, microfibrils, and cell adhesion (TGFBI, ECM1, POSTN, LTBP2. LTBP4, TGM2, THBS4, TNXB) are significantly upregulated. snRNAseq showed many of these ECM proteins and their regulators (e.g., Col14, Col6, ECM1, LTBP2) are predominantly produced by cardiac fibroblasts. Similar alterations in matrisome and transcriptome were observed in human dysfunctional RV tissues. Our data suggest that dynamic changes in ECM and ECM‐associated proteins likely determine RV stiffness and dysfunction in PH. Changes in ECM structure and its interaction with cells may play a more important role than ECM deposition alone. Targeting matrisome may be a novel strategy to reduce RV stiffness and improve RV function in settings of PH.

## A178 CS014, a Novel Precision Deuterated VPA with HDAC Inhibitor Properties, Demonstrates Preclinical Efficacy in Pulmonary Vascular Disease and Clinical Safety

Dr Tatiane Abreu Dall'agnol^1^, Dr Fredrik Frick^1^, Dr Rahul Agrawal^1^, Dr Björn Dahlöf^1^, Dr Johan Nilsson^2^, Dr Johan Bylund^2^, Mr Erik Westrin^2^, Dr Nicholas Oakes^1^

^1^Cereno Scientific, Gothenburg, Sweden, ^2^CTC, Uppsala, Sweden

CS014, a novel precision deuterated valproic acid (VPA) and histone deacetylase (HDAC) inhibitor is in development for rare pulmonary diseases characterized by vascular remodeling and fibrosis. These include idiopathic pulmonary fibrosis (IPF), pulmonary hypertension associated with interstitial lung disease (PH‐ILD) and pulmonary arterial hypertension (PAH). In nonclinical studies, PAH was induced in rats by a single dose of Sugen followed by 3 weeks of hypoxia and then 3 weeks of normoxia. Animals were treated orally with Vehicle or CS014 (20–300 mg/kg/day). In a first‐in‐human (FIH) trial, healthy adults (34–65 years) were assigned to single oral doses of CS014 (100, 300, 800, 1400, or 2000 mg; n = 6/cohort) or multiple doses (600, 900, 1200 mg once daily for 7 days; n = 6/cohort).

In the rat model, CS014 induced a robust, dose‐dependent amelioration of vascular remodeling with reduced arteriolar vessel occlusion, reductions of intimal proliferation (including plexiform lesion) and fibrosis. Effects occurred at daily unbound exposures of 33.3–68 μg/mL·hr. In the FIH trial, CS014 was safe and well‐tolerated at all doses, exhibiting dose‐dependent exposure. In the multiple‐dose cohorts, estimated steady‐state unbound AUCτ values were 85.5 μg/mL·hr at 600 mg, 103 μg/mL·hr at 900 mg, and 334 μg/mL·hr at 1200 mg/day. Adverse reactions (ARs) were all mild and fully resolved. Among multiple‐dose participants, 6/18 (33%) experienced ARs, most commonly gastrointestinal disorders (5/18, 28%). The most frequent ARs among the SAD cohorts were headache (6/30, 20%) and fatigue (2/30, 6.7%); while the most frequent AR in the MAD part was nausea (2/18, 11%). CS014 reduced fibrosis and dose‐dependently improved pulmonary arteriolar remodeling, including plexiform lesions, in the Sugen/hypoxia rat model of PAH. The results suggest that unbound exposures required for these effects were safe and well‐tolerated in healthy volunteers, supporting further development in severe cardiovascular and pulmonary diseases.

## A179 Selective Pulmonary Vasodilation by Intravenous Pdno in Patients With Pulmonary Hypertension After Cardiac Surgery ‐ Results From an Open‐Label, Multicentre Study to Evaluate the Dose, Efficacy, Safety and Tolerability of PDNO

Dr Kristofer Nilsson^1^, Dr Christofer Adding^2^, Dr Jenny Seilitz^1^, Dr Tor Damen^3^, Dr Andreas Nygren^3^, Dr Per Agvald^4^, Dr Sven‐Erik Ricksten^3^

^1^Department of Cardiothoracic and Vascular Surgery, Faculty of Medicine and Health, Örebro University, Örebro, Sweden, ^2^Department of Molecular Medicine and Surgery, Section of Urology, Karolinska Institute, Stockholm, Sweden, ^3^Department of Anesthesiology and Intensive Care, Sahlgrenska University Hospital, Gothenburg, Sweden, ^4^Department of Physiology and Pharmacology, Karolinska Institute, Stockholm, Sweden

Acute pulmonary hypertension (aPH) with right ventricular failure after cardiac surgery with cardiopulmonary bypass (CPB) is a life‐threatening condition with limited treatment options. Current intravenous vasodilators lack pulmonary selectivity and cause systemic hypotension. PDNO, a new nitric oxide–donating iv. prodrug, has shown selective pulmonary vasodilation in preclinical studies and safety in Phase I. This Phase IIa evaluated the potential pulmonary selective vasodilatory effect in patients with aPH after mitral valve surgery. This multicenter, open‐label “first in patient” trial enrolled adults with aPH following CPB for mitral valve surgery. In the ICU, intubated patients received incremental doses of PDNO (2.5–60 nmol/kg/min) with invasive systemic and pulmonary hemodynamic measurements (PAC, CVC, and radial artery catheter). The primary endpoint was change in pulmonary vascular resistance (PVR); secondary endpoints included mean pulmonary artery pressure (mPAP), pulmonary capillary wedge pressure (PCWP), cardiac output (CO), PVR/SVR ratio, and safety. PDNO produced a clear dose‐dependent reduction in PVR, the mean relative change from baseline ranged from ‐6.73% to ‐44.9% in the dose range 2.5‐30 nmol/kg/min, driven by decreases in mPAP and increases in CO, while PCWP decreased. There was a large inter‐individual dosing‐rate variability where PDNO had a selective pulmonary vasodilator effect (decreased PVR/SVR ratio) in 11 of 12 patients. Pooled data showed a significant reduction of the PVR/SVR‐ratio at 5 nmol/kg/min (p = 0.008) and 10 nmol/kg/min (p = 0.026). PDNO was well tolerated with no unexpected safety concerns. Intravenous PDNO was safe, well tolerated, and produced dose‐dependent pulmonary vasodilation with preserved systemic pressure. Individual dose‐titration of PDNO in the dose range 2,5‐20 nmol seem to be optimal in most patients. Reductions in mPAP, PVR, PVR/SVR ratio along with improved cardiac output support PDNO as a promising intravenous selective pulmonary vasodilator therapy for postoperative acute pulmonary hypertension.

## A180 Artificial Intelligence(AI)‐Facilitated Analysis of Single‐Image Tissue Doppler Signal to Characterize Right Ventricular Dysfunction

Xin Tan^2^, MD Akila Bersali^1^, MD Sadeer Al Kindi^1^, MD Sandeep Sahay^1^, Katelyn Ingram^1^, PhD Meng Li^2^, Dr Ashrith Guha^1^

^1^Houston Methodist Hospital, Houston, 77401, ^2^Rice University, Houston, United States

Quantitative assessment of right ventricular (RV) function by transthoracic echocardiogram (TTE) commonly relies on tricuspid annular plane systolic excursion (TAPSE) and lateral tricuspid annulus peak systolic velocity (S’). However, full cardiac cycle data may provide additional information beyond these two systolic measures. We sought to (1) automate the estimation of systolic parameters (TAPSE and S’) from tissue spectral Doppler imaging (TDI) and (2) integrate these tabular systolic parameters and the full‐cycle functional signal to estimate RV systolic function. We identified 387 patients who underwent both TTE and cardiac magnetic resonance imaging (CMR) within 24 hours. We developed an automated algorithm to extract TAPSE and S’ from raw TDI and validated its outputs against clinician measurements. We trained 2 classifier models for RV dysfunction (RVEF < 45%): (1) Tabular model (RVDTABULAR) using algorithmic measurement of TAPSE/S’ and age/sex, and (2) Integrated model (RVDINTEGRATED) based on an attention‐based neural network model using the entire digitized TDI waveforms in addition to tabular data. In the TTE‐CMR paired dataset, the proposed algorithm accurately estimated S’ (mean error: –0.05 cm/s) and TAPSE (mean error: –0.97 mm). Tabular model RVDTABULAR achieved an AUROC of 0.71 and an AUPRC of 0.48 for predicting RVEF < 45%. The fully combined attention‐based neural network model, which incorporated waveform trajectories, systolic parameters, and demographics, significantly improved performance (AUROC: 0.768; AUPRC: 0.56). In the external validation cohort of 88 patients with pulmonary hypertension, the integrated model's prediction of RVEF < 45% was significantly associated with event‐free survival (p = 0.036). We developed a fully automated pipeline that integrates digitized TDI waveforms with both parametric and non‐parametric features to classify RVEF < 45%. This approach can effectively risk‐stratify patients with pulmonary hypertension.

## A181 Pulmonary Tumor Thrombotic Microangiopathy: A Retrospective Review of Seven Cases

Dr Keiichiro Kuronuma^1^, MD Hiroto Shimokawahara^1^, MD Hiromi Matsubara^1^

^1^NHO Okayama Medical Center, Okayama, Japan

Pulmonary tumor thrombotic microangiopathy (PTTM) is a critical complication of malignancy characterized by rapidly progressive pulmonary hypertension (PH). The pathophysiology involves obstruction of pulmonary arterioles by tumor microemboli, activation of thrombogenic cascades, and vascular remodeling driven by tumor‐derived growth factors such as platelet‐derived growth factor and vascular endothelial growth factor. Diagnosis during life is difficult, and untreated patients usually die within days to weeks from symptom onset. Although case reports suggest that chemotherapy may improve survival, the overall prognosis remains poor. The aim of this study was to retrospectively review seven cases of PTTM from our institution. Patients were aged 30‐68 years and presented primarily with dry cough, dyspnea, or syncope. Right heart catheterization confirmed PH in all patients (mean pulmonary artery pressure 36‐53 mmHg). Primary malignancies consisted of gastric adenocarcinoma, lung squamous cell carcinoma, and cervical cancer; the origin was unknown in four other cases. Diagnosis was made by pulmonary artery wedge aspiration cytology or transbronchial lung biopsy in some patients, while others were diagnosed at autopsy. One patient with gastric adenocarcinoma demonstrated hemodynamic improvement with imatinib, allowing withdrawal of extracorporeal support and survival for 17 months after symptom onset. Another patient achieved temporary disease control with chemotherapy including cisplatin (CDDP)‐based chemotherapy for signet‐ring cell like adenocarcinoma of unknown origin, surviving 21 months from symptom onset. The remaining five patients deteriorated rapidly despite chemotherapy or supportive care, with survival ranging from 10 days to 5 months after initial symptoms. PTTM remains an under‐recognized, rapidly progressive cause of PH with extremely poor outcomes. Chemotherapy may transiently improve clinical status in selected cases. Early recognition, proactive diagnostic approaches, and timely initiation of targeted therapy may provide short‐term benefit. Further accumulation of cases and prospective studies are needed to clarify optimal diagnostic and therapeutic strategies.

## A182 Glucose Utilization by Lung Interstitial Macrophages in Murine Hypoxia‐Induced Pulmonary Hypertension

Mr Kevin Nolan^1,2^, Dr. Claudia Mickael^4^, Dr. Rahul Kumar^1,2,3^, Dara C. Fonseca Balladares^1,2^, Annika Bai^1,2,5^, Dr. Rubin M. Tuder^4^, Dr. Brian B. Graham^1,2^, Dr. Michael H. Lee^1,2^

^1^Division of Pulmonary and Critical Care Medicine, San Francisco, United States, ^2^Lung Biology Center, Department of Medicine, San Francisco, United States, ^3^Genentech, South San Francisco, United States, ^4^Division of Pulmonary Sciences and Critical Care Medicine, Aurora, United States, ^5^University of California Santa Barbara, San Barbara, United States

Hypoxia inducible factor‐1 (HIF‐1) has been shown to promote various forms of pulmonary hypertension (PH) through increased glycolysis, among other mechanisms. Although Hif1α and glycolytic gene deletion in the vascular constituents (endothelial cells, smooth muscle cells) reduced PH severity in animals, the role of Hif‐1α in inflammatory cells like macrophages remains uncharacterized, particularly in the context of hypoxia‐induced PH (Hx‐PH). Macrophage subpopulations have been described, with discreet functions, including CX3CR1 positive, FOLR2 positive resident interstitial macrophages (IMs). We hypothesized that Hif‐1α and glucose utilization by lung IMs would attenuate Hx‐PH. As a proxy for HIF‐1‐induced glycolysis, we first measured glucose uptake in lung IMs, by intraperitoneally injecting wildtype mice exposed to 2‐day hypoxia with 2‐NBDG (fluorescent glucose analogue) immediately prior to sacrificing and measuring its intensity with flow cytometry. We also deleted HIF‐1α in fractalkine receptor (CX3CR1)‐positive monocytes and resident IMs in CX3CR1CreER x HIF‐1αLoxP/LoxP (HIF‐1α∆CX3CR1) mice. Prior to hypoxia exposure, the HIF‐1α∆CX3CR1 mice received either tamoxifen or corn oil (vehicle control), with tamoxifen‐injected HIF‐1α‐floxed mice (without Cre), serving as a second control group. PH severity was quantified with right heart catheterization. After hypoxia exposure, we observed increased 2‐NBDG uptake by all three subsets of lung IM: FOLR2‐positive, C‐C chemokine receptor 2 (CCR2)‐positive, and MHCII‐high lung IMs as compared to normoxic controls. Of these, FOLR2‐positive IMs demonstrated greatest avidity for 2‐NBDG, suggesting that increased glycolysis by resident lung IMs may critically contribute to Hx‐PH. Deleting HIF‐1α in CX3CR1‐positive monocytes and resident IMs, however, did not protect the mice from PH. The lack of protection against Hx‐PH suggests:1) the dispensable nature of HIF‐1α in resident lung IMs; 2) compensatory changes in other metabolic pathways in response to HIF‐1α deletion, and/or 3) a critical role of HIF‐1α biology in recruited lung IMs in the context of Hx‐PH.

